# A glycan foldamer that uses carbohydrate–aromatic interactions to perform catalysis

**DOI:** 10.1038/s41557-025-01763-6

**Published:** 2025-02-26

**Authors:** Kaimeng Liu, Martina Delbianco

**Affiliations:** Department of Biomolecular Systems, Max Planck Institute of Colloids and Interfaces, Potsdam, Germany

**Keywords:** Carbohydrate chemistry, Supramolecular chemistry, Catalyst synthesis

## Abstract

In nature, the ability to catalyse reactions is primarily associated with proteins and ribozymes. Inspired by these systems, peptide-based catalysts have been designed to accelerate chemical reactions and/or ensure regio- and stereoselective transformations. We wondered whether other biomolecules (such as glycans) could be designed to perform catalytic functions, expanding the portfolio of synthetic functional oligomers. Here we report a glycan foldamer inspired by the natural Sialyl Lewis X antigen that acts as catalyst in a chemical reaction. This glycan-based catalyst benefits from structural rigidity and modular adaptability, incorporating a substrate-recognition motif alongside a catalytic active site. Leveraging the inherent ability of carbohydrates to engage in CH–π interactions with aromatic substrates, we demonstrate the recruitment and functionalization of a tryptophan via a Pictet–Spengler transformation. Our modular glycan catalyst accelerates the reaction kinetics, enabling the modification of tryptophan-containing peptides in aqueous environments. Our findings pave the way for the development of glycan-based catalysts and suggest the possibility of catalytic capabilities of glycans in biological contexts.

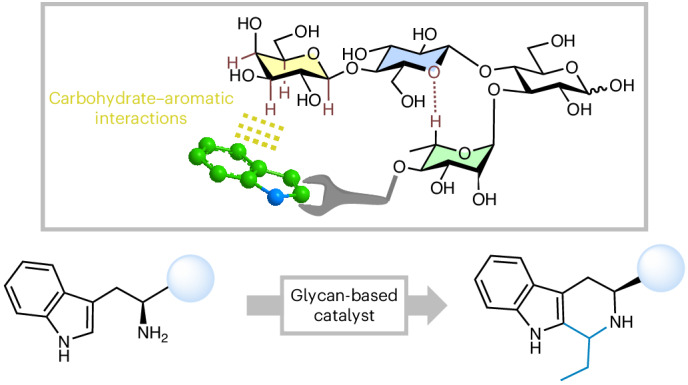

## Abstract (web summary)

In nature, catalysis is generally performed by proteins and ribozymes. Now it has been shown that glycans can be designed to perform catalysis. Exploiting carbohydrate–aromatic interactions, a glycan foldamer enables a Pictet–Spengler transformation of tryptophan and tryptophan-containing peptides in water.

## Main

**Fig. 1:**
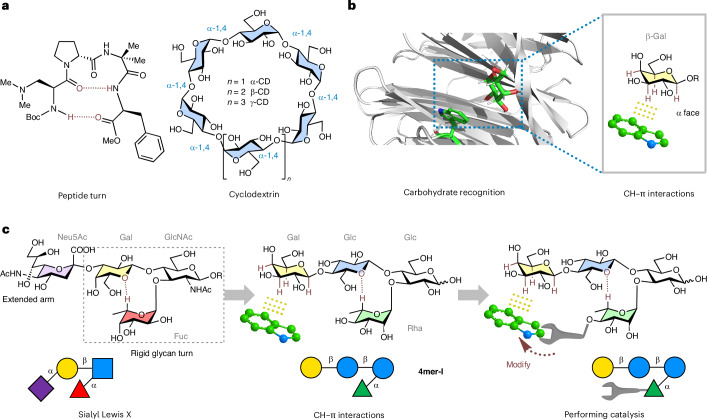
Design of a functional glycan foldamer. **a**, Examples of folded oligomers used in catalysis. **b**, A visualization of carbohydrate–aromatic interactions between a Trp residue and a Gal involved in carbohydrate recognition (PDB ID: 5ajb). **c**, The chemical structure of Sialyl Lewis X and its modification to access a glycan foldamer that performs catalysis. GlcNAc, blue square; Glc, blue circle; Gal, yellow circle; Neu5Ac, purple rhombus; Fuc, red triangle; Rha, green triangle. The monosaccharide residues are represented following the Symbol Nomenclature for Glycans (SNFG)^67^.

Folding endows biomolecules with functions. In proteins, the strategic proximity of chemical functionalities achieved through specific geometric arrangements facilitates chemical reactions by enhancing kinetics and ensuring regio- and/or stereoselectivity^1,2^. Inspired by nature, miniaturized analogues of these biomolecules have been created, such as peptide turns for stereoselective catalysis (Fig. 1a)^3–6^ and synthetic foldamers to accelerate chemical transformations^7,8^. This process not only introduced novel synthetic methodologies^5,6,9–13^ but also served as impetus for the development of efficient syntheses of foldamer motives^14–17^ and pushed the boundaries of structural analysis^18,19^. These folded molecules share a common design principle: a programmable backbone forming a stable three-dimensional conformation sustained by intramolecular non-covalent interactions that can be systematically adjusted to tune specific interactions with the substrate(s) and/or reaction intermediate(s). We wondered whether a similar design can be applied to other biopolymers, such as glycans, to expand the portfolio of functional oligomers.

Glycans offer a pool of over 100 monosaccharides, intrinsic chirality and several options for directional functionalization, making them prime candidates for crafting modular, functional oligomers^20^. Moreover, glycans can engage in multiple interactions with a substrate due to synergies of hydrogen bonding and hydrophobic effects^21^. It is therefore not a surprise that the term artificial enzyme was first coined for a cyclodextrin (CD)-based structure^22^, a cyclic α-1,4 linked oligomer of glucose (Glc) (Fig. 1a). CDs leverage the amphiphilic nature of carbohydrates to form a hydrophobic cavity that can encapsulate guest molecules, while exposing the hydrophilic hydroxyls to the solvent. These groups can be further functionalized^23,24^ to achieve a broad spectrum of functions, including catalysis^25–30^. Since their first discovery in 1891 (ref. ^31^), the chemical space of CDs has been extensively explored, resulting in the smallest known CDs^32^ and enantiomeric l-CDs^33^. Still, the functional carbohydrate space remained locked within the cyclic α-1,4 Glc framework.

Given the success of peptide-based catalysis and the unexplored potential of functional glycans, we set out to identify a glycan sequence capable of performing a catalytic reaction. This challenge was envisioned to push the boundaries of glycan synthesis and structural analysis, while increasing our understanding of carbohydrate interactions with other molecules. Here we present the design, synthesis and structural analysis of a carbohydrate sequence capable of (1) folding into a defined conformation, (2) coordinating a substrate via weak carbohydrate–aromatic interactions and (3) performing a catalytic transformation.

## Results and discussion

### General design

To craft our glycan catalyst, we sought inspiration from nature, beginning with an exploration of the diverse ways that glycans can coordinate substrates. In the carbohydrate-binding domains of proteins, aromatic moieties are abundant, facilitating binding via CH–π interactions (Fig. 1b)^34–36^. These interactions, involving the electrostatic attraction between the π electron cloud of an aromatic ring and the electron-poor C–H bonds of the carbohydrate ring, are fundamental for carbohydrate recognition in aqueous environments, as demonstrated with monosaccharide models and computational calculations^35,37^. CH–π interactions occur preferentially at the α-face of pyranosides, where the majority of axial C–H bonds are present^38^, and were exploited in artificial systems for glycan recognition^39,40^. Our strategy looked at CH–π interactions from the opposite perspective: we aimed to harness CH–π interactions to recruit an aromatic substrate and position it in proximity to the catalytic site of our glycan catalyst.

To design the backbone of our glycan catalyst, the naturally occurring Sialyl Lewis X^41^ served as the blueprint (Fig. 1c). Sialyl Lewis X is a glycan frame that possesses an extended sialyl acid (*N*-acetylneuraminic acid, Neu5Ac) arm connected to a rigid glycan turn (Gal–GlcNAc–Fuc, where Gal is galactose, GlcNAc is *N*-acetylglucosamine and Fuc is fucose), sustained by an unconventional H-bonding^42^. In our adaptation, the Neu5Ac unit was replaced with a β-linked Gal unit to form CH–π interactions and recruit an aromatic substrate (Fig. 1c). The β-Gal is particularly suited for this role owing to its three axial and one equatorial C–H bonds on the α-face, making it ideal for engaging in CH–π interactions^35^. Further modifications to the Sialyl Lewis X frame were made to optimize the glycan structure for its catalytic role: (1) the Gal unit was replaced by a Glc, avoiding multiple interaction sites (that is, two Gal units) with the aromatic substrate and orienting the α-face of the terminal Gal towards the inside of the glycan foldamer; (2) the Fuc unit was exchanged with rhamnose (Rha), featuring an equatorial hydroxyl group at C-4 for installation of the catalytic group in proximity to the key interaction site; (3) the branching GlcNAc was converted into a Glc, easing the synthetic steps, while preserving a similar folding behaviour^43,44^.

### Using CH–π interactions to recruit an aromatic substrate

**Fig. 2:**
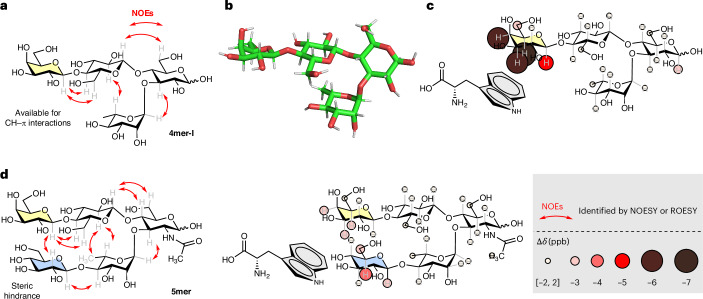
Characterization of 4mer-I, conformation and interactions with an aromatic substrate. **a**, Experimentally observed nuclear Overhauser effects (NOEs) extracted from NOESY NMR experiments for **4mer-I** (red arrows) confirming the rigid conformation of the turn motif and the orientation of the Gal arm with the α-face pointing towards the hydroxyl group of Rha C-4. **b**, A representative snapshot of **4mer-I** obtained from MD simulations. **c**, Experimentally observed CH–π interactions for **4mer-I** (3.5 mM) incubated with Trp (10.5 mM) indicating CH–π interactions localized on the Gal unit of **4mer-I**. **d**, Experimentally observed NOEs extracted from ROESY NMR experiments for **5mer** confirming its folded conformation and CH–π interactions for **5mer** (3.5 mM) incubated with Trp (10.5 mM); only minimal interactions are detected on the Gal (yellow) and Glc (blue) residues.

The initial target glycan frame (**4mer-I**) was prepared by automated glycan assembly (AGA) (Supplementary Information section 3.5.2) to study its conformation and ability to engage in CH–π interactions with an aromatic substrate. Multiple nuclear magnetic resonance (NMR) experiments (^1^H NMR, correlation spectroscopy (COSY), heteronuclear single quantum coherence (HSQC) and selective one-dimensional (1D) total correlation spectroscopy (TOCSY); Supplementary Information section 4.4) permitted the assignment of all the protons in the molecules. The downfield shift of Rha-5 indicated that the presence of the unconventional H-bonding stabilizing the folded conformation of the turn motif^42,43^. Nuclear Overhauser effect spectroscopy (NOESY) analysis supported by molecular dynamics (MD) simulations (Fig. 2a,b and Supplementary Information section 4.3) confirmed the rigid conformation of the turn motif (Supplementary Fig. 16) and the correct orientation of the Gal arm (that is, with the α-face pointing towards the hydroxyl group of Rha C-4).

l-Tryptophan (Trp), frequently present in the carbohydrate-recognition site of proteins^35,45^, was chosen for the CH–π interaction analysis. **4mer-I** (3.5 mM) was incubated with Trp (10.5 mM) in water, and the mixture was analysed by ^1^H NMR and selective 1D TOCSY (Supplementary Information section 4.5.2)^35^. Chemical shift changes localized on the Gal unit (Fig. 2c) indicated CH–π interactions between **4mer-I** and Trp^35^. A minor contribution of unspecific interactions (including hydrophobic effects) could also be involved in such interactions^46^.

To further support the position of the interaction between **4mer-I** and Trp, we synthesized **5mer** (Supplementary Information section 3.5.7), in which the α-face of Gal was hindered by the presence of a Glc residue on the lower arm. Rotating-frame nuclear Overhauser effect spectroscopy (ROESY) experiments (Fig. 2d and Supplementary Fig. 43) confirmed the proximity between the α-face of Gal and the Glc unit. Comparative analysis confirmed that obstructing the α-face of Gal in **5mer** diminished CH–π interactions with Trp (Fig. 2d and Supplementary Information section 4.5.4). Similar results were obtained when the CH–π interactions analysis was performed with the neutral 5-OH-indole instead of Trp (Supplementary Information sections 4.5.3 and 4.5.5), ruling out the influence of potential ionic interactions.

Taken together, these observations proved that **4mer-I** folds into a rigid conformation and can recognize an aromatic substrate via CH–π interactions localized on the Gal unit. Thus, **4mer-I** was further functionalized to install a reactive group capable of promoting a chemical reaction on an aromatic substrate.

### Insertion of a reactive functional group

**Fig. 3:**
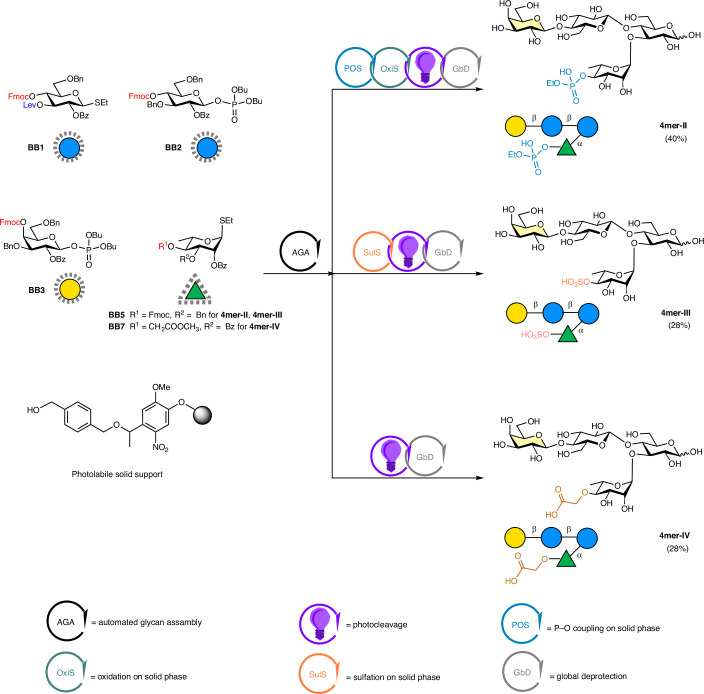
Synthesis of three glycan foldamers carrying an acid group. AGA and post-AGA steps to obtain three glycan structures starting from protected monosaccharide building blocks (represented with SNFG icons surrounded by grey dots). Overall yields are reported in parentheses. Reaction conditions for AGA and post-AGA are reported in the Supplementary Information. The colours of the chemical structures relate to the colours of the icons for the various functionalization processes: blue for phosphate modification, orange for sulfate modification and brown for carboxylic modification.

For the functionalization of our glycan foldamer, we focused on groups enabling the Brønsted-acid type of catalysis^47^, used in wide range of organic transformations such as C–C/C–X bond formation^48–50^. The functionalization was strategically planned at the equatorial hydroxyl group of Rha C-4 (Fig. 3), proximal to the Gal α-face. Inspired by binaphthol-derived acid catalysts^51,52^, we designed **4mer-II**, which incorporates a phosphoric acid group. The target glycan was prepared by AGA from **BB1-3** and **BB5** following iterative cycles of glycosylation and deprotection. The solid bound tetrasaccharide, carrying a free hydroxyl group at Rha C-4, was further subjected to P–O coupling followed by oxidation to afford the protected phosphorylated intermediate^53^. Cleavage from the solid support promoted by ultraviolet irradiation and global deprotection afforded the target **4mer-II** in a 40% overall yield (Supplementary Information section 3.5.3). A similar strategy was used to construct the sulfated tetrasaccharide **4mer-III**. AGA was followed by sulfation on solid phase^54^, photocleavage and global deprotection to give **4mer-III** in a 28% overall yield (Supplementary Information section 3.5.4). **4mer-IV** carrying a carboxylic acid group was synthesized using the carboxylic ester functionalized **BB7** in place of **BB5**. AGA, photocleavage and global deprotection resulted in a 28% overall yield (Supplementary Information section 3.5.5).

**4mer-II**, **4mer-III**, and **4mer-IV** were analysed by NOESY or ROESY (Supplementary Figs. 21, 26 and 32) to verify that the functional modifications did not compromise the conformational integrity of the glycan backbone. The observed spatial correlations for all modified 4mers were consistent with those of the original **4mer-I**, confirming that these functional groups did not alter the foldamer conformation (Supplementary Figs. 22, 27 and 33). An additional spatial correlation between Glc′ H-2 with Rha H-6 was observed for **4mer-II** and **4mer-III** (Supplementary Figs. 21 and 26) and to a lesser extent for **4mer-IV** (Supplementary Figs. 31 and 32), indicating a slightly more rigid conformation than **4mer-I** (Supplementary Fig. 16). This result was further supported by MD simulations of **4mer-I** versus **4mer-III** (Supplementary Figs. 5 and 6) and might be connected with the electron-withdrawing nature of the acid substituents, which strengthens the unconventional H-bonding between Rha H-5 and the endocyclic oxygen of Glc in the turn unit^55^.

**Fig. 4:**
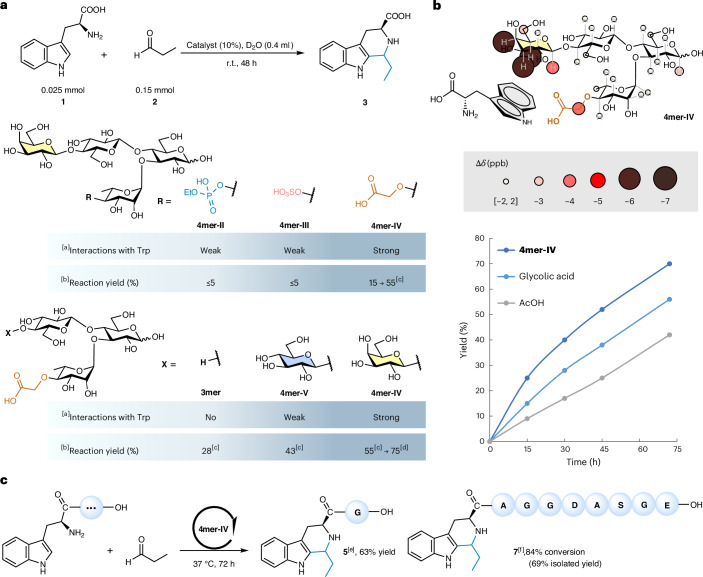
Application of a glycan foldamer as catalyst in a Pictet–Spengler reaction. **a**, Pictet–Spengler reaction between Trp **1** and propionaldehyde **2**. ^a^Interactions with Trp are defined from the sum of Δ*δ* (ppb) for all the protons pointing to the α-face (H-1, H-3, H-4, H-5 and H-6 for **4mer-II–IV** and H-1, H-3, H-5 and H-6 for **4mer-V**): weak <20 ppb, strong ≥20 ppb). ^b^The reaction yield was monitored by ^1^H NMR. ^c^Variations from standard condition: reaction time 168 h, **2** (0.3 mmol). ^d^Optimized condition: **4mer-IV** (20%), 72 h, 37 °C, **2** (0.3 mmol). **b**, Experimentally observed CH–π interactions for **4mer-IV** (3.5 mM) incubated with Trp (10.5 mM) and kinetic analysis of the Pictet–Spengler reaction catalysed by **4mer-IV** (20%), glycolic acid (20%) or AcOH (20%), 37 °C using ^1^H NMR. **c**, Functionalization of peptides via **4mer-IV** (20%) catalysed Pictet–Spengler reaction. ^e^The reaction was conducted in water and monitored by ^1^H NMR. ^f^The reaction was conducted in buffer and monitored by ^1^H NMR. r.t., room temperature. Source data

We then investigated the impact of the ionic groups on CH–π interactions, following the same experimental protocol used for **4mer-I** and Trp (Supplementary Information sections 4.5.6–4.5.8). The phosphorylated and sulfated variants, **4mer-II** and **4mer-III**, exhibited a reduction in CH–π interactions compared with the neutral **4mer-I**, which can be attributed to hinderance of the bulky acid groups and the increased rigidity of the glycan scaffolds. By contrast, **4mer-IV** displayed interaction levels akin to those of the neutral **4mer-I** (Fig. 4b). We reasoned that the extra methylene group in **4mer-IV** may offer enhanced flexibility to better accommodate the aromatic group in proximity to the Gal unit.

### Catalysing a Pictet–Spengler reaction with a glycan foldamer

Equipped with a set of glycan foldamers carrying a Brønsted-acid functional group, we set to test their catalytic performance in a Pictet–Spengler transformation^56^. The Pictet–Spengler reaction is a well-established method for the construction of asymmetric alkaloid frames, extensively explored in organic medium^57,58^ and, more recently, adapted to aqueous environments^59,60^. The latter aligns with the requirement of chemical biology, despite the challenge of typically slower reaction kinetics in such conditions.

We tested the Pictet–Spengler reaction between Trp **1** and propionaldehyde **2** in D_2_O (to allow NMR monitoring) at room temperature. The reaction showed negligible conversion in the absence of catalysis (Supplementary Information section 5.2) or in the presence of **4mer-II** or **4mer-III** as catalyst (Fig. 4a). By contrast, using **4mer-IV** as the catalyst led to the formation of the desired product, albeit with a modest yield of 15 %. Longer reaction times (168 h) and an increased amount of aldehyde (0.3 mmol) improved the yield to 55% (Fig. 4a). These observations suggested that the CH–π interactions between the catalyst **4mer-IV** and the Trp substrate were beneficial for the reaction.

To further validate the role of the CH–π interactions in promoting the desired transformation, we prepared a second series of catalysts containing the carboxylic acid functionality. **3mer** is an analogue of **4mer-IV** lacking the coordinating Gal unit and thus showing no interactions with Trp. **4mer-V** is equipped with a Glc arm in place of the Gal unit. CH–π interaction analysis for **4mer-V** revealed minor chemical shift changes localized on the Glc (Supplementary Information section 4.5.9). When the Pictet–Spengler transformation was performed in the presence of **3mer**, the reaction yielded the desired product (Fig. 4a), but the conversion rate was notably slower than in the presence of **4mer-IV**. This result stressed the key contribution of the CH–π interactions in positioning the Trp substrate near the catalytic site. Intermediate yields were obtained when the reaction was performed with **4mer-V** (Fig. 4a), confirming the correlation between CH–π interactions and reaction rate.

With **4mer-IV** identified as the most effective catalyst, further optimization of the reaction conditions enabled achieving an 84% conversion and a 75% yield (Fig. 4a and Supplementary Information section 5.2). Control experiments using acetic acid (AcOH), AcOH + Gal, glycolic acid and **3mer** as substitutes for **4mer-IV** (Supplementary Information section 5.2, entries 13, 14, 15 and 16) resulted in lower conversions and yields. Different kinetic processes were observed when **4mer-IV** or other acids (AcOH and glycolic acid) were used as the catalyst, with **4mer-IV** promoting the acceleration of the desired reaction pathway (Fig. 4b). Despite the multiple chiral centres in **4mer-IV**, similar *cis*:*trans* ratios were detected for the control experiments. This outcome aligns with existing literature, which attributes the diastereoselectivity of the reaction primarily to the chirality of the Trp substrate, rather than the catalyst^61^.

Finally, we tested the glycan-catalysed Pictet–Spengler reaction to modify the N-terminus of Trp-containing peptides. The reaction could be successfully performed in both water and buffer (Fig. 4c), preserving faster kinetics than the AcOH-catalysed controls (Supplementary Information section 5.5). These findings suggest the potential of rationally designed glycan catalysts in chemical biology, for example, for the selective functionalization of Trp in proteins^62,63^.

## Conclusions

In summary, we designed a glycan foldamer capable of catalysing a Pictet–Spengler transformation. Our design featured a conformationally stable tetrasaccharide inspired by Sialyl Lewis X. This modular glycan frame was rationally modified to include (1) a Gal unit to engage in carbohydrate–aromatic interactions with an aromatic substrate and (2) a reactive carboxylic acid group to perform a catalytic transformation. We demonstrated that our glycan could successfully recognize a Trp substrate via carbohydrate–aromatic interactions localized on the Gal unit. This interaction resulted in the acceleration of the Pictet–Spengler reaction between Trp and an aldehyde substrate in water.

These results demonstrated that glycans can be designed to perform catalytic functions. While this challenge was envisioned to push the boundaries of glycan synthesis and increase our understanding of carbohydrate interactions with other molecules, our results suggest that glycan foldamers could have important applications in chemical biology, offering water-soluble and modular catalysts for organic transformations. Our scaffold is modular and could be easily adapted to accommodate other functional groups (for example, amino groups) to open up new catalytic pathways. To this end, improvements in glycan synthesis^64,65^ and mechanistic studies^46^ are key to inspire the design of new glycan catalysts and obtain greater quantities of materials.

Lastly, these findings raise the question of whether glycans could have active catalytic roles also in natural settings, like proteins and some RNAs^66^. For example, it was recently reported that cation–π interactions could modulate the reactivity of selected Trp in proteins^62^. We speculate that carbohydrate–aromatic interactions could have similar effects and (dis)favour selective protein modifications. Similarly, natural glycans carry numerous ionic groups that might engage in interactions with a substrate and tune its reactivity, opening up new perspectives in the glycosciences.

## Methods

### Synthesis

The oligosaccharides were prepared using a home-built synthesizer designed at the Max Planck Institute of Colloids and Interface^68^. All details concerning **BB** synthesis, AGA modules and post-AGA manipulations can be found in Supplementary Information sections 2 and 3.

### MD simulations

For all simulations, the modified version GLYCAM06 force field was used^69^. Initial conformations for single-hairpin simulations were constructed with the Glycam Carbohydrate builder and tleap (https://glycam.org/). Both compounds with a free reducing end were modelled as β anomers. The topology was subsequently converted using the Python script acpype. Both simulations were performed in water as solvent using TIP5P as the water model^70^. The simulation time for the single-molecule experiments was 500 ns. Bonds involving hydrogens were constrained using the LINear Constraint Solver (LINCS) to allow a 2 fs timestep. Non-bonded interactions were cut off at 1.4 nm, and long-range electrostatics were calculated using the particle mesh Ewald method^71^. After energy minimization (steepest descent algorithm) and before the production run, the systems were equilibrated at 300 K for 50 ns in a canonical (NVT) ensemble (constant number of particles, volume and temperature) and subsequently at 300 K and 1 bar for 50 ns in an isothermal–isobaric (NPT) ensemble. All MD simulations were performed using Gromacs 5.1.2 (ref. ^72^). A Nosé–Hoover thermostat^73^ kept the constant temperature of 303 K constant while a Parrinello–Rahman barostat^74^ ensured a constant pressure of 1 bar. The analysis was visualized using OriginPro 2021b. Further details on MD simulations are reported in Supplementary Information section 4.3.

### NMR analysis

^1^H, ^13^C, HSQC, 1D and two-dimensional (2D) TOCSY, 2D ROESY and 2D NOESY NMR spectra were recorded on a Varian 400-MR (400 MHz), Varian 600-NMR (600 MHz) and Bruker Biospin AVANCE700 (700 MHz) spectrometer. Samples were prepared by dissolving lyophilized samples in D_2_O. Proton resonances of the oligosaccharides were assigned using a combination of ^1^H, 2D COSY, HSQC, and 1D and 2D TOCSY. Selective 1D TOCSY (HOHAHA, pulse program: seldigpzs) spectra were recorded using different mixing times to assign all the resonances (mixing time d9 is 40, 80, 120, 160, 200, 350 and 450 ms). Two-dimensional TOCSY (pulse program: mlevphpp) spectra were recorded using a mixing time of d9 at 80 ms. Two-dimensional ROESY (pulse program: reosyph.2) and 2D NOESY (pulse program: noesygpphpp) spectra were recorded using different mixing times (mixing time p15 is 200 ms for ROESY and mixing time d8 is 1,000 ms for NOESY). d8 determines the duration of mixing time. It largely depends on the relaxation behavior of the investigated molecule. d9 determines the duration of the spin lock and hence over how many protons the magnetization will be distributed. p15 is the duration of the spin lock and is equal to the mixing time. The full NMR analysis of glycans and CH–π interactions reported in this article can be found in Supplementary Information sections 4.4 and 4.5.

### Reporting summary

Further information on research design is available in the Nature Portfolio Reporting Summary linked to this article.

## Online content

Any methods, additional references, Nature Portfolio reporting summaries, source data, extended data, supplementary information, acknowledgements, peer review information; details of author contributions and competing interests; and statements of data and code availability are available at 10.1038/s41557-025-01763-6.

## Supplementary information

Supplementary Information Supplementary Figs. 1–77.

Reporting Summary

## Source data

Source Data Fig. 4 Statistical source data.

## Data Availability

The authors declare that all data supporting the findings of this study are available within the Article and its Supplementary Information files. Raw data for NMR analysis and MD simulations are available at 10.17617/3.QMXNSH. Data are also available from the corresponding author upon request (for example, if a different format is needed). Source data are provided with this paper.
