# Supplementary material for: A glycan foldamer that uses carbohydrate–aromatic interactions to perform catalysis
Source: Nat Chem. 2025 Feb 26;17(6):883–9. doi: 10.1038/s41557-025-01763-6 (PMC12141037; doi:10.1038/s41557-025-01763-6)
Supplement: Supplementary file 1 — Supplementary Figs. 1–77. [file 41557_2025_1763_MOESM1_ESM.pdf]

nature chemistry

Supplementary information https://doi.org/10.1038/s41557-025-01763-6

A glycan foldamer that uses carbohydrate–aromatic interactions to perform catalysis

In the format provided by the authors and unedited

“In nature, the ability to catalyze reactions is a prerogative of proteins and ribozymes. Now it has been shown that glycans can be designed to perform catalysis. Exploiting carbohydrate-aromatic interactions, a glycan foldamer enables a Pictet-Spengler transformation of tryptophan and tryptophan-containing peptides in water.”

Contents

1 General materials and methods 5
2 Building blocks 6
2.1 Synthesis of BB2 7
2.2 Synthesis of BB3 8
2.3 Synthesis of BB5 12
2.4 Synthesis of BB7 21
3 Automated glycan assembly (AGA) 28
3.1 General materials and method 28
3.2 Preparation of stock solutions 28
3.3 Modules for automated synthesis 28
3.3.1 Module A: Resin preparation for synthesis (20 min) 28
3.3.2 Module B: Acidic wash with TMSOTf solution (20 min) 29
3.3.3 Module C1: Thioglycoside glycosylation (2 h – 3 h) 29
3.3.4 Module C2: Glycosyl phosphate glycosylation (2.5 h) 30
3.3.5 Module D: Capping (30 min) 30
3.3.6 Module E1: Fmoc deprotection (18 min) 31
3.3.7 Module E2: Lev deprotection (95 min) 31
3.4 Post-synthesizer manipulations (Post-AGA) 32
3.4.1 Module F: The coupling of P-O bond on resin 32
3.4.2 Module G: Oxidation on resin 32
3.4.3 Module H: Sulfation on resin 32
3.4.4 Module I1: Cleavage from solid support 32
3.4.5 Module I2: Micro-cleavage from solid support 33
3.4.6 Module J1: Solution-phase methanolysis 33
3.4.7 Module J2: Solution-phase hydrolysis 33
3.4.8 Module K1: Hydrogenolysis 33
3.4.9 Module K2: Hydrogenolysis at ambient pressure 33
3.4.10 Module L: Purification 33
3.5 Oligosaccharides synthesis 35
3.5.1 3mer 36
3.5.2 4mer-I 40
3.5.3 4mer-II 44
3.5.4 4mer-III 49
3.5.5 4mer-IV 53

3.5.6 4mer-V 57
3.5.7 5mer 61
4 Structural analysis 66
4.1 General materials and methods for molecular dynamics simulations 66
4.2 General materials and methods for NMR 66
4.3 Molecular dynamics simulations 67
4.4 NMR analysis 72
4.4.1 NMR analysis of 3mer 72
4.4.2 NMR analysis of 4mer-I 77
4.4.3 NMR analysis of 4mer-II 82
4.4.4 NMR analysis of 4mer-III 87
4.4.5 NMR analysis of 4mer-IV 92
4.4.6 NMR analysis of 4mer-V 98
4.4.7 NMR analysis of 5mer 103
4.5 CH/π interactions analysis by NMR 108
4.5.1 General method 108
4.5.2 CH/π interactions analysis: 4mer-I with L-tryptophan 108
4.5.3 CH/π interactions analysis: 4mer-I with 5OH-indole 112
4.5.4 CH/π interactions analysis: 5mer with L-tryptophan 115
4.5.5 CH/π interactions analysis: 5mer with 5OH-indole 119
4.5.6 CH/π interactions analysis: 4mer-II with L-tryptophan 123
4.5.7 CH/π interactions analysis: 4mer-III with L-tryptophan 126
4.5.8 CH/π interactions analysis: 4mer-IV with L-tryptophan 130
4.5.9 CH/π interactions analysis: 4mer-V with L-tryptophan 134
5 Pictet-Spengler reaction 138
5.1 Pictet-Spengler modification of L- tryptophan 138
5.2 Reaction conditions optimization 140
5.3 NMR of crude reaction system 141
5.4 Kinetic investigation 157
5.4.1 General method 157
5.4.2 NMR of crude reaction 159
5.5 Pictet-Spengler modification of peptides 172
5.5.1 Functionalization of H-Trp-Gly-OH in the presence of 4mer-IV 172
5.5.2 Functionalization of H-Trp-Gly-OH in the presence of AcOH 178

5.5.3 Functionalization of H-Trp-Ala-Gly-Gly-Asp-Ala-Ser-Gly-Glu-OH in the presence of 4mer-IV 180

5.5.4 Functionalization of H-Trp-Ala-Gly-Gly-Asp-Ala-Ser-Gly-Glu-OH in the presence of AcOH 186

5.6 Limitations of the method 187

5.6.1 Functionalization of tryptamine 187

5.6.2 Functionalization of an internal Trp 189

6 References 191

1 General materials and methods

All chemicals used were reagent grade and used as supplied unless otherwise noted. The automated syntheses were performed on a home-built synthesizer developed at the Max Planck Institute of Colloids and Interfaces.[1] Analytical thin-layer chromatography (TLC) was performed on Merck silica gel 60 F254 plates (0.25 mm). Compounds were visualized by UV irradiation or dipping the plate in a staining solution (sugar stain: 10% H_2SO_4 in EtOH; CAM: 48 g/L ammonium molybdate, 60 g/L ceric ammonium molybdate in 6% H_2SO_4 aqueous solution). Flash column chromatography was carried out by using forced flow of the indicated solvent on Fluka Kieselgel 60 M (0.04 – 0.063 mm). Analysis and purification by reverse phase HPLC were performed by using an Agilent 1200 series. Products were lyophilized using a Christ Alpha 2-4 LD plus freeze dryer. ^{1}H, ^{13}C, COSY and HSQC NMR spectra were recorded on a Varian 400-MR (400 MHz), Varian 600-MR (600 MHz), or Bruker Biospin AVANCE700 (700 MHz) spectrometer. Spectra were recorded in $CDCl_3$ by using the solvent residual peak chemical shift as the internal standard ($CDCl_3$: 7.26 ppm ^{1}H, 77.16 ppm ^{13}C) or in D_2O using the solvent as the internal standard in ^{1}H NMR (D_2O: 4.79 ppm ^{1}H). High resolution mass spectra were obtained using a 6210 ESI-TOF mass spectrometer (Agilent) and a MALDI-TOF autoflex^{TM} (Bruker).

2 Building blocks

Building blocks (BBs) **BB1**, **BB2** and **BB3** were synthesized according to previous literature procedures.[2, 3] **BB4**, and **BB6** were purchased from GlycoUniverse (Germany). Merrifield resin equipped with a photocleavable linker (**L**, loading 0.34 mmol/g) was prepared according to previous literature.[4]

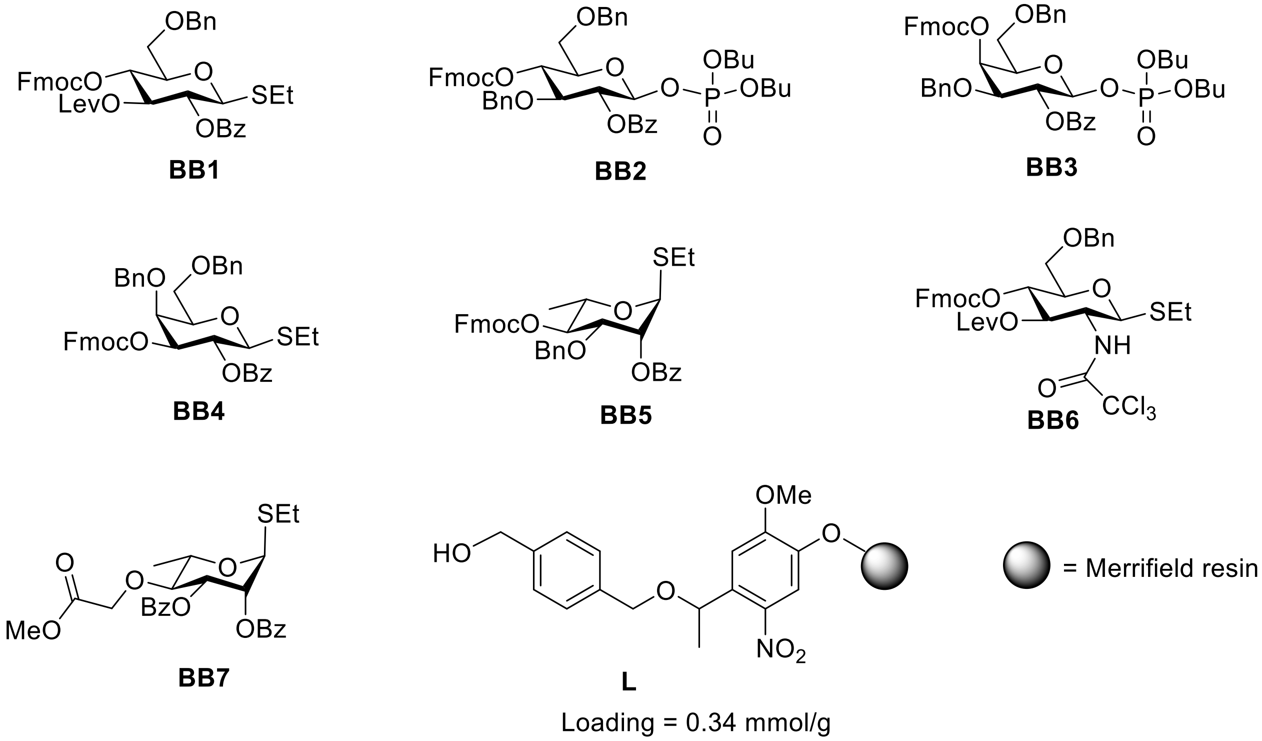

Figure S1 BBs and solid support used in this work.

2.1 Synthesis of BB2

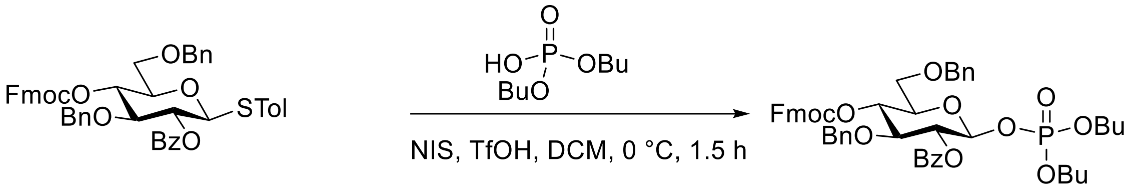

S-a (purchased from GlycoUniverse) (2.0 g, 2.5 mmol) was co-evaporated three times with DCM and toluene, dissolved in anhydrous DCM (12 mL), and stirred in the presence of 4 Å molecular sieves (1.6 g, flamed dried) under N_2 atmosphere. A solution of di-*n*-butyl phosphate (0.75 mL, 3.8 mmol) in anhydrous DCM (6 mL) was added to above reaction mixture cooled to 0 °C, followed by slow addition of *N*-iodosuccinimide (0.67 g, 3.0 mmol) and TfOH (15 µL, 0.15 mmol). The reaction was stirred at 0 °C for 2 h, after which time it was quenched with pyridine (20 µL, 0.25 mmol). The mixture was filtered over celite, and washed with a saturated aqueous solution of $Na_2S_2O_3$ and brine. The organic layer was dried with Na_2SO_4 and concentrated under reduced pressure. The crude product was purified by silica gel flash column chromatography (Hexane : EA = from 1 : 0.4 to 1 : 1) to give **BB2** (1.8 g, 80%) as a colorless sticky liquid. NMR data were consistent with the reported literature.[3]

^{1}H NMR (400 MHz, $CDCl_3$) δ 7.97 – 7.90 (m, 2H), 7.71 – 7.63 (m, 2H), 7.55 – 7.43 (m, 3H), 7.39 – 7.28 (m, 4H), 7.26 – 7.13 (m, 8H), 7.05 – 6.93 (m, 5H), 5.36 – 5.28 (m, 2H), 5.02 (t, *J* = 9.6 Hz, 1H), 4.52 (d, *J* = 11.6 Hz, 1H), 4.48 – 4.40 (m, 3H), 4.32 – 4.21 (m, 2H), 4.08 – 4.01 (m, 2H), 3.99 – 3.89 (m, 2H), 3.85 – 3.73 (m, 2H), 3.70 – 3.53 (m, 4H), 1.54 – 1.43 (m, 2H), 1.21 (m, 6H), 0.98 – 0.87 (m, 2H), 0.79 (t, *J* = 7.4 Hz, 3H), 0.59 (t, *J* = 7.3 Hz, 3H).

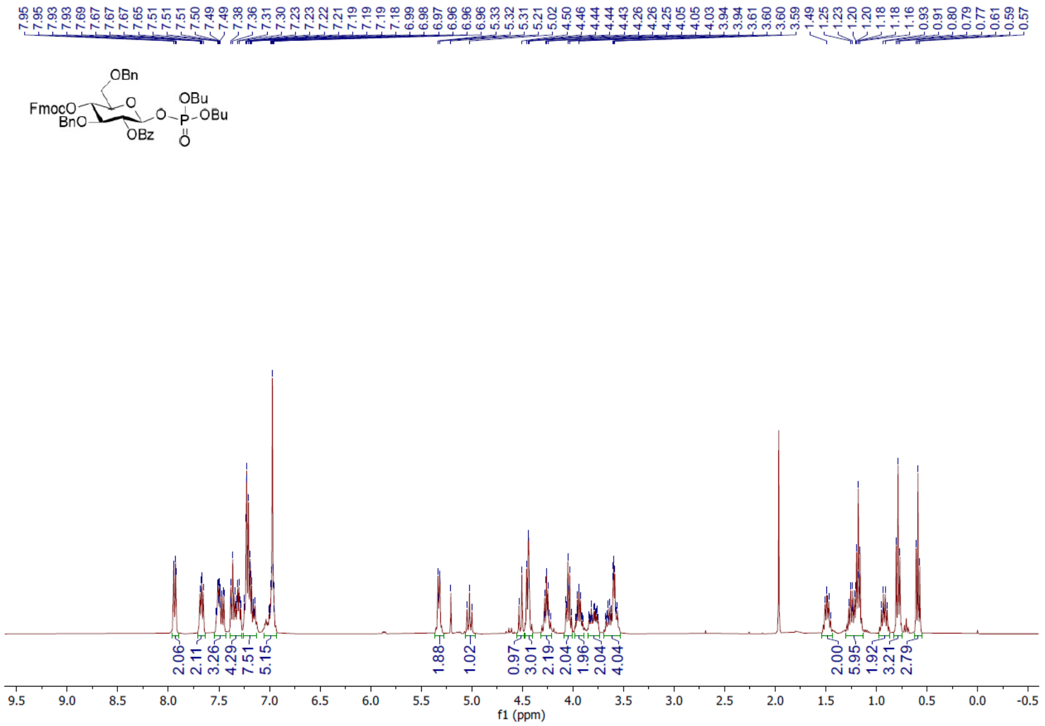

^{1}H NMR of **BB2** (400 MHz, $CDCl_3$)

2.2 Synthesis of BB3

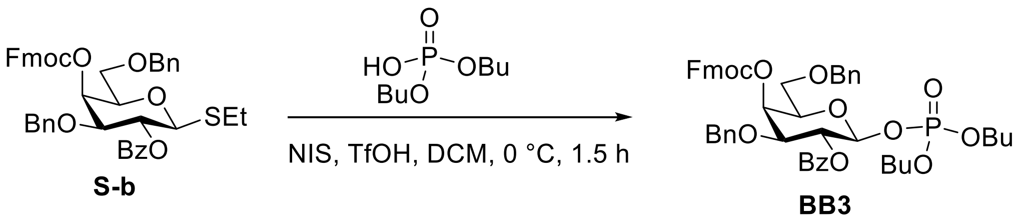

S-b (purchased from GlycoUniverse) (2.03 g, 2.76 mmol) was co-evaporated three times with DCM and toluene, dissolved in anhydrous DCM (12 mL), and stirred in the presence of 4 Å molecular sieves (1.6 g, flamed dried) under N_2 atmosphere. A solution of di-*n*-butyl phosphate (2.25 mL, 11.3 mmol) in anhydrous DCM (6 mL) was added to the above reaction mixture cooled to 0 °C, followed by slow addition of *N*-iodosuccinimide (0.75 g, 3.33 mmol) and TfOH (18 µL, 0.18 mmol). The reaction was stirred at 0 °C for 1.5 h, after which time it was quenched with pyridine (20 µL, 0.25 mmol). The mixture was filtered over celite and washed with a saturated aqueous solution of $Na_2S_2O_3$ and brine. The organic layer was dried with Na_2SO_4 and concentrated under reduced pressure. The crude product was purified by silica gel flash column chromatography (Hexane : EA = from 1 : 0.4 to 1 : 1) to give **BB3** (1.72 g, 71%) as a colorless sticky liquid.

^{1}H NMR (400 MHz, $CDCl_3$) δ 8.08 – 8.02 (m, 2H, Ar), 7.83 – 7.77 (m, 2H, Ar), 7.74 (dd, *J* = 7.5, 1.1 Hz, 1H, Ar), 7.67 (dd, *J* = 7.5, 1.0 Hz, 1H, Ar), 7.63 (t, *J* = 7.4 Hz, 1H, Ar), 7.51 – 7.39 (m, 5H, Ar), 7.36 – 7.26 (m, 6H, Ar), 7.17 – 7.09 (m, 3H, Ar), 7.04 (dd, *J* = 8.0, 6.8 Hz, 2H, Ar), 5.68 (dd, *J* = 10.0, 8.1 Hz, 1H, H-2), 5.56 (d, *J* = 3.1 Hz, 1H, H-4), 5.38 (t, *J* = 7.8 Hz, 1H, H-1), 4.72 (d, *J* = 12.7 Hz, 1H, CH_2-Bn), 4.60 – 4.45 (m, 4H, CH_2-Fmoc, CH_2-Bn), 4.32 – 4.25 (m, 2H, CH_2-Fmoc, CH-Fmoc), 4.11 – 4.01 (m, 2H, CH_2-Bu), 3.99 (m, 1H, H-5), 3.73 (m, 5H, H-6, H-3, CH_2-Bu), 1.66 – 1.58 (m, 2H, CH_2-Bu), 1.43 – 1.33 (m, 2H, CH_2-Bu), 1.31 – 1.27 (m, 2H, CH_2-Bu), 1.02 (m, 2H, CH_2-Bu), 0.92 (t, *J* = 7.4 Hz, 3H, CH_3-Bu), 0.69 (t, *J* = 7.4 Hz, 3H, CH_3-Bu). ^{13}C NMR (101 MHz, $CDCl_3$) δ 165.18, 155.07, 143.74, 143.22, 141.42, 141.31, 137.48, 137.01, 133.43, 130.12, 129.60, 128.62, 128.52, 128.37, 128.13, 128.09, 128.04, 127.99, 127.94, 127.92, 127.43, 125.84, 125.40, 120.09, 120.07, 97.01 (C-1), 76.00, 73.90, 72.86, 71.24, 70.78, 70.69, 70.44, 69.98, 68.16, 68.10, 68.02, 67.96, 67.33, 46.63, 32.15, 32.08, 31.89, 31.82, 18.69, 18.33, 13.70, 13.50. ^{31}P NMR (162 MHz, $CDCl_3$) δ -2.69. ESI-HRMS m/z 901.3322 $[M+Na]^+$ ($C_{50}H_{55}NaO_{12}P$ requires 901.3329).

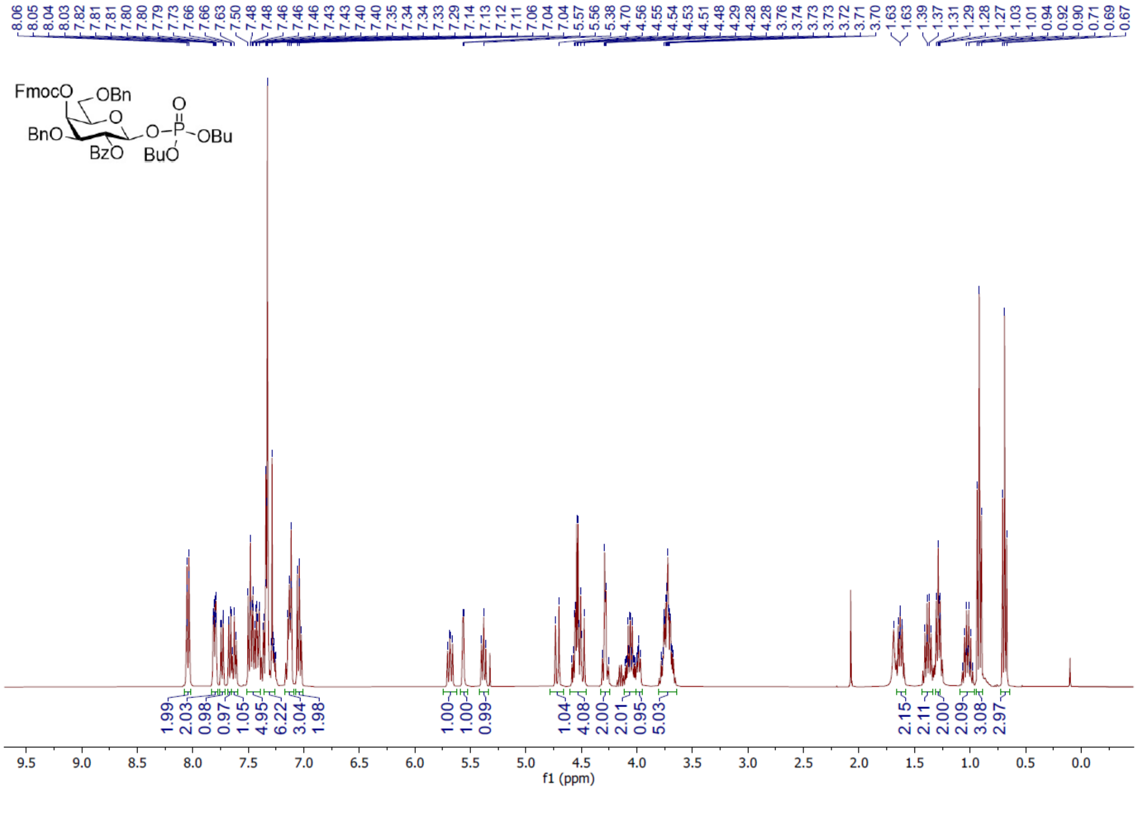

^{1}H NMR of **BB3** (400 MHz, $CDCl_3$)

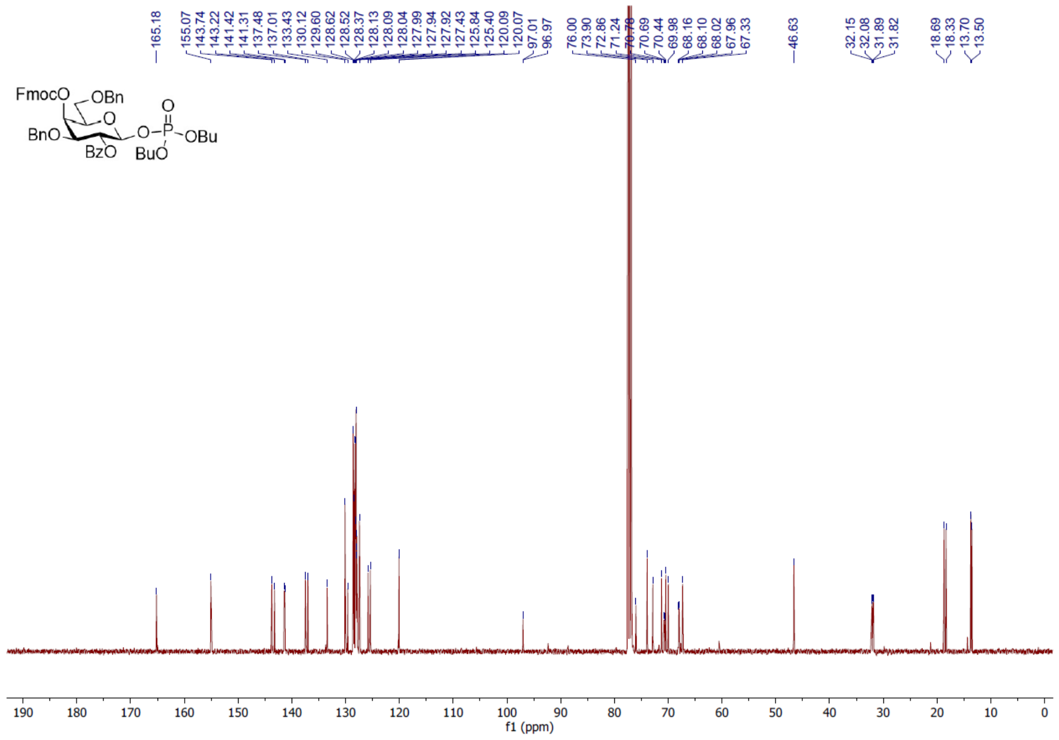

^{13}C NMR of **BB3** (101 MHz, $CDCl_3$)

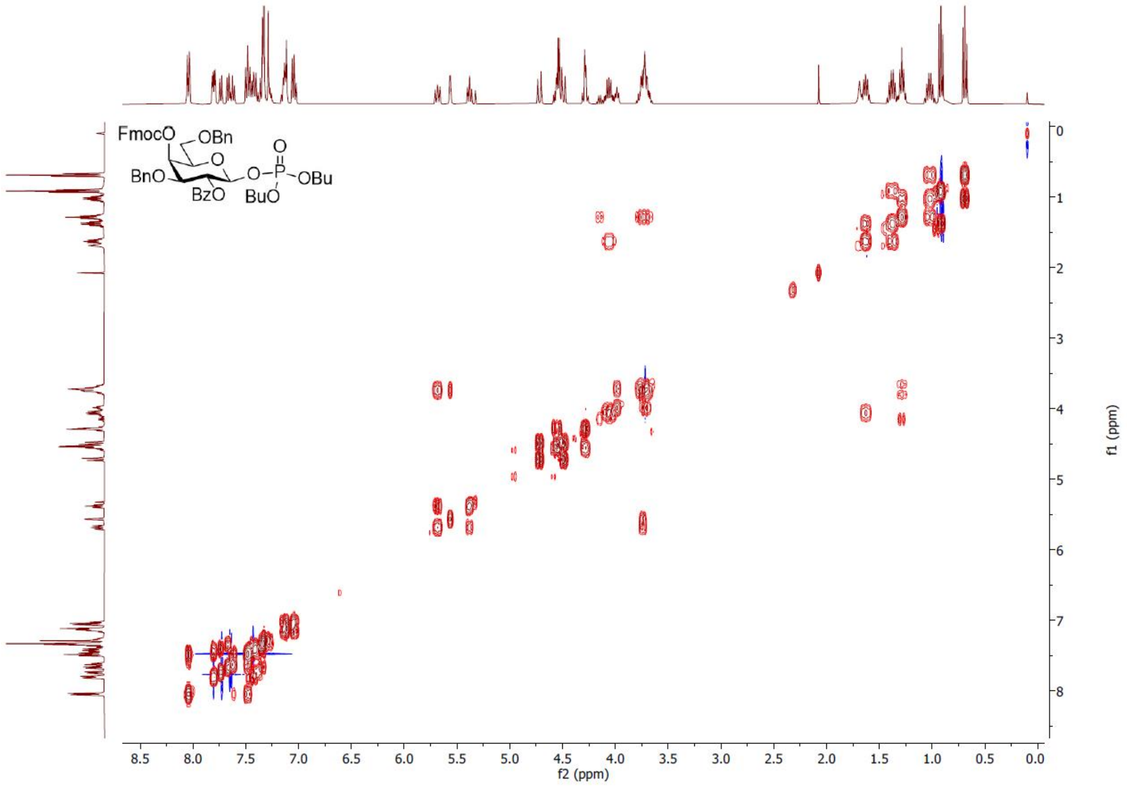

COSY NMR of **BB3** ($CDCl_3$)

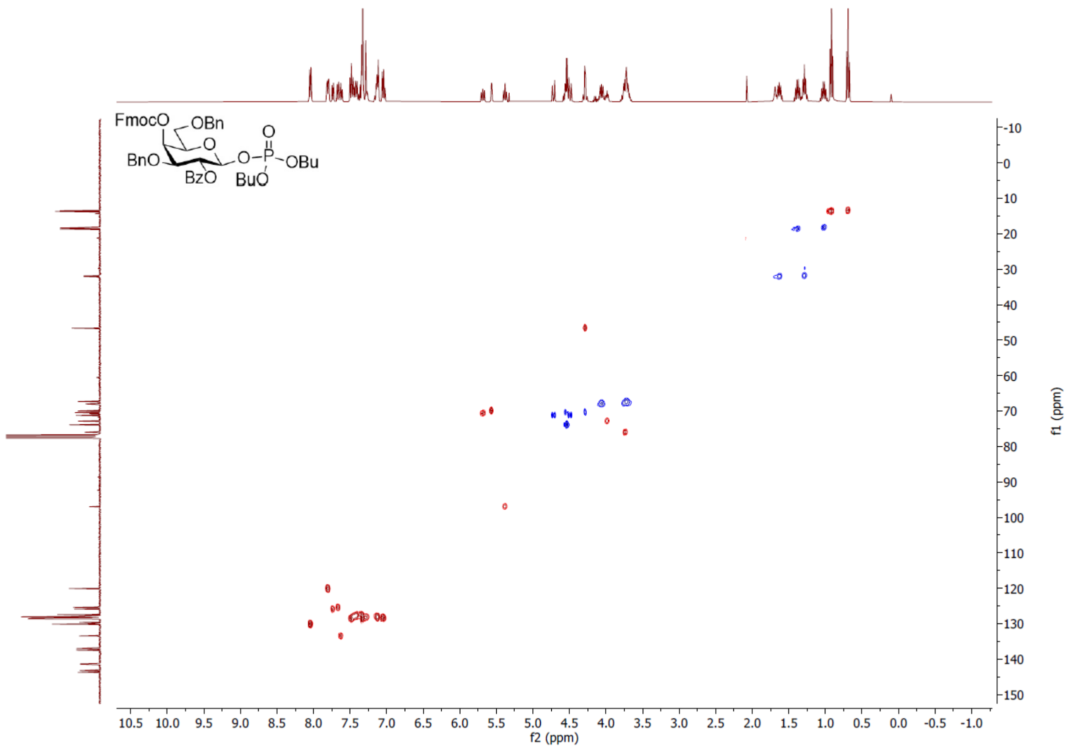

HSQC NMR of **BB3** ($CDCl_3$)

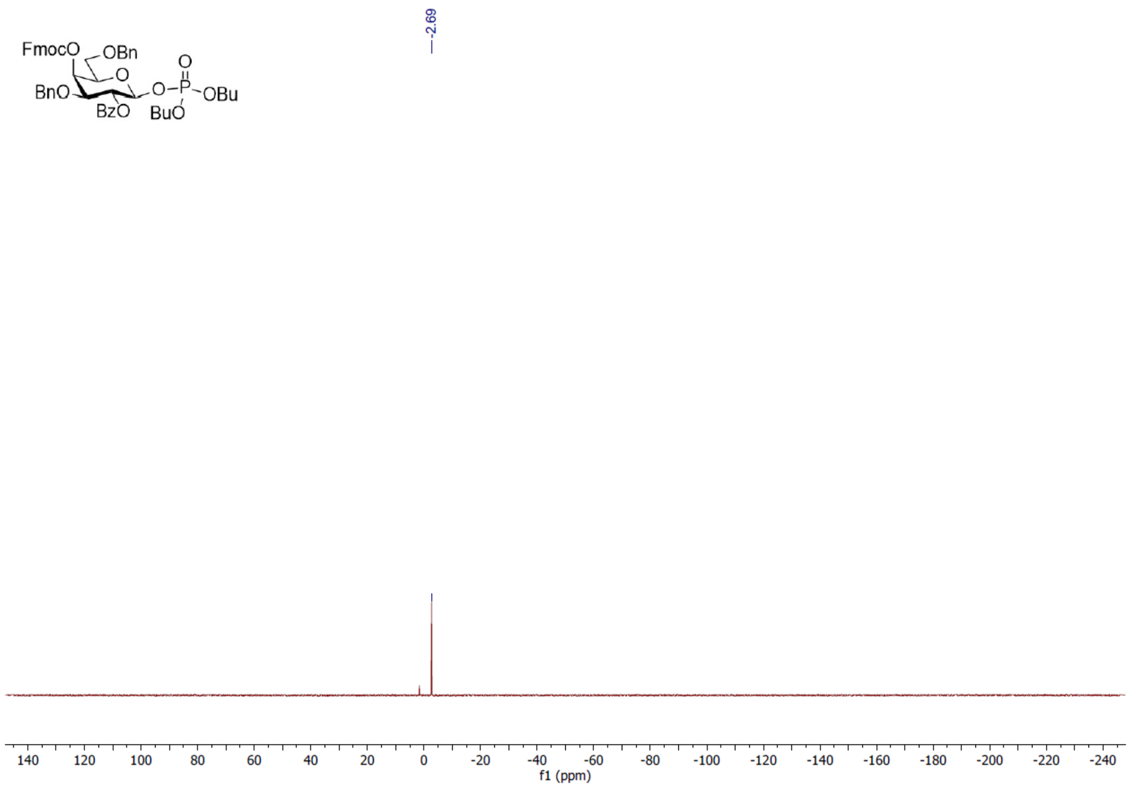

^{31}P NMR of **BB3** (162 MHz, $CDCl_3$)

2.3 Synthesis of BB5

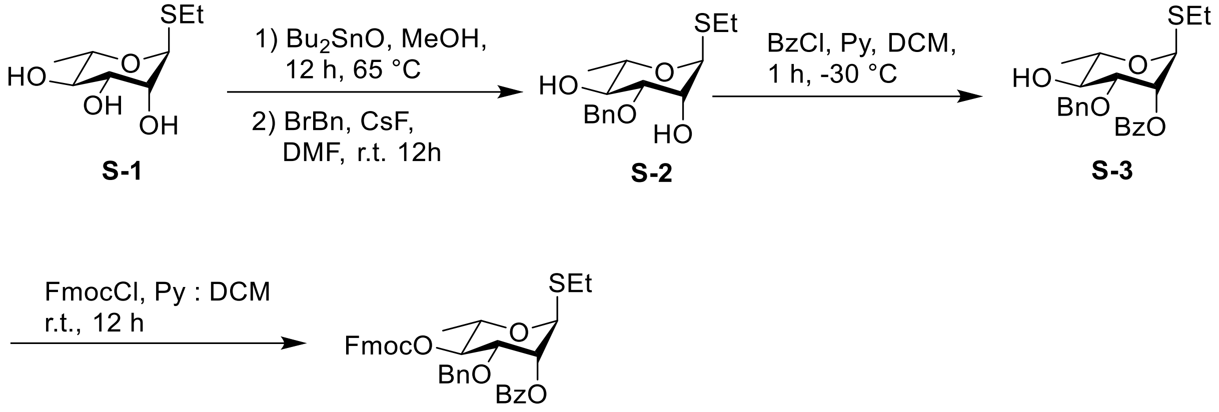

Figure S2 Synthesis of **BB5**.

Synthesis of S-2

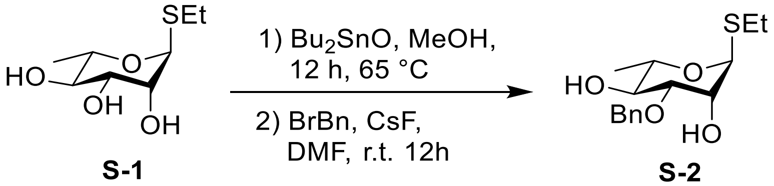

To a solution of **S-1** (10.0 g, 0.05 mol) in MeOH (200 mL) di-*n*-butyltin oxide (14.9 g, 0.06 mol) was added. The mixture (white suspension) was heated at reflux at 65 °C while stirring for 12 h. The formation of a clear solution indicated completion of the reaction. The mixture was cooled to room temperature and concentrated under reduced pressure. The resulting product was used for the next step without further purification. The crude product was dissolved in DMF (100 mL). Benzyl bromide (8.9 mL, 0.075 mol) and cesium(I) fluoride (12.2 g, 0.08 mol) were added while stirring at room temperature. The mixture was stirred for 12 h under Ar atmosphere. The mixture was filtered and the solution was concentrated under reduced pressure. The crude product was dissolved in EA and washed with brine twice. The organic layer was dried with Na_2SO_4 and concentrated. The crude product was purified by silica gel flash column chromatography (Hexane : Acetone = from 8 : 1 to 4 : 1) to give **S-2** (11.0 g, 74%) as a colorless sticky liquid.

^{1}H NMR (400 MHz, $CDCl_3$) δ 7.38 (m, 5H, Ar), 5.32 (d, *J* = 1.5 Hz, 1H, H-1), 4.71 (d, *J* = 11.5 Hz, 1H, CH_2-Bn), 4.58 (d, *J* = 11.5 Hz, 1H, CH_2-Bn), 4.11 (m, 1H, H-2), 4.09 – 4.01 (m, 1H, H-5), 3.65 – 3.56 (m, 2H, H-3,4), 2.72 – 2.53 (m, 2H, CH_2-SEt), 2.45 (s, 2H, OH), 1.35 – 1.28 (m, 6H, CH_3-

SEt, H-6). ^{13}C NMR (101 MHz, $CDCl_3$) δ 137.49, 128.87, 128.44, 128.18, 83.48 (C-1), 80.13, 72.00, 71.84, 69.47, 68.28, 25.13, 17.69, 15.01. ESI-HRMS m/z 321.1148 $[M+Na]^+$ ($C_{15}H_{22}NaO_4S$ requires 321.1136).

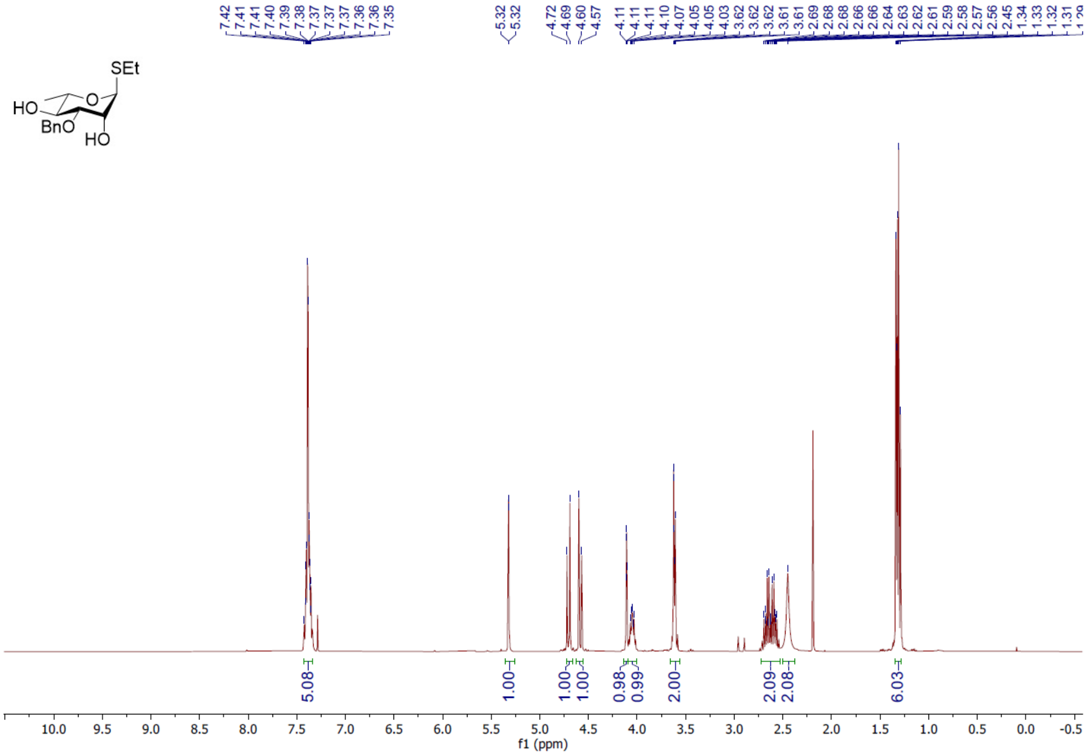

^1H NMR of **S-2** (400 MHz, $CDCl_3$)

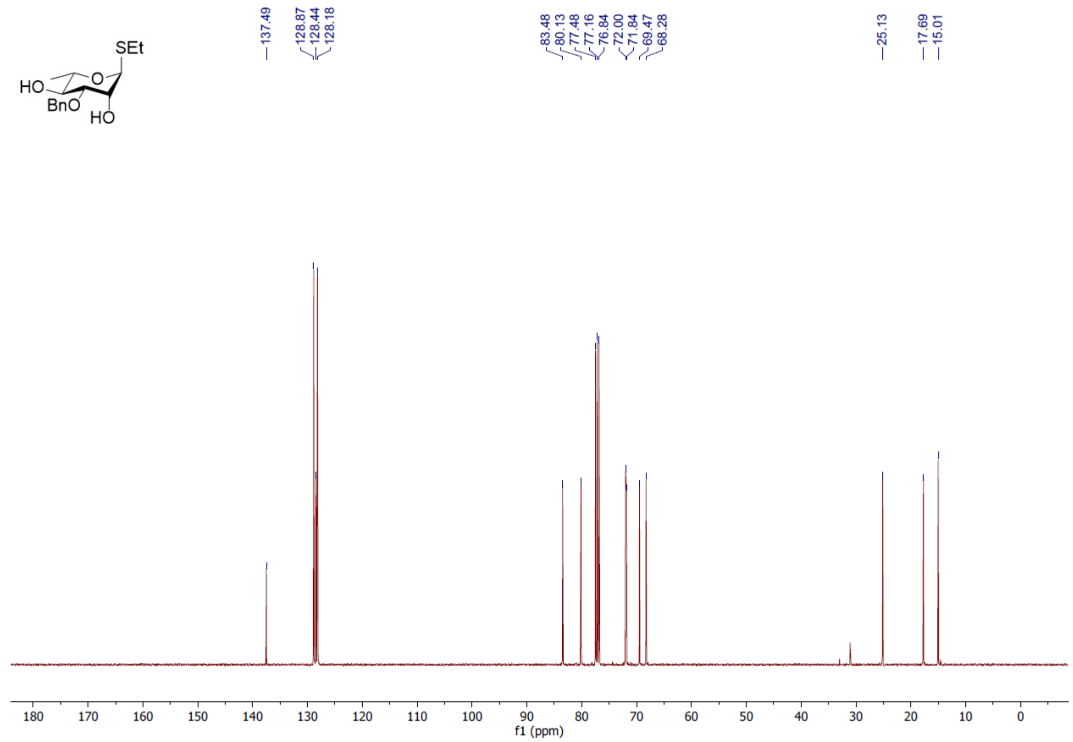

^{13}C NMR of **S-2** (101 MHz, $CDCl_3$)

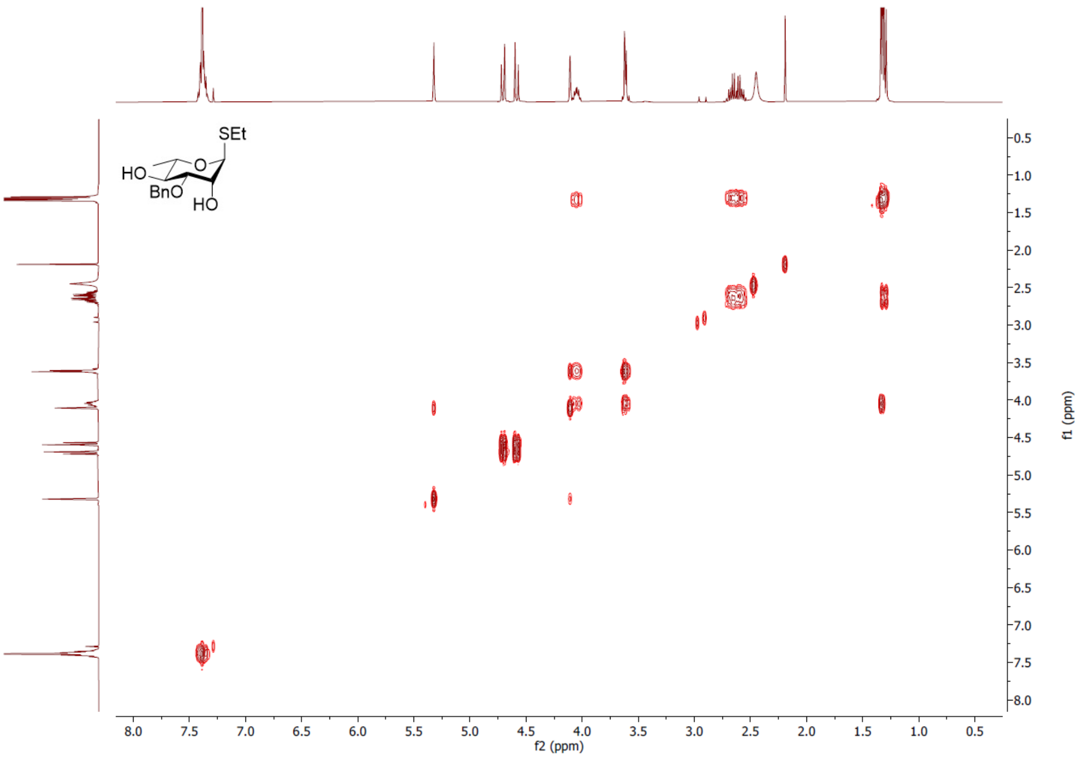

COSY NMR of **S-2** ($CDCl_3$)

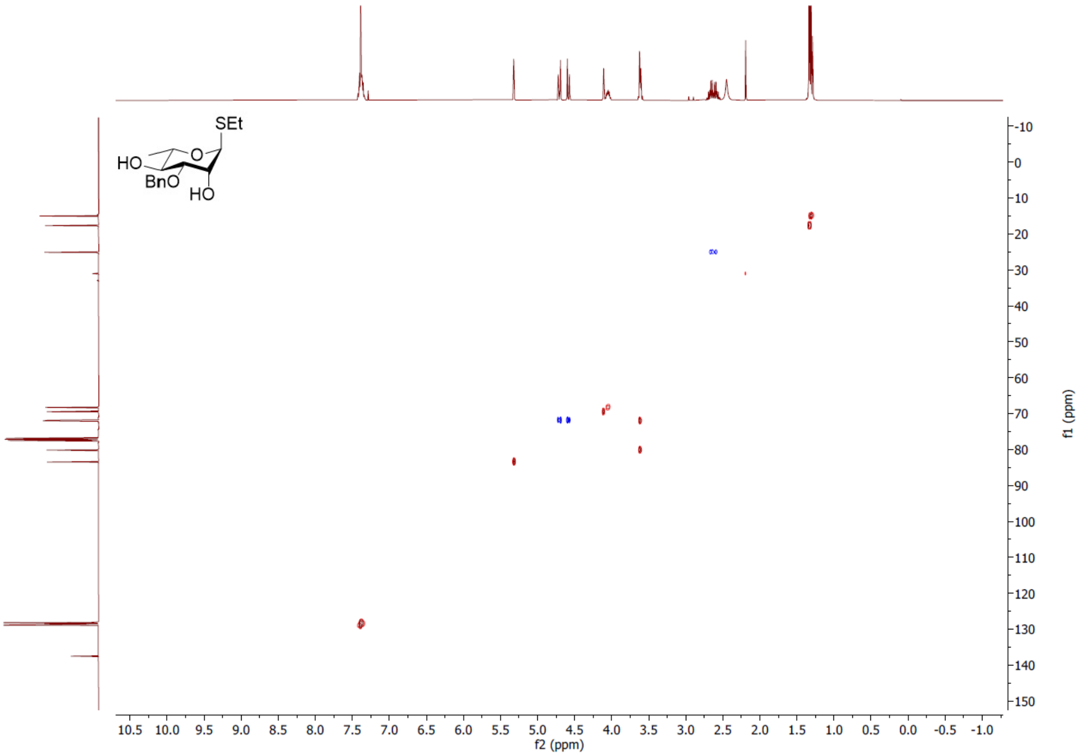

HSQC NMR of **S-2** ($CDCl_3$)

Synthesis of S-3

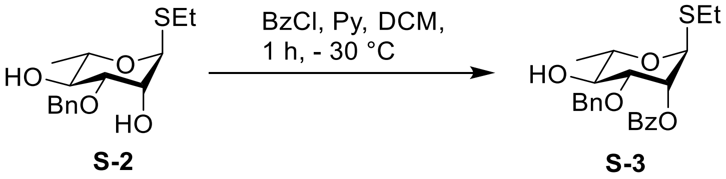

To a solution of **S-2** (11.0 g, 0.037 mol) in a mixture of pyridine and DCM (30 mL : 30 mL) at -30 °C a solution of benzoylchlorid (5.1 mL, 0.044 mol) in DCM (30 mL) was added dropwise. The reaction was kept at -30 °C for additional 1 h until TLC indicated completion of the reaction. The reaction was quenched with MeOH and washed twice with brine. The organic layers were combined, dried by Na_2SO_4 and concentrated under reduced pressure. The resulting crude product was purified by silica gel flash column chromatography (Hexane : EtOAc = from 7 : 1 to 4 : 1) to give **S-3** (11.3 g, 76%) as a colorless sticky liquid.

^{1}H NMR (400 MHz, $CDCl_3$) δ 8.06 – 7.95 (m, 2H, Ar), 7.55 – 7.47 (m, 1H, Ar), 7.44 – 7.34 (m, 2H, Ar), 7.20 (d, *J* = 1.8 Hz, 5H, Ar), 5.60 (dd, *J* = 2.8, 1.6 Hz, 1H, H-2), 5.29 (d, *J* = 1.5 Hz, 1H, H-1),

4.68 (d, J = 11.2 Hz, 1H, CH_2-Bn), 4.37 (d, J = 11.2 Hz, 1H, CH_2-Bn), 4.08 – 4.00 (m, 1H, H-5), 3.74 – 3.61 (m, 2H, H-3,4), 2.59 (m, 2H, CH_2-SEt), 2.28 (br, 1H, OH), 1.31 (d, J = 6.2 Hz, 3H, H-6), 1.24 (t, J = 7.4 Hz, 3H, CH_3-SEt). ^{13}C NMR (101 MHz, $CDCl_3$) δ 165.85, 137.44, 133.44, 130.01, 129.87, 128.69, 128.58, 128.36, 128.21, 82.62 (C-1), 78.21, 72.34, 71.47, 70.50, 68.78, 25.82, 17.94, 15.12. ESI-HRMS m/z 425.1388 [M+Na]$^+$ ($C_{22}H_{26}NaO_5S$ requires 425.1399).

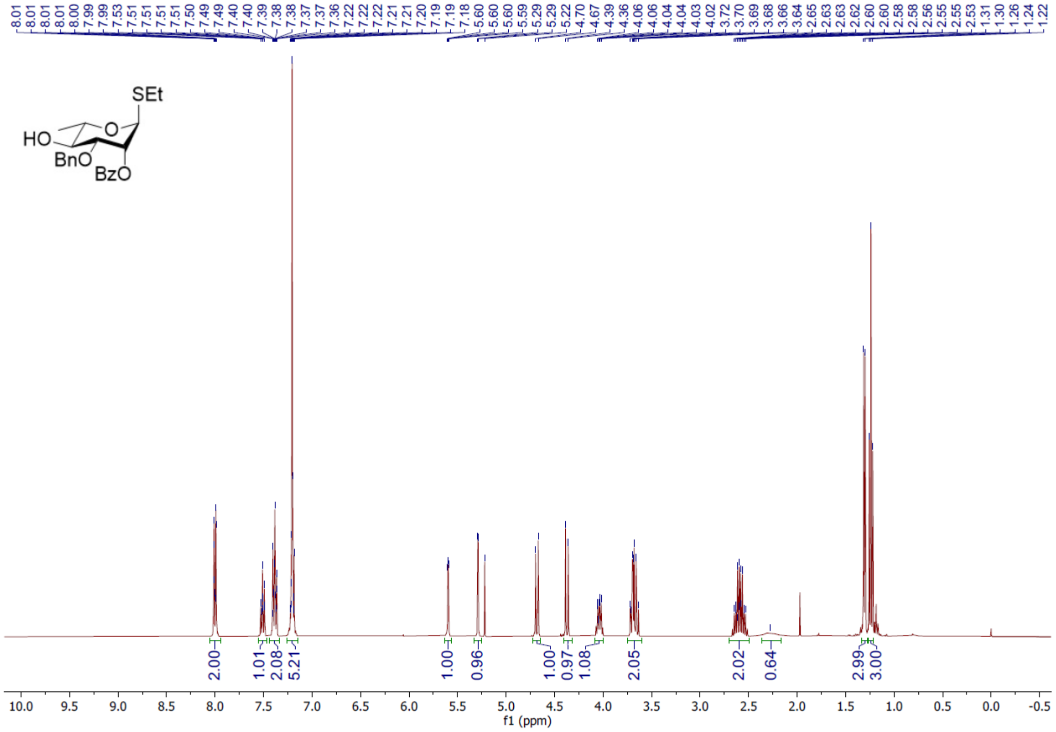

^{1}H NMR of **S-3** (400 MHz, $CDCl_3$)

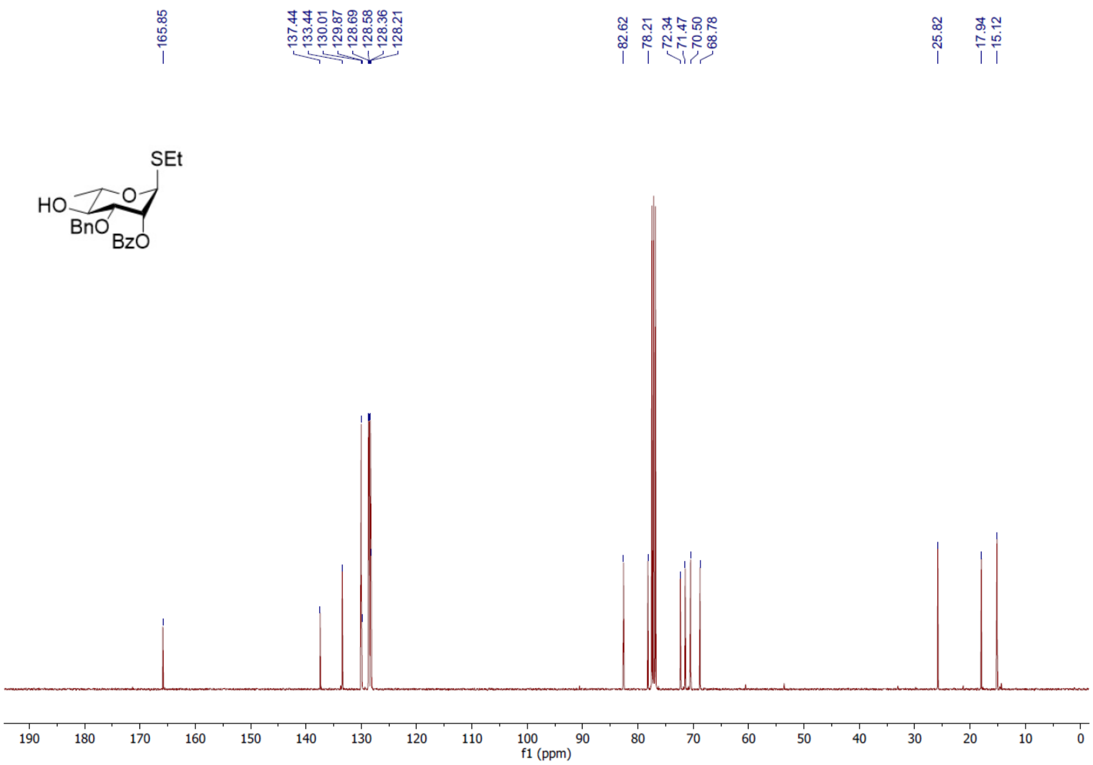

^{13}C NMR of **S-3** (101 MHz, $CDCl_3$)

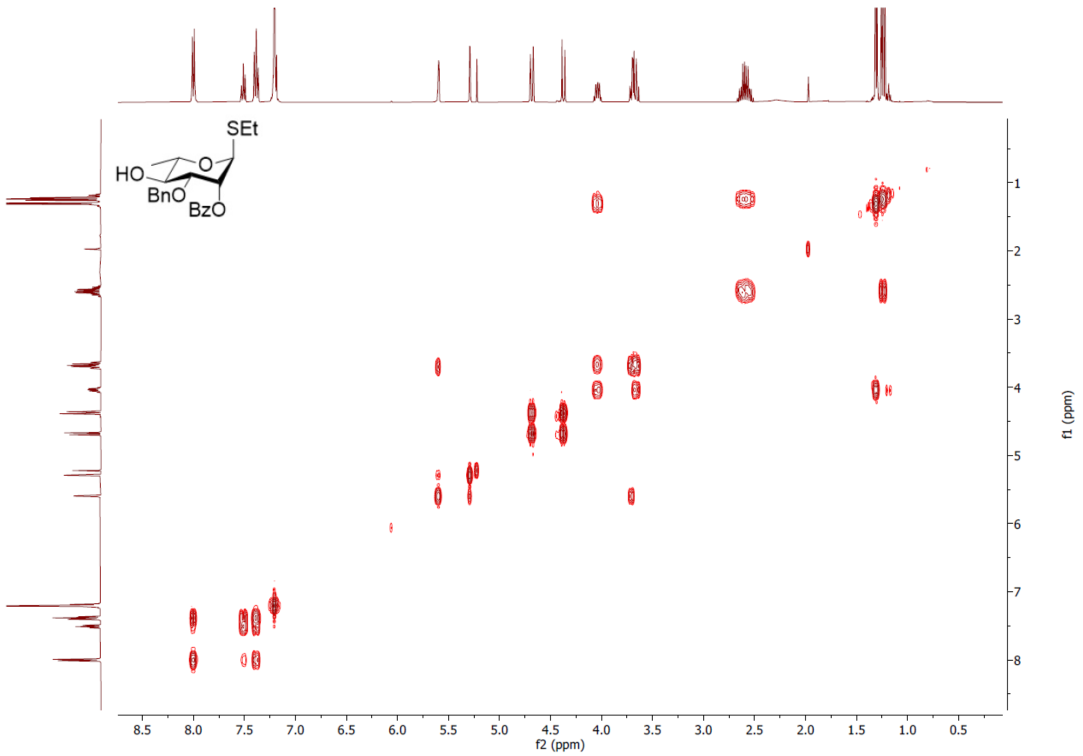

COSY NMR of **S-3** ($CDCl_3$)

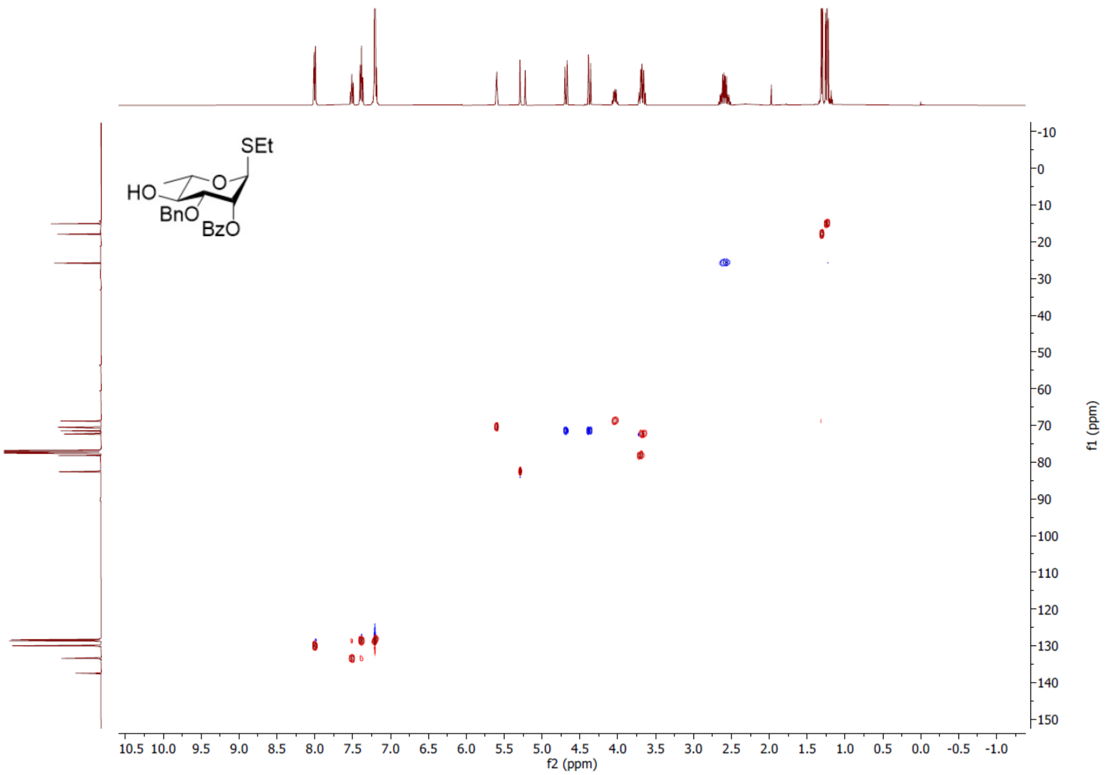

HSQC NMR of **S-3** ($CDCl_3$)

Synthesis of BB5

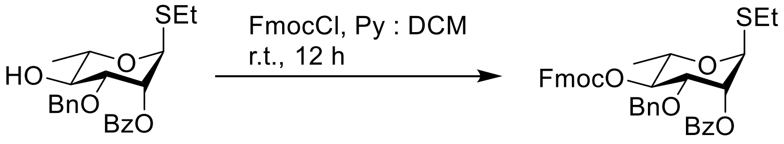

S-3 (2.0 g, 5.0 mmol) was dissolved in a mixture of pyridine (10 mL) and DCM anhydrous (30 mL) and cooled to 0 °C. 9-Fluorenylmethoxycarbonyl chloride was dissolved in DCM (6 mL) and added dropwise to the stirred mixture. The reaction was allowed to room temperature and stirred for additional 12 h. The reaction was quenched with an aqueous solution of HCl (10 %) at 0 °C. The mixture was diluted with DCM and washed with an aqueous solution of HCl (10 %) and brine. The organic layer was dried by Na_2SO_4 and concentrated under reduced pressure. The resulting crude product was purified by silica gel flash column chromatography (Hexane : EA = from 8 : 1 to 3 : 1) to give **BB5** (3.0 g, 96%) as a colorless sticky solid.

^{1}H NMR (400 MHz, $CDCl_3$) δ 8.05 – 7.99 (m, 2H, Ar), 7.70 (d, *J* = 7.5 Hz, 2H, Ar), 7.57 – 7.46 (m, 3H, Ar), 7.41 – 7.30 (m, 4H, Ar), 7.22 (m, 2H, Ar), 7.18 – 7.10 (m, 5H, Ar), 5.56 (dd, *J* = 3.3, 1.6

Hz, 1H, H-2), 5.28 (d, *J* = 1.5 Hz, 1H, H-1), 4.95 (t, *J* = 9.8 Hz, 1H, H-4), 4.61 (d, *J* = 12.1 Hz, 1H, CH_2-Bn), 4.45 (d, *J* = 12.1 Hz, 1H, CH_2-Bn), 4.38 (d, *J* = 7.2 Hz, 2H, CH_2-Fmoc), 4.25 – 4.14 (m, 2H, H-5, CH-Fmoc), 3.89 (dd, *J* = 9.7, 3.3 Hz, 1H, H-3), 2.66 – 2.48 (m, 2H, CH_2-SEt), 1.25 – 1.19 (m, 6H, H-6, CH_3-SEt). ^{13}C NMR (101 MHz, $CDCl_3$) δ 165.88, 155.04, 143.53, 143.29, 141.44, 141.41, 137.48, 133.48, 130.08, 129.71, 128.62, 128.45, 128.03, 127.89, 127.88, 127.31, 125.22, 125.20, 120.22, 82.48 (C-1), 77.55, 75.12, 71.52, 71.17, 70.09, 67.02, 46.93, 25.87, 17.61, 15.10. ESI-HRMS m/z 647.2101 $[M+Na]^+$ ($C_{37}H_{36}NaO_7S$ requires 647.2079).

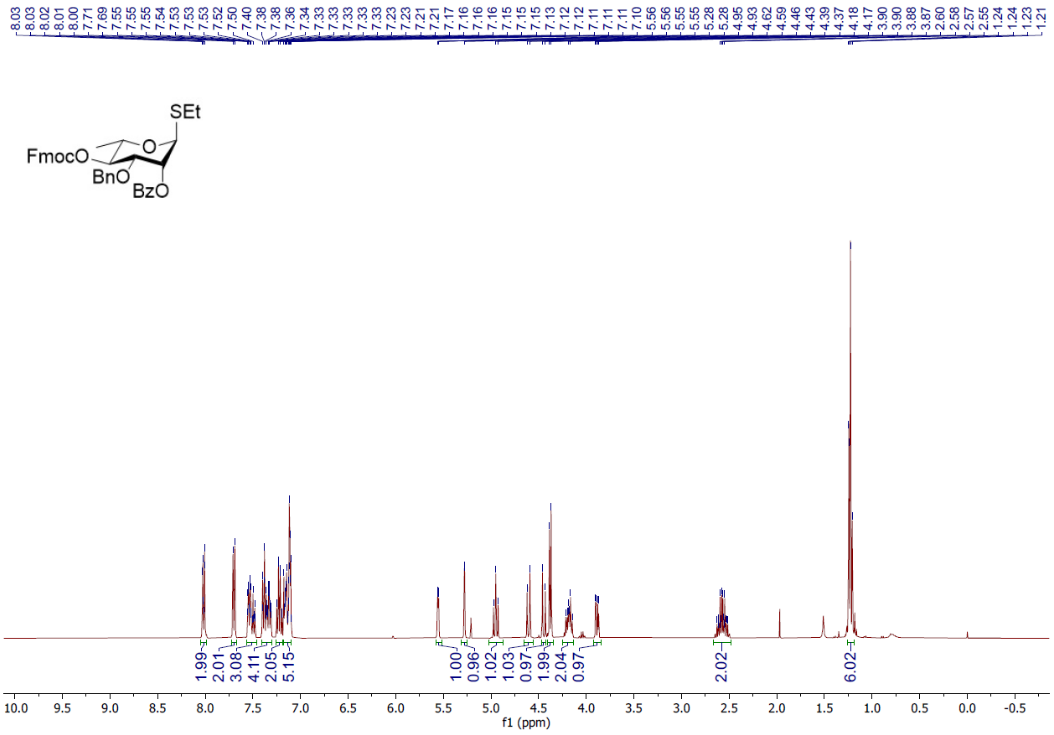

^{1}H NMR of **BB5** (400 MHz, $CDCl_3$)

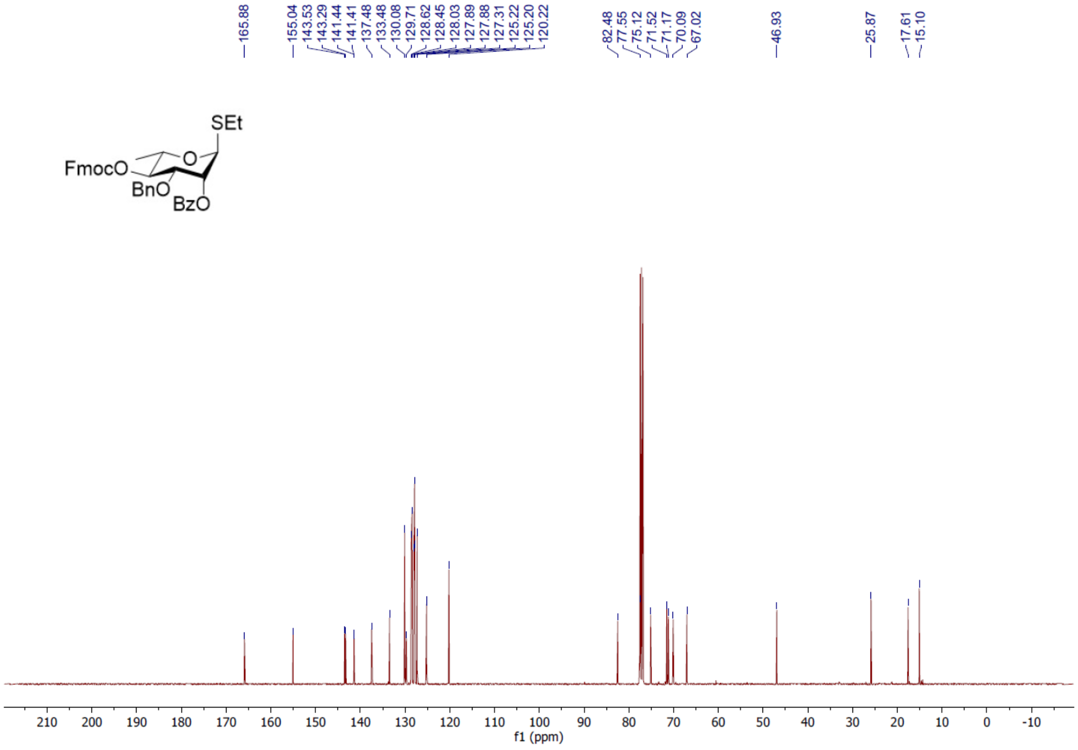

^{13}C NMR of **BB5** (101 MHz, $CDCl_3$)

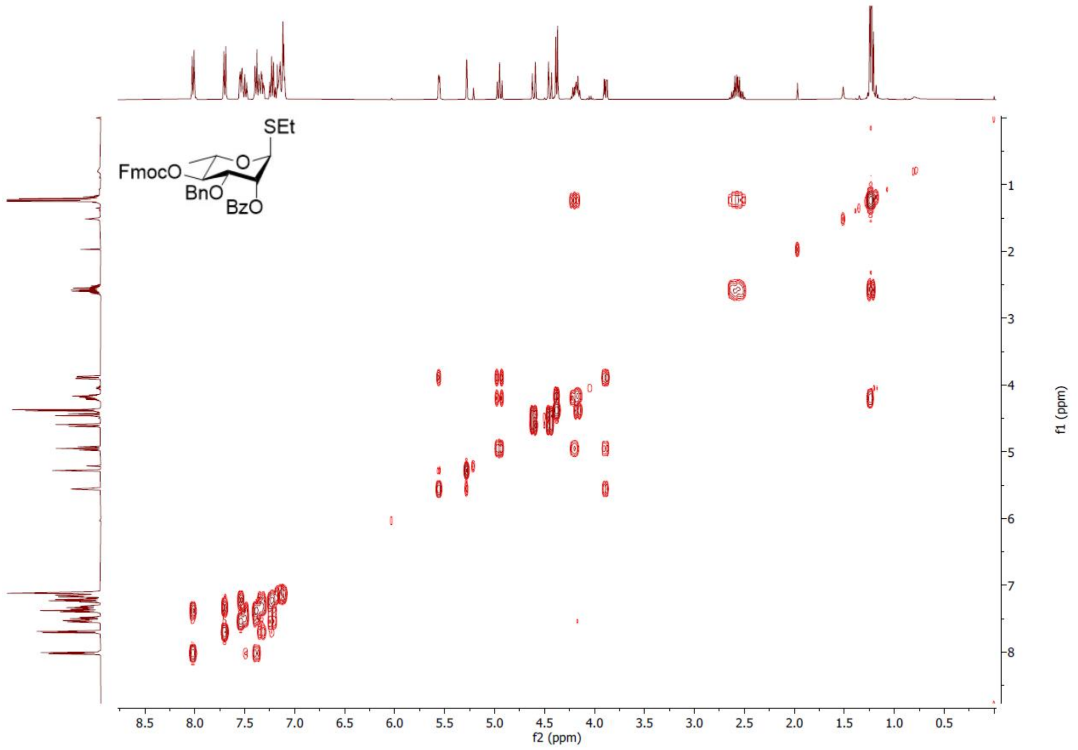

COSY NMR of **BB5** ($CDCl_3$)

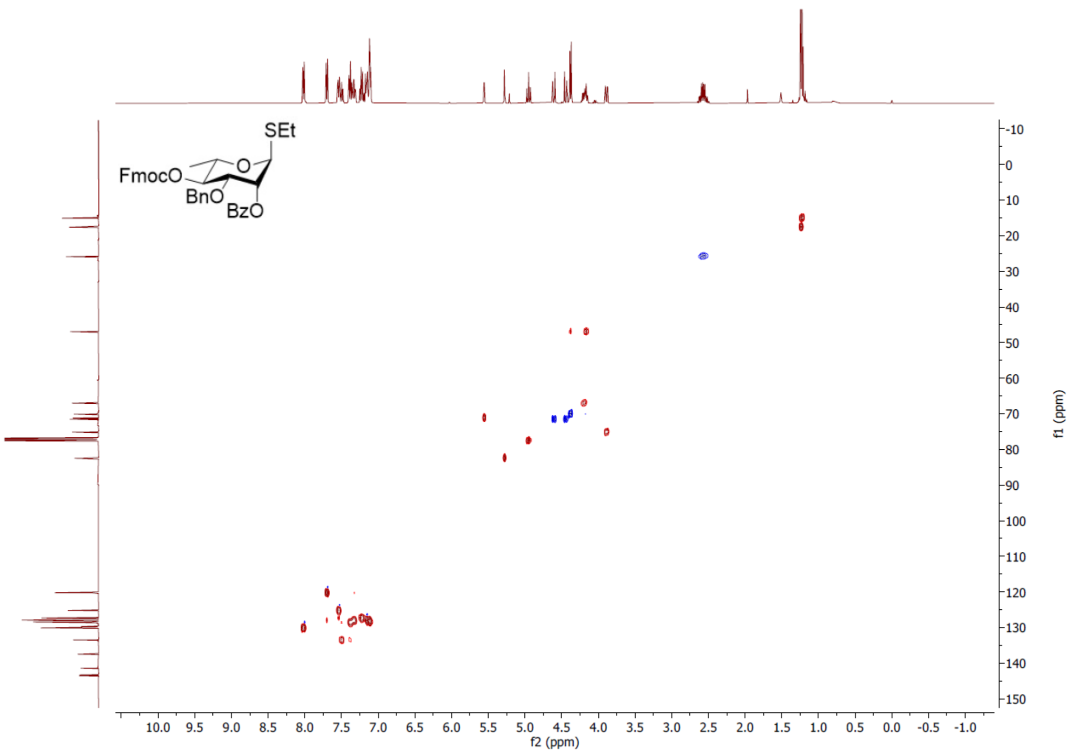

HSQC NMR of **BB5** ($CDCl_3$)

2.4 Synthesis of BB7

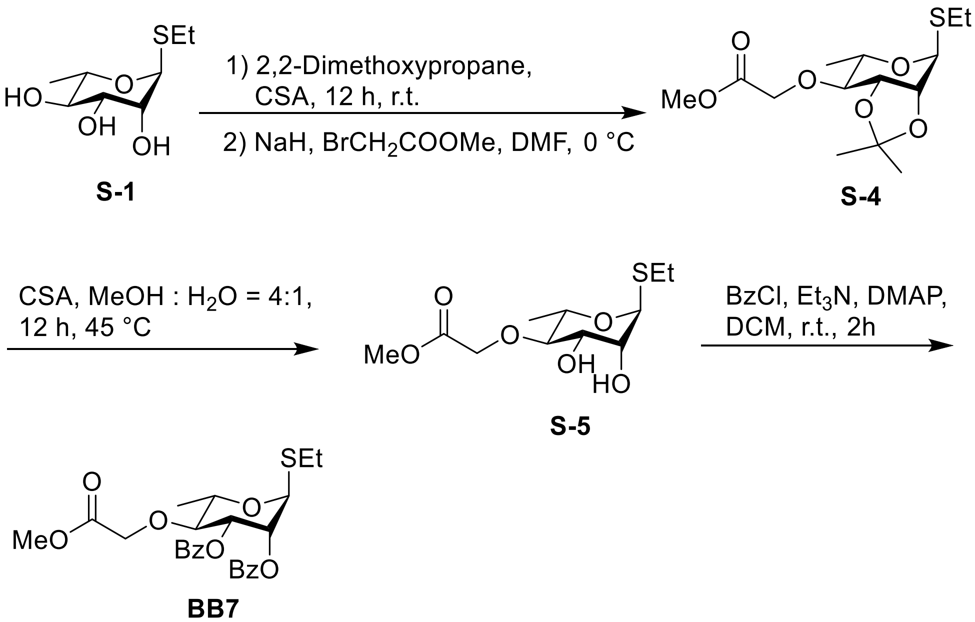

Figure S3 Synthesis of **BB7**.

Synthesis of S-4

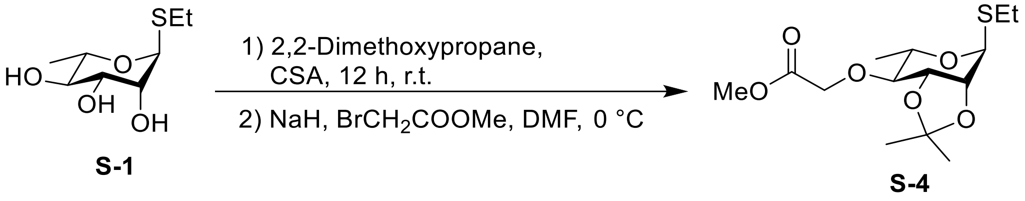

To a solution of **S-1** (10.0 g, 0.05 mol) in 2,2-dimethoxypropane (60 mL, 0.49 mol) at room temperature, (1S)-(+)-10-camphorsulfonic acid (47.00 mg, 0.20 mmol) was added while stirring. After 12 h, the reaction was quenched with N, N-diethylethanamine and concentrated under reduced pressure to obtain a sticky colorless liquid. The resulting crude product was used for next step directly without further purification. The crude product was dissolved in anhydrous DMF (60 mL) and methyl 2-bromoacetate (7.1 mL, 0.075 mol) was added. The mixture was cooled to 0 °C and sodium hydride was added slowly (60% in mineral oil, 3.0 g, 0.075 mmol)**.** The reaction was allowed to room temperature and stirred for additional 2.5 h. A second addition of methyl 2-bromoacetate (7.1 mL, 0.075 mol) and sodium hydride (3.0 g, 0.075 mmol) was performed and the above described operation repeated. TLC was used to monitor the reaction progress. After completion, the reaction was quenched with methanol at 0 °C, diluted with ethyl acetate (300 mL), and washed two times with brine. The organic layer was dried with Na_2SO_4 and concentrated. The resulting crude product was purified by silica gel flash column chromatography (Hexane : EA = 7 : 1) to give **S-4** as a white solid (14.3 g, 89%).

^{1}H NMR (400 MHz, $CDCl_3$) δ 5.43 (s, 1H, H-1), 4.31 (d, *J* = 17 Hz, 2H, CH_2COOMe), 4.14 (dd, *J* = 7.3, 5.6 Hz, 1H, H-3), 4.08 (d, *J* = 5.6 Hz, 1H, H-2), 4.01 – 3.93 (m, 1H, H-5), 3.69 (s, 3H, OMe), 3.22 (dd, *J* = 9.8, 7.3 Hz, 1H, H-4), 2.63 – 2.54 (m, 1H, CH_2-SEt), 2.46 (m, 1H, CH_2-SEt), 1.44 (s, 3H, CH_3), 1.29 – 1.18 (m, 9H, CH_3, CH_3-SEt, H-6). ^{13}C NMR (101 MHz, $CDCl_3$) δ 169.90, 108.60, 81.88, 78.56 (C-1), 77.10, 76.64, 67.31, 64.08, 51.01, 27.27, 25.64, 23.61, 16.85, 13.89. ESI-HRMS m/z 343.1180 $[M+Na]^+$ ($C_{14}H_{24}NaO_6S$ requires 343.1191).

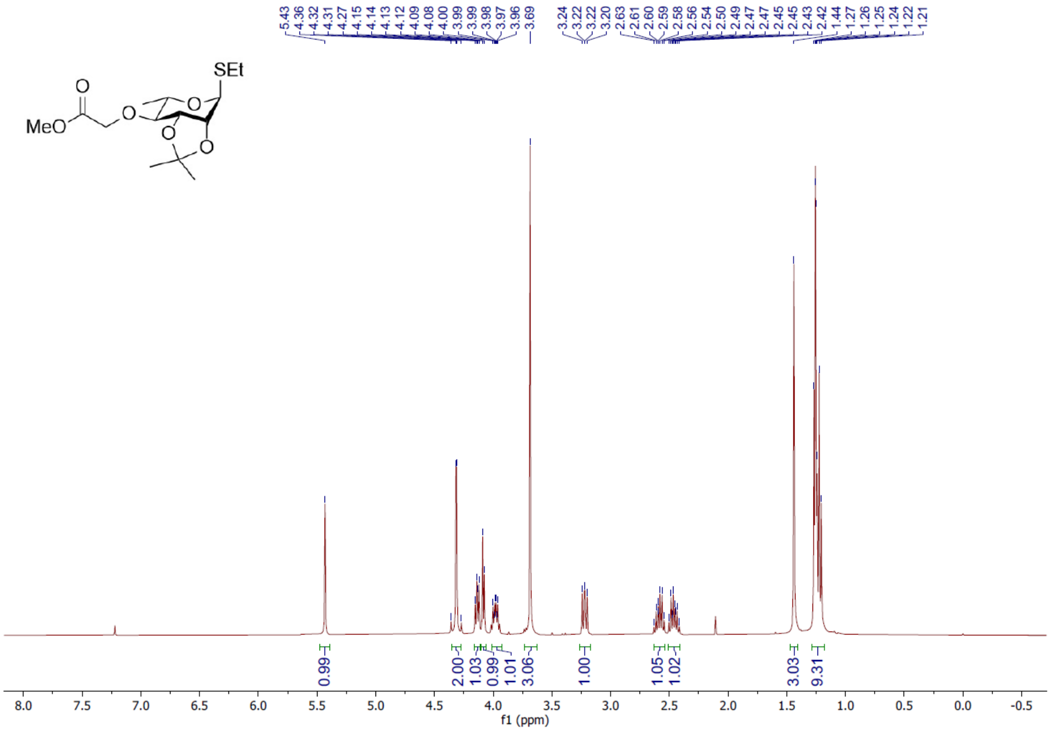

^{1}H NMR of **S-4** (400 MHz, $CDCl_3$)

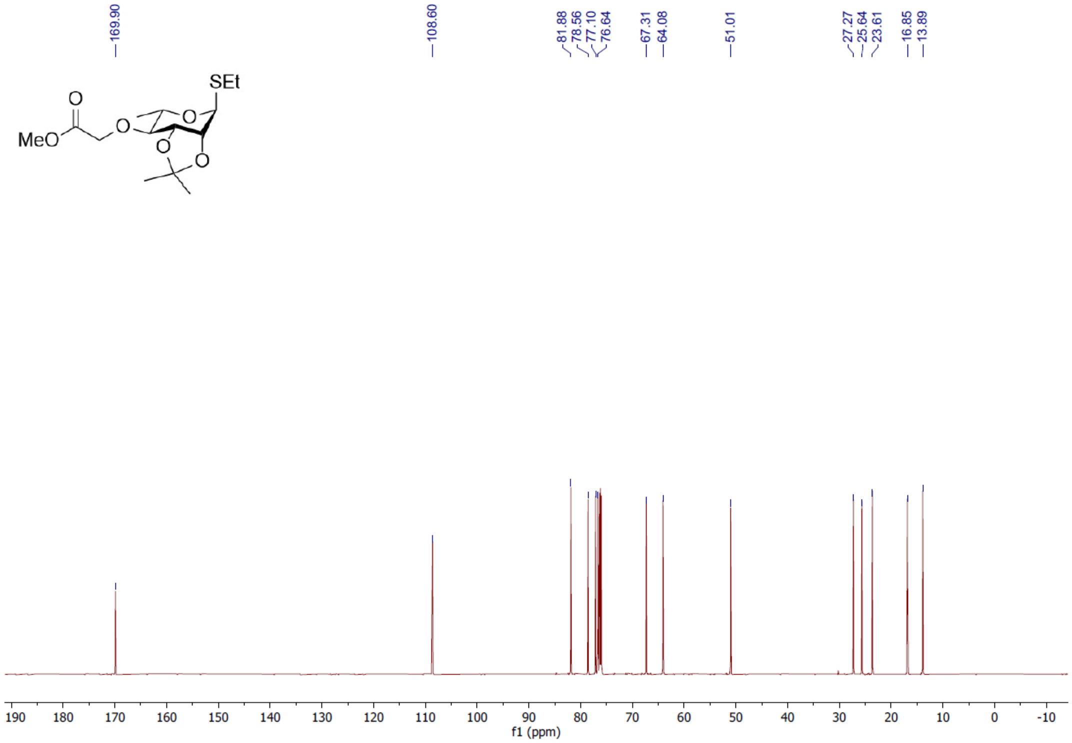

^{13}C NMR of **S-4** (101 MHz, $CDCl_3$)

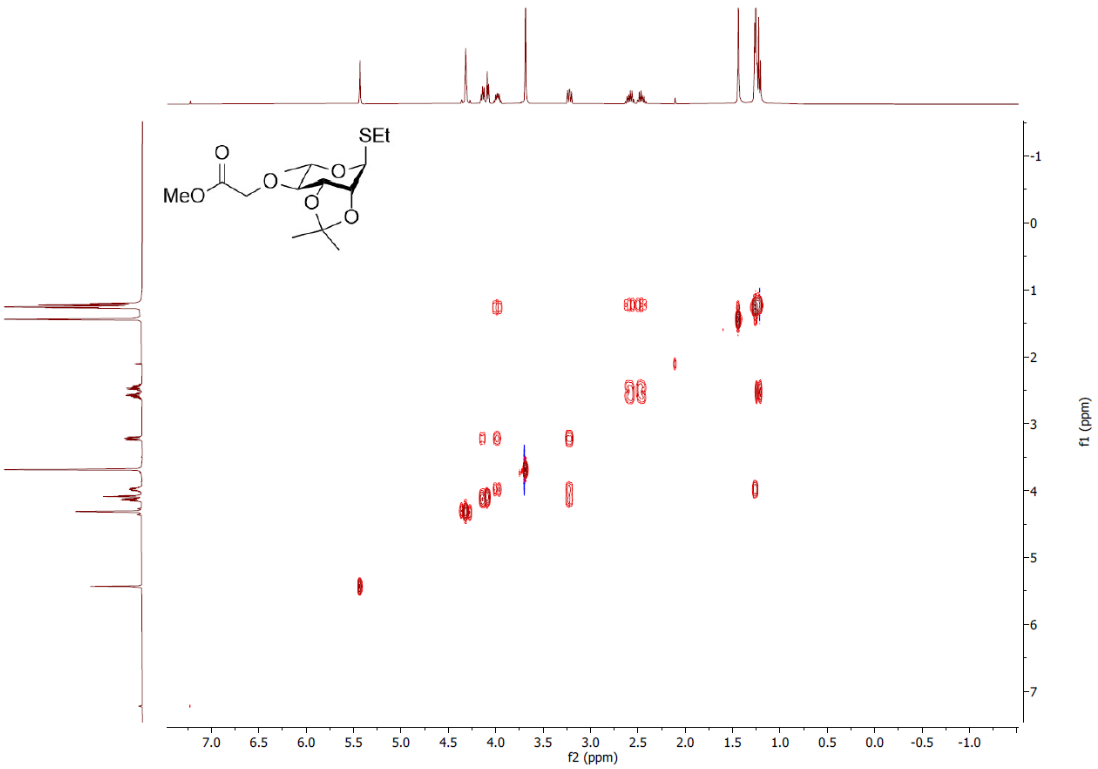

COSY NMR of **S-4** ($CDCl_3$)

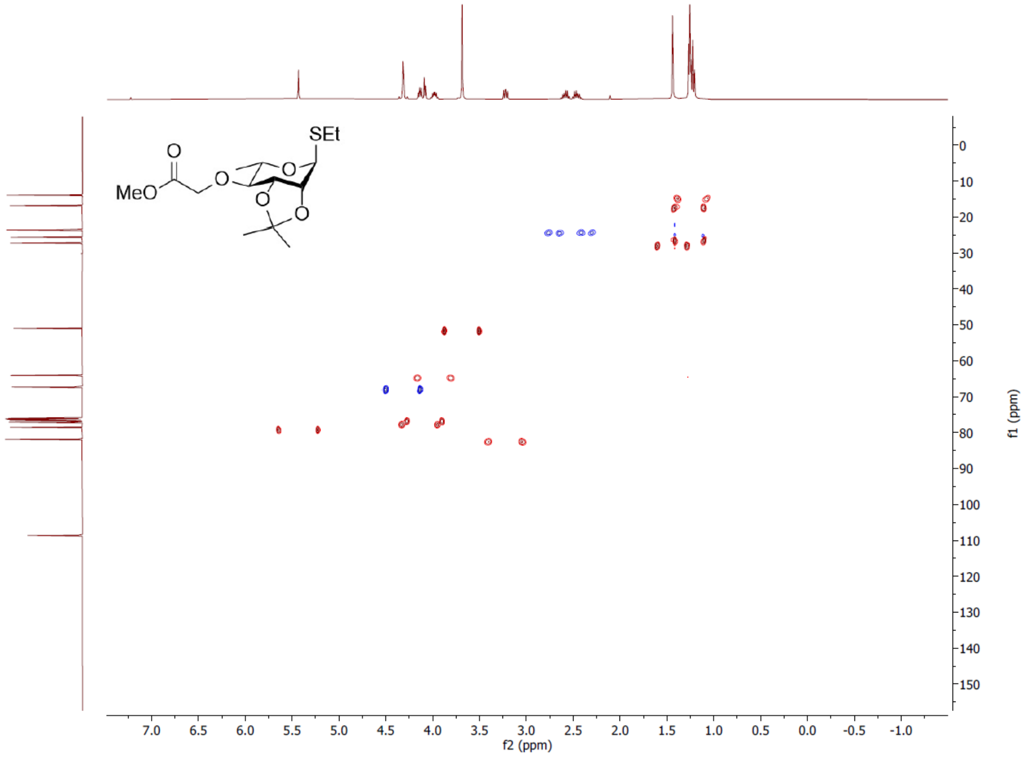

HSQC NMR of **S-4** ($CDCl_3$)

Synthesis of BB7

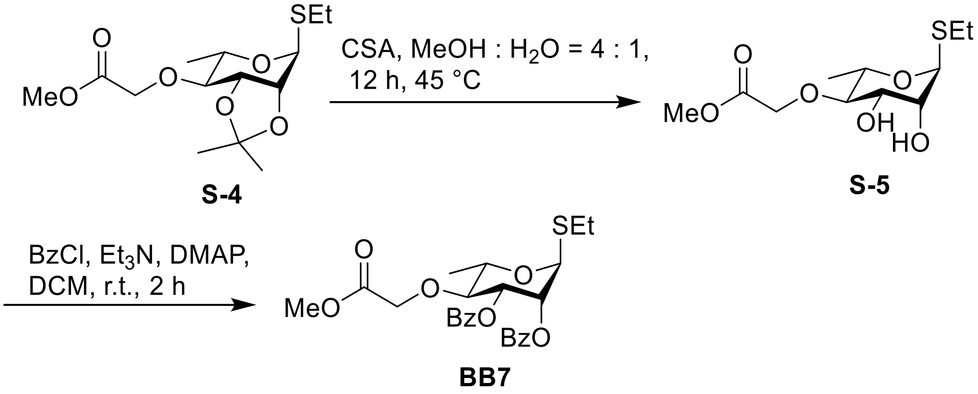

S-4 (10.0 g, 0.031 mmol) was dissolved in a mixture of MeOH: H_2O (40 mL: 10 mL) and (1S)-(+)-10-camphorsulfonic acid (47.00 mg, 0.20 mmol) was added. The mixture was heated to 45 °C for 12 h. After completion, the reaction was quenched with N, N-diethylethanamine and concentrated under reduced pressure. The resulting crude product was purified by silica gel flash column chromatography (Hexane : EA = from 7 : 1 to 2 : 1) to give the intermediate **S-5** as a white solid (2.8 g, 32%). To a solution of **S-5** (2.8 g, 10.0 mmol) in DCM (15 mL) N, N-diethylethanamine (2.1 mL, 15.0 mmol) and 4-dimethylaminopyridine (60.0 mg, 0.49 mmol) were added. The mixture was cooled to 0 °C and benzoylchlorid (1.7 mL, 15.0 mmol) was added dropwise. The reaction was allowed to room temperature and stirred for additional 2 h. After completion, the reaction was quenched with MeOH at 0 °C and then concentrated under reduced pressure. The crude product was purified by silica gel flash column chromatography (Hexane : EA = from 7 : 1 to 2 : 1) to give **BB7** (3.2 g, 66%) as a light-yellow solid.

^{1}H NMR (400 MHz, $CDCl_3$) δ 7.97 – 7.92 (m, 2H, Ar), 7.85 – 7.80 (m, 2H, Ar), 7.57 – 7.49 (m, 1H, Ar), 7.48 – 7.43 (m, 1H, Ar), 7.40 (t, *J* = 7.7 Hz, 2H, Ar), 7.29 (t, *J* = 7.8 Hz, 2H, Ar), 5.64 (dd, *J* = 3.4, 1.6 Hz, 1H, H-2), 5.55 (dd, *J* = 9.5, 3.4 Hz, 1H, H-3), 5.30 (d, *J* = 1.6 Hz, 1H, H-1), 4.43 – 4.24 (m, 3H, H-5, CH_2COOMe), 3.68 (t, *J* = 9.4 Hz, 1H, H-4), 3.44 (s, 3H, OMe), 2.62 (m, 2H, CH_2-SEt), 1.42 (d, *J* = 6.2 Hz, 3H, H-6), 1.26 (t, *J* = 7.4 Hz, 3H, CH_3-SEt). ^{13}C NMR (101 MHz, $CDCl_3$) δ 170.24, 165.50, 165.15, 133.55, 133.37, 129.90, 129.68, 128.66, 128.57, 82.05 (C-1), 81.19, 73.07, 72.78, 70.14, 68.03, 51.87, 25.67, 18.15, 15.08. ESI-HRMS m/z 511.1406 $[M+Na]^+$ ($C_{25}H_{28}NaO_8S$ requires 511.1403).

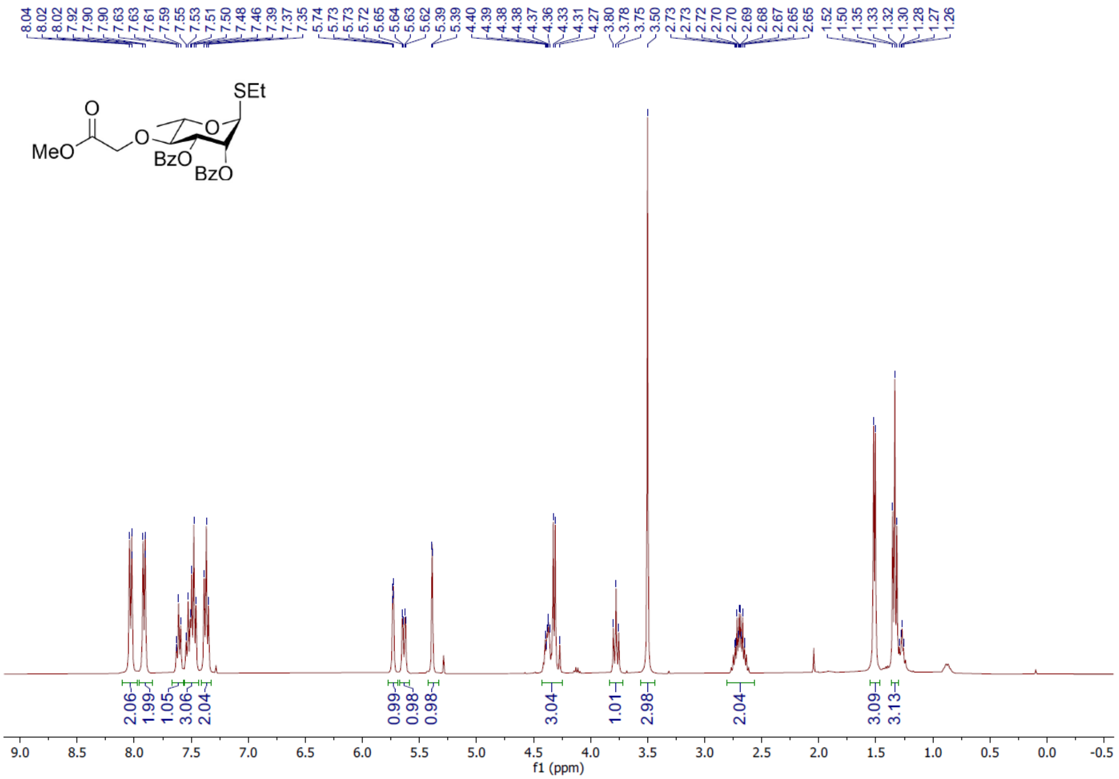

^{1}H NMR of **BB7** (400 MHz, $CDCl_3$)

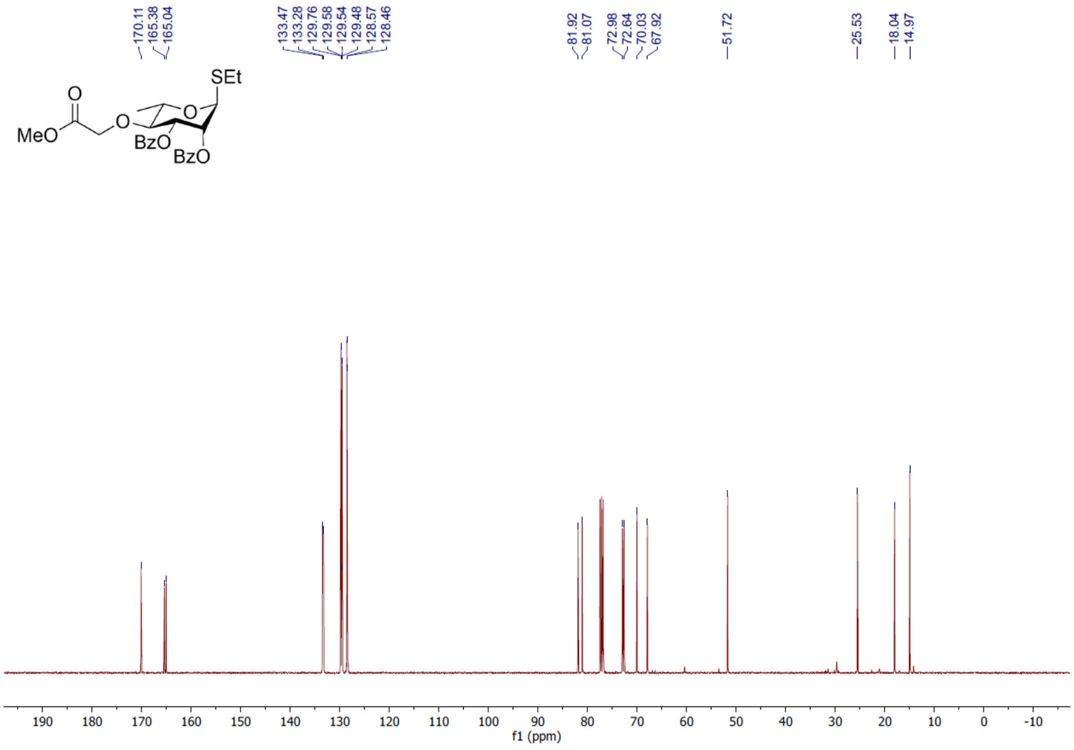

^{13}C NMR of **BB7** (101 MHz, $CDCl_3$)

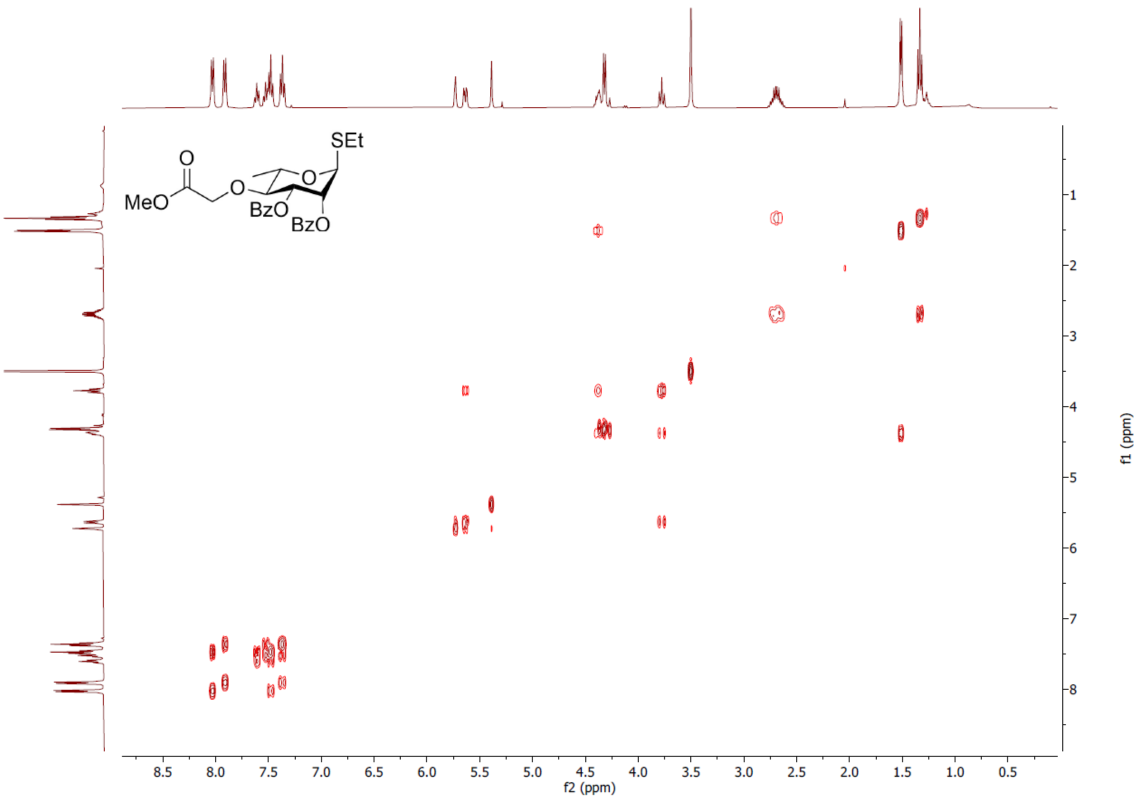

COSY NMR of **BB7** ($CDCl_3$)

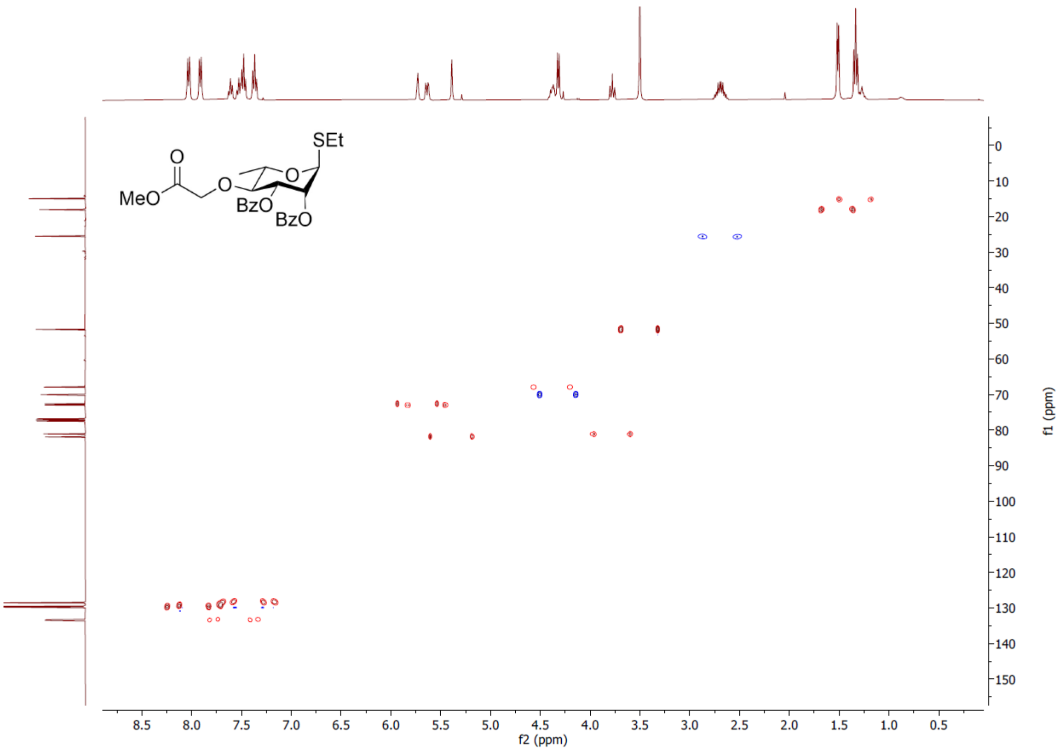

HSQC NMR of **BB7** ($CDCl_3$)

3 Automated glycan assembly (AGA)

3.1 General materials and method

The automated syntheses were performed on a home-built synthesizer developed at the Max Planck Institute of Colloids and Interfaces.[1] All solvents used were HPLC-grade. The solvents used for the BBs, activator, TMSOTf and capping solutions were taken from an anhydrous solvent system (J.C. Meyer) and further dried with molecular sieves (4 Å) for moisture-sensitive solutions. The building blocks were co-evaporated three times with toluene and dried for 1 h on high vacuum before use. Oven-heated, argon-flushed flasks were used to prepare all moisture-sensitive solutions. Activator, capping, deprotection, acidic wash and building block solutions were freshly prepared and kept under argon during the automation run. All yields of products obtained by AGA were calculated on the basis of resin loading. Resin loading was determined following previously established procedures.[5]

3.2 Preparation of stock solutions

- **Building block solution**: Between 0.06 and 0.10 mmol of building block (depending on the BB, see Module C1 and C2) was dissolved in DCM (1 mL).
- **NIS/TfOH activator solution**: 1.35 g (6.0 mmol) of recrystallized NIS was dissolved in 40 mL of a 2 :1 v/v mixture of anhydrous DCM and anhydrous dioxane. Then triflic acid (55 μL, 0.6 mmol) was added. The solution was kept at 0 °C for the duration of the automation run.
- **Fmoc deprotection solution**: A solution of 20% piperidine in DMF (v/v) was prepared.
- **Lev deprotection solution**: Hydrazine acetate (550 mg, 5.97 mmol) was dissolved in pyridine/AcOH/H_2O (40mL, v/v, 30 : 8 : 2) and sonicated for 10 min.
- **TMSOTf solution**: TMSOTf (0.45 mL, 2.49 mmol) was added to DCM (40 mL).
- **Capping solution**: A solution of 10% acetic anhydride and 2% methanesulfunic acid in DCM (v/v) was prepared.

3.3 Modules for automated synthesis

3.3.1 Module A: Resin preparation for synthesis (20 min)

All automated syntheses were performed on 0.019 mmol scale. Resin **L** was placed in the reaction vessel and swollen in DCM for 20 min at room temperature prior to synthesis. During this time, all

reagent lines needed for the synthesis were washed and primed. After the swelling, the resin was washed with DMF, THF, and DCM (three times each with 2 mL for 25 s).

3.3.2 Module B: Acidic wash with TMSOTf solution (20 min)

The resin was swollen in 2 mL DCM and the temperature of the reaction vessel was adjusted to -20 °C. Upon reaching the low temperature, TMSOTf solution (1 mL) was added drop wise to the reaction vessel. After bubbling for 3 min, the acidic solution was drained and the resin was washed with 2 mL DCM for 25 s.

Action	Cycles	Solution	Amount	T (°C)	Incubation time
Cooling	-	-	-	-20	(15 min)*
Deliver	1	DCM	2 mL	-20	-
Deliver	1	TMSOTf solution	1 mL	-20	3 min
Wash	1	DCM	2 mL	-20	25 sec

*Time required to reach the desired temperature.

3.3.3 Module C1: Thioglycoside glycosylation (2 h – 3 h)

The building block solution (0.08 mmol of BB in 1 mL of DCM per glycosylation) was delivered to the reaction vessel. After the set temperature was reached, the reaction was started by dropwise addition of the NIS/TfOH activator solution (1.0 mL, excess). The glycosylation conditions (T_1, T_2, t_1, and t_2) are building block dependent and are reported in a table below. After completion of the reaction, the solution was drained and the resin was washed with DCM (3 mL for 15 s), dioxane (2 mL) and DCM (2 mL for 15 s). The temperature of the reaction vessel was increased to 25 °C for the next module. This procedure was repeated two times.

Action	Cycles	Solution	Amount	T (°C)	Incubation time
Cooling	-	-	-	T_1	-
Deliver	1	BB solution	1 mL	T_1	-
Deliver	1	NIS/TfOH activator solution	1 mL	T_1	-
Reaction time (BB dependent)	1			T_1 to T_2	t_1 t_2
Wash	1	DCM	3 mL	T_2	15 sec
Wash	1	Dioxane	2 mL	T_2	-
Heating	-	-	-	25	-
Wash	2	DCM	2 mL	> 0	15 sec

BB	Equiv.	t_1 (min)	T_1 (°C)	t_2 (min)	T_2 (°C)
**1**	4.3	20	-20	40	0
**4**	6.5	20	-20	40	0
**5**	4.3	20	-20	40	0
**6**	6.5	30	-20	50	0
**7**	4.3	20	-20	40	0

3.3.4 Module C2: Glycosyl phosphate glycosylation (2.5 h)

The building block solution (0.06 mmol of BB in 1 mL of DCM per glycosylation) was delivered to the reaction vessel. After the set temperature was reached, the reaction was started by dropwise addition of the TMSOTf solution (1.0 mL, stoichiometric). After completion of the reaction, the solution was drained and the resin washed with DCM (six times, each with 2 mL for 25 s). The temperature of the reaction vessel was increased to 25 °C for the next module. This procedure was repeated two times.

Action	Cycles	Solution	Amount	T (°C)	Incubation time
Cooling	-	-	-	-30	-
Deliver	1	BB solution	1 mL	-30	-
Deliver	1	TMSOTf solution	1 mL	-30	-
Reaction time (BB dependent)	1			-30 to -10	30 min 40 min
Wash	1	DCM	2 mL	-10	5 sec
Heating	-	-	-	25	-
Wash	6	DCM	2 mL	> 0	25 sec

BB	Equiv.	t_1 (min)	T_1 (°C)	t_2 (min)	T_2 (°C)
**2**	3.3	30	-30	40	-10
**3**	3.3	30	-30	40	-10

3.3.5 Module D: Capping (30 min)

The resin was washed with DMF (two times with 2 mL for 25 s) and the temperature of the reaction vessel was adjusted to 25 °C. 2 mL of pyridine solution (10% in DMF) was delivered into the reaction vessel. After 1 min, the reaction solution was drained and the resin washed with DCM

(three times with 3 mL for 25 s). 4 mL of capping solution was delivered into the reaction vessel. After 20 min, the reaction solution was drained and the resin washed with DCM (three times with 3 mL for 25 s).

Action	Cycles	Solution	Amount	T (°C)	Incubation time
Heating	-	-	-	25	(5 min)*
Wash	2	DMF	2 mL	25	25 sec
Deliver	1	10% Pyridine in DMF	2 mL	25	1 min
Wash	3	DCM	3 mL	25	25 sec
Deliver	1	Capping Solution	4 mL	25	20 min
Wash	3	DCM	3 mL	25	25 sec

*Time required to reach the desired temperature.

3.3.6 Module E1: Fmoc deprotection (18 min)

The resin was washed with DMF (three times with 2 mL for 25 s) and the temperature of the reaction vessel was adjusted to 25 °C. 2 mL of Fmoc deprotection solution was delivered to the reaction vessel and kept under Ar bubbling. After 5 min, the reaction solution was drained and the resin washed with DMF (three times with 3 mL for 25 s) and DCM (five times each with 2 mL for 25 s). The temperature of the reaction vessel was decreased to -20 °C for the next module. This procedure was repeated two times.

Action	Cycles	Solution	Amount	T (°C)	Incubation time
Wash	3	DMF	2 mL	25	25 sec
Deliver	1	Fmoc depr. solution	2 mL	25	5 min
Wash	1	DMF	3 mL		
Cooling	-	-	-	-20	-
Wash	3	DMF	3 mL	< 25	25 sec
Wash	5	DCM	2 mL	< 25	25 sec

3.3.7 Module E2: Lev deprotection (95 min)

The resin was washed with DCM (three times with 2 mL for 25 s). DCM (1.3 mL) was delivered to the reaction vessel and the temperature of the reaction vessel was adjusted to 40 °C. 2 mL of Lev deprotection solution was delivered to the reaction vessel that was kept under pulsed Ar bubbling for 30 min. This procedure was repeated three times. The reaction solution was drained

and the resin washed with DMF (three times with 3 mL for 25 s) and DCM (five times each with 2 mL for 25 s).

Action	Cycles	Solution	Amount	T (°C)	Incubation time
Wash	3	DMF	2 mL	25	25 sec
Deliver	2	Lev depr. solution	2 mL	25	30 min
Wash	1	DMF	2 mL		
Cooling	-	-	-	-20	-
Wash	3	DMF	3 mL	< 25	25 sec
Wash	5	DCM	2 mL	< 25	25 sec

3.4 Post-synthesizer manipulations (Post-AGA)

3.4.1 Module F: The coupling of P-O bond on resin

Diisopropylamine (200 µL, 1.4 mmol) was added dropwise to a solution of dichlorophosphite (42 µL, 0.4 mmol) in Et_2O (1.5 mL). The mixture was kept at room temperature for 20 min then the organic salts were filtered and washed two times with Et_2O (0.4 mL×2). The organic layer and the tetrazole solution (0.45 M in ACN, 3.6 mL) were added to the resin (0.019 mmol scale) and the mixture was gently shaken for 24 h at room temperature. After microcleavage (see **Module I2**), MALDI analysis indicated completion of. The resin was repeatedly washed with ACN (2 mL x 3) and DCM (2 mL x 3).

3.4.2 Module G: Oxidation on resin

The resin was swelled in pyridine (2.6 mL). A solution of I_2 in pyridine (0.24 M, 1.5 mL) and H_2O (2.1 mL) were added to resin. The mixture was gently shaken at room temperature for 4 h. After microcleavage (see **Module I2**), MALDI analysis indicated completion of the oxidation. The resin was repeatedly washed with pyridine (2 mL x 5) and DCM (2 mL x 3).

3.4.3 Module H: Sulfation on resin

The resin was suspended in 5 mL of a 1 M SO_3·pyridine in pyridine. The reaction was rotated for 12 h at 45 °C. After microcleavage (see **Module I2**), MALDI analysis indicated completion of sulfation. The resin was repeatedly washed with pyridine (2 mL x 5) and DCM (2 mL x 3).

3.4.4 Module I1: Cleavage from solid support

The oligosaccharides were cleaved from the solid support using a continuous-flow photoreactor as described previously.[6]

3.4.5 Module I2: Micro-cleavage from solid support

Trace amount of resin (around 20 beads) was dispersed in DCM (0.1 mL) and irradiated with a UV lamp (6 watt, 356 nm) for 10 minutes. ACN (10 µL) was then added to the resin and the resulting solution analyzed by MALDI.

3.4.6 Module J1: Solution-phase methanolysis

The product was dissolved in THF (4 mL). NaOMe in MeOH (0.5 M, 0.6 mL) was added and the mixture was stirred at room temperature. After completion, amberlite IR 120 acid resin was added to adjust the pH to 7.0 and then the resin was filtered and washed with MeOH (2mL x 3). The resulting solution was concentrated *in vacuo.*

3.4.7 Module J2: Solution-phase hydrolysis

The product was dissolved in a mixture of THF and MeOH (4 mL, 4 : 1). 1.5 mL of LiOH in H_2O (1 M) was added and the mixture was stirred at room temperature for 12 h. After completion, amberlite IR 120 acid resin was added to adjust the pH to 7.0 and then the resin was filtered and washed with MeOH (2mL x 3). The resulting solution was concentrated *in vacuo.*

3.4.8 Module K1: Hydrogenolysis

The crude compound was dissolved in 2.5 mL of EA : *t*BuOH : H_2O (0.5 : 1 : 1). 100% by weight $Pd(OH)_2$-C (10% weight) was added and the reaction was stirred in a pressurized reactor under H_2 atmosphere (60 psi). The reaction progress was monitored to avoid undesired side products formation. Upon completion, the reaction was filtered and washed with EA, *t*BuOH and H_2O. The filtrates were concentrated *in vacuo.*

3.4.9 Module K2: Hydrogenolysis at ambient pressure

The crude compound obtained from last step was dissolved in 2.5 mL of EA : *t*BuOH : H_2O (0.5 : 1 : 1). 100% by weight $Pd(OH)_2$-C (10% weight) was added and the reaction was stirred in a flask equipped with a H_2 balloon. The reaction progress was monitored to avoid undesired side products formation. Upon completion, the reaction was filtered and washed with EA, *t*BuOH and H_2O. The filtrates were concentrated *in vacuo.*

3.4.10 Module L: Purification

The purification of the crudes was conducted using a silica gel flash column chromatography (for protected compound), reverse phase HPLC (Agilent 1200 Series, for global deprecation compound). The pure compound was analyzed using analytical HPLC (Agilent 1200 Series).

- **Method A:** The purification of protected crudes was conducted using a silica gel flash column chromatography (Hexane : EA = 1 : 1 for **3mer**, **4mer-I**, **4mer-IV**, **4mer-V** and **5 mer** or DCM : MeOH = 15 : 1 for **4mer-II**, **4mer-III**).
- **Method B1:** (Hypercarb column, 150 x 4.6 mm, 3 µm) flow rate of 0.7 mL/min with H_2O (0.1% formic acid) as eluents [isocratic (5 min), linear gradient to 30% ACN (30 min), linear gradient to 100% ACN (10 min)].
- **Method B2:** (Hypercarb column, 150 x 4.6 mm, 3 µm) flow rate of 0.7 mL/min with H_2O (0.1% formic acid) as eluents [isocratic (5 min), linear gradient to 30% ACN (60 min), linear gradient to 100% ACN (10 min)].
- **Method C1:** (Hypercarb column, 150 x 10 mm, 5 µm), flow rate of 3 mL/min with H_2O (0.1% formic acid) as eluents [isocratic (5 min), linear gradient to 30% ACN (30 min), linear gradient to 100% ACN (10 min)].
- **Method C2:** (Hypercarb column, 150 x 10 mm, 5 µm), flow rate of 3 mL/min with H_2O (0.1% formic acid) as eluents [isocratic (5 min), linear gradient to 30% ACN (60 min), linear gradient to 100% ACN (10 min)].
- **Method C3:** (Hypercarb column, 150 x 10 mm, 5 µm), flow rate of 3 mL/min with H_2O (0.1% formic acid) as eluents [isocratic (5 min), linear gradient to 60% ACN (60 min), linear gradient to 100% ACN (10 min)].

Following final purification, all deprotected products were lyophilized on a Christ Alpha 2-4 LD plus freeze dryer prior to characterization.

3.5 Oligosaccharides synthesis

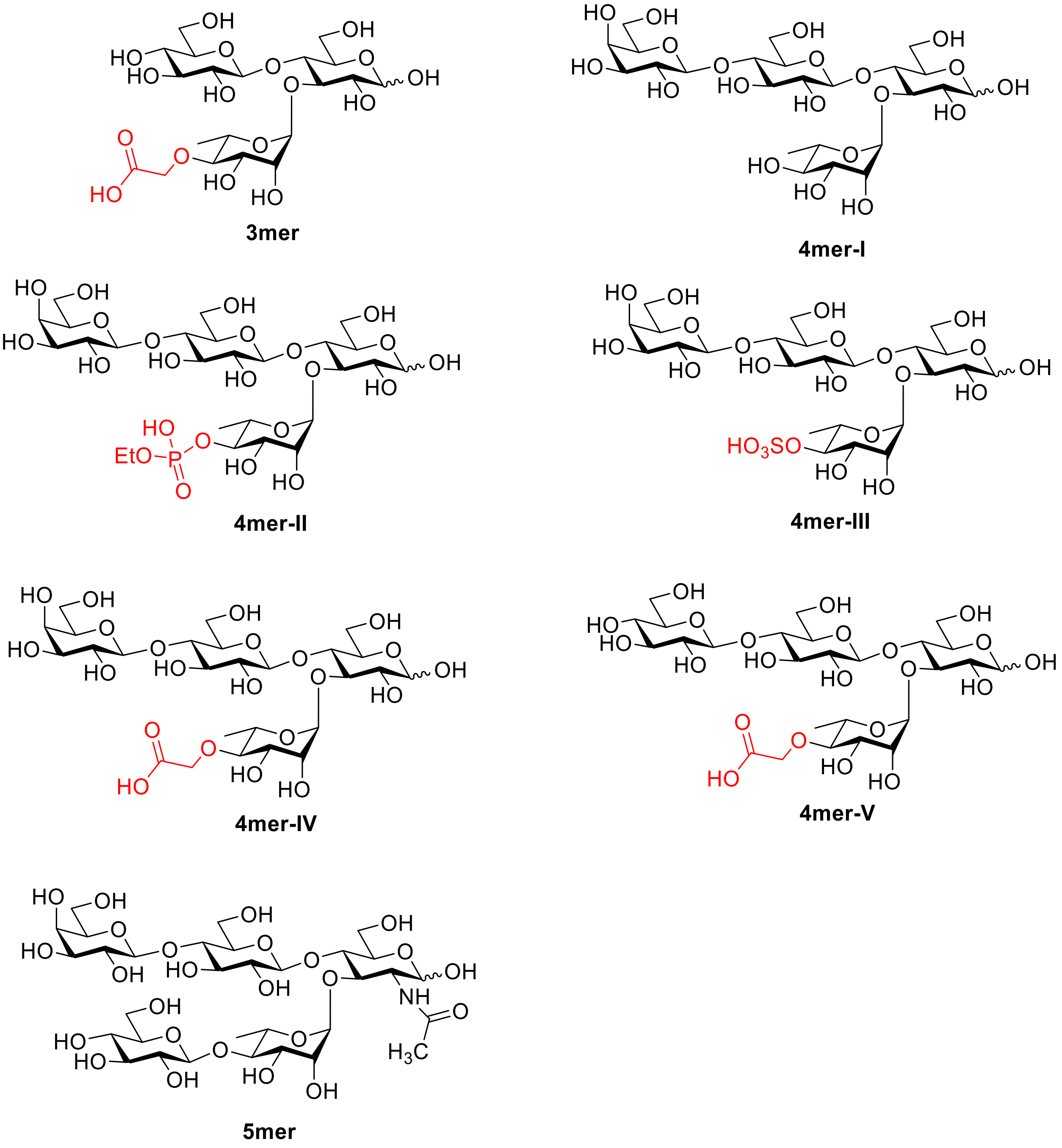

Figure S4 Collection of oligosaccharides synthesized in this work.

3.5.1 3mer

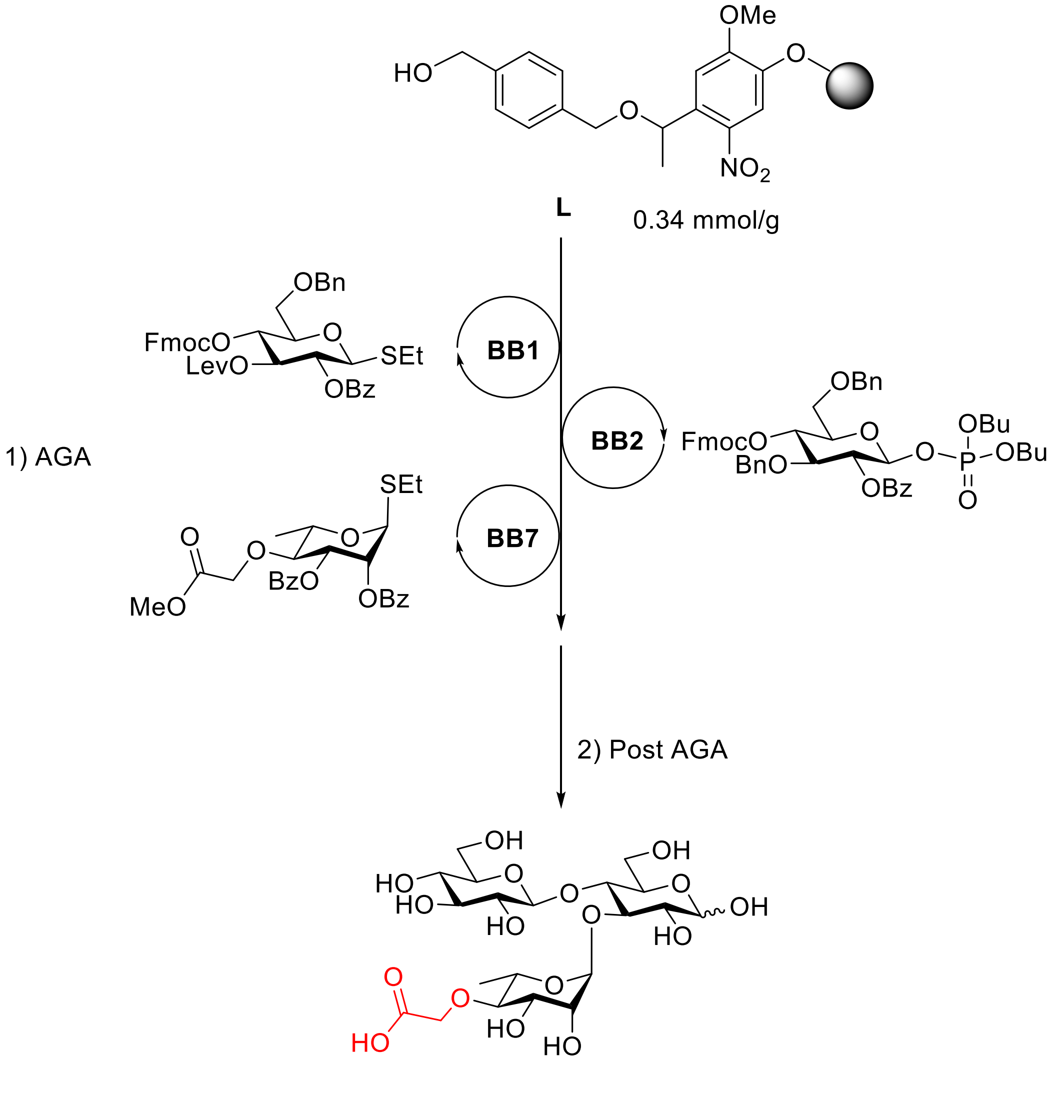

3mer

Step	Sequence	Modules	Notes
AGA		**A**	**L** swelling
	**BB1**	**B**, **C1**, **D**, **E1**	**C1**: (-20°C for 20 min, 0°C for 40 min)
	**BB2**	**B**, **C2**, **D**, **E1, D, E2**	**C2**: (-30°C for 30 min, -10°C for 40 min)
	**BB7**	**B**, **C1**, **D**	**C1**: (-30°C for 30 min, -10°C for 40 min)
Post-AGA		**I1**, **L** (Method A)**,** **J1**, **K2**, **L** (Method C2)	**L** (Method B2, t_R = 32.7 min, 34.3 min)

Automated synthesis (was conducted in 0.019 mmol scale), global deprotection, and purification afforded **3mer** as a white solid (3.2 mg, 31% overall yield, α : β = 45 : 55).

^{1}H NMR (600 MHz, D_2O) δ 5.27 (d, *J* = 1.7 Hz, 0.5H, H-1β, Rha), 5.22 (d, *J* = 1.8 Hz, 0.5H, H-1α Rha), 5.20 (d, *J* = 3.8 Hz, 0.5H, H-1α Glc), 4.66 (d, *J* = 8.0 Hz, 0.5H, H-1β Glc), 4.51 – 4.42 (m, 2H, H-5 Rha, H-1 Glc'), 4.23 (dd, *J* = 16.6, 1.0 Hz, 1H), 4.09 (dd, *J* = 3.4, 1.7 Hz, 1H), 4.01 – 3.92 (m, 4.5H), 3.90 – 3.87 (m, 1H), 3.86 – 3.76 (m, 3H), 3.69 (dd, *J* = 9.9, 3.8 Hz, 0.5H), 3.58 (m, 0.5H), 3.48 (td, *J* = 9.3, 1.4 Hz, 1H), 3.44 – 3.38 (m, 1.5H), 3.32 (dd, *J* = 9.8, 9.1 Hz, 1H), 3.29 – 3.24 (m, 2H), 1.31 (dd, *J* = 6.4, 2.8 Hz, 3H). ^{13}C NMR (151 MHz, D_2O) δ 179.28, 101.35 (C-1, Glc'), 100.33, 100.20 (C-1, Rha), 95.78 (C-1β, Glc), 92.05 (C-1α, Glc), 82.64, 78.14, 76.38, 75.79, 75.47, 75.34, 75.29, 73.64, 72.70, 72.44, 70.92, 69.97, 69.62, 69.40, 67.78, 67.72, 61.48, 59.55, 16.45. (ESI-HRMS) m/z 569.1683 $[M+Na]^+$ ($C_{20}H_{34}NaO_{17}$ requires 569.1694).

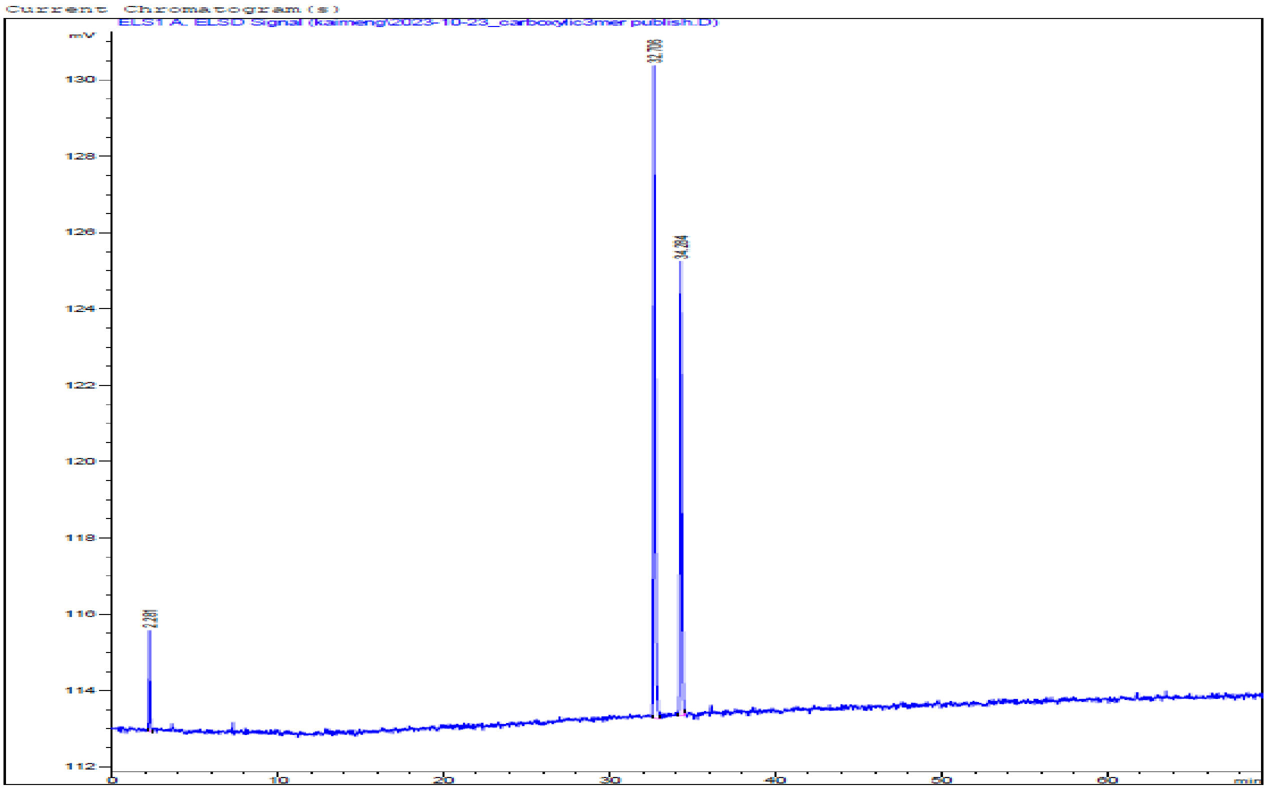

RP-HPLC of **3mer** (ELSD trace, Method B2 t_R = 32.7 min, 34.3 min)

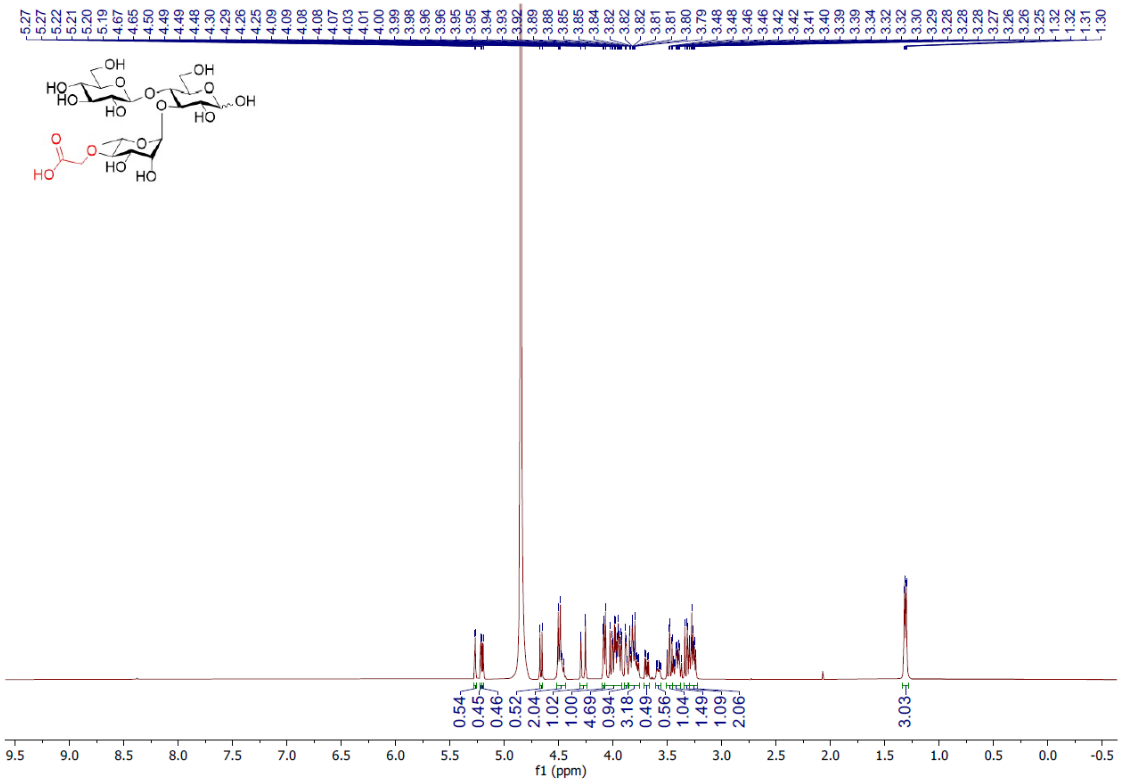

^{1}H NMR of 3mer (600 MHz, D_2O)

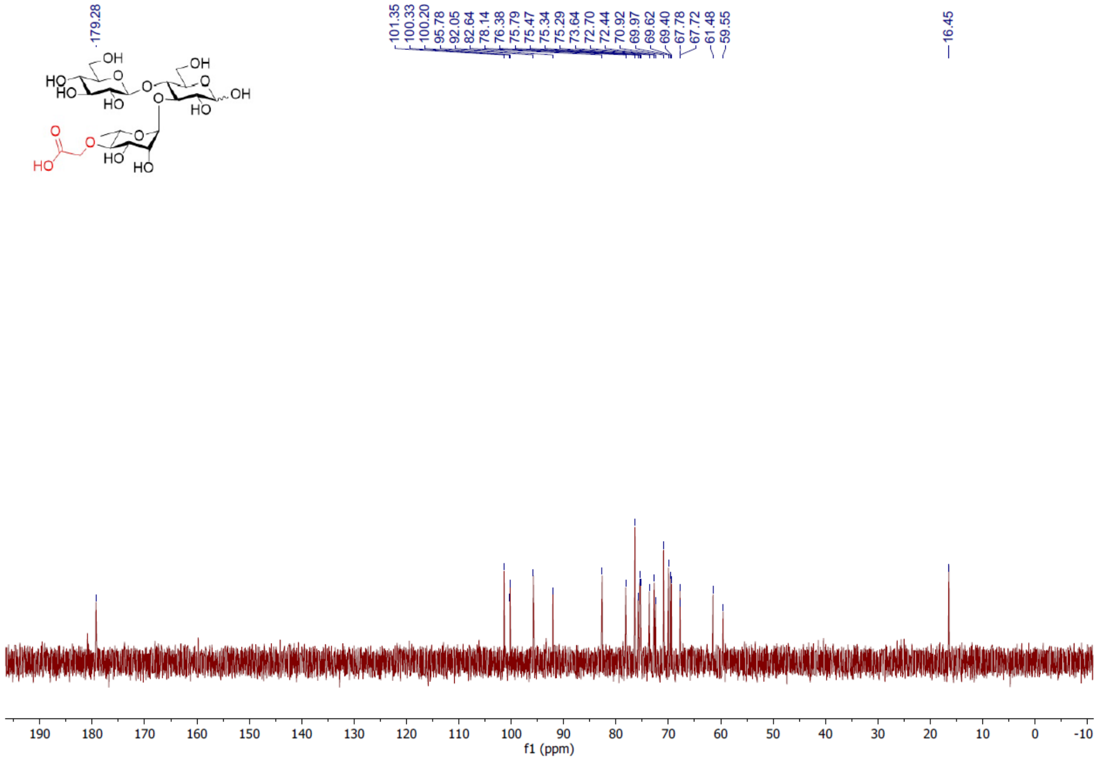

^{13}C NMR of 3mer (151 MHz, D_2O)

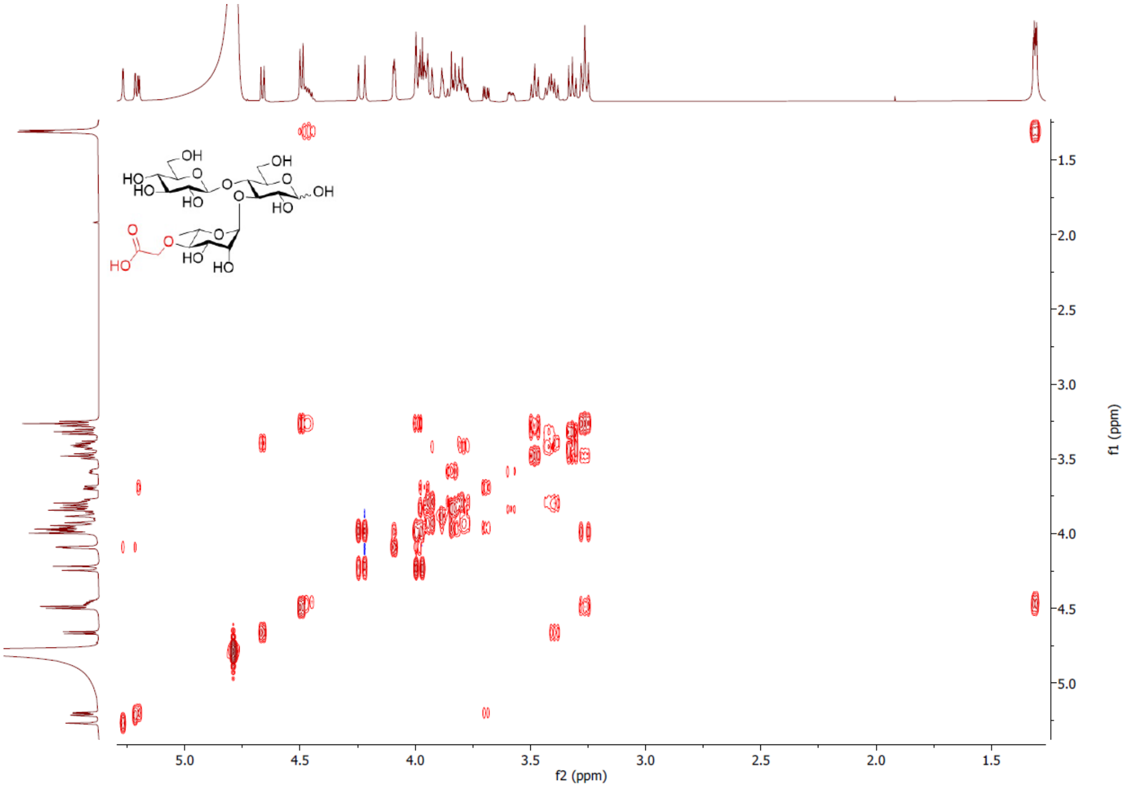

COSY NMR of 3mer (D_2O)

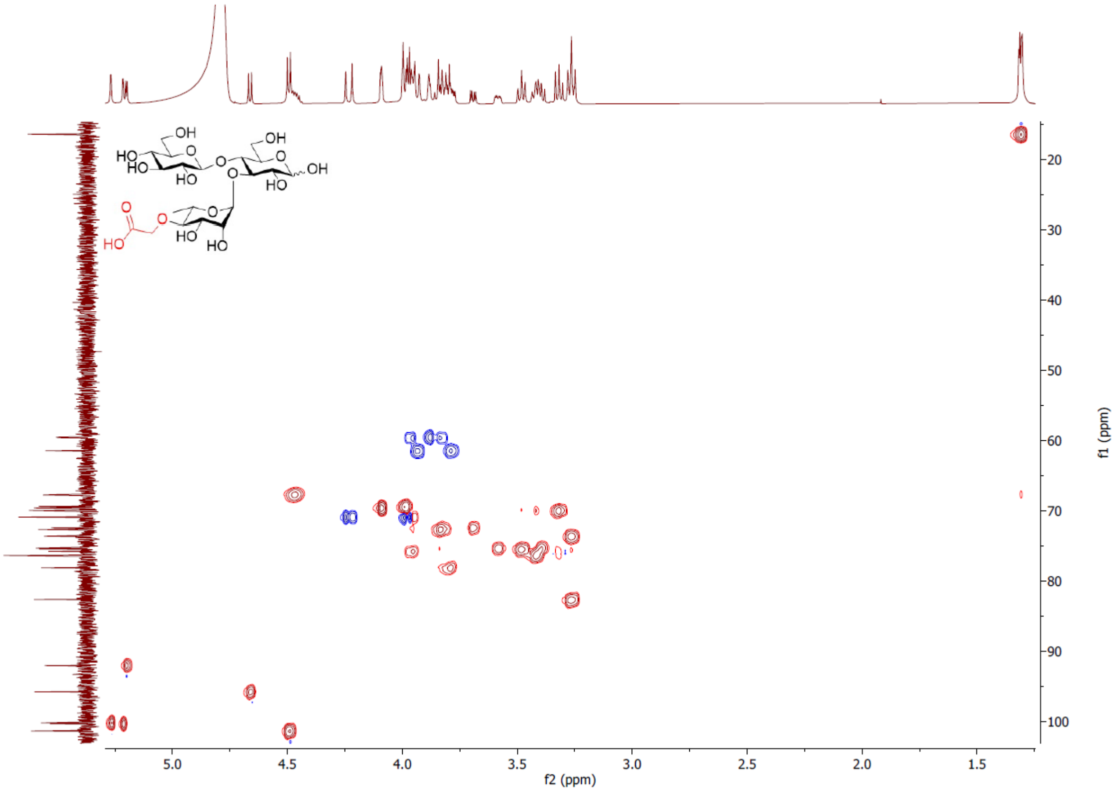

HSQC NMR of 3mer (D_2O)

3.5.2 4mer-I

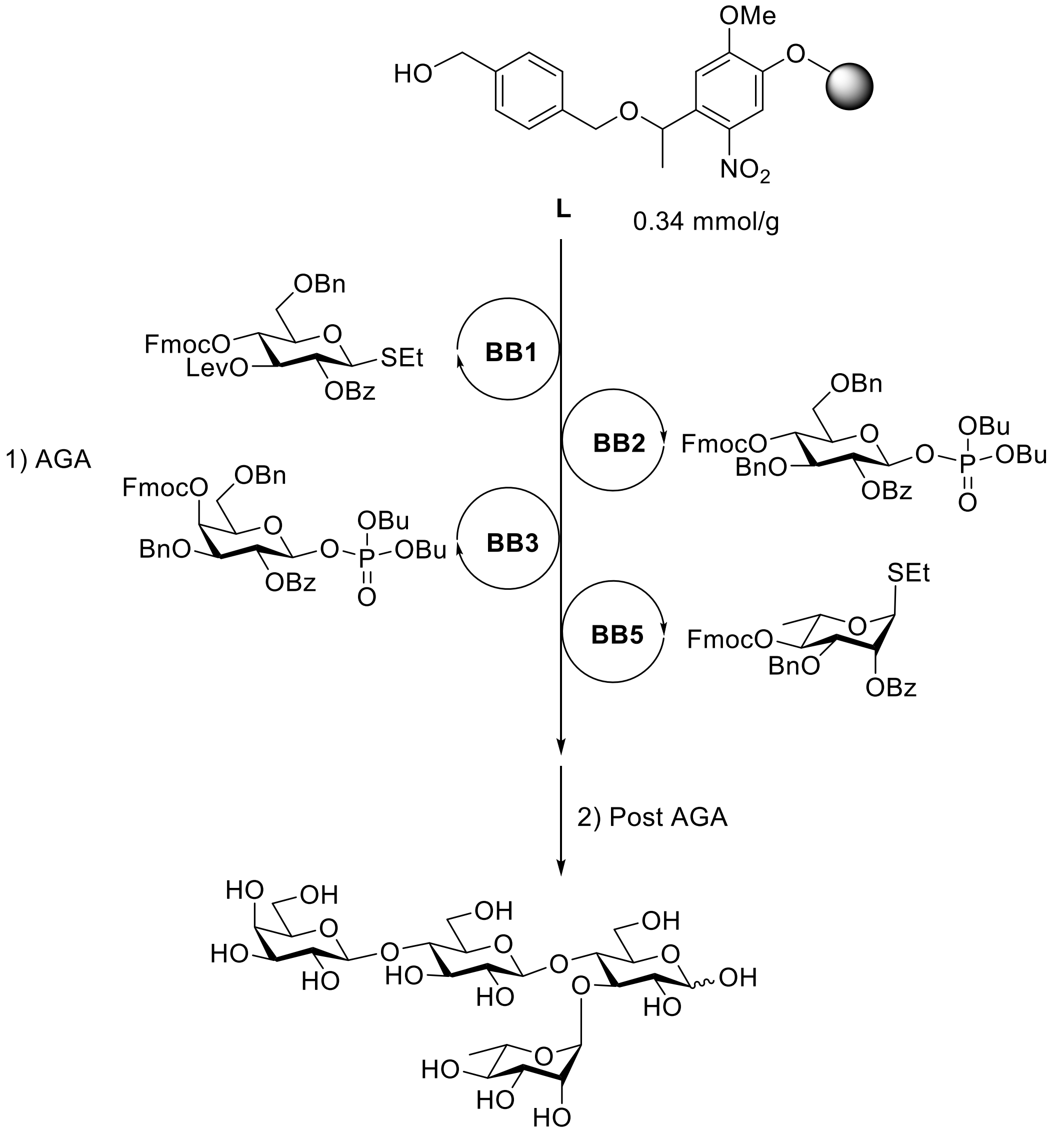

4mer-I

Step	Sequence	Modules	Notes
AGA		**A**	**L** swelling
	**BB1**	**B**, **C1**, **D**, **E1**	**C1**: (-20°C for 20 min, 0°C for 40 min)
	**BB2**	**B**, **C2**, **D**, **E1**	**C2**: (-30°C for 30 min, -10°C for 40 min)
	**BB3**	**B**, **C2**, **D**, **E1, D, E2**	**C2**: (-30°C for 30 min, -10°C for 40 min)
	**BB5**	**B**, **C1**, **D**, **E1**	**C1**: (-20°C for 20 min, 0°C for 40 min)

Post-AGA	**I1**, **L** (Method A), **J1**, **K2**, **L** (Method C1)	**L** (Method B1, t_R = 20.4 min, 20.8 min)

Automated synthesis (was conducted in 0.019 mmol scale), global deprotection, and purification afforded **4mer-I** as a white solid (3.6 mg, 44% overall yield, α : β = 47 : 53).

^{1}H NMR (700 MHz, D_2O) δ 5.22 (s, 0.5H, H-1β, Rha), 5.17 (s, 0.5H, H-1α Rha), 5.15 (d, *J* = 3.8 Hz, 0.5H, H-1α Glc), 4.62 (d, *J* = 7.9 Hz, 0.5H, H-1β Glc), 4.48 (d, *J* = 7.9 Hz, 1H, H-1 Glc'), 4.39 (m, 2H, H-5 Rha, H-1 Gal), 4.01 – 3.95 (m, 2H), 3.91 (m, 1.5H), 3.88 (d, *J* = 3.4 Hz, 1H), 3.85 (d, *J* = 3.7 Hz, 1H), 3.80 (m, 3H), 3.77 – 3.68 (m, 3H), 3.66 (m, 1H), 3.65 – 3.57 (m, 3.5H), 3.51 (m, 2.5H), 3.40 (t, *J* = 9.7 Hz, 1H), 3.34 (t, *J* = 8.5 Hz, 0.5H), 3.25 (m, 1H), 1.21 (m, 3H). ^{13}C NMR (176 MHz, D_2O) δ 102.87 (C-1, Gal), 101.42 (C-1, Glc'), 101.38, 100.68, 100.55 (C-1, Rha), 95.69 (C-1β, Glc), 91.96 (C-1α, Glc), 78.38, 78.22, 75.87, 75.25, 75.22, 75.20, 75.00, 74.07, 74.05, 73.24, 73.22, 73.05, 73.01, 72.42, 72.37, 71.86, 70.86, 70.81, 70.02, 69.92, 69.88, 68.44, 68.41, 68.34, 60.90, 60.32, 59.46, 59.38, 16.42, 16.40. (ESI-HRMS) m/z 673.2161 $[M+Na]^+$ ($C_{24}H_{42}NaO_{20}$ requires 673.2167).

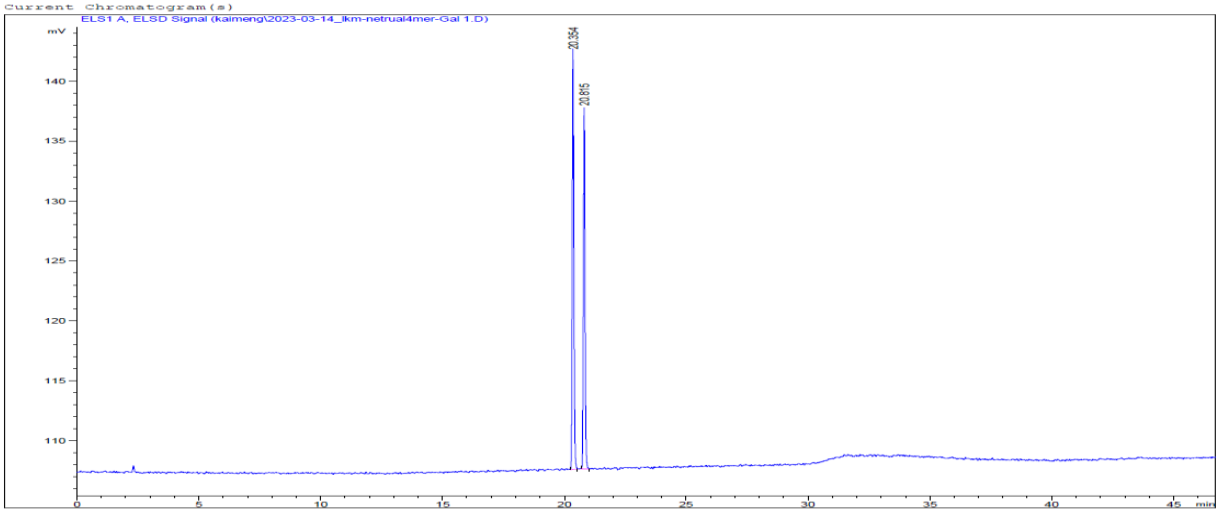

RP-HPLC of **4mer-I** (ELSD trace, Method B1 t_R = 20.4 min, 20.8 min)

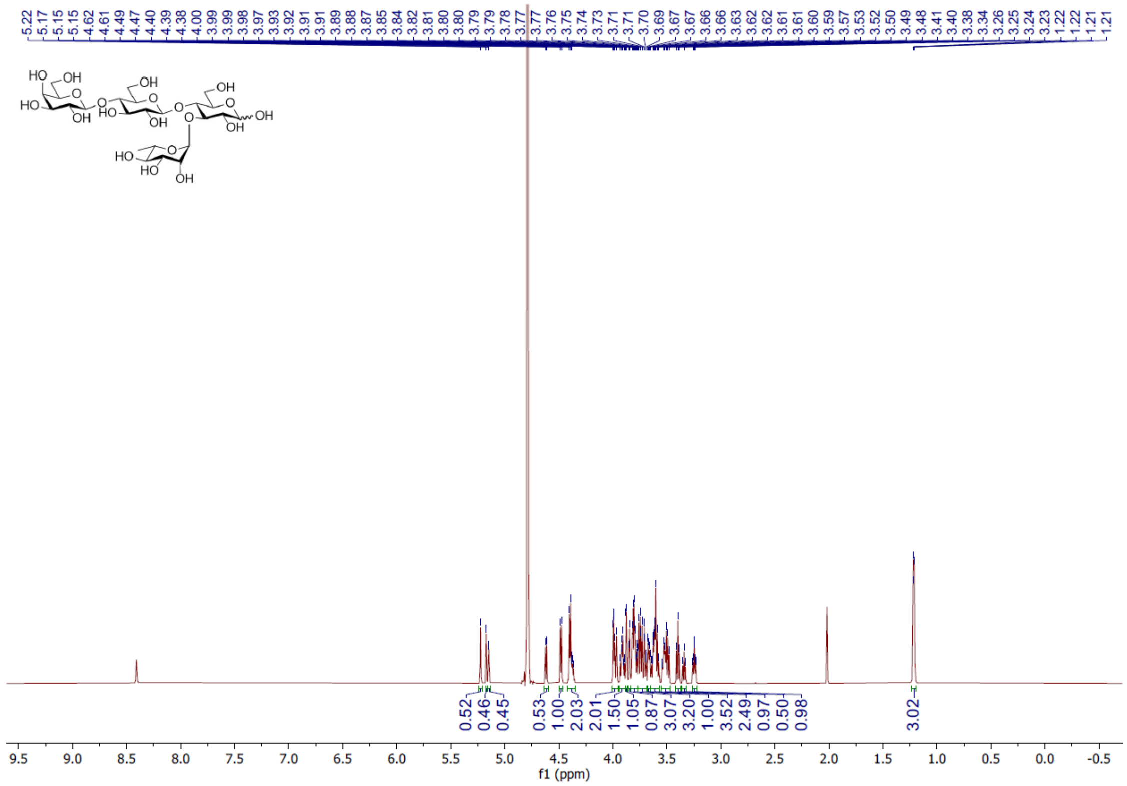

^{1}H NMR of 4mer-I (700 MHz, D_2O)

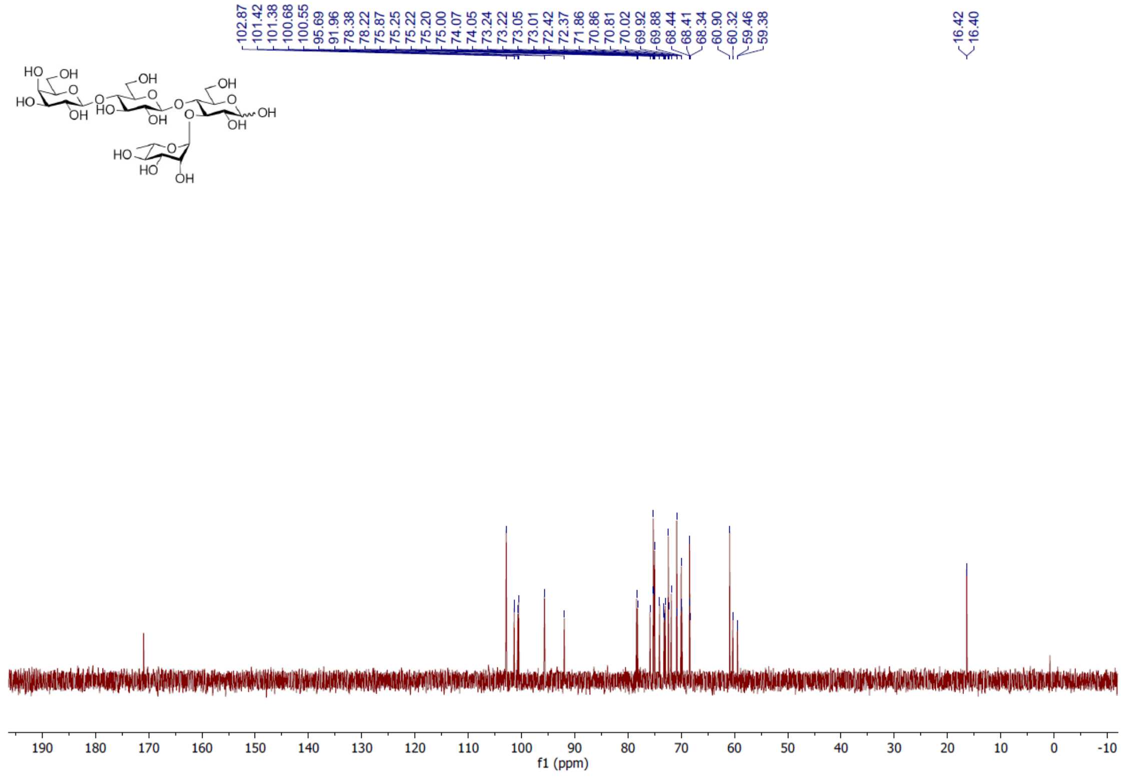

^{13}C NMR of 4mer-I (176 MHz, D_2O)

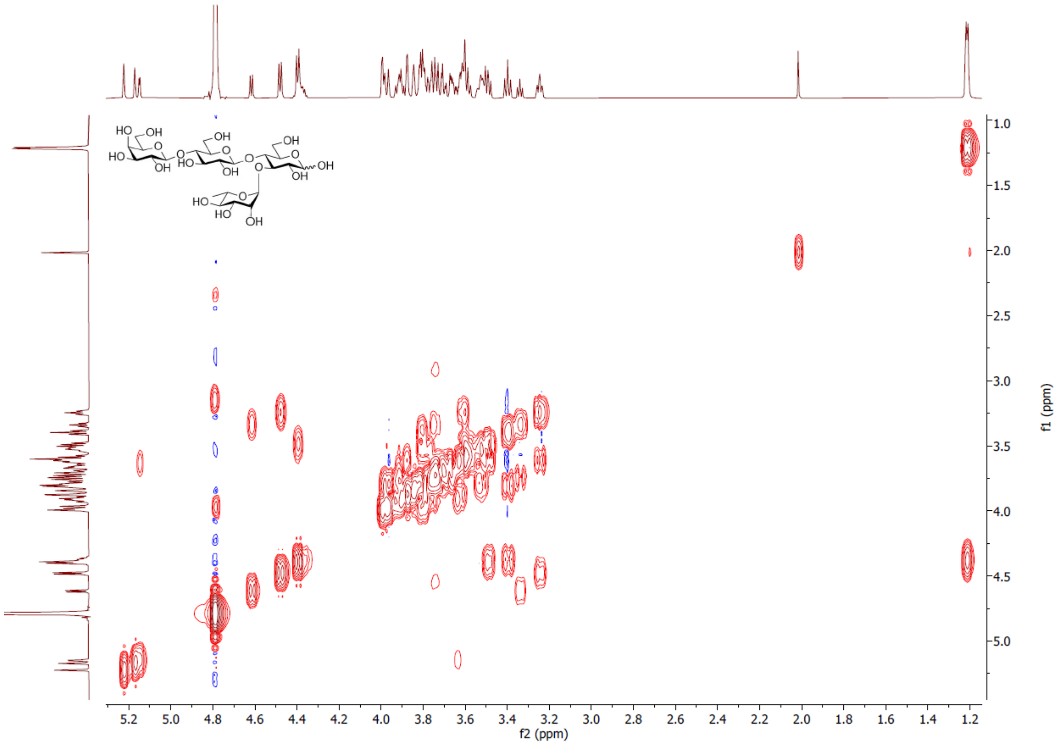

COSY NMR of 4mer-I (D$_2$O)

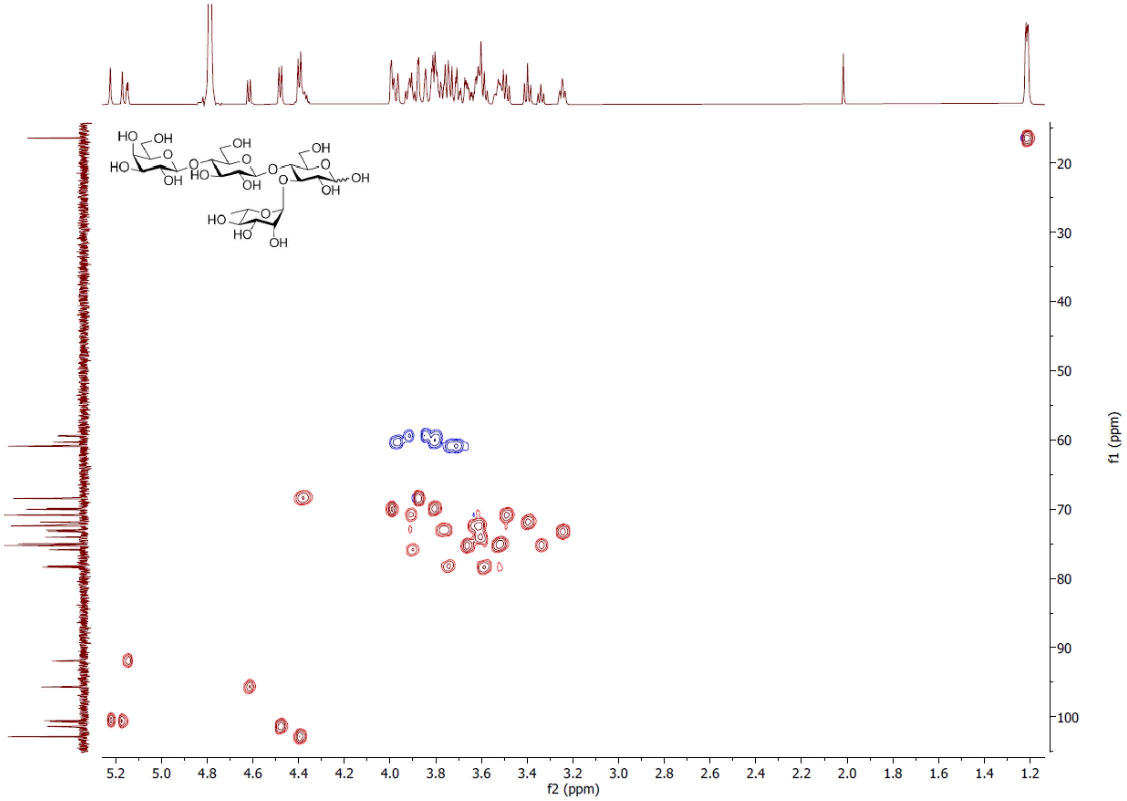

HSQC NMR of 4mer-I (D$_2$O)

3.5.3 4mer-II

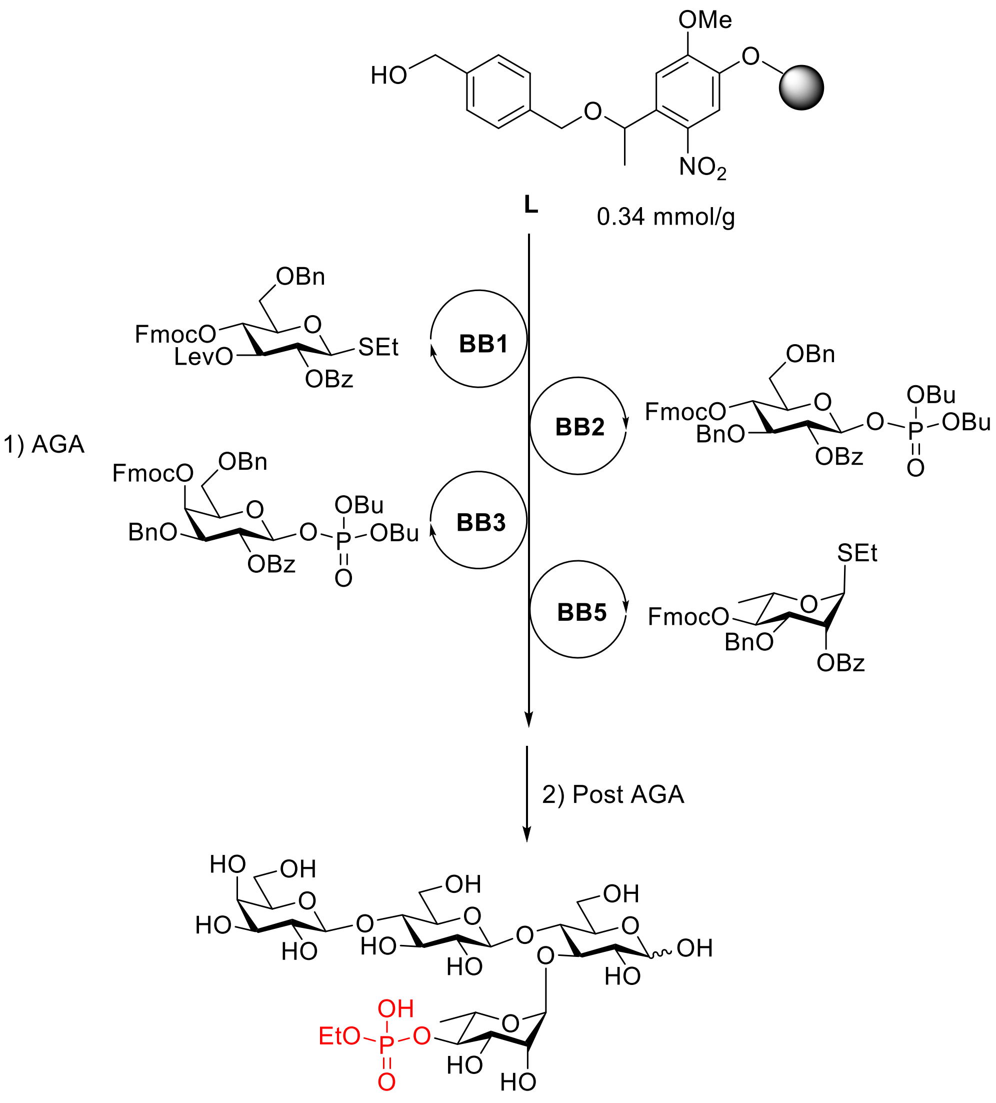

4mer-II

Step	Sequence	Modules	Notes
AGA		**A**	**L** swelling
	**BB1**	**B**, **C1**, **D**, **E1**	**C1**: (-20°C for 20 min, 0°C for 40 min)
	**BB2**	**B**, **C2**, **D**, **E1**	**C2**: (-30°C for 30 min, -10°C for 40 min)
	**BB3**	**B**, **C2**, **D**, **E1, D, E2**	**C2**: (-30°C for 30 min, -10°C for 40 min)
	**BB5**	**B**, **C1**, **D**, **E1**	**C1**: (-20°C for 20 min, 0°C for 40 min)
Post-AGA		**F, G, I1**, **L** (Method A)**, J2**, **K2**, **L** (Method C1)	**L** (Method B1, t_R = 26.8 min, 26.9 min)

Automated synthesis (was conducted in 0.019 mmol scale), global deprotection, and purification afforded **4mer-II** as a white solid (5.8 mg, 40% overall yield, α : β = 45 : 55).

^{1}H NMR (700 MHz, D_2O) δ 5.27 (s, 0.5H, H-1β, Rha), 5.21 (s, 0.5H, H-1α Rha), 5.15 (d, *J* = 3.8 Hz, 0.5H, H-1α Glc), 4.62 (d, *J* = 8.0 Hz, 0.5H, H-1β Glc), 4.47 (dd, *J* = 12.7, 7.1 Hz, 2H, H-5 Rha, H-1 Glc'), 4.42 (d, *J* = 7.9 Hz, 1H, H-1 Gal), 4.09 – 4.04 (m, 1H), 4.02 – 3.98 (m, 2H), 3.98 – 3.90 (m, 4.5H), 3.89 (d, *J* = 3.4 Hz, 1H), 3.83 (d, *J* = 3.9 Hz, 1H), 3.80 – 3.69 (m, 6H), 3.64 (m, 1.5H), 3.59 (t, *J* = 9.2 Hz, 1H), 3.55 (t, *J* = 9.1 Hz, 1.5H), 3.48 (dd, *J* = 10.0, 7.9 Hz, 1H), 3.40 (t, *J* = 9.3 Hz, 1H), 3.35 (t, *J* = 8.4 Hz, 0.5H), 3.25 (m, 1H), 1.27 (m, 3H), 1.24 (t, *J* = 7.1 Hz, 3H). ^{13}C NMR (176 MHz, D_2O) δ 103.34 (C-1, Gal), 101.11(C-1, Glc'), 100.02, 99.92 (C-1, Rha), 95.67 (C-1β, Glc), 92.00 (C-1α, Glc), 80.67, 80.63, 77.51, 75.34, 75.21, 75.15, 74.12, 73.20, 72.75, 72.50, 72.44, 70.89, 70.82, 69.92, 69.68, 68.38, 62.60, 62.57, 61.35, 60.73, 59.49, 16.52, 15.77, 15.73. ^{31}P NMR (162 MHz, D_2O) δ 0.57. (ESI-HRMS) m/z 759.2347 $[M+H]^+$ ($C_{26}H_{48}O_{23}P$ requires 759.2324).

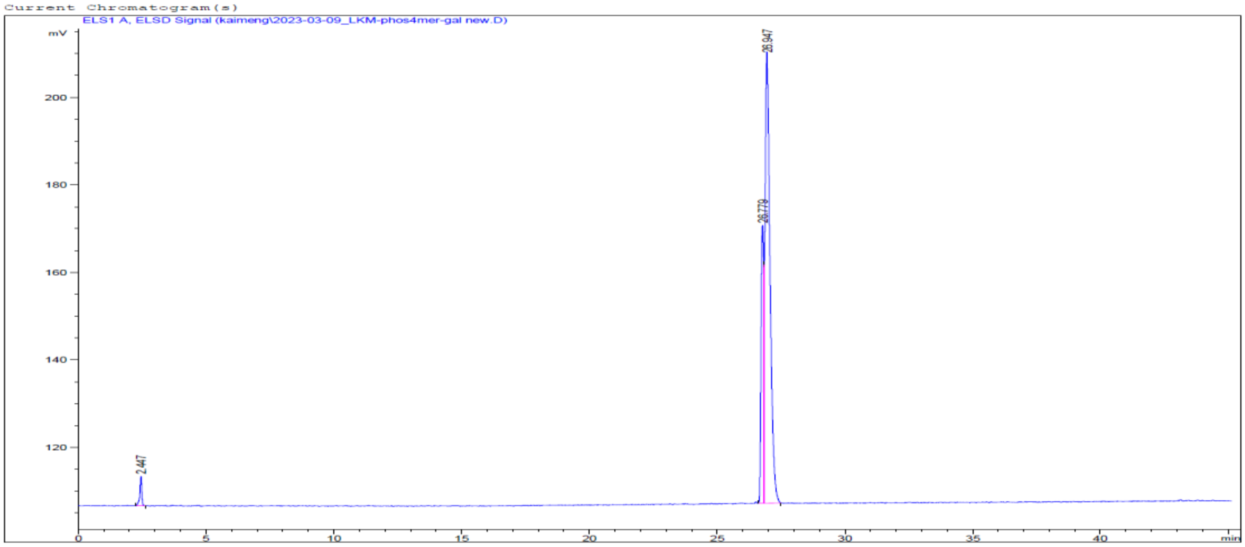

RP-HPLC of **4mer-II** (ELSD trace, Method B1 t_R = 26.8 min, 26.9 min).

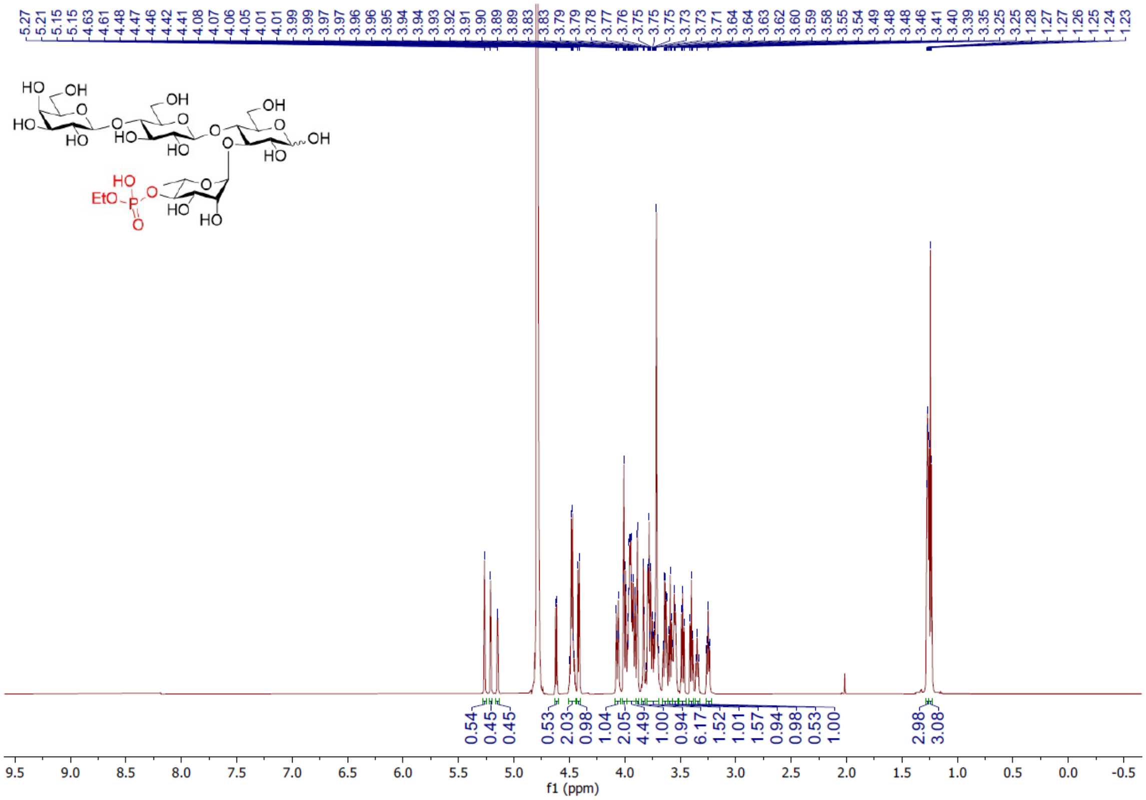

^{1}H NMR of 4mer-II (700 MHz, D_2O)

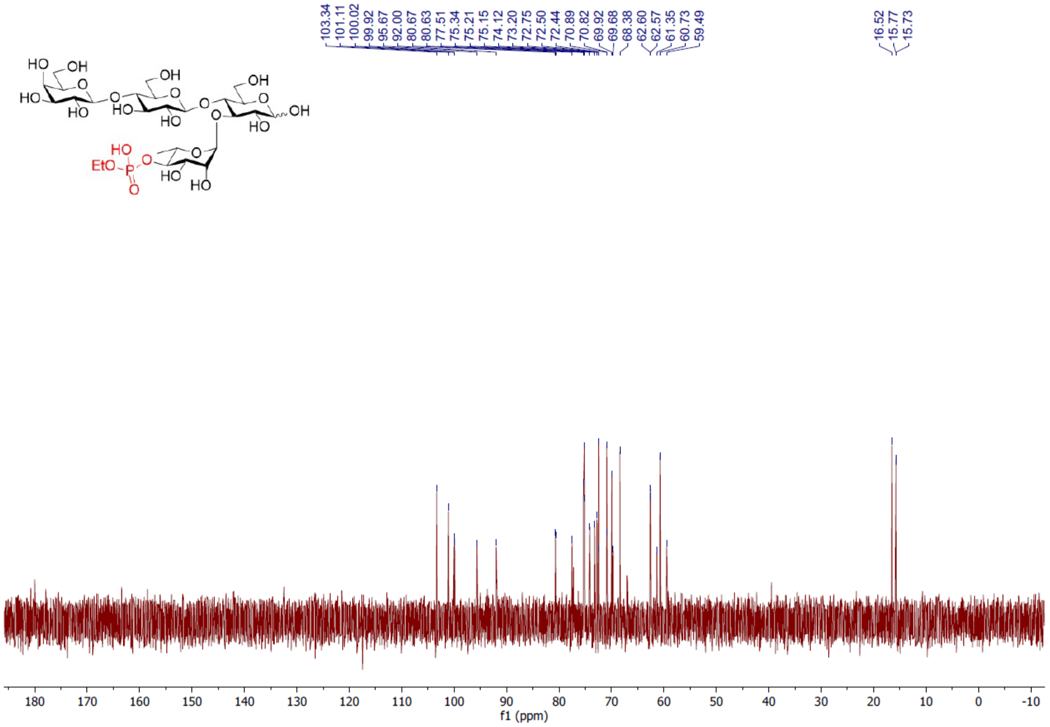

^{13}C NMR of 4mer-II (176 MHz, D_2O)

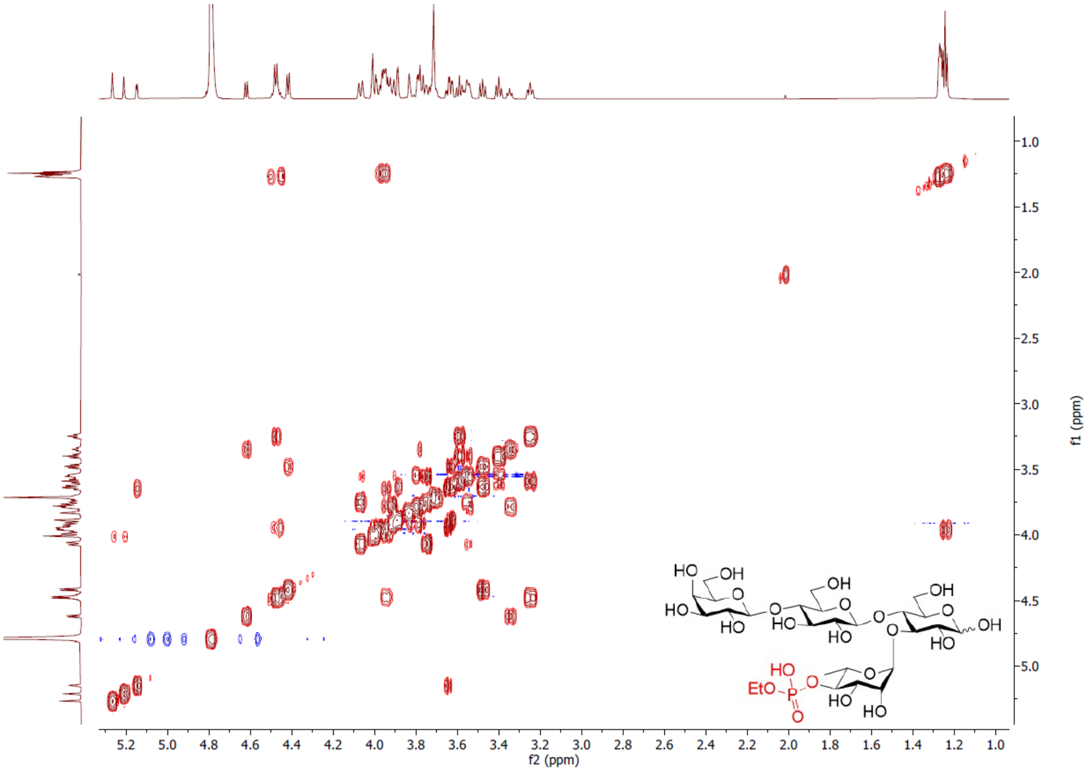

COSY NMR of 4mer-II (D_2O)

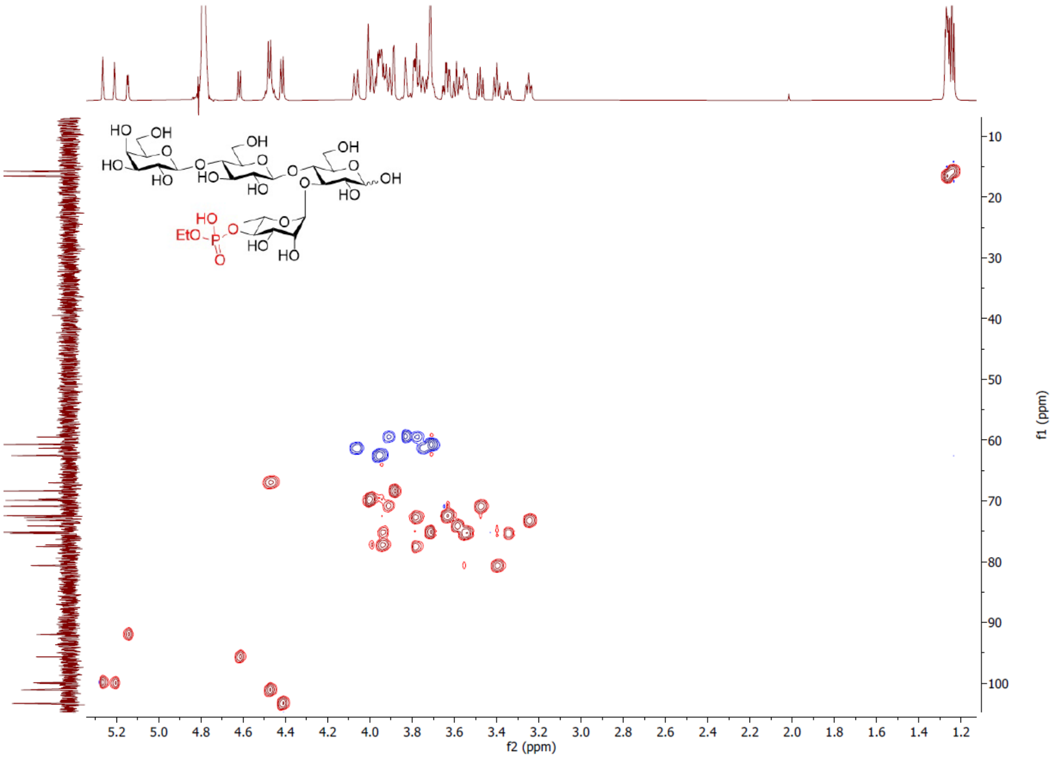

HSQC NMR of 4mer-II (D_2O)

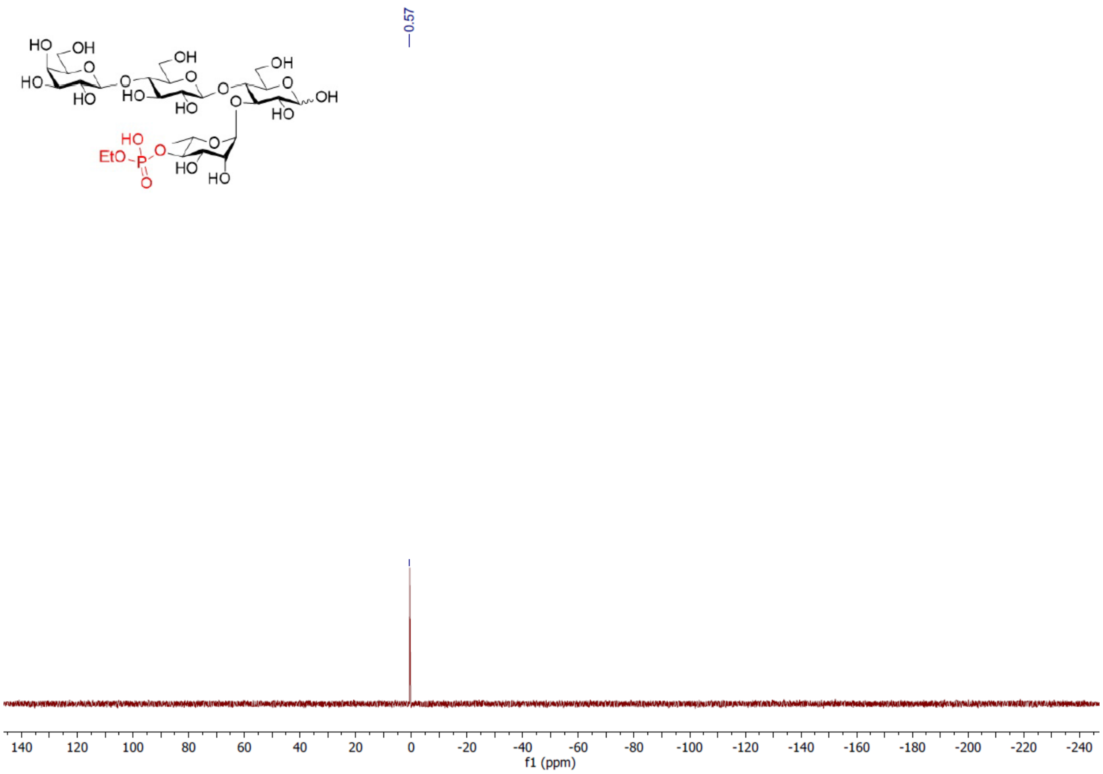

^{31}P NMR of **4mer-II** (162 MHz, D_2O)

3.5.4 4mer-III

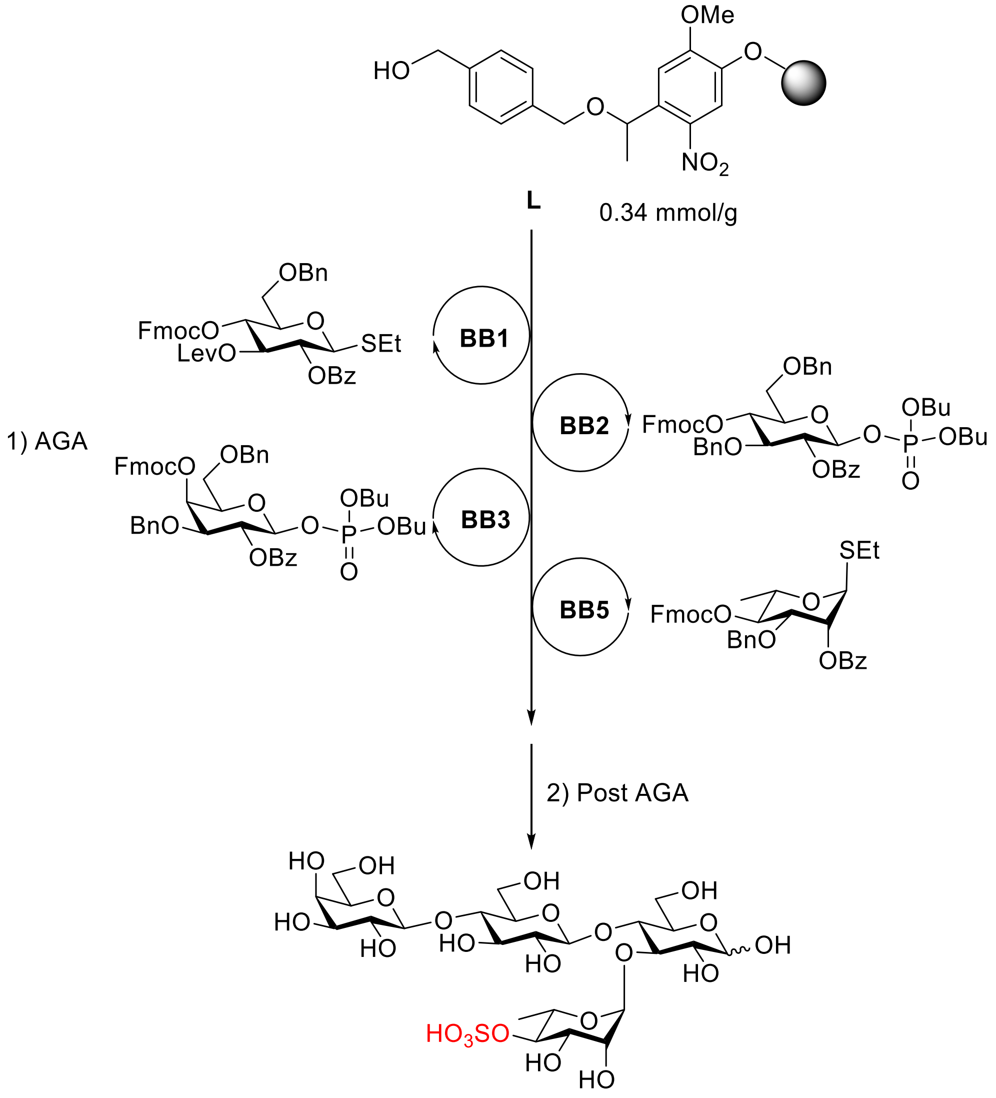

4mer-III

Step	Sequence	Modules	Notes
AGA		**A**	**L** swelling
	**BB1**	**B**, **C1**, **D**, **E1**	**C1**: (-20°C for 20 min, 0°C for 40 min)
	**BB2**	**B**, **C2**, **D**, **E1**	**C2**: (-30°C for 30 min, -10°C for 40 min)
	**BB3**	**B**, **C2**, **D**, **E1, D, E2**	**C2**: (-30°C for 30 min, -10°C for 40 min)
	**BB5**	**B**, **C1**, **D**, **E1**	**C1**: (-20°C for 20 min, 0°C for 40 min)
Post-AGA		**H, I1**, **L** (Method A)**, J1**, **K2**, **L** (Method C1)	**L** (Method B1, t_R = 25.5 min, 25.7 min)

Automated synthesis (was conducted in 0.019 mmol scale), global deprotection, and purification afforded **4mer-III** as a white solid (3.9 mg, 28% overall yield, α : β = 47 : 53).

^{1}H NMR (600 MHz, D_2O) δ 5.32 (d, *J* = 1.8 Hz, 0.5H, H-1β, Rha), 5.26 (d, *J* = 1.8 Hz, 0.5H, H-1α Rha), 5.19 (d, *J* = 3.8 Hz, 0.5H, H-1α Glc), 4.66 (d, *J* = 8.0 Hz, 0.5H, H-1β Glc), 4.62 – 4.56 (m, 1H), 4.52 (d, J = 7.9 Hz, 1H, H-1 Glc'), 4.43 (d, J = 8.0 Hz, 1H, H-1 Gal), 4.23 (t, J = 9.5 Hz, 1H), 4.15 – 4.01 (m, 3H), 3.98 – 3.92 (m, 2.5H), 3.87 – 3.74 (m, 7H), 3.70 – 3.59 (m, 4H), 3.51 (m, 2H), 3.41 – 3.37 (m, 0.5H), 3.28 (m, 1H), 1.32 (dd, J = 6.4, 3.0 Hz, 3H). ^{13}C NMR (151 MHz, D_2O) δ 103.38 (C-1, Gal), 101.16 (C-1, Glc'), 99.79, 99.70 (C-1, Rha), 95.70 (C-1β, Glc), 92.06 (C-1α, Glc), 80.75, 80.49, 77.38, 75.46, 75.39, 75.25, 75.10, 74.95, 74.06, 73.49, 72.77, 72.62, 72.52, 70.91, 69.94, 69.01, 68.96, 68.39, 66.07, 61.17, 60.62, 59.56, 59.47, 16.50. (ESI-HRMS) m/z 753.1750 $[M+Na]^+$ ($C_{24}H_{42}NaO_{23}S$ requires 753.1735).

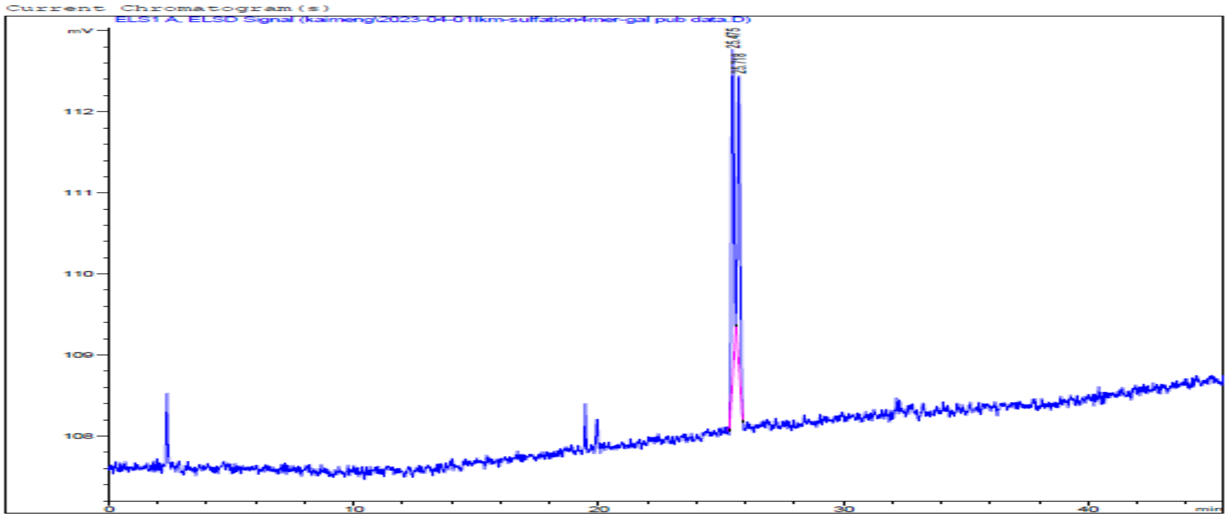

RP-HPLC of **4mer-III** (ELSD trace, Method B1 t_R = 25.5 min, 25.7 min).

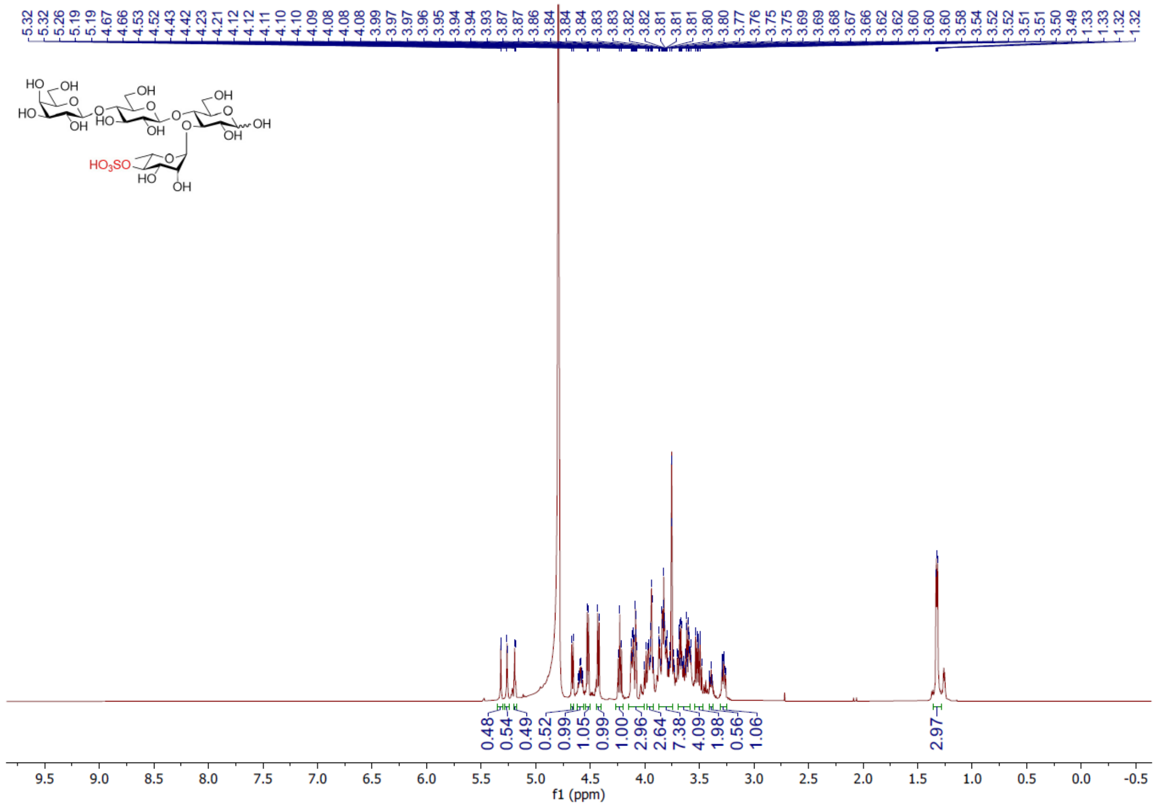

^{1}H NMR of 4mer-III (600 MHz, D_2O)

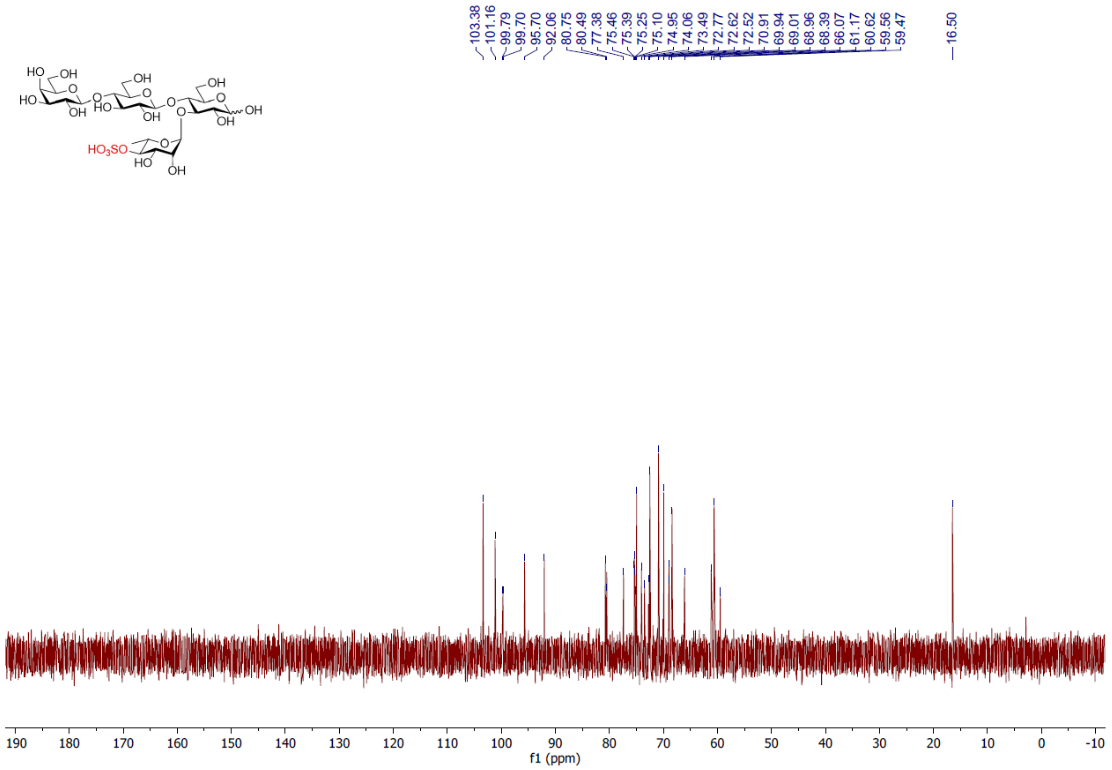

^{13}C NMR of 4mer-III (151 MHz, D_2O)

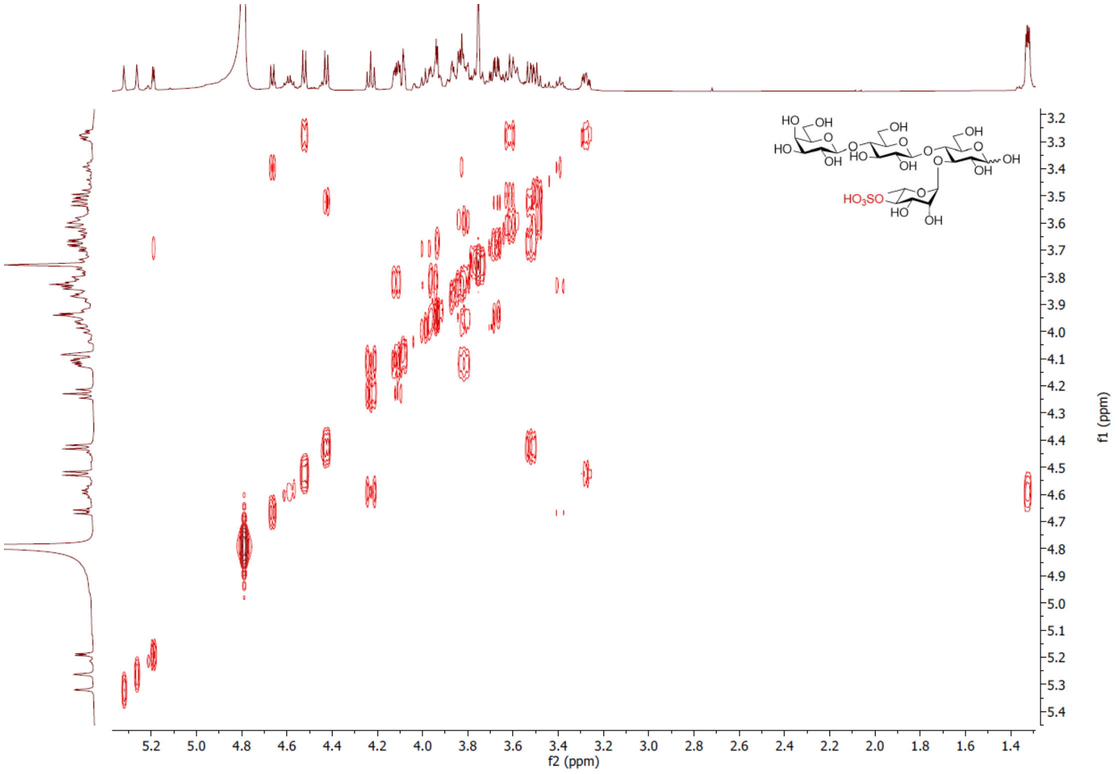

COSY NMR of 4mer-III (D_2O)

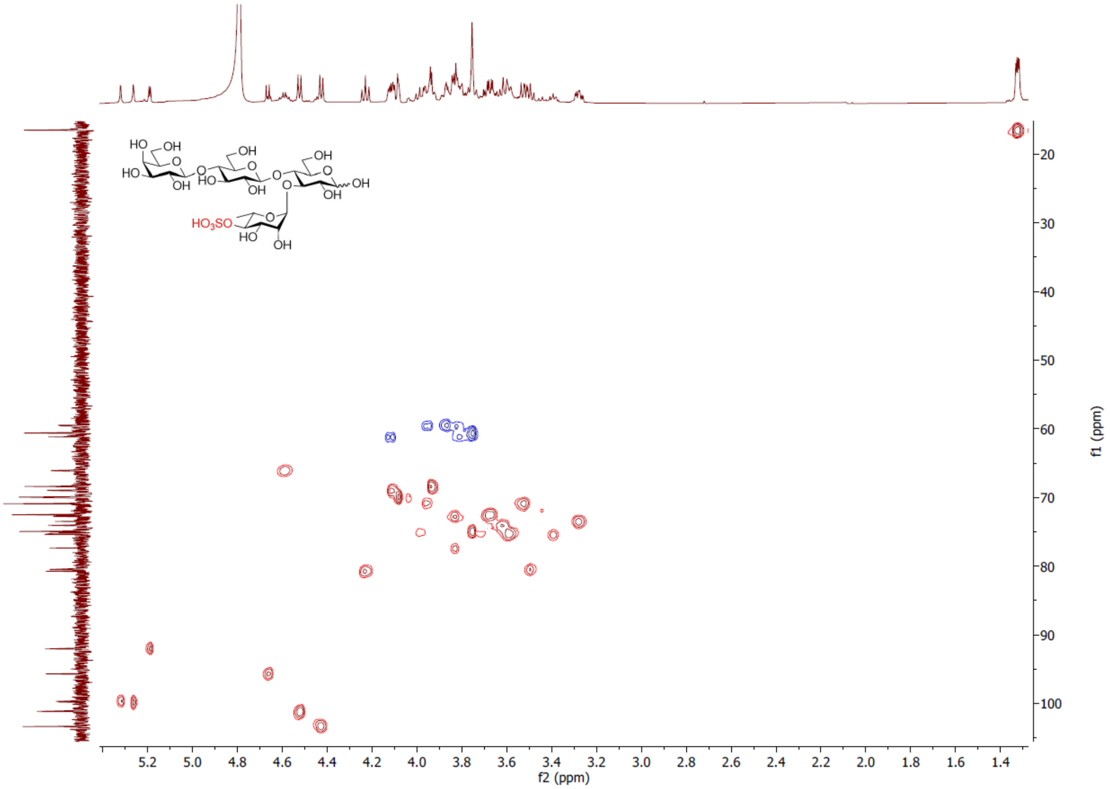

HSQC NMR of 4mer-III (D_2O)

3.5.5 4mer-IV

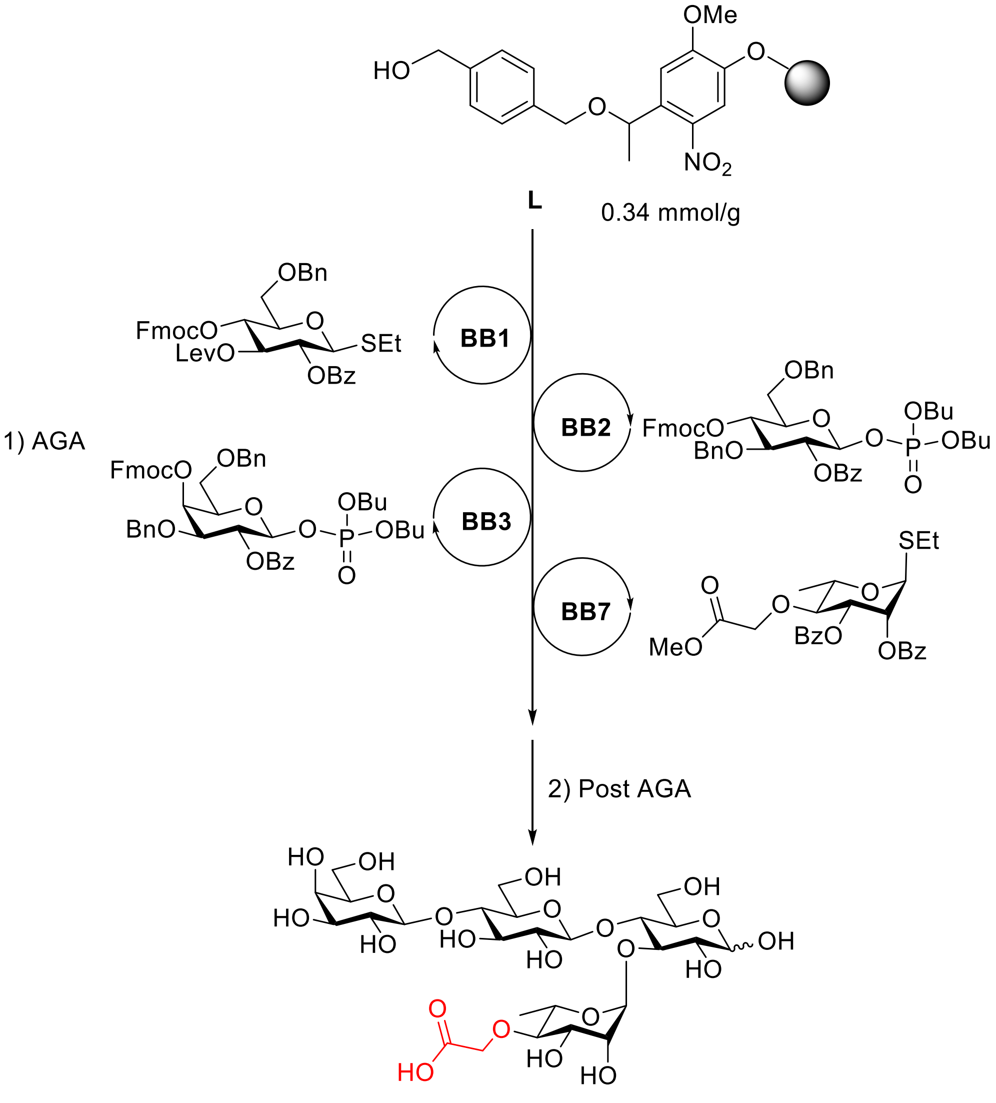

4mer-IV

Step	Sequence	Modules	Notes
AGA		**A**	**L** swelling
	**BB1**	**B**, **C1**, **D**, **E1**	**C1**: (-20°C for 20 min, 0°C for 40 min)
	**BB2**	**B**, **C2**, **D**, **E1**	**C2**: (-30°C for 30 min, -10°C for 40 min)
	**BB3**	**B**, **C2**, **D**, **E1, D, E2**	**C2**: (-30°C for 30 min, -10°C for 40 min)
	**BB7**	**B**, **C1**, **D**	**C1**: (-20°C for 20 min, 0°C for 40 min)

Post-AGA	**I1**, **L** (Method A), **J1**, **K2**, **L** (Method C2)	**L** (Method B2, t_R = 36.8 min)

Automated synthesis (was conducted in 0.019 mmol scale), global deprotection, and purification afforded **4mer-IV** as a white solid (3.8 mg, 28% overall yield, α : β = 47 : 53).

^{1}H NMR (700 MHz, D_2O) δ 5.23 (d, *J* = 1.7 Hz, 0.5H, H-1β, Rha), 5.18 (d, *J* = 1.8 Hz, 0.5H, H-1α Rha), 5.14 (d, *J* = 3.7 Hz, 0.5H, H-1α Glc), 4.60 (d, *J* = 8.0 Hz, 0.5H, H-1β Glc), 4.48 – 4.39 (m, 2H, H-1 Glc', H-5 Rha), 4.30 – 4.23 (m, 2H, H-1 Gal, CH_2COOH), 4.05 (dd, *J* = 16.9, 2.0 Hz, 1H), 4.03 – 3.96 (m, 3H), 3.94 – 3.89 (m, 1.5H), 3.86 (d, *J* = 3.4 Hz, 1H), 3.83 – 3.74 (m, 4H), 3.72 – 3.67 (m, 2H), 3.62 (m, 2.5H), 3.57 (m, 1H), 3.54 – 3.45 (m, 3.5H), 3.33 (dd, *J* = 9.2, 7.9 Hz, 0.5H), 3.27 – 3.20 (m, 2H), 1.26 (dd, *J* = 6.4, 3.6 Hz, 3H). ^{13}C NMR (176 MHz, D_2O) δ 177.61, 102.96 (C-1, Gal), 101.22, 101.20 (C-1, Glc'), 100.06, 99.94 (C-1, Rha), 95.66 (C-1β, Glc), 91.98 (C-1α, Glc), 83.10, 83.08, 78.72, 77.46, 75.34, 75.30, 75.20, 75.14, 73.95, 73.93, 73.37, 73.34, 72.69, 72.45, 72.33, 70.87, 70.79, 70.49, 69.57, 69.40, 69.35, 68.46, 67.45, 67.39, 60.94, 60.74, 59.49, 59.40, 16.35. (ESI-HRMS) m/z 731.2238 $[M+Na]^+$ ($C_{26}H_{44}NaO_{22}$ requires 731.2222).

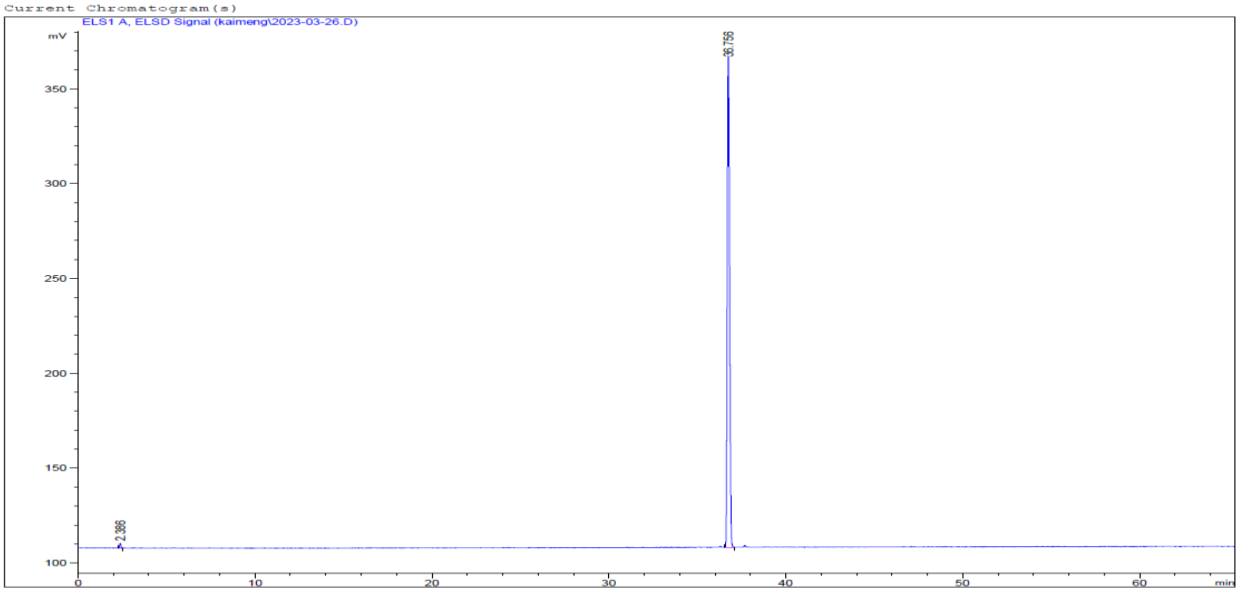

RP-HPLC of **4mer-IV** (ELSD trace, Method B2 t_R = 36.8 min).

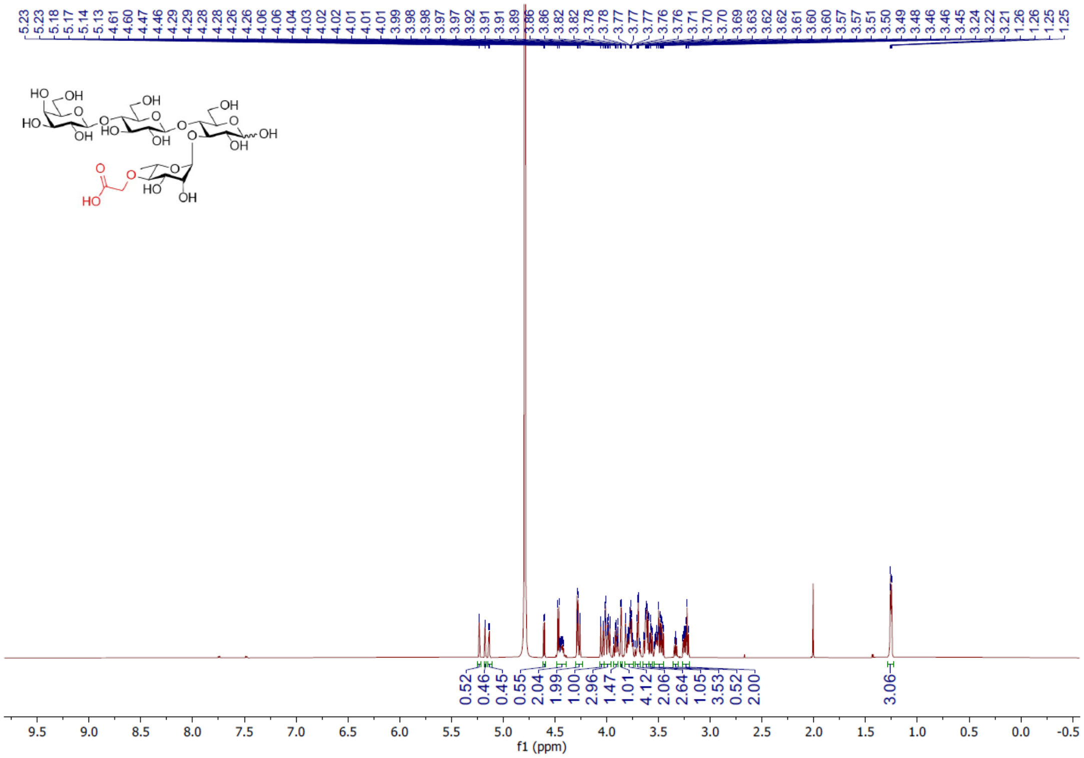

^{1}H NMR of 4mer-VI (700 MHz, D_2O)

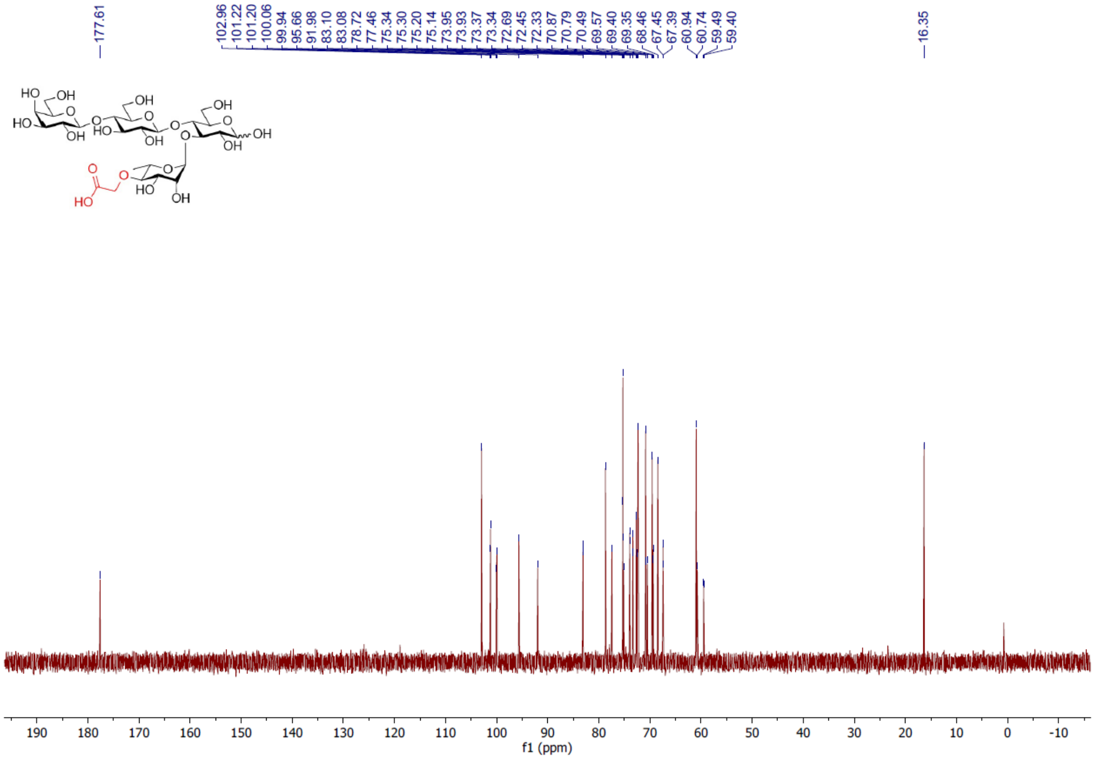

^{13}C NMR of 4mer-VI (176 MHz, D_2O)

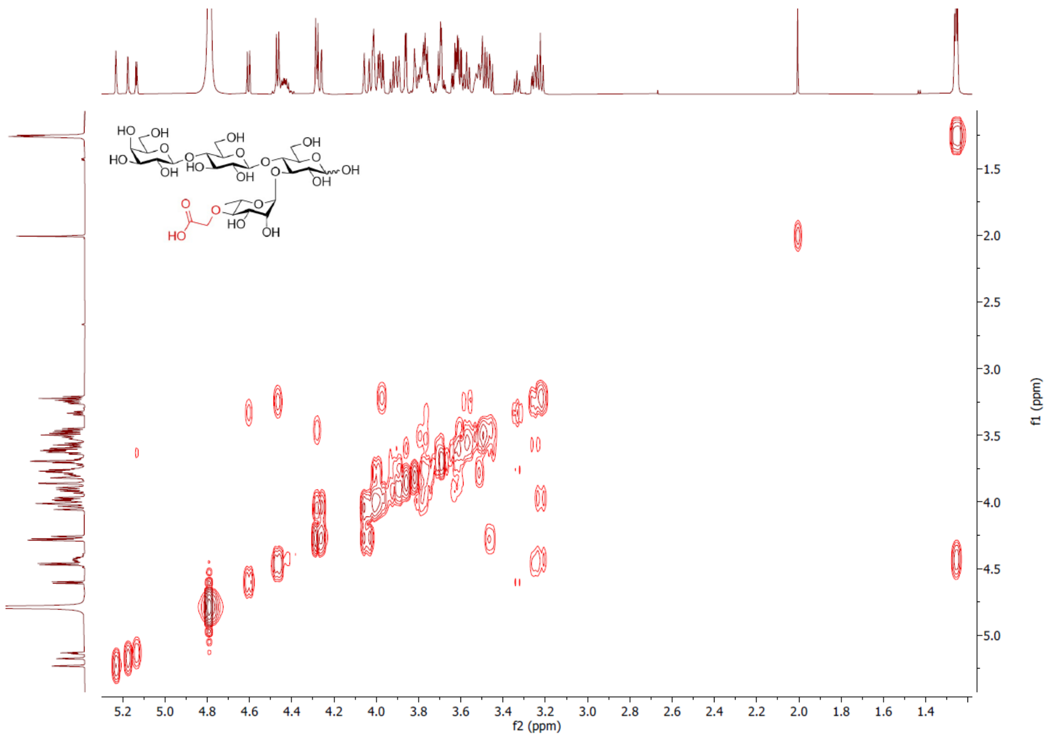

COSY NMR of 4mer-VI (D_2O)

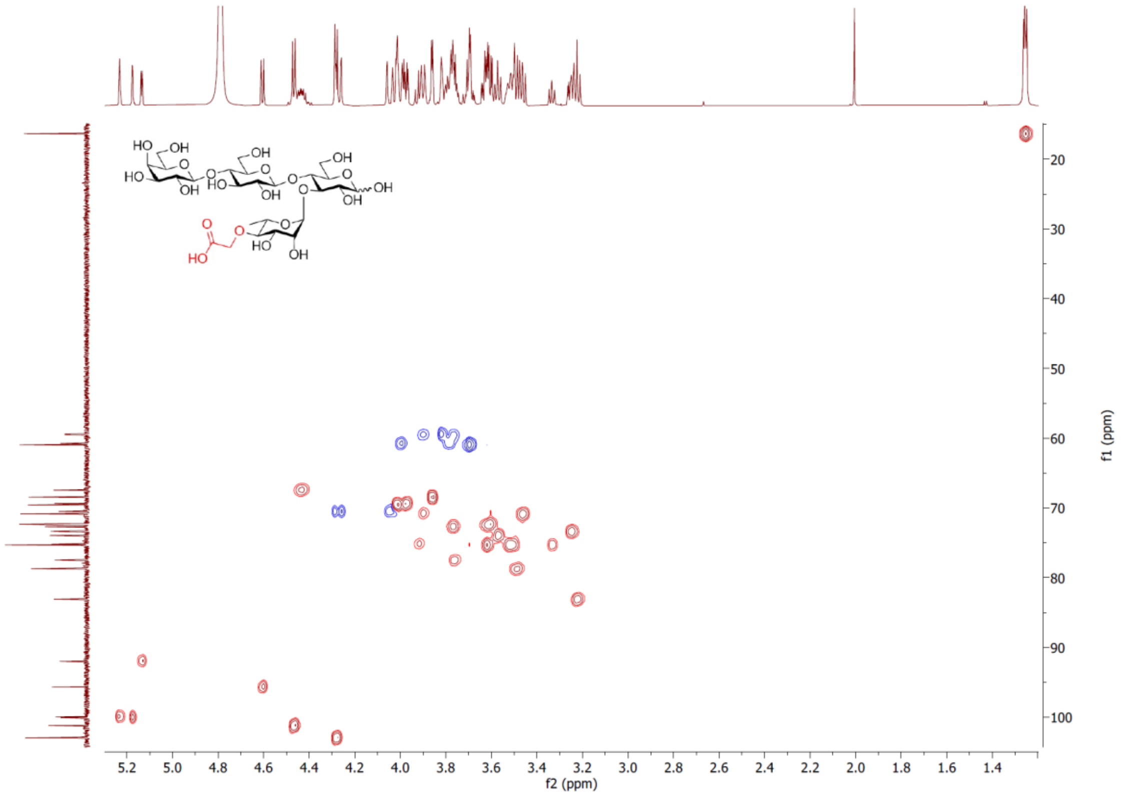

HSQC NMR of 4mer-VI (D_2O)

3.5.6 4mer-V

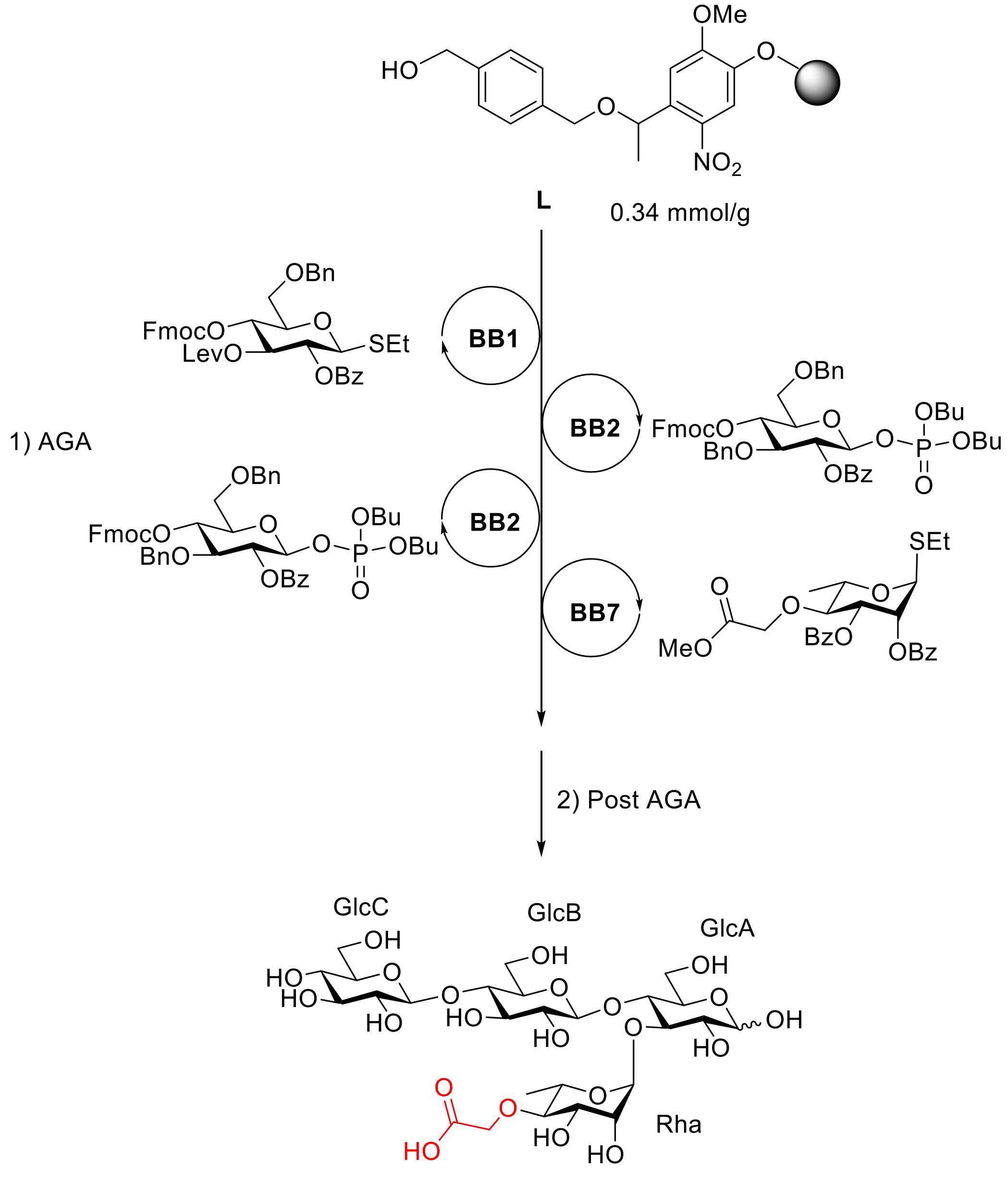

4mer-V

Step	Sequence	Modules	Notes
**AGA**		**A**	**L** swelling
	**BB1**	**B**, **C1**, **D**, **E1**	**C1**: (-20°C for 20 min, 0°C for 40 min)
	**BB2**	**B**, **C2**, **D**, **E1**	**C2**: (-30°C for 30 min, -10°C for 40 min)
	**BB2**	**B**, **C2**, **D**, **E1, D, E2**	**C2**: (-30°C for 30 min, -10°C for 40 min)

	**BB7**	**B**, **C1**, **D**	**C1**: (-20°C for 20 min, 0°C for 40 min)
**Post-AGA**		**I1**, **L** (Method A)**,** **J1**, **K2**, **L** (Method C1)	**L** (Method B1, t_R = 26.6, 27.1 min)

Automated synthesis (was conducted in 0.019 mmol scale), global deprotection, and purification afforded **4mer-V** as a white solid (4.0 mg, 30% overall yield, α : β = 45 : 55).

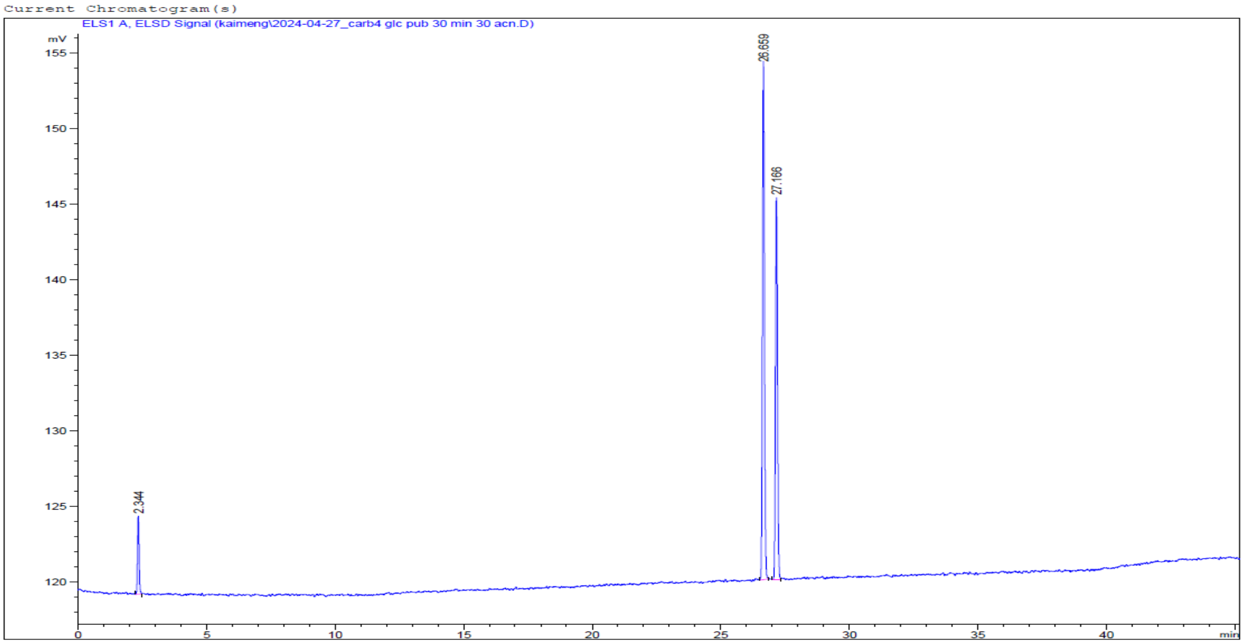

RP-HPLC of **4mer-V** (ELSD trace, Method B2 t_R = 26.6, 27.1 min).

^{1}H NMR (700 MHz, D_2O) δ 5.24 (d, *J* = 1.7 Hz, 0.5H, H-1β, Rha), 5.18 (d, *J* = 1.7 Hz, 0.5H, H-1α Rha), 5.14 (d, *J* = 3.8 Hz, 0.5H, H-1α GlcA), 4.61 (d, *J* = 7.9 Hz, 0.5H, H-1β GlcA), 4.47 (d, *J* = 7.9 Hz, 1H, H-1 GlcB), 4.44 (m, 1H, H-5 Rha), 4.35 (d, *J* = 7.9 Hz, 1H, H-1 GlcC), 4.32 (d, *J* = 16.6, 1H), 4.15 (d, *J* = 16.9, 1H), 4.03 – 3.97 (m, 3H), 3.94 – 3.89 (m, 1.5H), 3.86 – 3.82 (m, 2H), 3.80 – 3.75 (m, 3H), 3.68 (dd, *J* = 12.4, 5.5 Hz, 1H), 3.64 (dd, *J* = 9.8, 3.8 Hz, 0.5H), 3.57 (td, *J* = 9.1, 1.6 Hz, 1H), 3.52 (m, 1.5H), 3.49 – 3.44 (m, 2H), 3.39 (m, 1H), 3.37 – 3.33 (m, 1.5H), 3.25 (m, 3H), 1.26 (dd, *J* = 6.4, 3.6 Hz, 3H). ^{13}C NMR (176 MHz, D_2O) δ 176.51, 102.58 (C-1, GlcC), 101.19, 101.17 (C-1, GlcB), 100.09, 99.97 (C-1, Rha), 95.67 (C-1β, GlcA), 91.99 (C-1α, GlcA), 82.99, 82.98, 79.14, 77.43, 75.91, 75.32, 75.30, 75.20, 75.11, 73.90, 73.88, 73.41, 73.38, 73.05, 72.67, 72.47, 70.79, 70.12, 69.72, 69.54, 69.49, 69.32, 67.37, 67.31, 60.78, 60.76, 60.42, 59.50, 59.41, 16.39. (ESI-HRMS) m/z 731.2216 $[M+Na]^+$ ($C_{26}H_{44}NaO_{22}$ requires 731.2222).

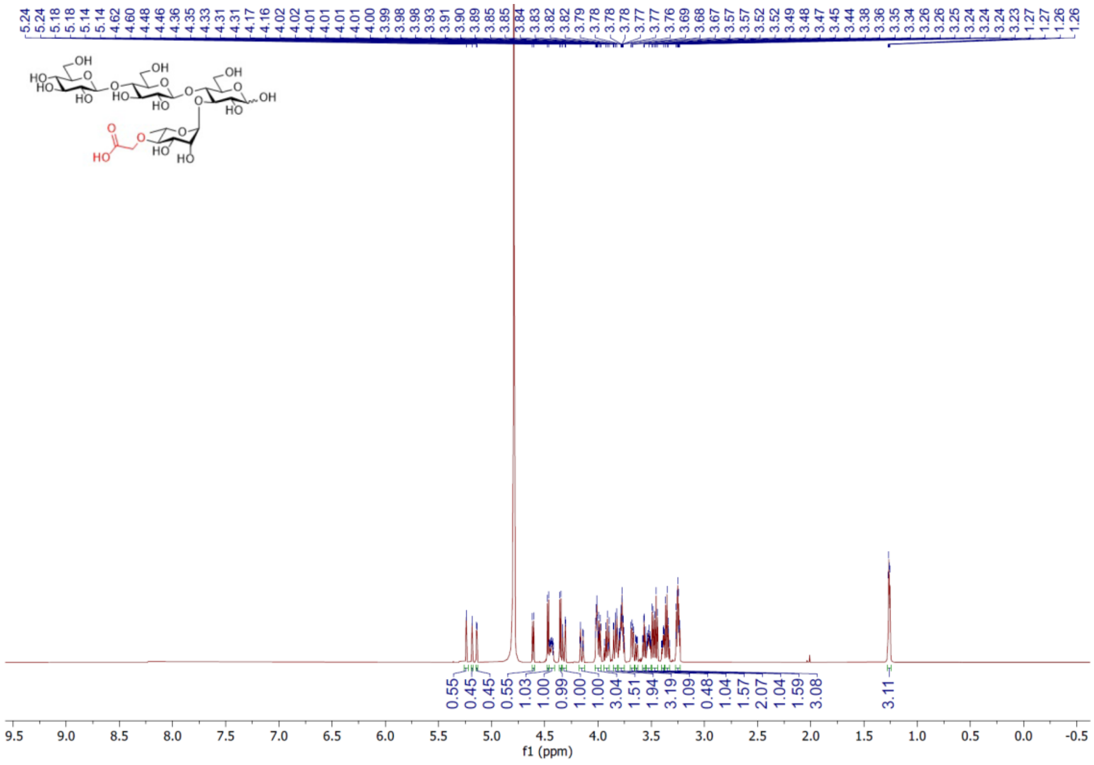

^{1}H NMR of 4mer-V (700 MHz, D_2O)

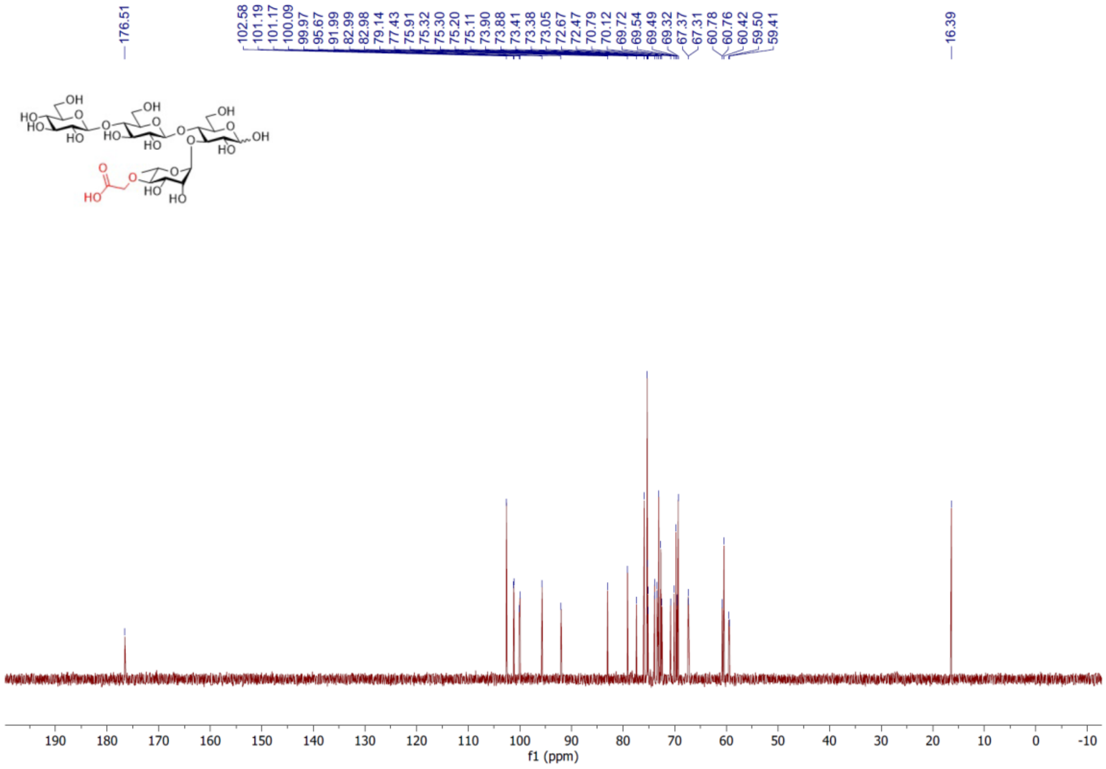

^{13}C NMR of 4mer-V (176 MHz, D_2O)

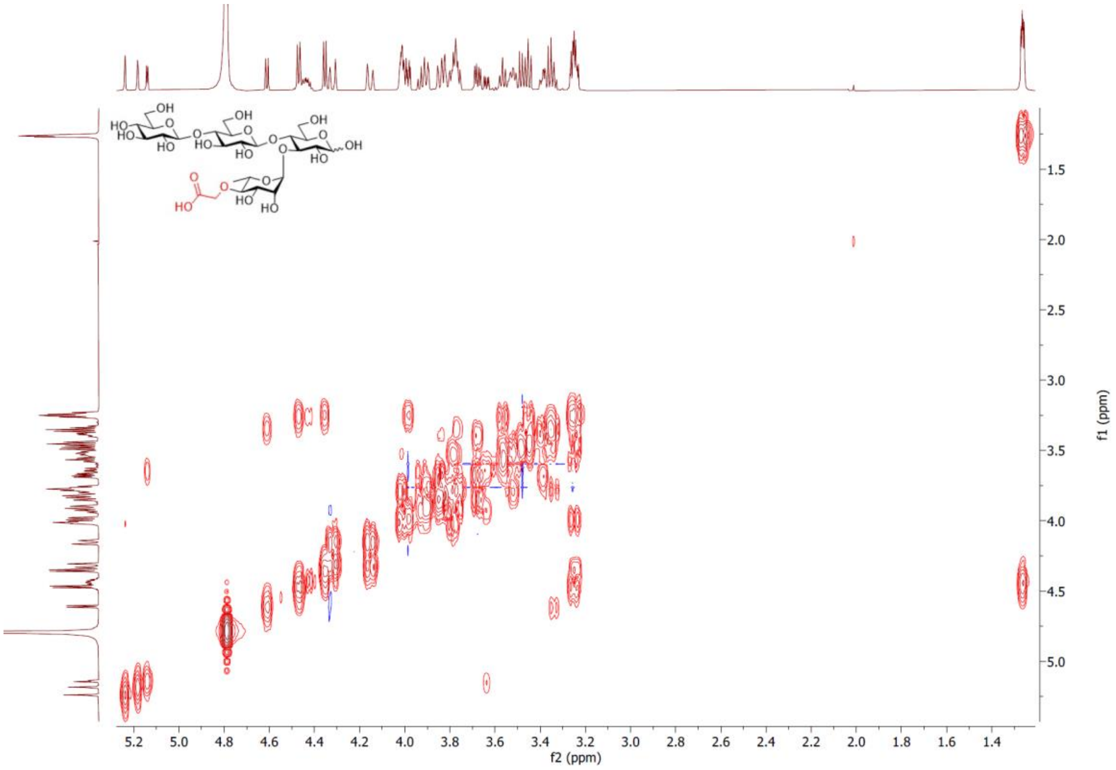

COSY NMR of 4mer-V (D_2O)

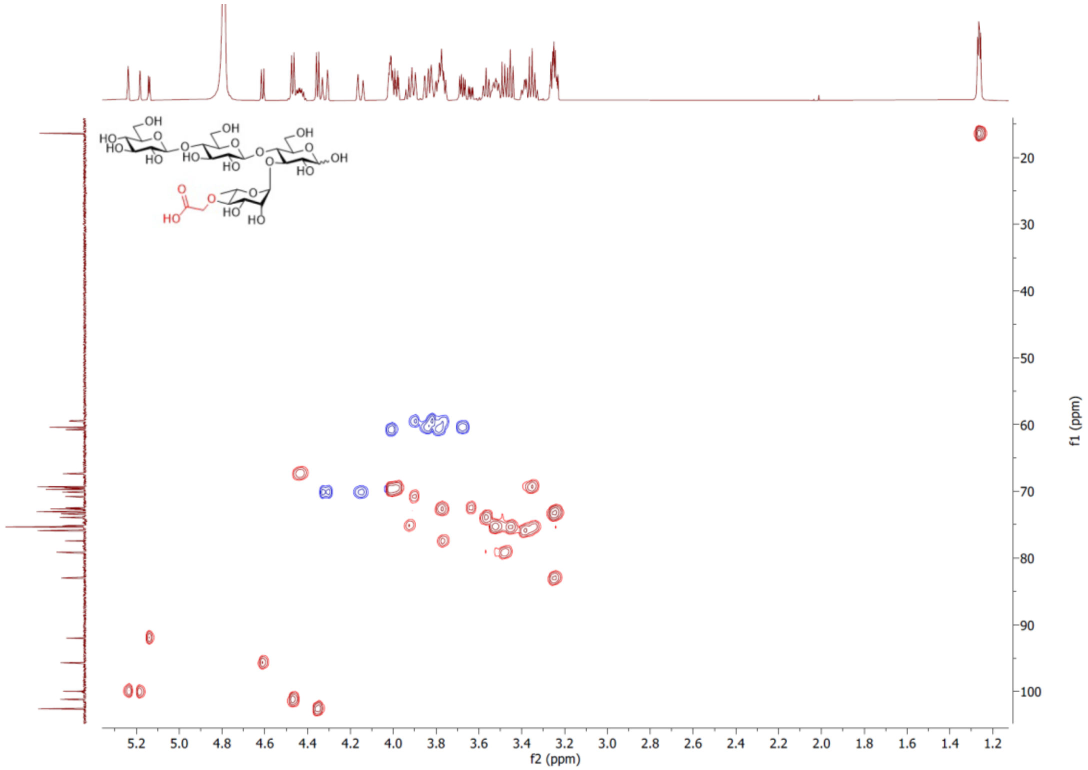

HSQC NMR of 4mer-V (D_2O)

3.5.7 5mer

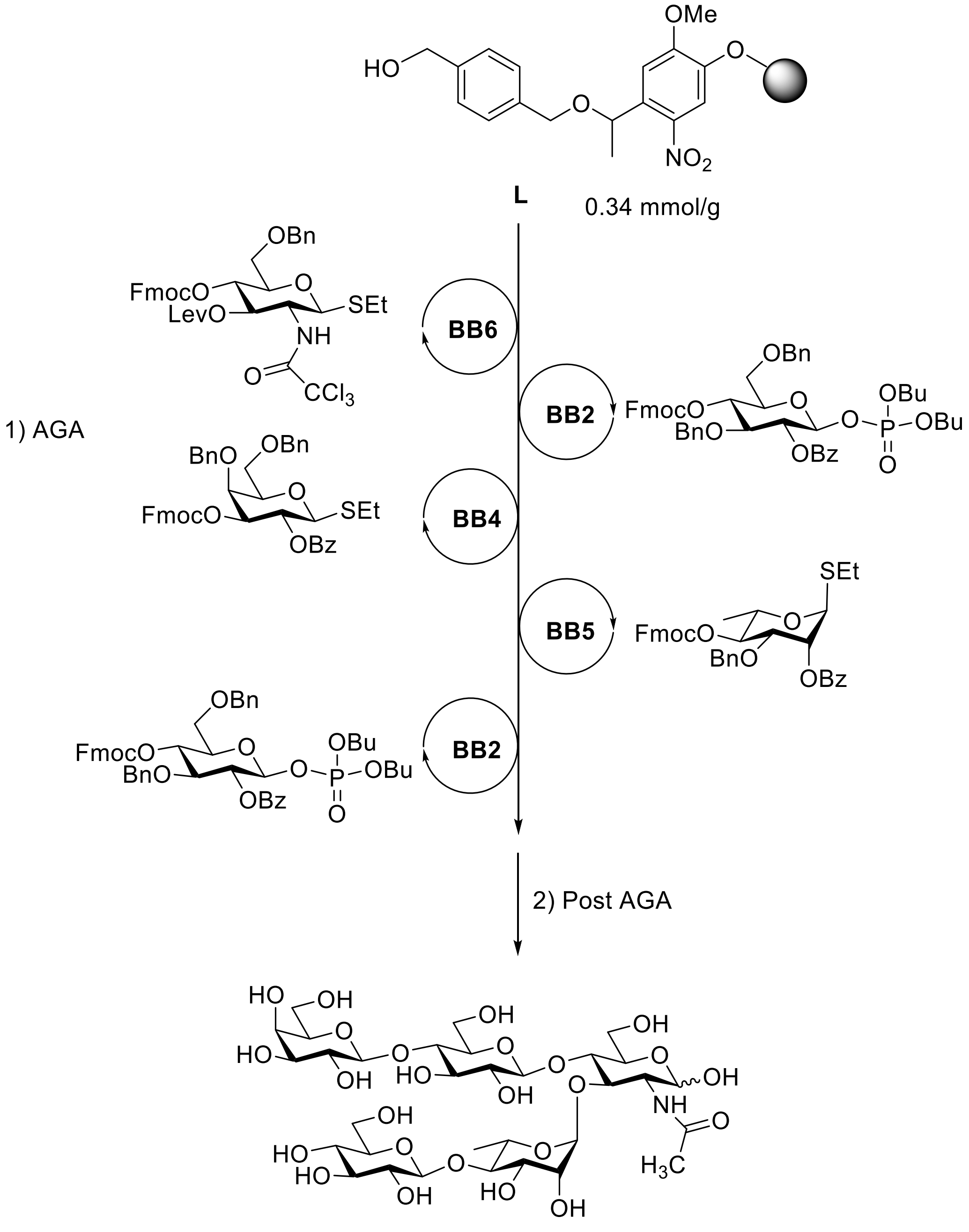

5mer

Step	Sequence	Modules	Notes
AGA		**A**	**L** swelling
	**BB6**	**B**, **C1**, **D**, **E1**	**C1**: (-20°C for 30 min, 0°C for 50 min)
	**BB2**	**B**, **C2**, **D**, **E1**	**C2**: (-30°C for 30 min, -10°C for 40 min)
	**BB4**	**B**, **C2**, **D**, **E1, D, E2**	**C2**: (-30°C for 30 min, -10°C for 40 min)

	**BB5**	**B**, **C1**, **D**, **E1**	**C1**: (-20°C for 20 min, 0°C for 40 min)
	**BB2**	**B**, **C2**, **D**, **E1**	**C2**: (-30°C for 30 min, -10°C for 40 min)
Post-AGA		**I1**, **L** (Method A)**,** **J1**, **K2**, **L** (Method C2)	**L** (Method B2, t_R = 35.5 min, 36.0 min)

Automated synthesis (was conducted in 0.019 mmol scale), global deprotection, and purification afforded **5mer** as a white solid (4.5 mg, 28% overall yield, α : β =60 : 40).

^{1}H NMR (700 MHz, D_2O) δ 5.09 (d, *J* = 3.6 Hz, 0.6H, H-1α, GlcNAc), 4.90 (d, *J* = 1.8 Hz, 1H, H-1, Rha), 4.72 (d, *J* = 7.9 Hz, 0.4H, H-1β, GlcNAc), 4.64 (d, *J* = 8.0 Hz, 1H, H-1, GlcC), 4.51 (dd, *J* = 7.9, 2.8 Hz, 1H, H-1, GlcB), 4.43 (m, 1H, H-5, Rha), 4.35 (d, *J* = 7.7 Hz, 1H, H-1, Gal), 4.09 (dd, *J* = 10.4, 3.6 Hz, 0.6H), 4.02 (dd, *J* = 12.3, 2.0 Hz, 1H), 3.99 – 3.92 (m, 2.6H), 3.91 – 3.86 (m, 3H), 3.86 – 3.84 (m, 1.2H), 3.84 – 3.78 (m, 2.2H), 3.77 (m, 1H), 3.75 – 3.70 (m, 3H), 3.68 (dd, *J* = 12.4, 5.7 Hz, 1H), 3.65 – 3.59 (m, 3H), 3.55 (m, 1.4H), 3.50 (dd, *J* = 10.0, 7.8 Hz, 1H), 3.48 – 3.42 (m, 2H), 3.39 (m, 1H), 3.36 – 3.32 (m, 1H), 3.27 (m, 2H), 2.02 (d, *J* = 1.5 Hz, 3H), 1.27 (dd, *J* = 6.4, 2.6 Hz, 3H). ^{13}C NMR (176 MHz, D_2O) δ 174.30, 174.13, 103.50 (C-1, GlcC), 103.21 (C-1, Gal), 101.05, 101.00 (C-1, GlcB), 100.24, 100.15 (C-1, Rha), 94.24 (C-1β, GlcNAc), 90.91 (C-1α, GlcNAc), 80.94, 80.88, 79.76, 79.72, 76.55, 76.03, 75.85, 75.28, 75.24, 75.16, 74.49, 74.26, 74.20, 74.18, 73.07, 73.01, 72.99, 72.35, 71.08, 70.87, 70.37, 70.32, 70.18, 70.13, 69.58, 68.36, 67.10, 67.04, 60.95, 60.89, 60.64, 59.51, 59.44, 56.81, 53.88, 22.03, 21.84, 16.77, 16.74. (ESI-HRMS) m/z 854.3184 $[M+H]^+$ ($C_{32}H_{56}NO_{25}$ requires 854.3141).

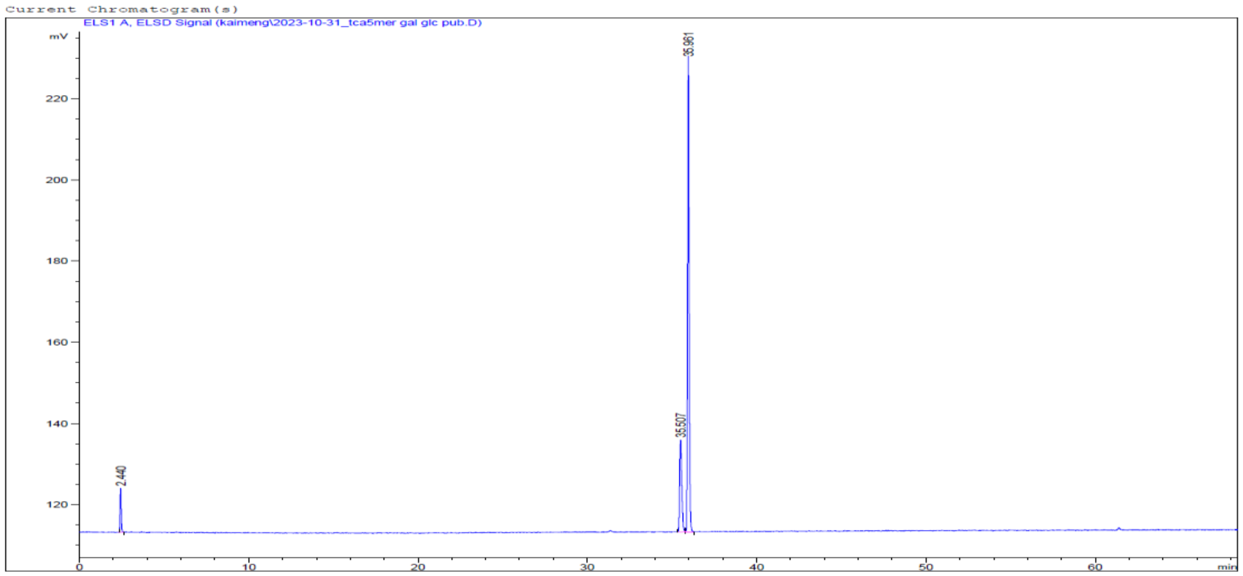

RP-HPLC of **5mer** (ELSD trace, Method B2 t_R = 35.5 min, 36.0 min).

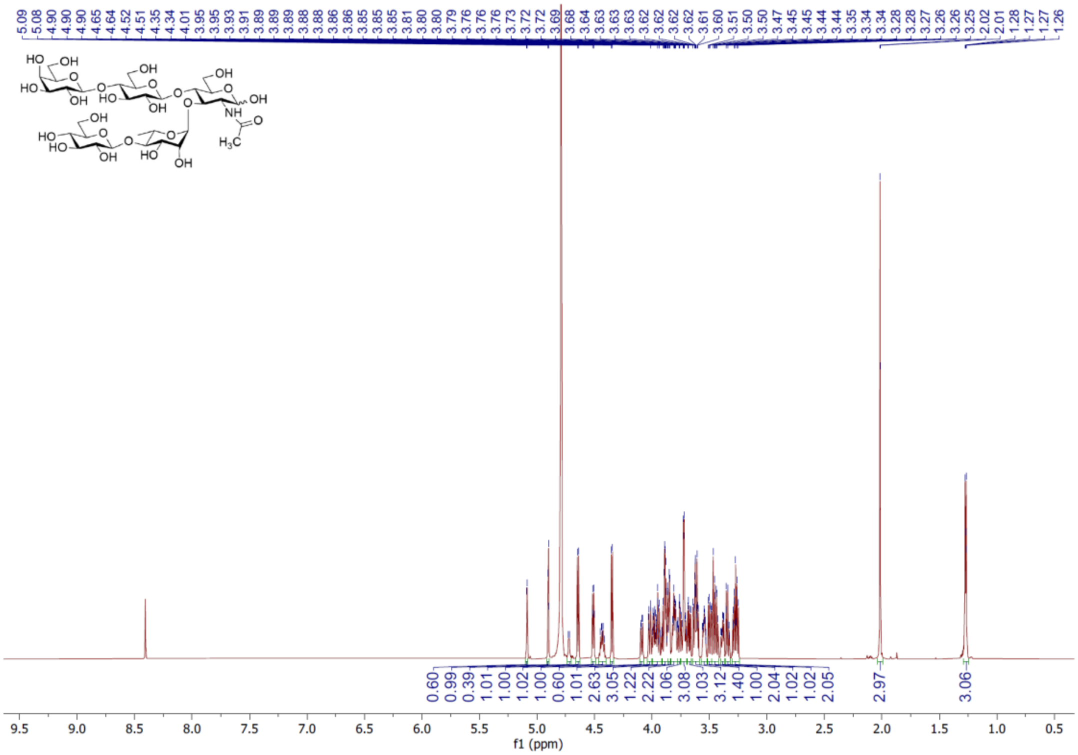

^{1}H NMR of 5mer (700 MHz, D_2O)

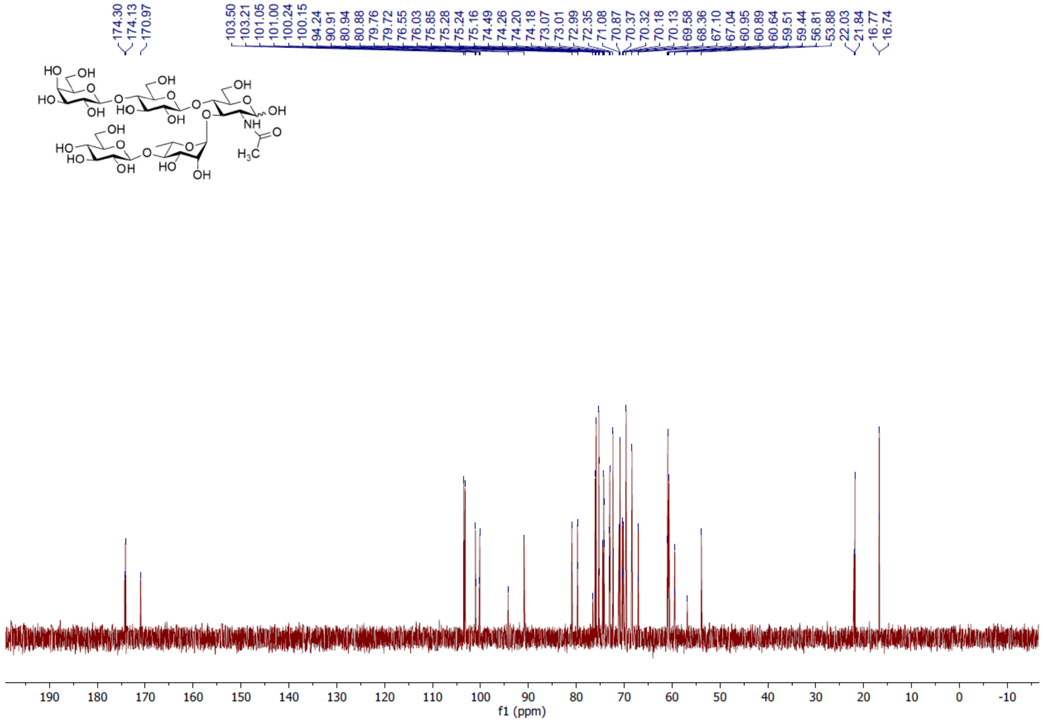

^{13}C NMR of 5mer (176 MHz, D_2O)

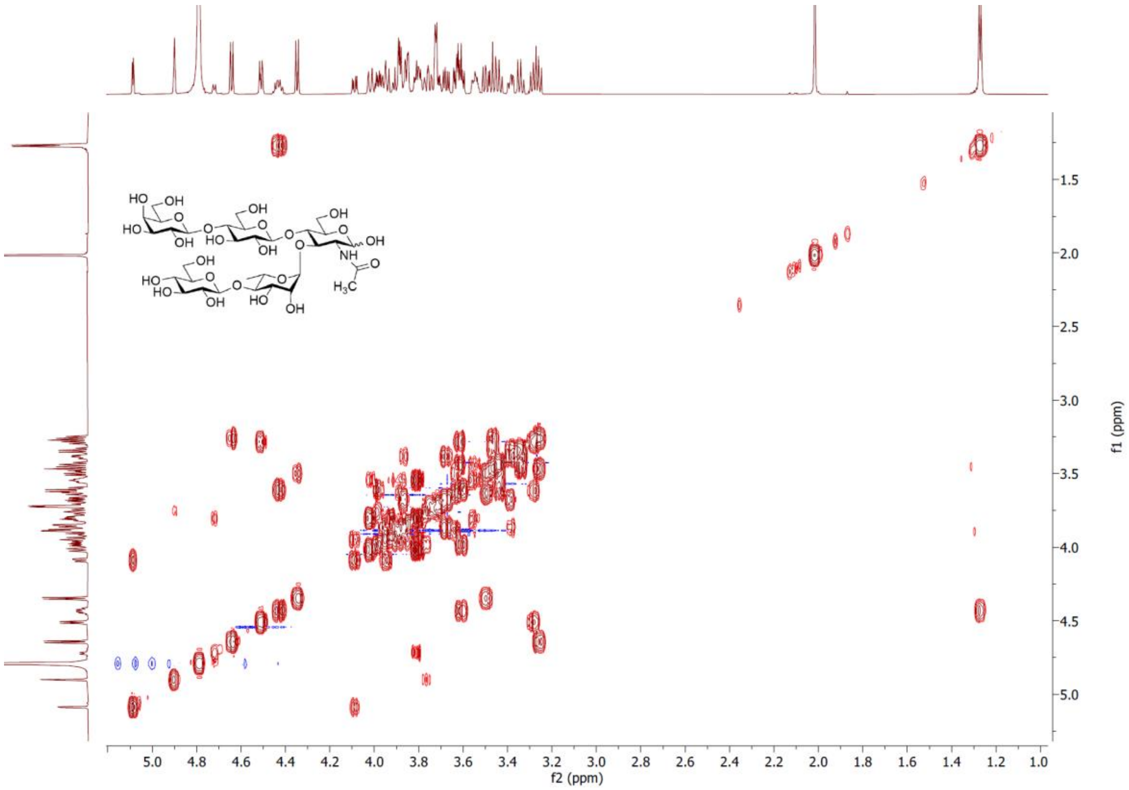

COSY NMR of 5mer (D_2O)

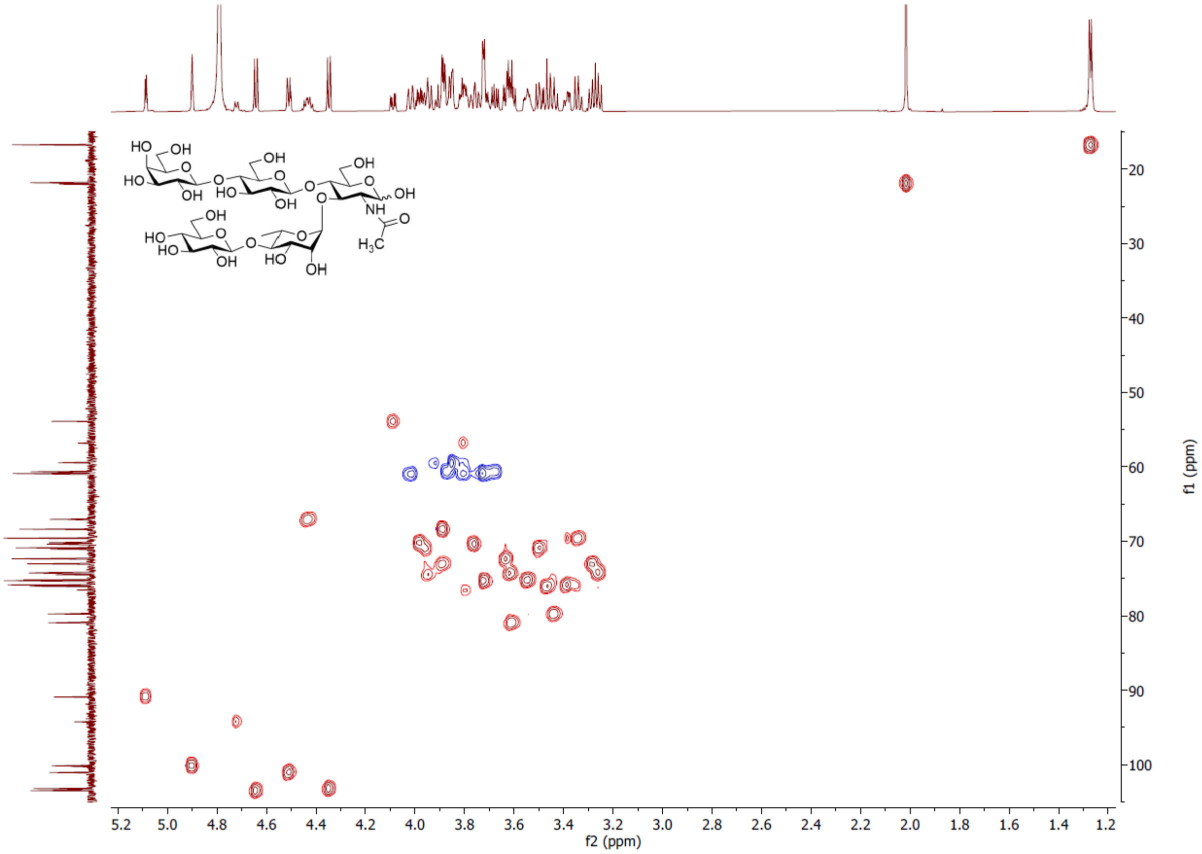

HSQC NMR of 5mer (D_2O)

4 Structural analysis

4.1 General materials and methods for molecular dynamics simulations

For all simulations, the modified version GLYCAM06 force field was used.[7] Initial conformations for single hairpin simulations were constructed with the Glycam Carbohydrate builder and tleap (https://glycam.org/). Both compounds with free reducing end were modelled as β anomers. The topology was subsequently converted using the python script acpype. Both simulations were performed in water as solvent using TIP5P as water model.[8] The simulation time for the single molecule experiments was 500 ns. Bonds involving hydrogens were constrained using the LINCS to allow a 2 fs time steps. Non-bonded interactions were cut-off at 1.4 nm, long range electrostatics were calculated using the particle mesh Ewald method.[9] After energy minimization (steepest descent algorithm) and before the production run, the systems were equilibrated at 300 K for 50 ns in a canonical (NVT) ensemble (constant number of particles, volume and temperature) and subsequently at 300 K and 1 bar for 50 ns in an isothermal-isobaric (NPT) ensemble. All molecular dynamics simulations were performed using Gromacs 5.1.2.[10] A Nosé-Hoover thermostat[11] kept the constant temperature of 303 K constant while a Parrinello-Rahman barostat[12] ensured a constant pressure of 1 bar. The analysis was visualized using OriginPro 2021b.

4.2 General materials and methods for NMR

^{1}H, ^{13}C, HSQC, 1D and 2D TOCSY, 2D ROESY, and 2D NOESY NMR spectra were recorded on a Varian 400-MR (400 MHz), Varian 600-NMR (600 MHz), Bruker Biospin AVANCE700 (700 MHz) spectrometer. Samples were prepared by dissolving lyophilized samples in D_2O. Proton resonances of the oligosaccharides were assigned using a combination of ^{1}H, 2D COSY, HSQC, 1D and 2D TOCSY. Selective 1D TOCSY (HOHAHA, pulse program: seldigpzs) spectra were recorded using different mixing times to assign all the resonances (d9 = 40, 80, 120, 160, and 200, 350, 450 ms). 2D TOCSY (pulse program: mlevphpp) spectra were recorded using mixing time (d9 = 80 ms). 2D ROESY (pulse program: reosyph.2) and 2D NOESY (pulse program: noesygpphpp) spectra were recorded using different mixing times (p15 = 200 ms for ROESY and d8 = 1000 ms for NOESY). Monosaccharide were named as follows: D-glucose (Glc), D-galactose (Gal), D-*N*-acetyl glucosamine (GlcNAc), L-rhamnose (Rha). Labelling of protons in a monosaccharide is done as follows: e.g. proton attached to C-1 of Rha is named “Rha1”. Resonances of residues at the reducing end (or with chemical shift affected by it) are additionally labelled with α or β.

4.3 Molecular dynamics simulations

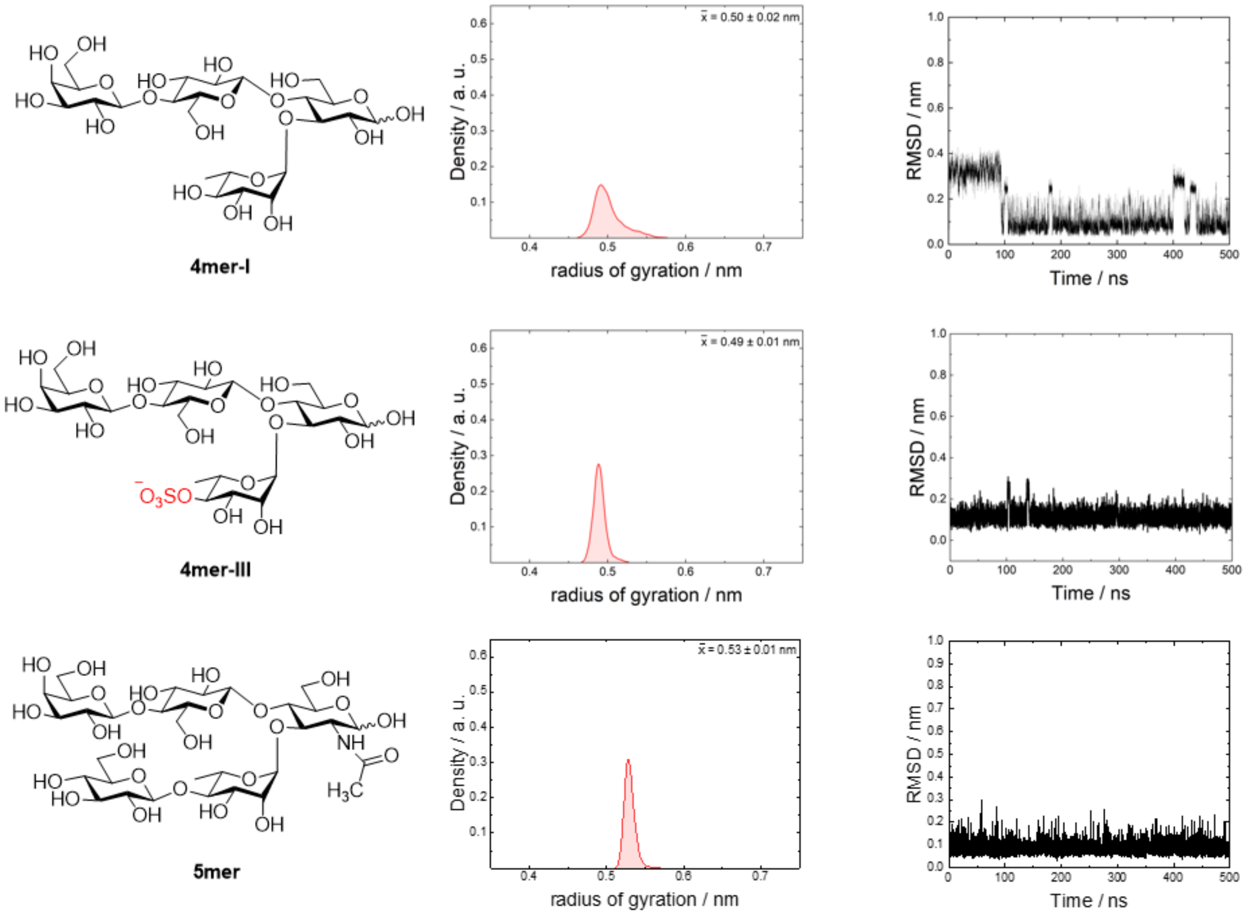

Figure S5 Radius of gyration and root-mean-square deviation (RMSD) analysis of **4mer-I, 4mer-III**, and **5mer**.

$$Definition\ \psi = C_1 - O_n - C_n - C_{n-1}$$

$$Definition\ \phi = O_5 - C_1 - O_n - C_n$$

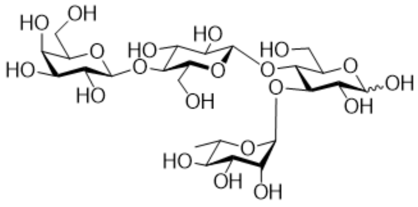

4mer-I

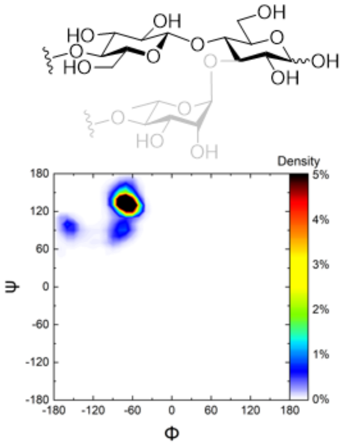

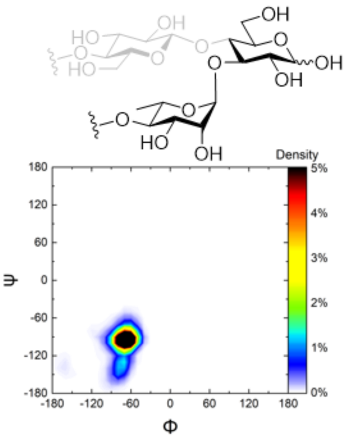

$$Definition\ \psi = C_1 - O_n - C_n - H_n$$

$$Definition\ \phi = H_1 - C_1 - O_n - C_n$$

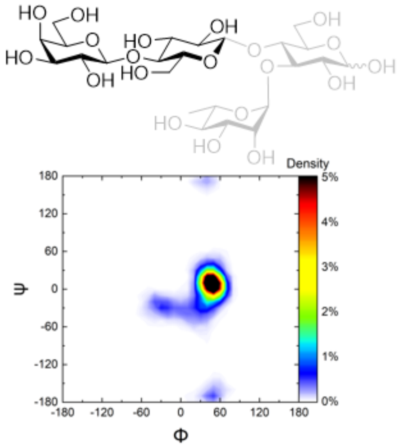

$Definition\ \psi = C_1 - O_n - C_n - C_{n-1}$

$Definition\ \phi = O_5 - C_1 - O_n - C_n$

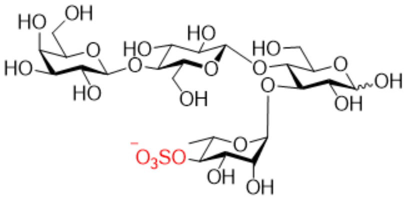

4mer-III

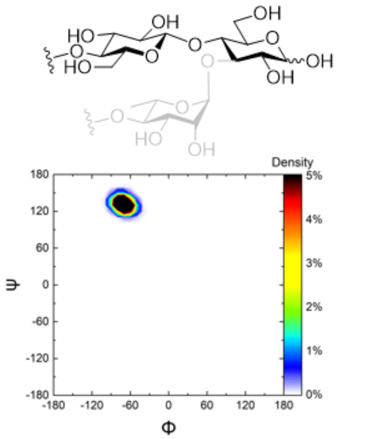

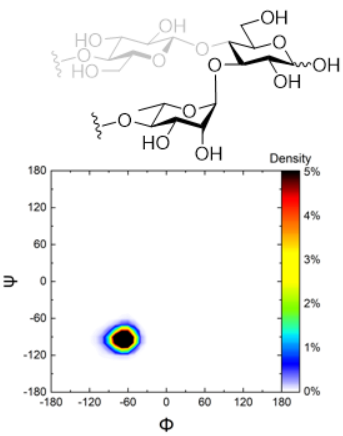

$Definition\ \psi = C_1 - O_n - C_n - H_n$

$Definition\ \phi = H_1 - C_1 - O_n - C_n$

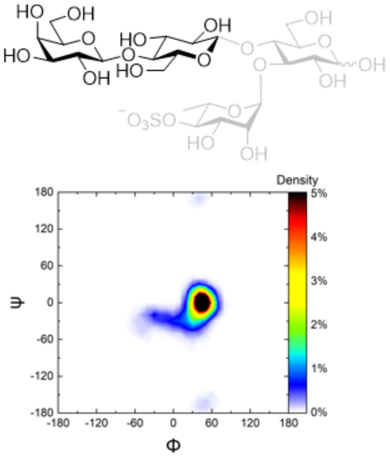

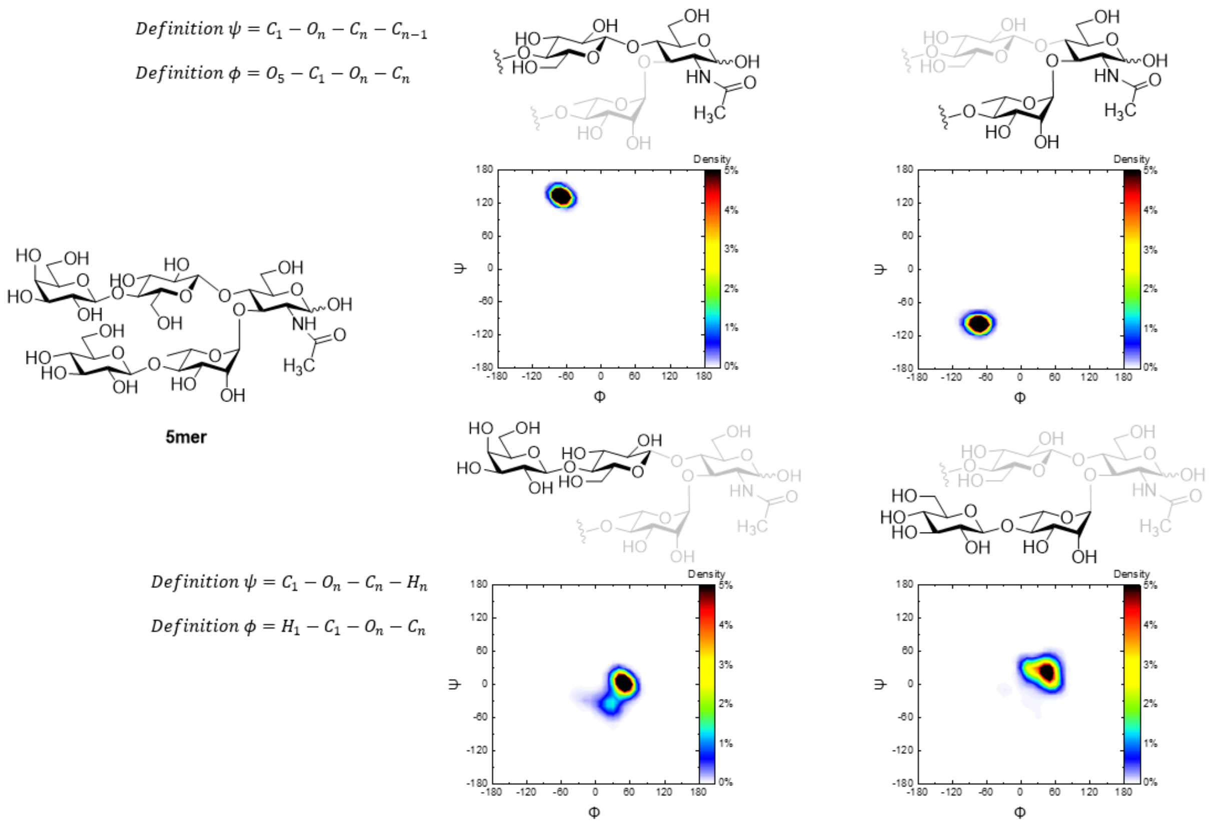

Figure S6 Ramachandran plots of **4mer-I, 4mer-III,** and **5mer.**

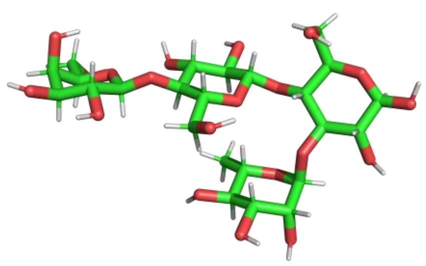

4mer-I

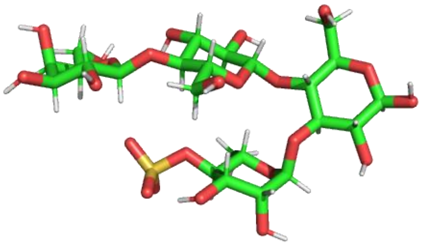

4mer-III

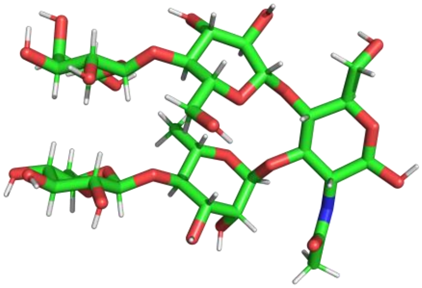

5mer

Figure S7 Representative snapshot of **4mer-I, 4mer-III,** and **5mer**.

4.4 NMR analysis

4.4.1 NMR analysis of 3mer

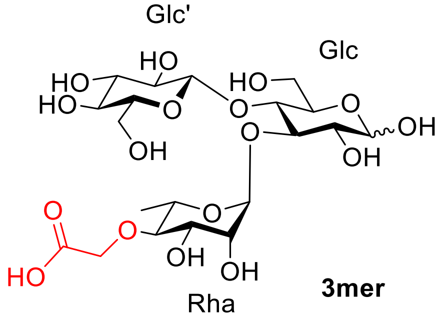

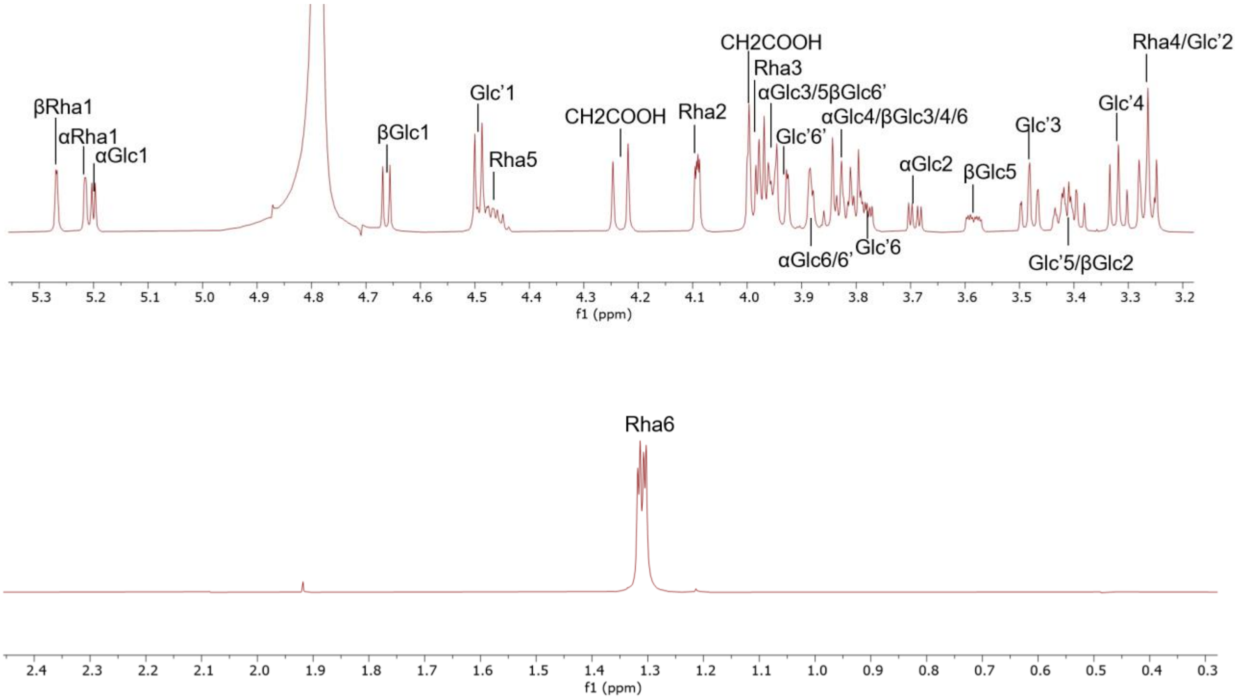

Figure S8 ^{1}H NMR of **3mer** (600 MHz, D_2O) with assignments.

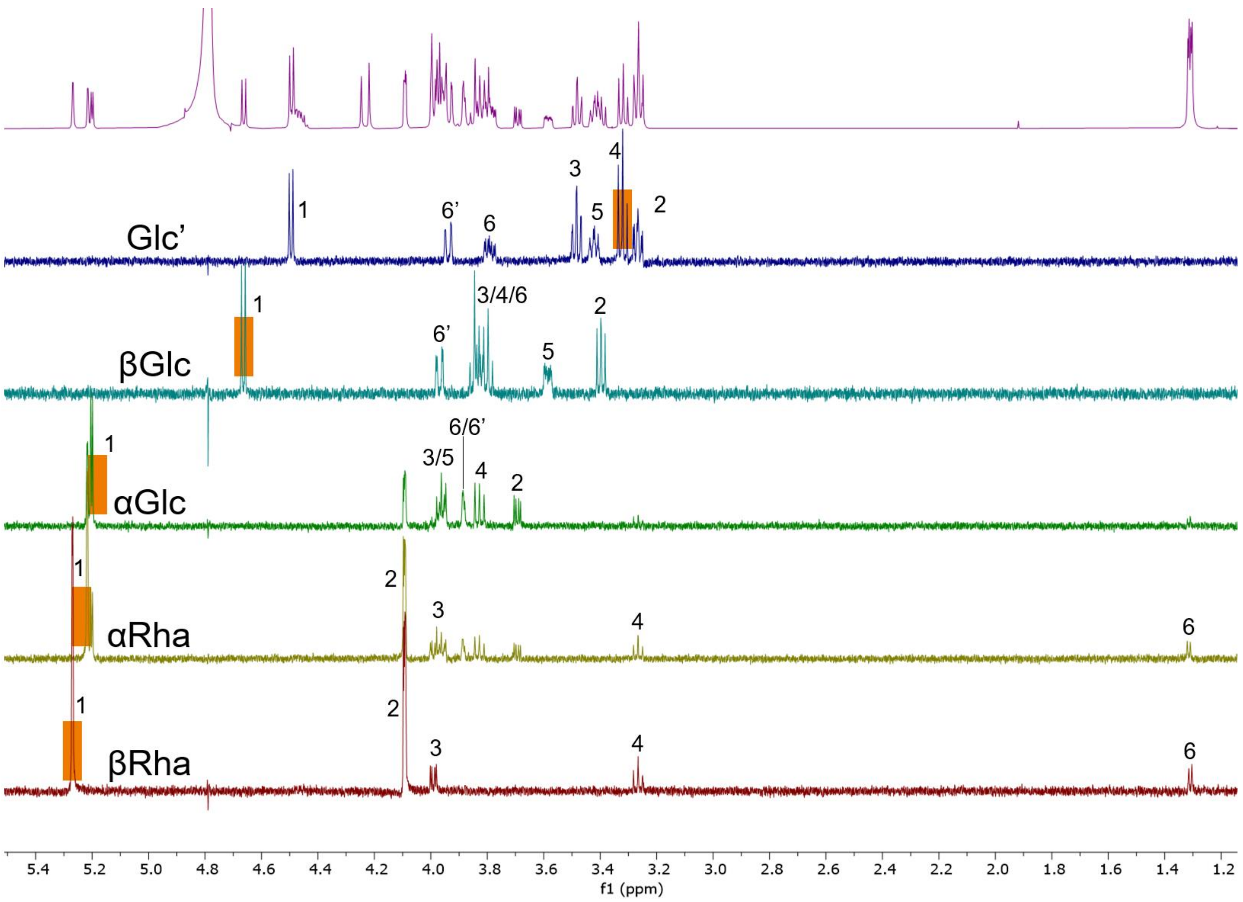

Figure S9 1D TOCSY (600 MHz, d9 = 200 ms, 293 K, D_2O) of **3mer** with assignments. Resonances chosen for selective excitation are highlighted in orange.

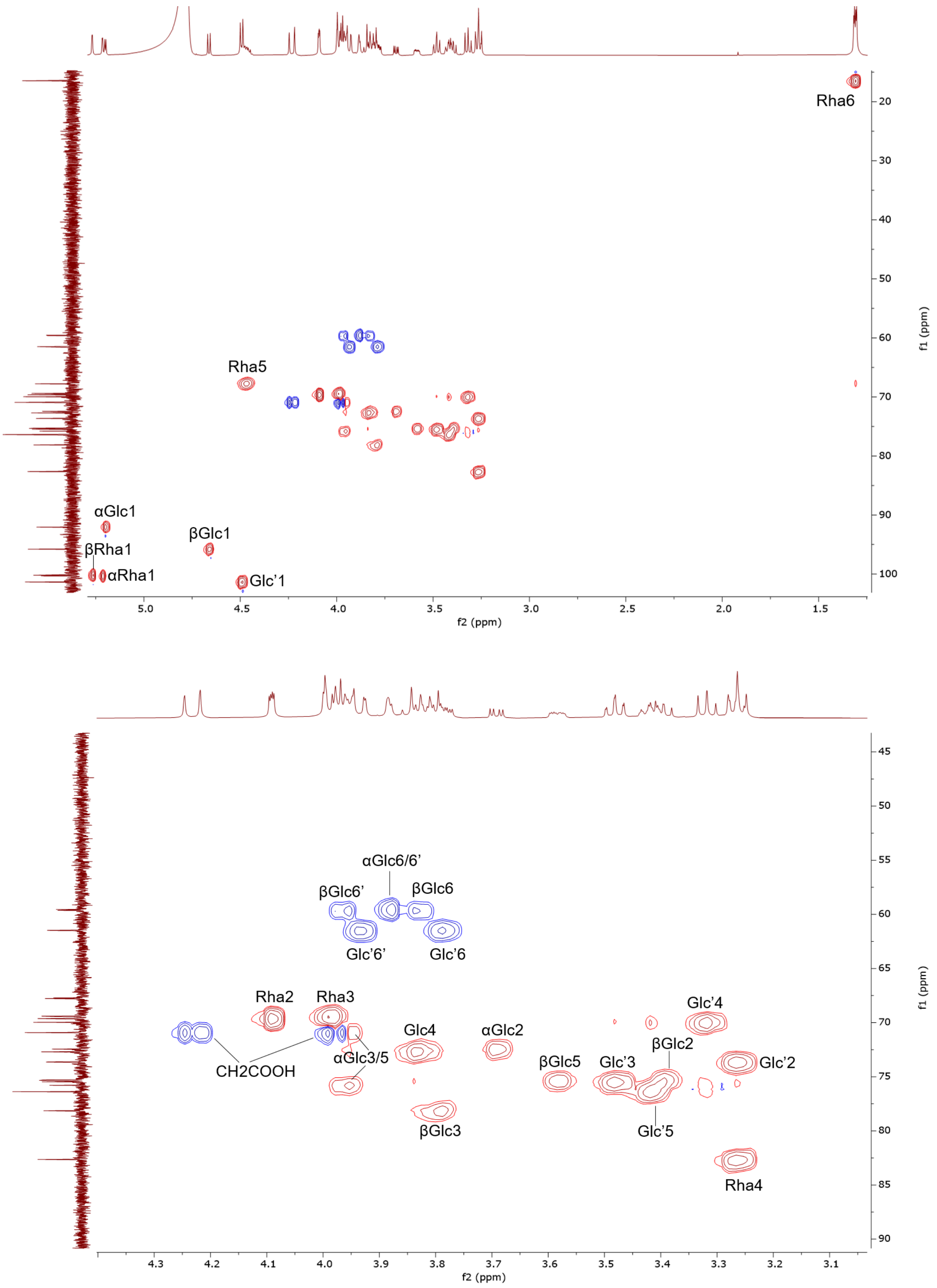

Figure S10 HSQC spectrum (D_2O) of **3mer** with assignments.

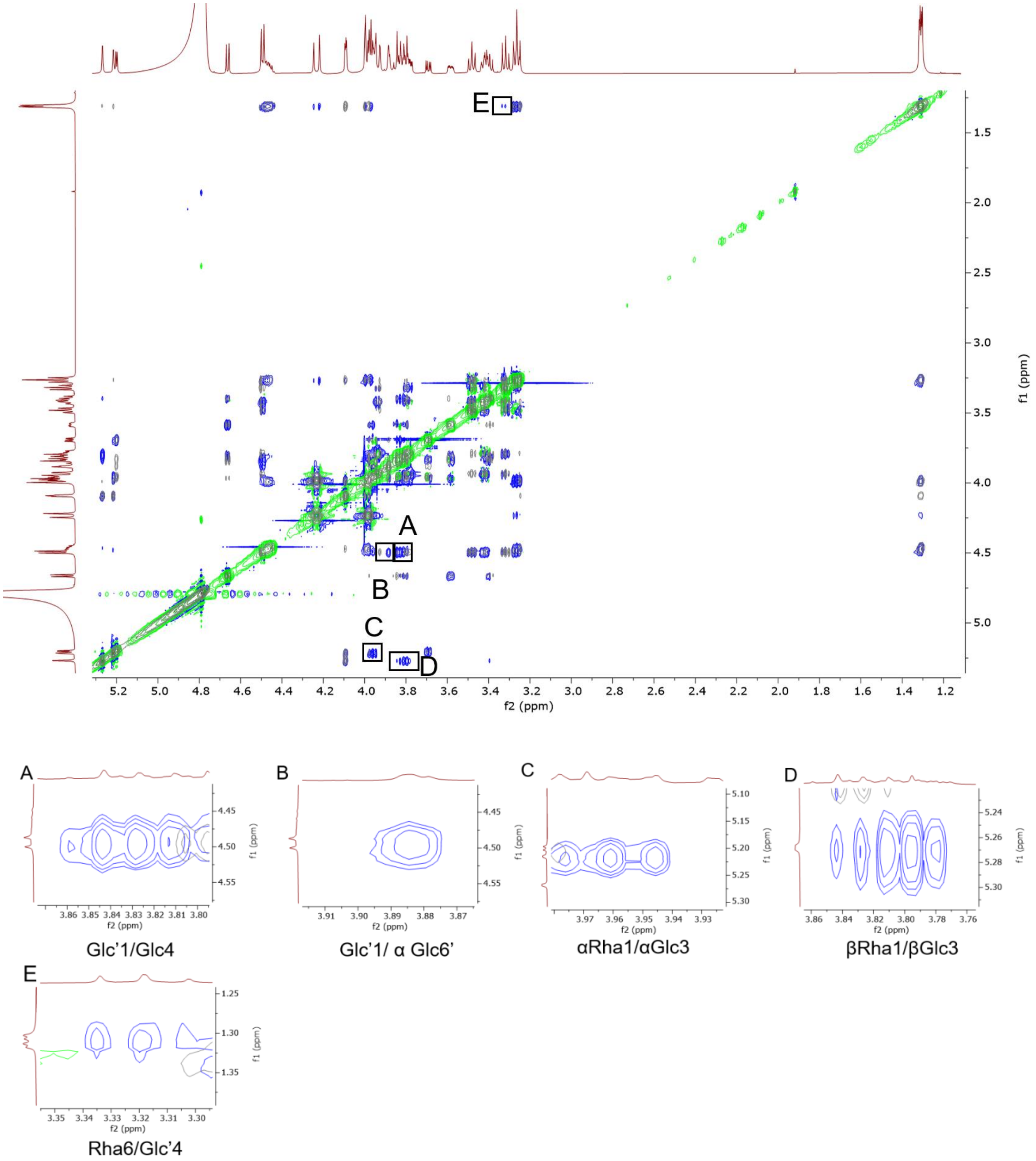

Figure S11 Overimposed 2D NOESY (green-blue, 600 MHz, d8 = 1000 ms, 293 K, D_2O) of **3mer** with assignments and 2D TOCSY spectrum (gray, 600 MHz, d9 = 80 ms, 293 K, D_2O).

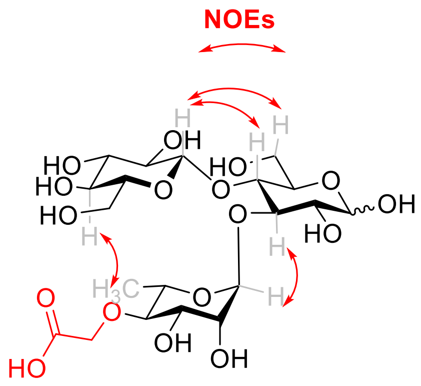

Figure S12 All experimentally observed NOEs (red arrows).

4.4.2 NMR analysis of 4mer-I

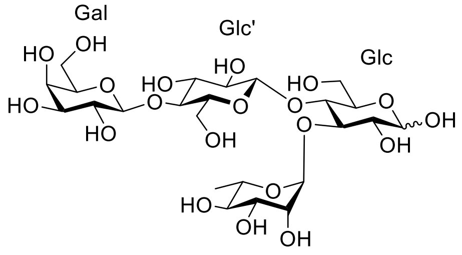

4mer-I

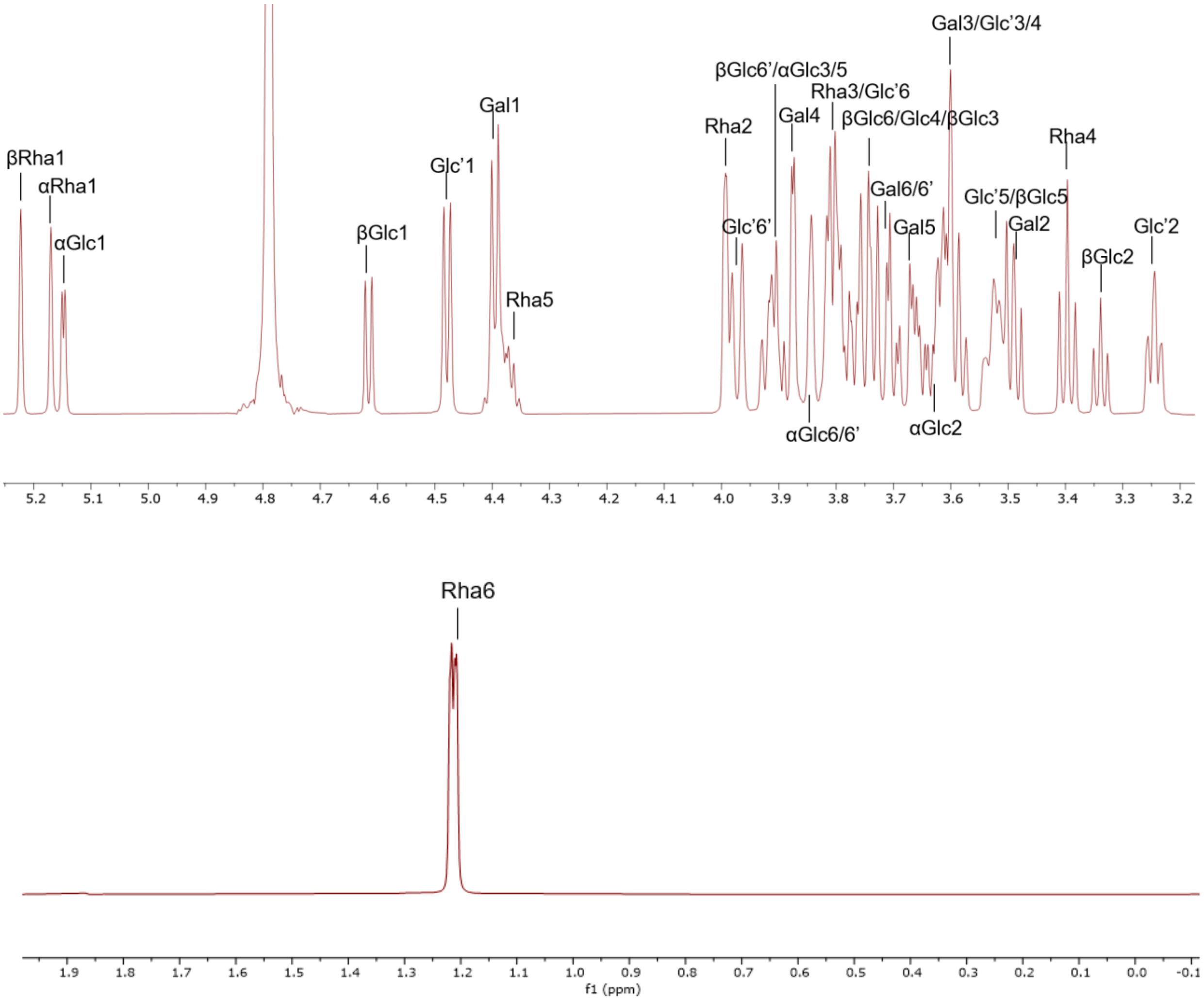

Figure S13^{1}H NMR of **4mer-I** (700 MHz, D_2O) with assignments.

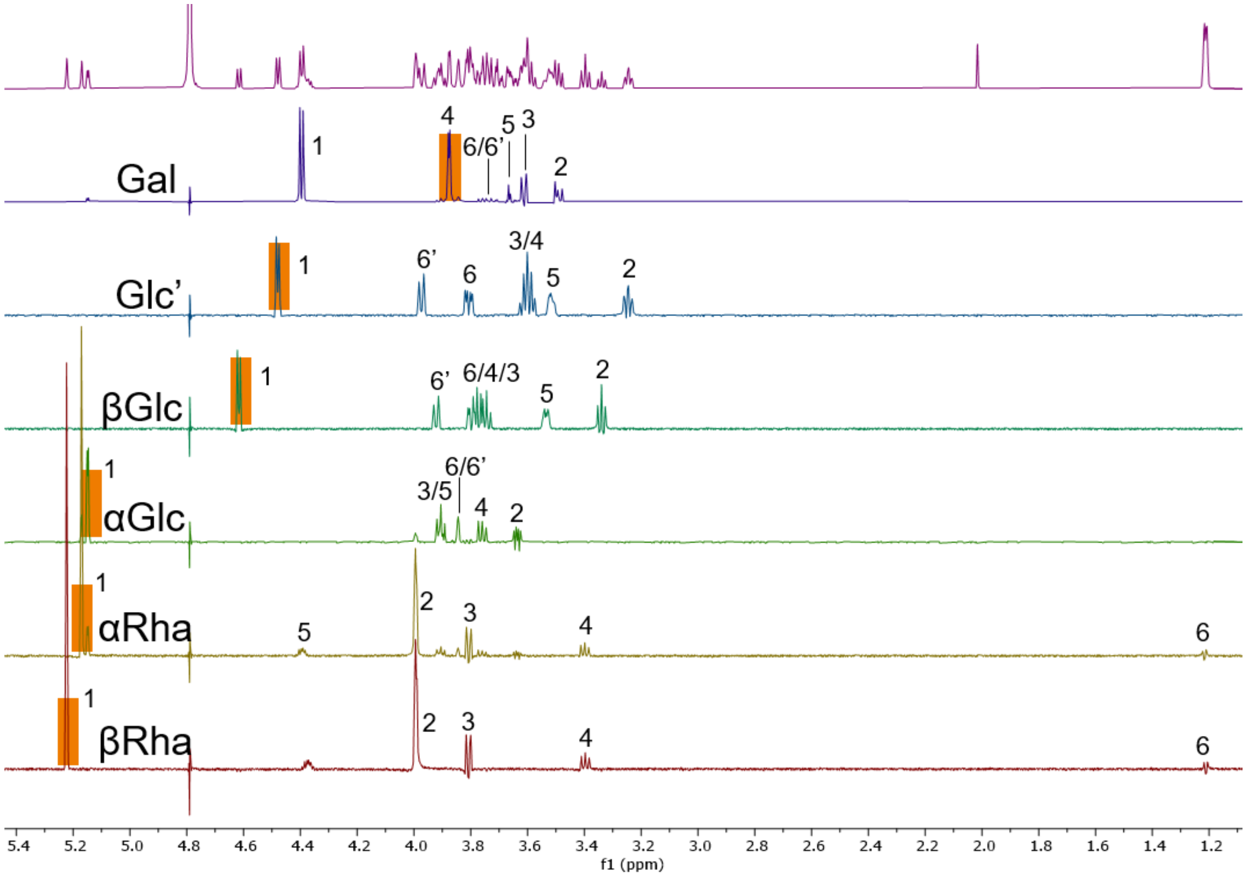

Figure S14 1D TOCSY (700 MHz, d9 = 200 ms, 293 K, D_2O) of **4mer-I** with assignments. Resonances chosen for selective excitation are highlighted in orange.

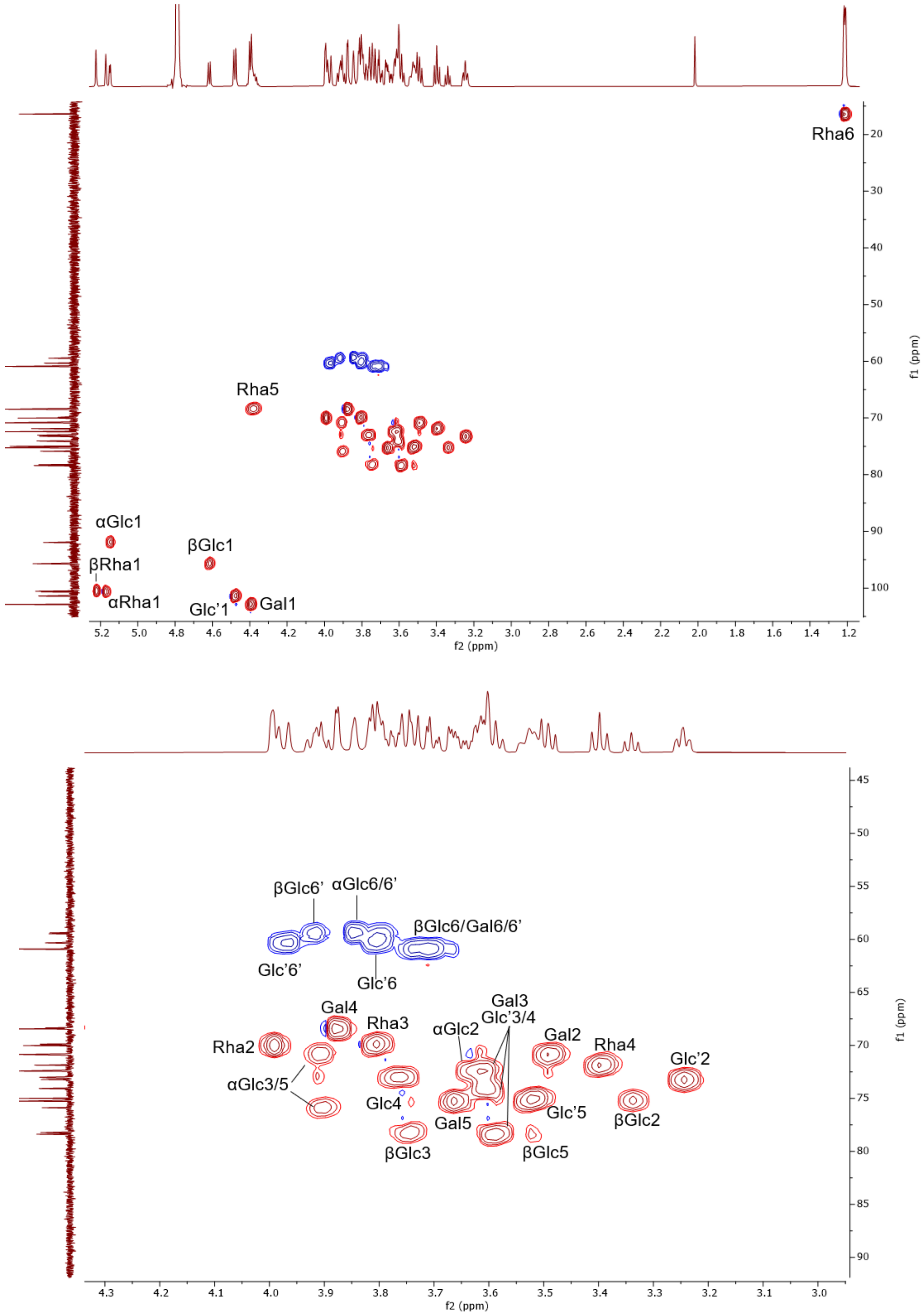

Figure S15 HSQC spectrum (D_2O) of **4mer-I** with assignments.

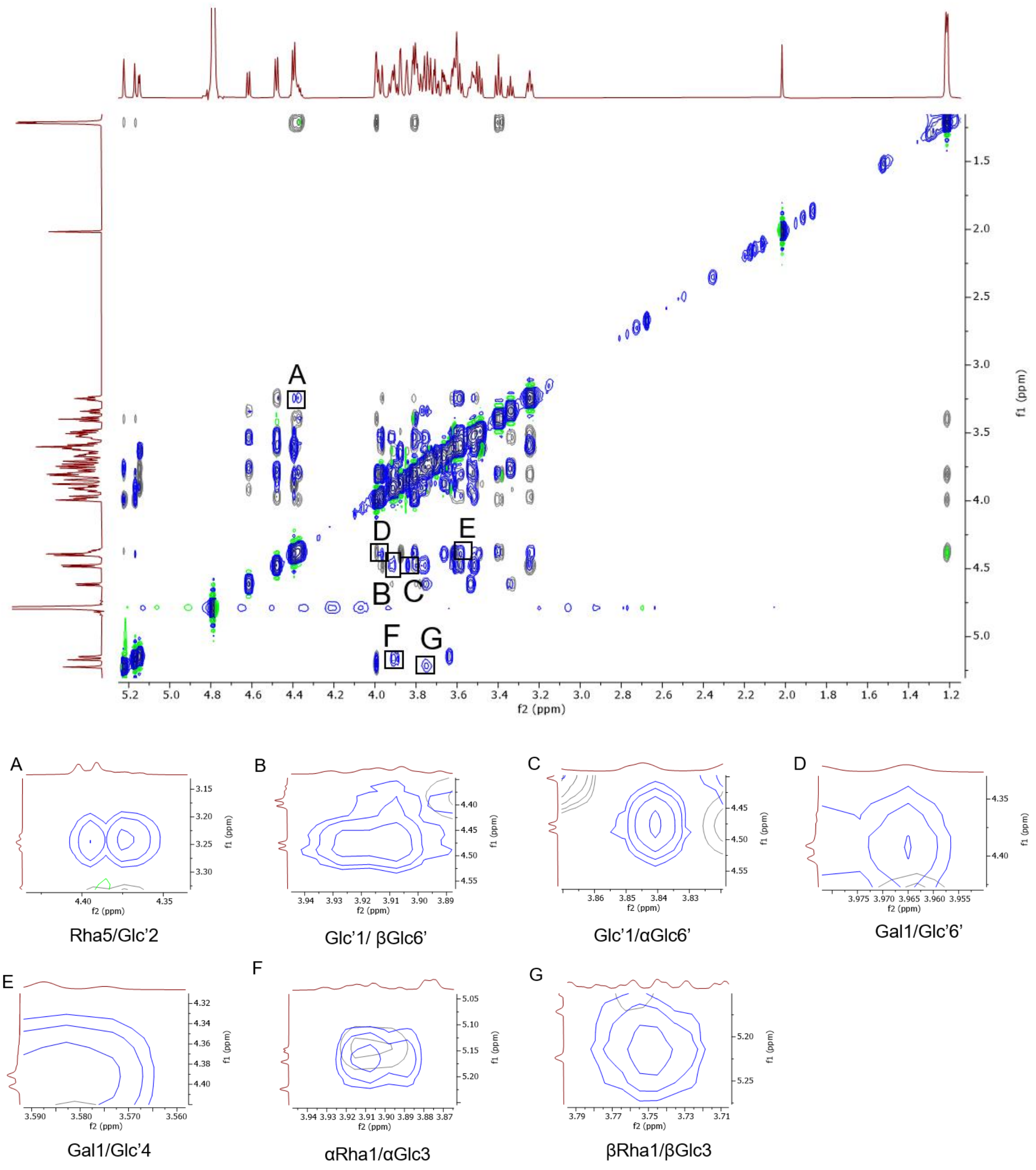

Figure S16 Overimposed 2D NOESY (green-blue, 700 MHz, d8 = 1000 ms, 293 K, D_2O) of **4mer-I** with assignments and 2D TOCSY spectrum (gray, 700 MHz, d9 = 80 ms, 293 K, D_2O).

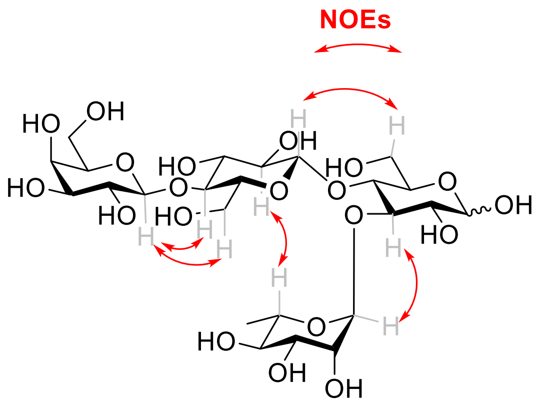

Figure S17 All experimentally observed NOEs (red arrows).

4.4.3 NMR analysis of 4mer-II

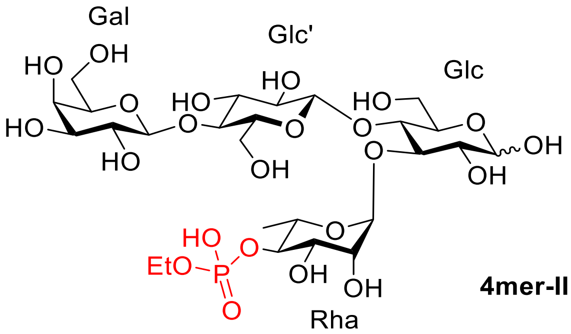

4mer-II

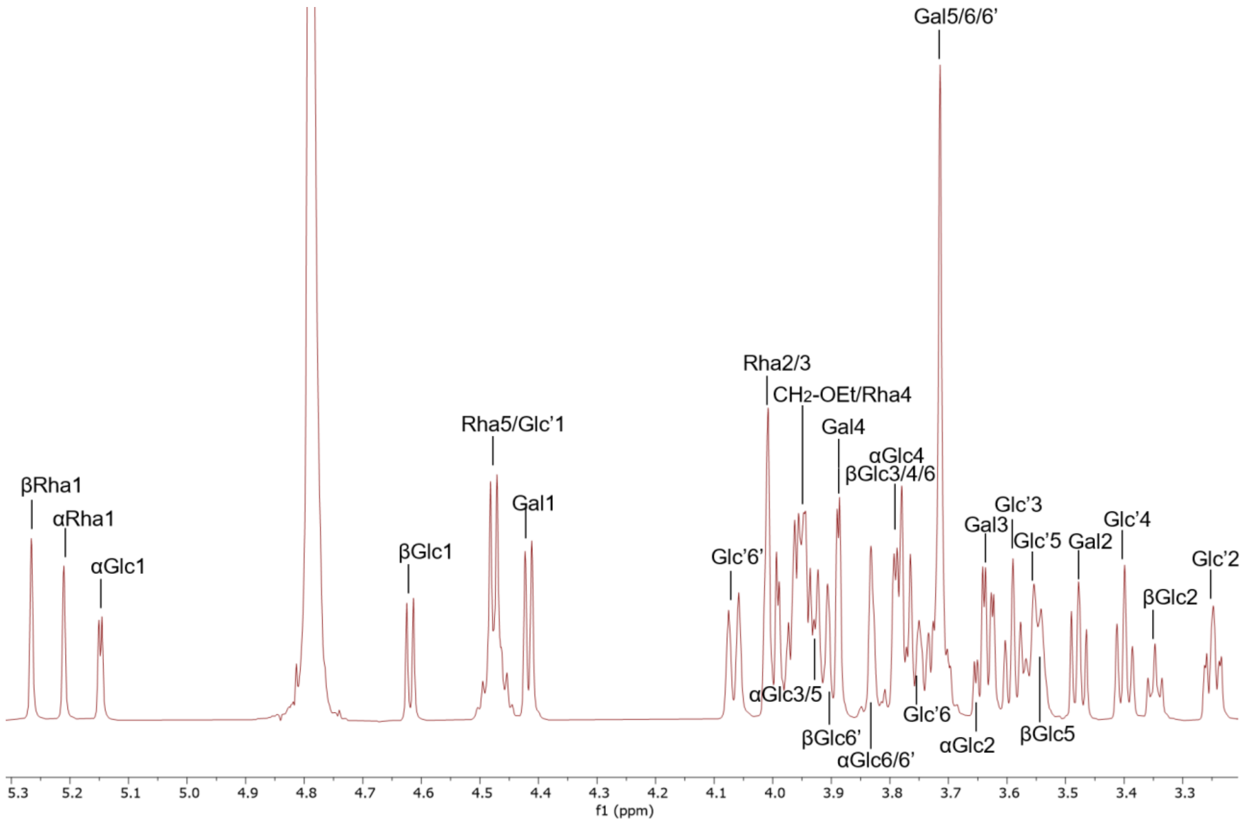

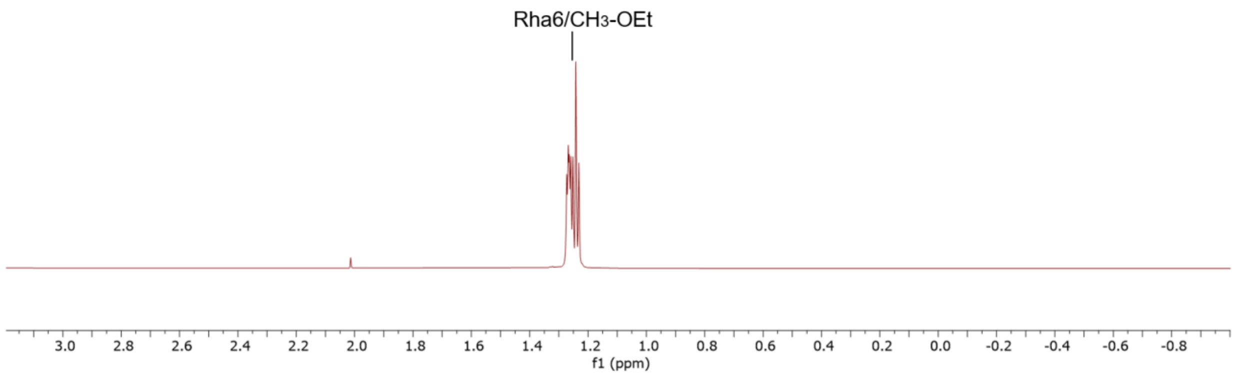

Figure S18 ^{1}H NMR of **4mer-II** (700 MHz, D_2O) with assignments.

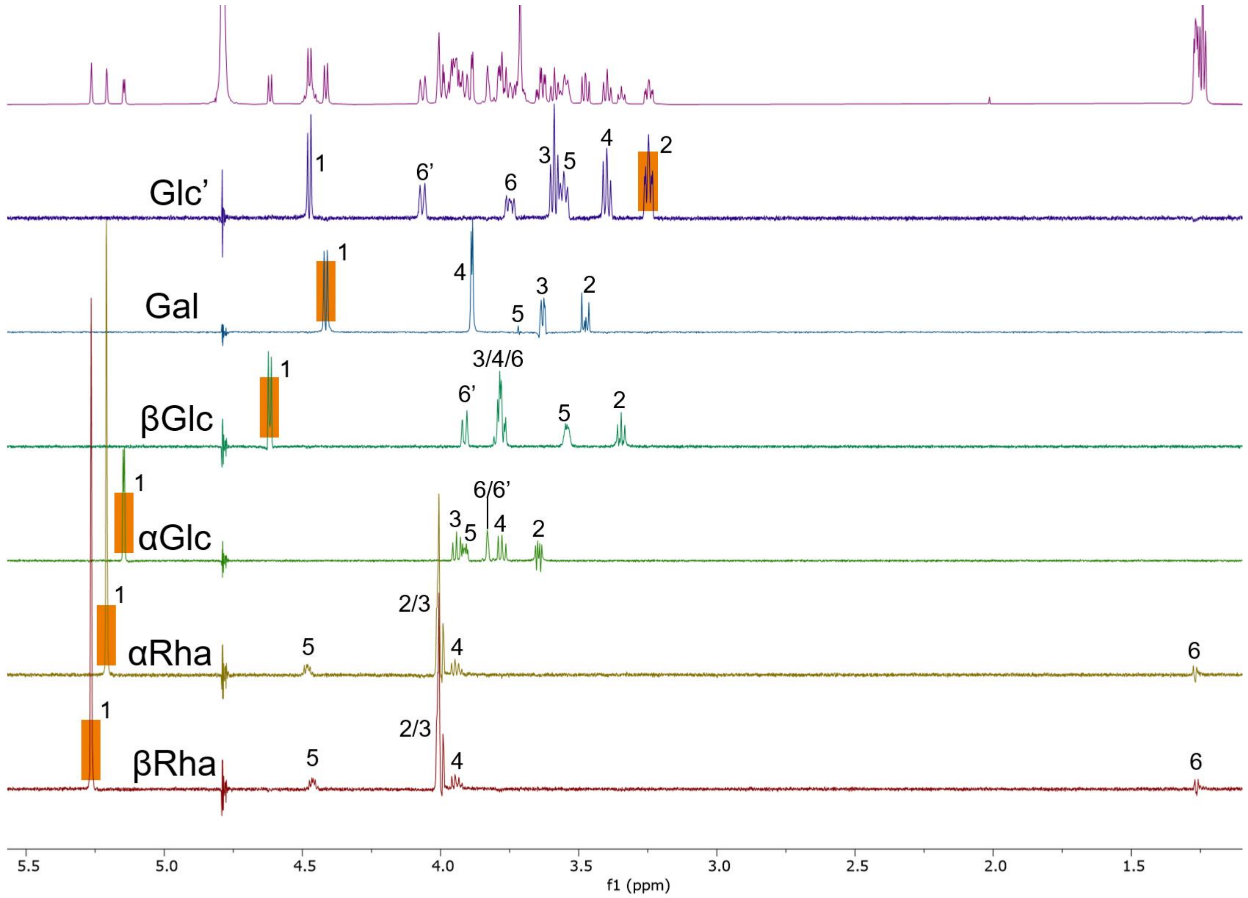

Figure S19 1D TOCSY (700 MHz, d9 = 200 ms, 293 K, D_2O) of **4mer-II** with assignments. Resonances chosen for selective excitation are highlighted in orange.

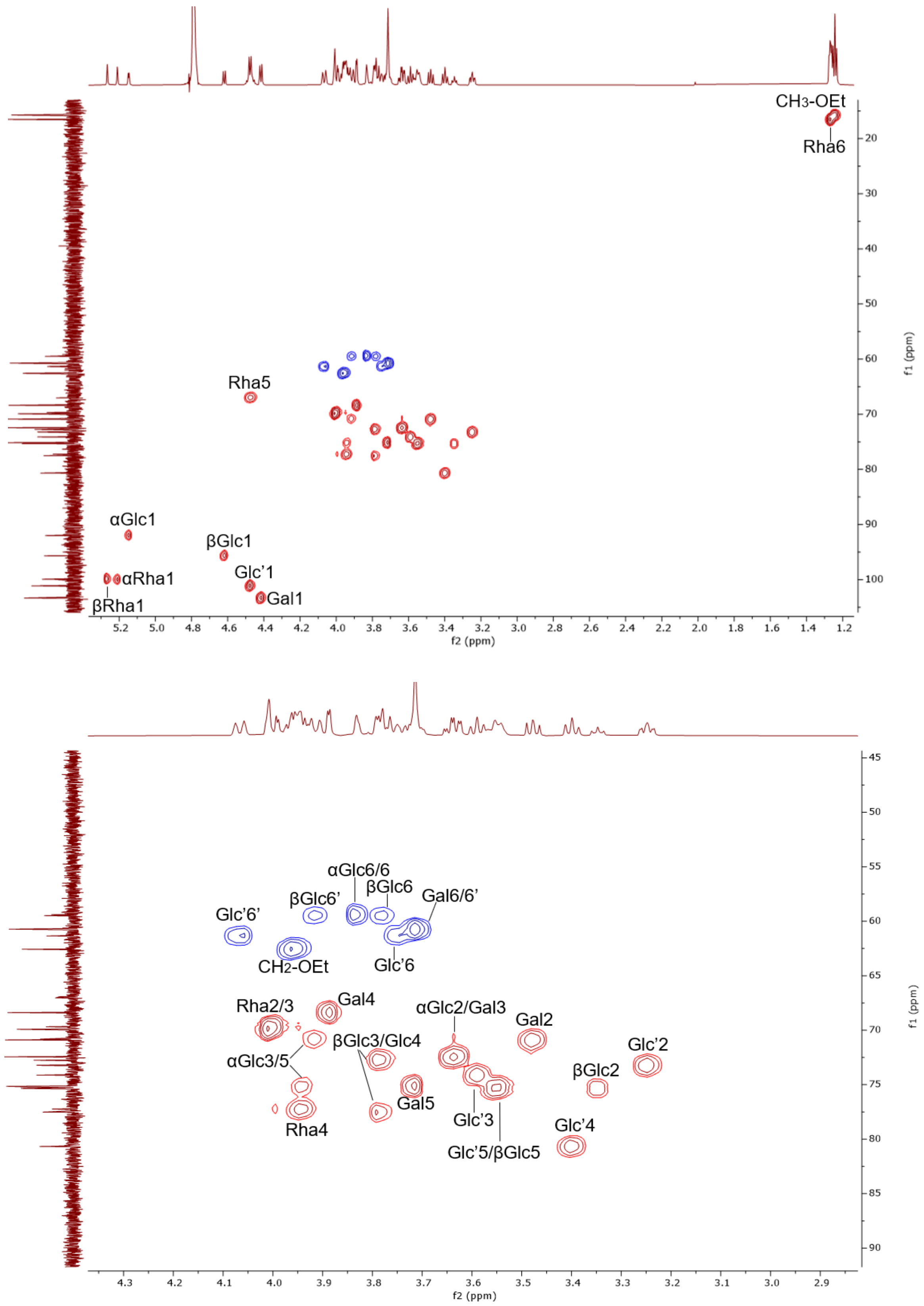

Figure S20 HSQC spectrum (D_2O) of **4mer-II** with assignments.

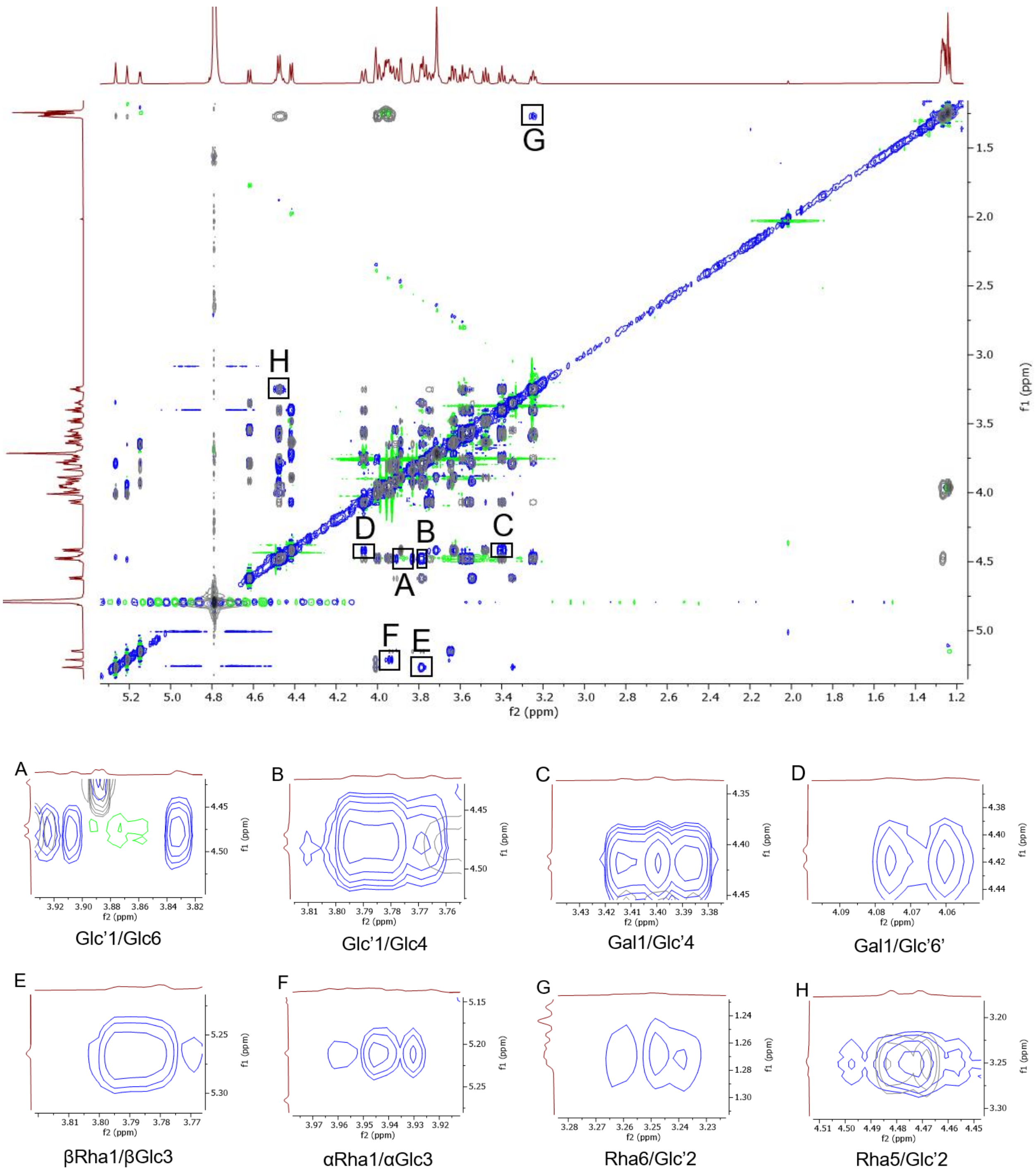

Figure S21 Overimposed 2D NOESY (green-blue, 700 MHz, d8 = 1000 ms, 293 K, D_2O) of **4mer-II** with assignments and 2D TOCSY spectrum (gray, 700 MHz, d9 = 80 ms, 293 K, D_2O).

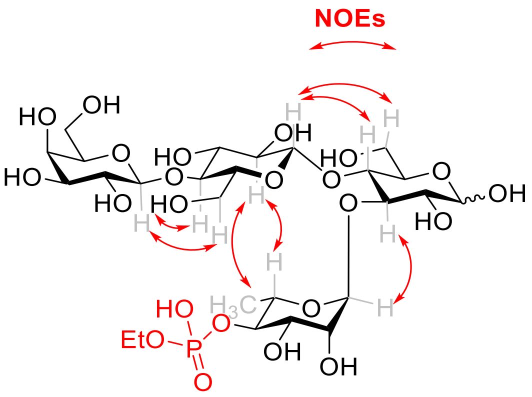

Figure S22 All experimentally observed NOEs (red arrows).

4.4.4 NMR analysis of 4mer-III

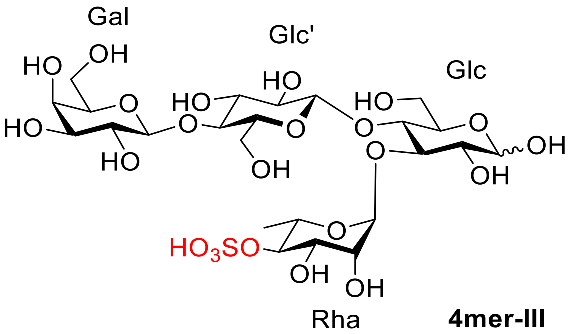

4mer-III

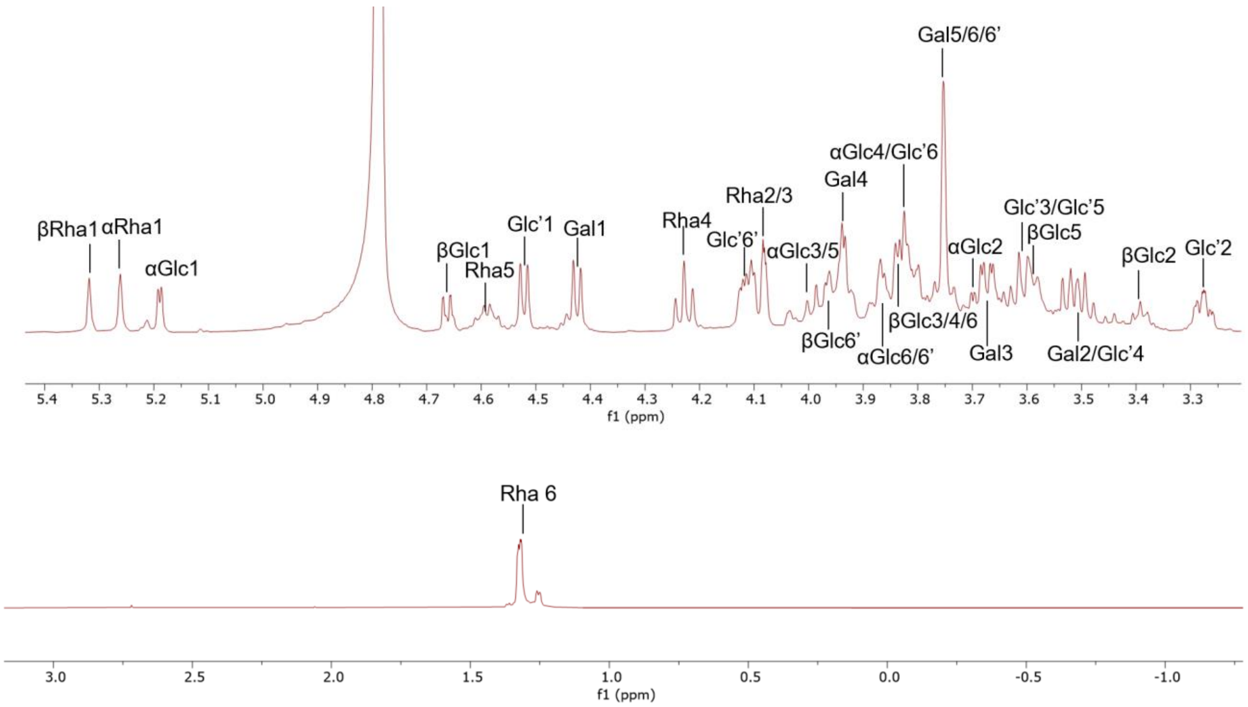

Figure S23 ^{1}H NMR of **4mer-III** (600 MHz, D_2O) with assignments.

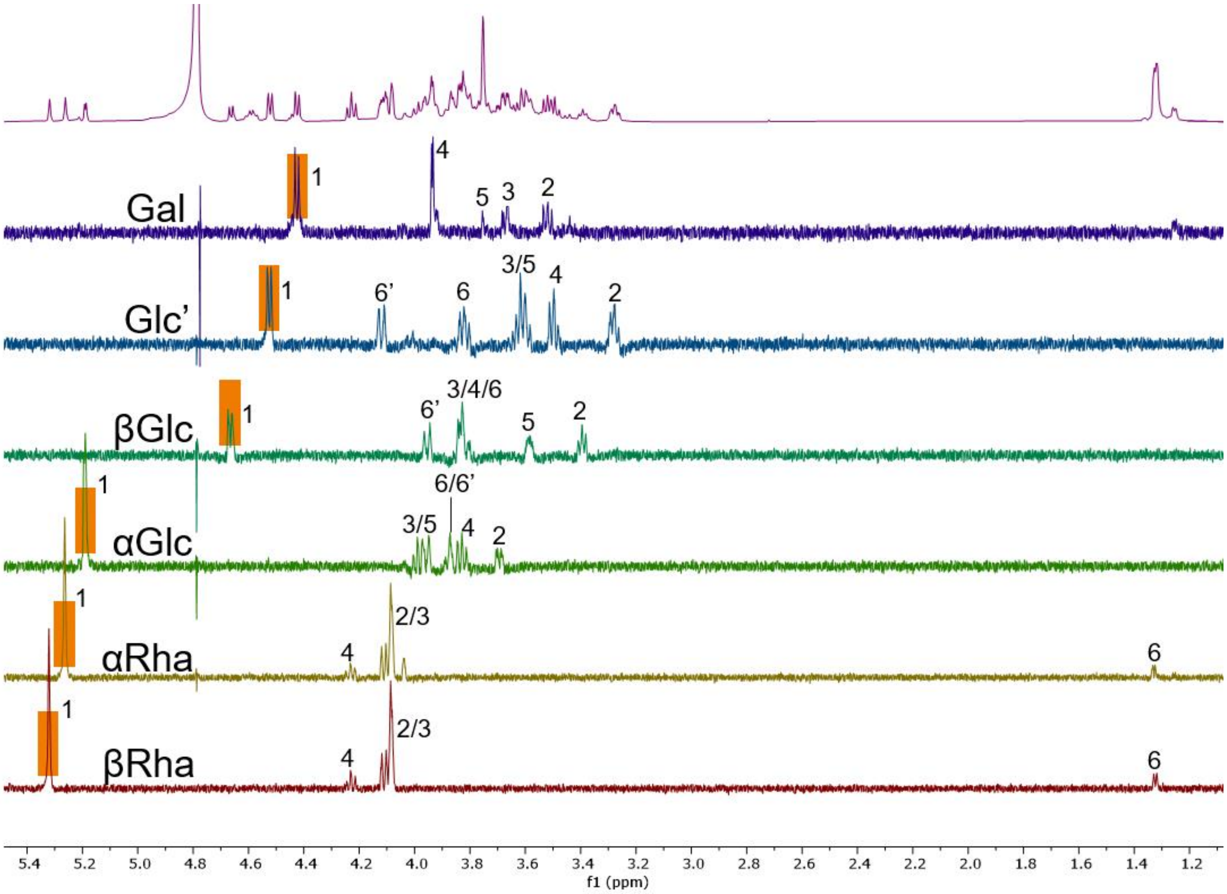

Figure S24 1D TOCSY (600 MHz, d9 = 350 ms (for Gal), 200 ms (for the other sugar), 293 K, D_2O) of **4mer-III** with assignments. Resonances chosen for selective excitation are highlighted in orange.

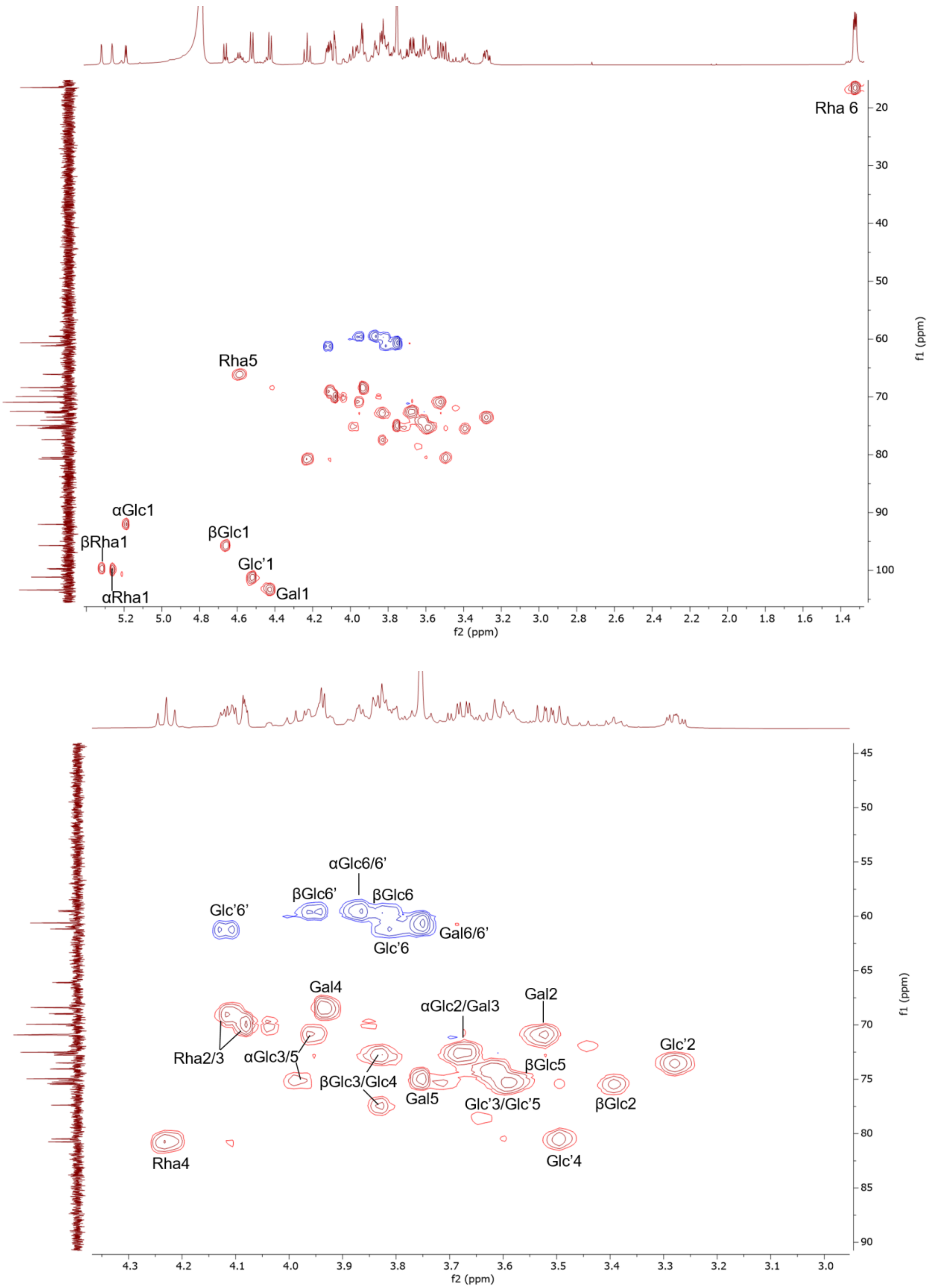

Figure S25 HSQC spectrum (D_2O) of **4mer-III** with assignments.

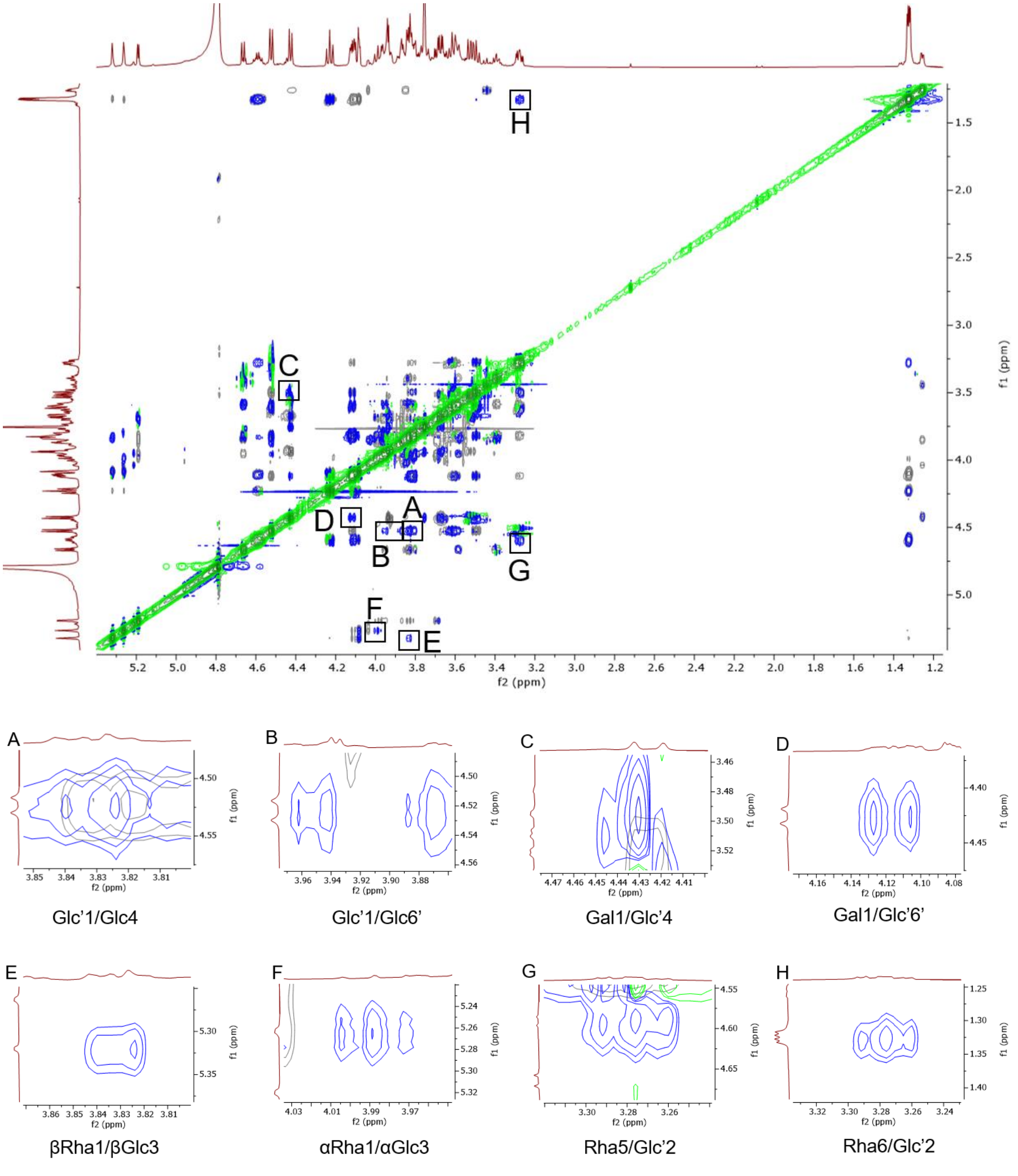

Figure S26 Overimposed 2D ROESY (green-blue, 600 MHz, P15 = 200 ms, 293 K, D_2O) of **4mer-III** with assignments and 2D TOCSY spectrum (gray, 600 MHz, d9 = 80 ms, 293 K, D_2O).

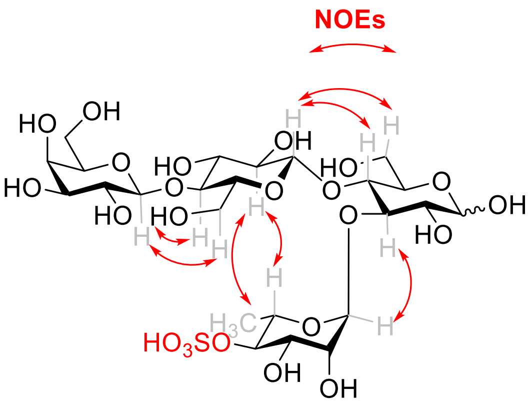

Figure S27 All experimentally observed NOEs (red arrows).

4.4.5 NMR analysis of 4mer-IV

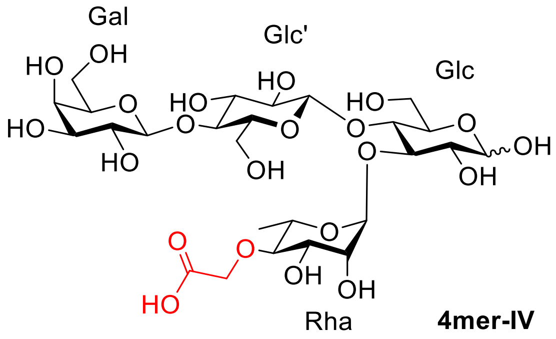

4mer-IV

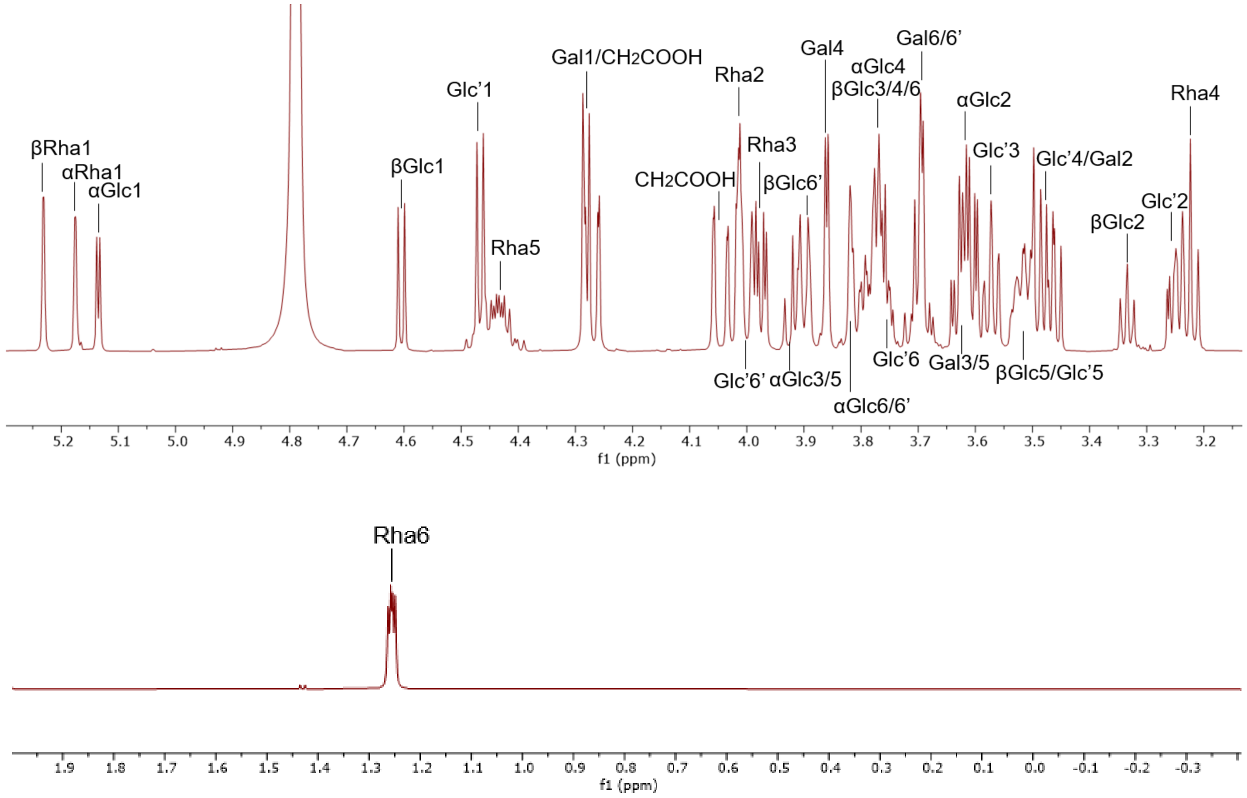

Figure S28 ^{1}H NMR of **4mer-IV** (700 MHz, D_2O) with assignments.

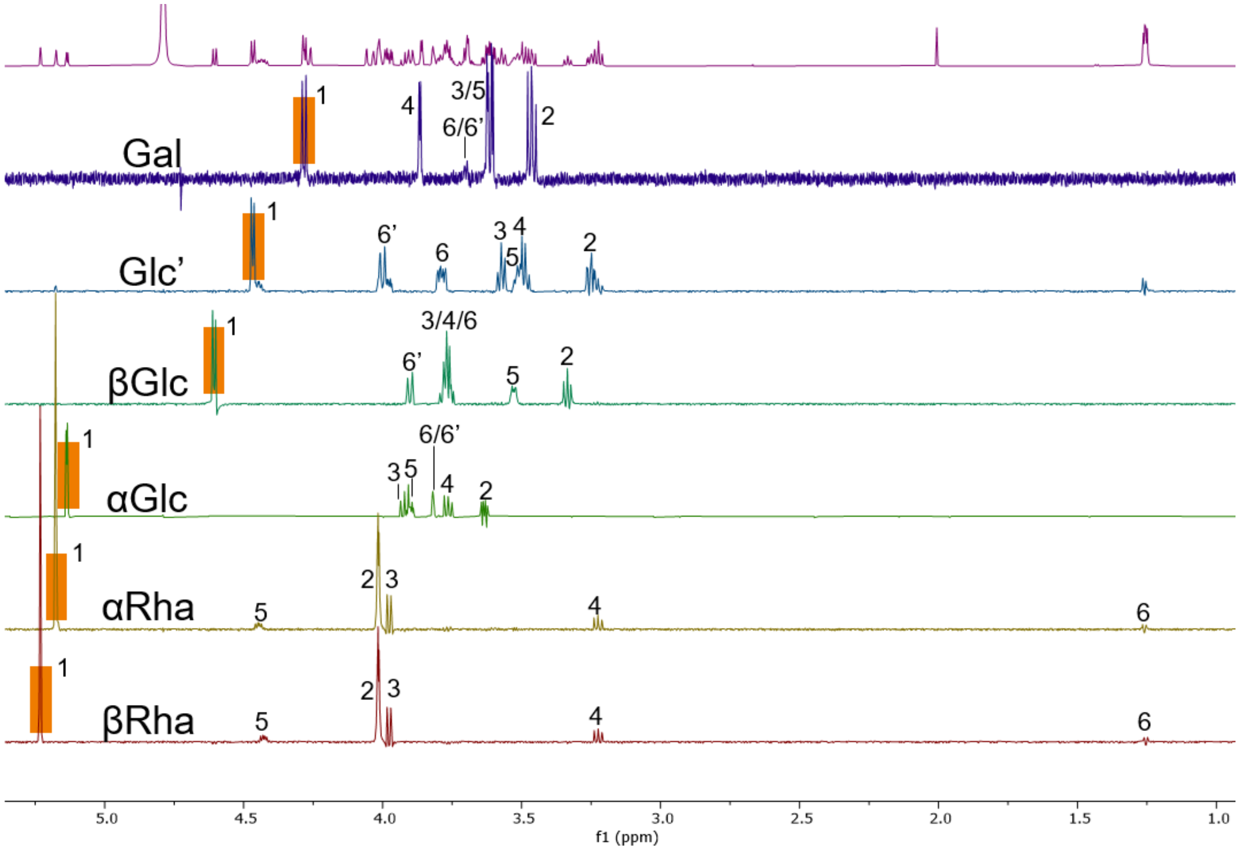

Figure S29 1D TOCSY (700 MHz, d9 = 450 ms (for Gal), 200 ms (for the other sugar), 293 K, D_2O) of **4mer-IV** with assignments. Resonances chosen for selective excitation are highlighted in orange.

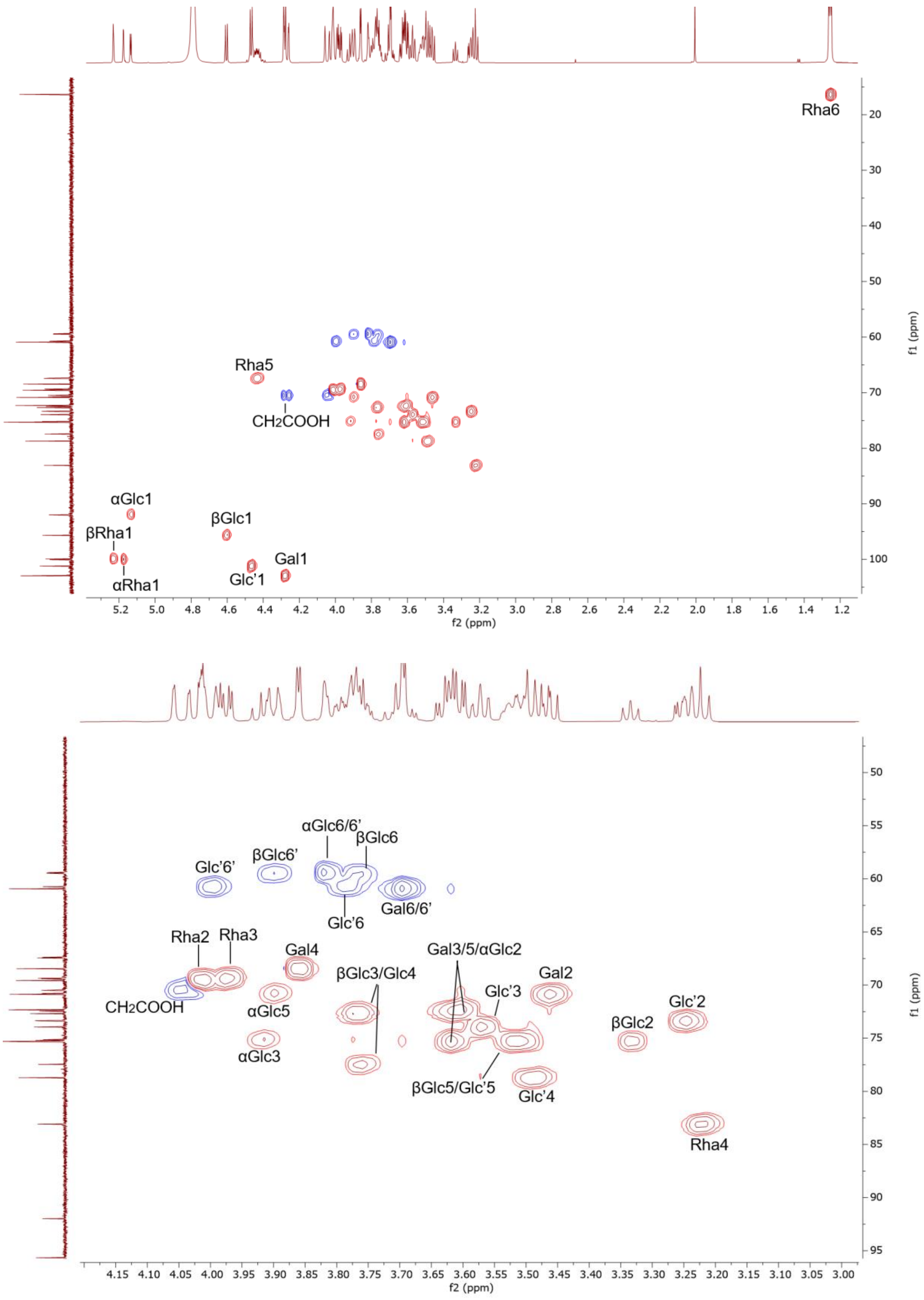

Figure S30 HSQC spectrum (D_2O) of **4mer-IV** with assignments.

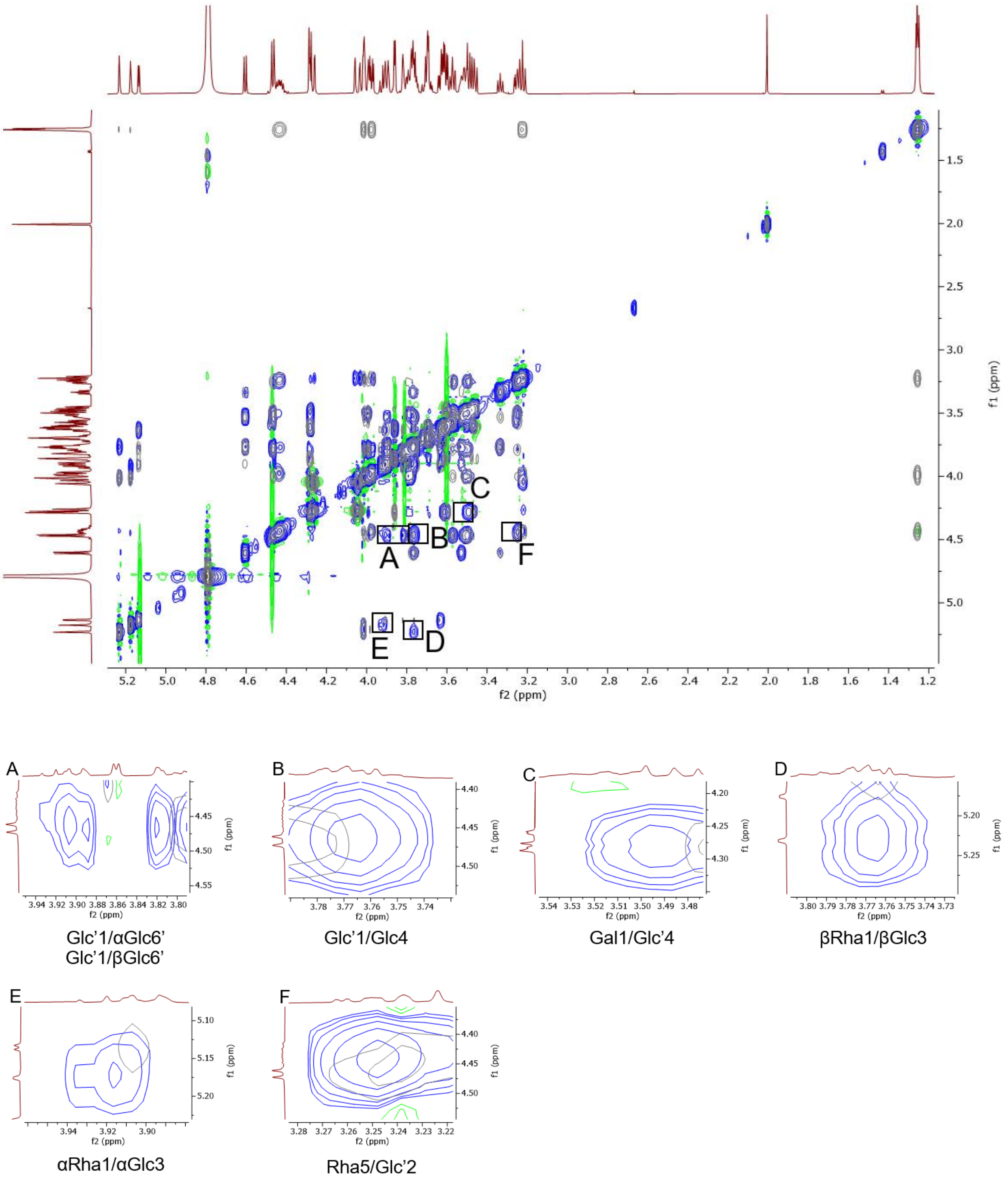

Figure S31 Overimposed 2D NOESY (green-blue, 700 MHz, d8 = 1000 ms, 293 K, D_2O) of **4mer-IV** with assignments and 2D TOCSY spectrum (gray, 700 MHz, d9 = 80 ms, 293 K, D_2O).

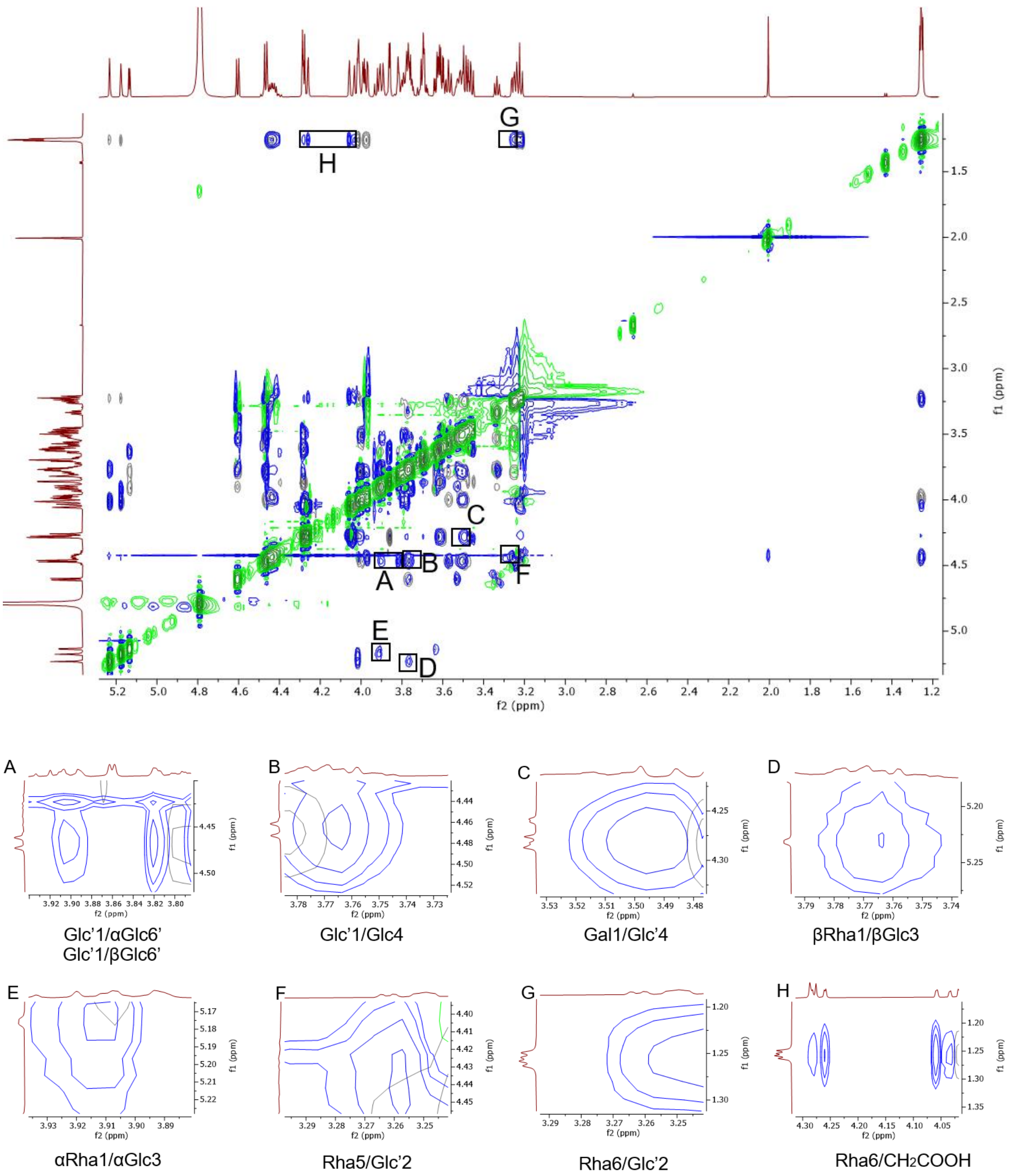

Figure S32 Overimposed 2D ROESY (green-blue, 700 MHz, P15 = 200 ms, 293 K, D_2O) of **4mer-IV** with assignments and 2D TOCSY spectrum (gray, 700 MHz, d9 = 80 ms, 293 K, D_2O).

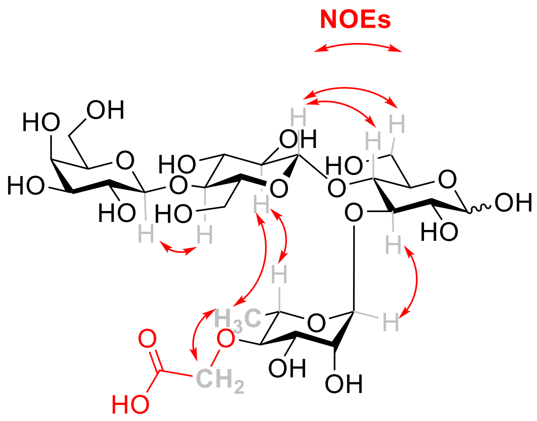

Figure S33 All experimentally observed NOEs (red arrows).

4.4.6 NMR analysis of 4mer-V

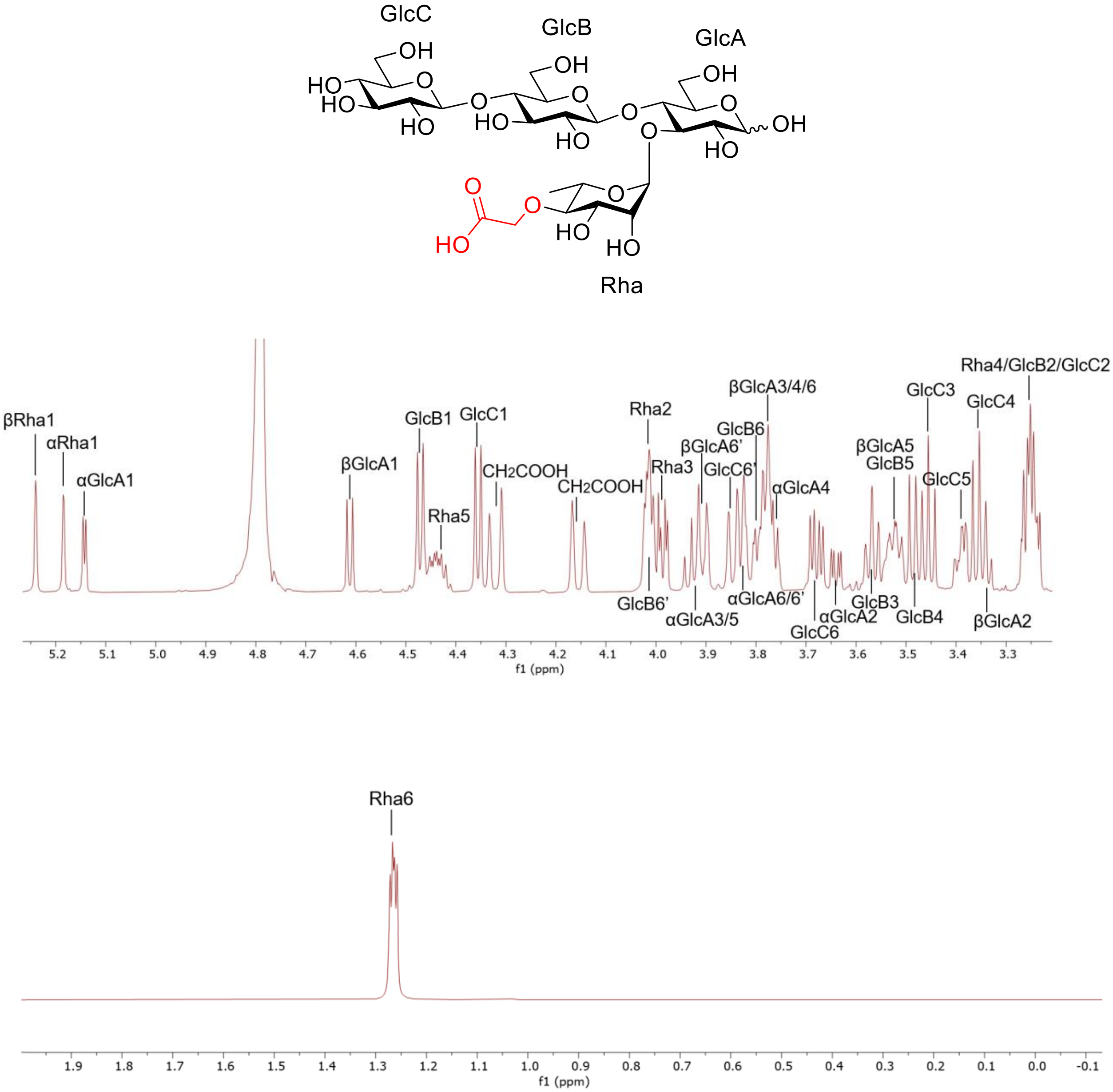

Figure S34 ^{1}H NMR of **4mer-V** (700 MHz, D_2O) with assignments.

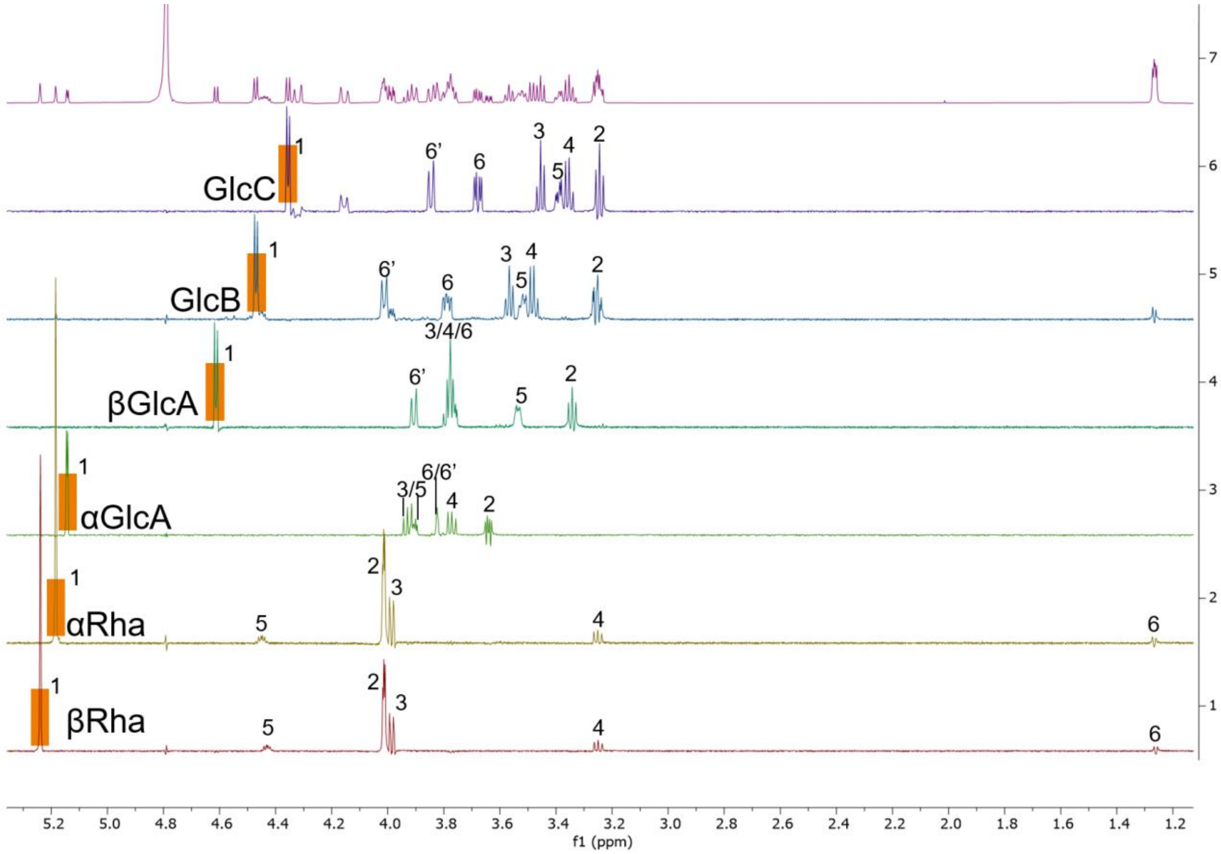

Figure S35 1D TOCSY (700 MHz, 200 ms, 293 K, D_2O) of **4mer-V** with assignments. Resonances chosen for selective excitation are highlighted in orange.

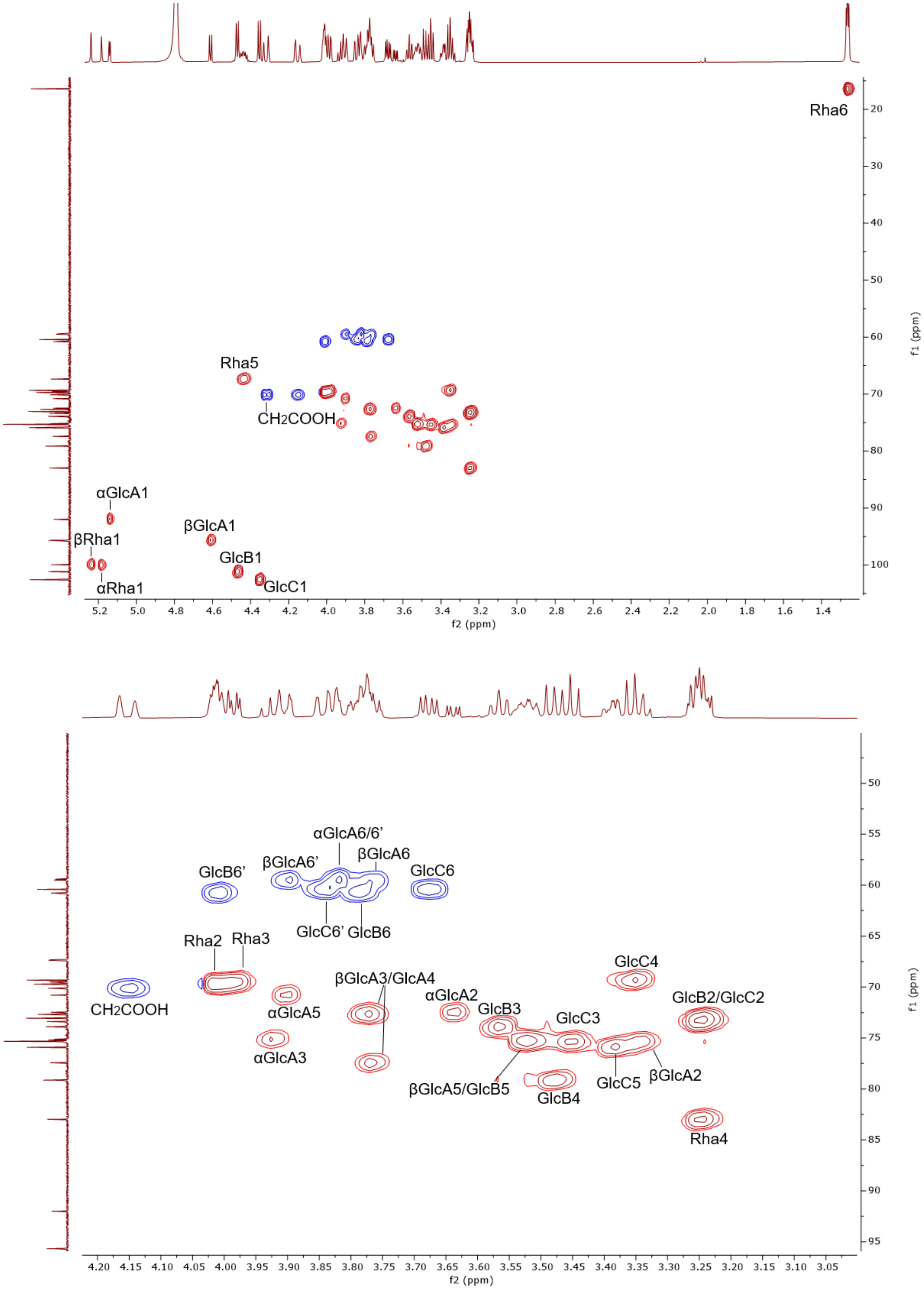

Figure S36 HSQC spectrum (D_2O) of **4mer-V** with assignments.

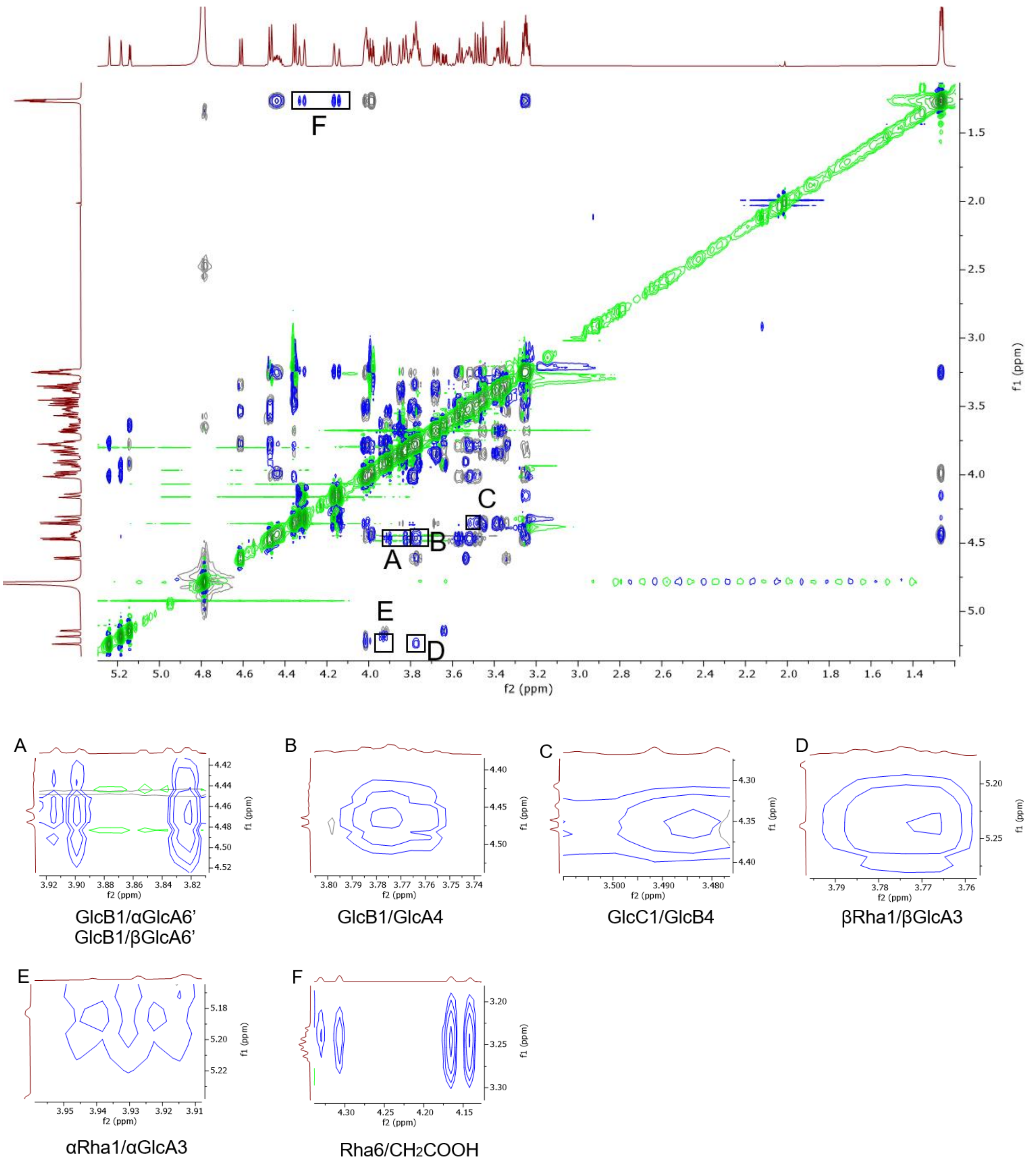

Figure S37 Overimposed 2D ROESY (green-blue, 700 MHz, P15 = 200 ms, 293 K, D_2O) of **4mer-V** with assignments and 2D TOCSY spectrum (gray, 700 MHz, d9 = 80 ms, 293 K, D_2O).

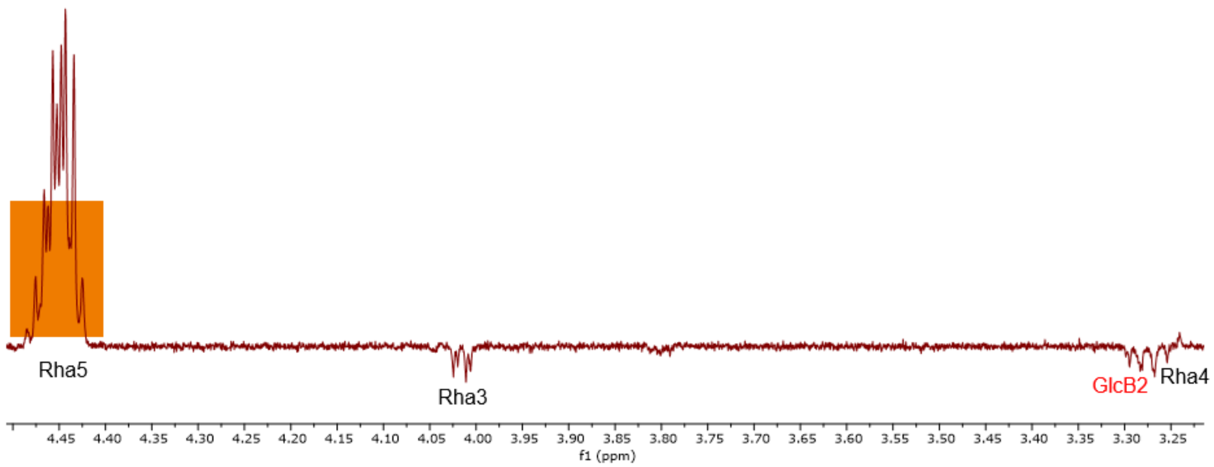

Figure S38 1D ROESY (700 MHz, 300 ms, 293 K, D_2O) of Rha5 to observe the spatial correlation between Rha5 and GlcB2. Resonances chosen for selective excitation are highlighted in orange.

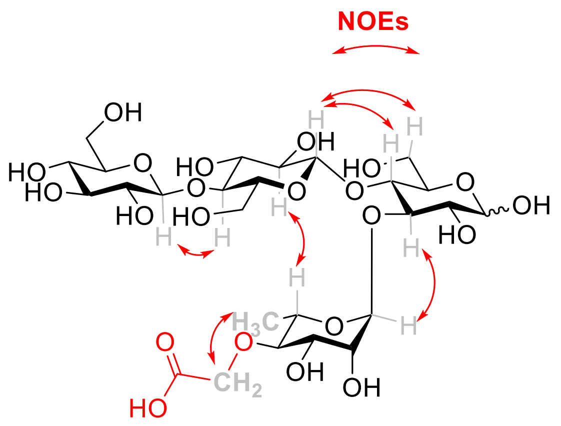

Figure S39 All experimentally observed NOEs (red arrows).

4.4.7 NMR analysis of 5mer

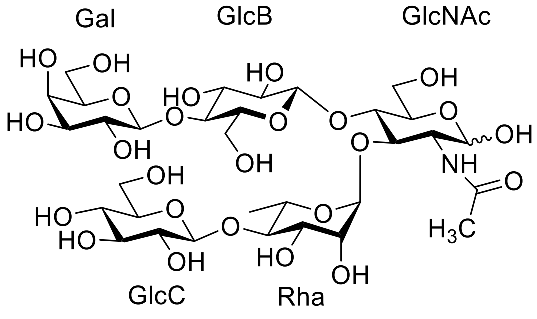

5mer

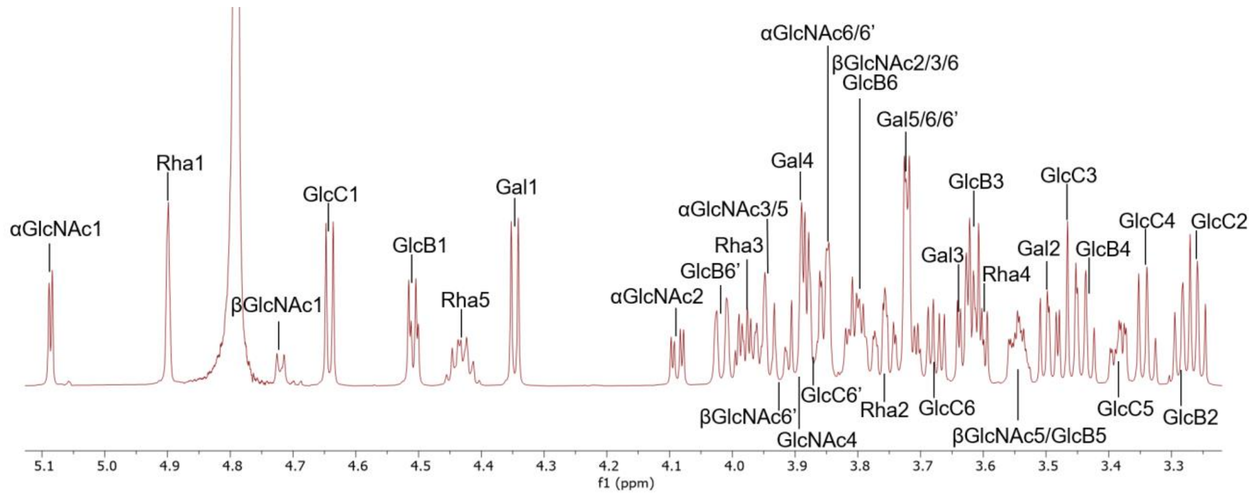

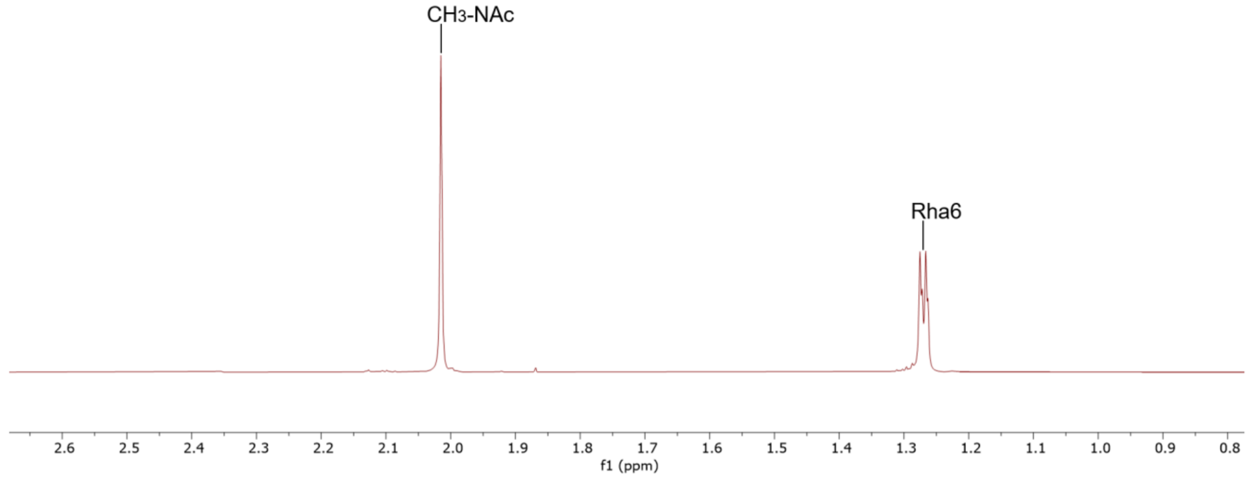

Figure S40 ^{1}H NMR of **5mer** (700 MHz, D_2O) with assignments.

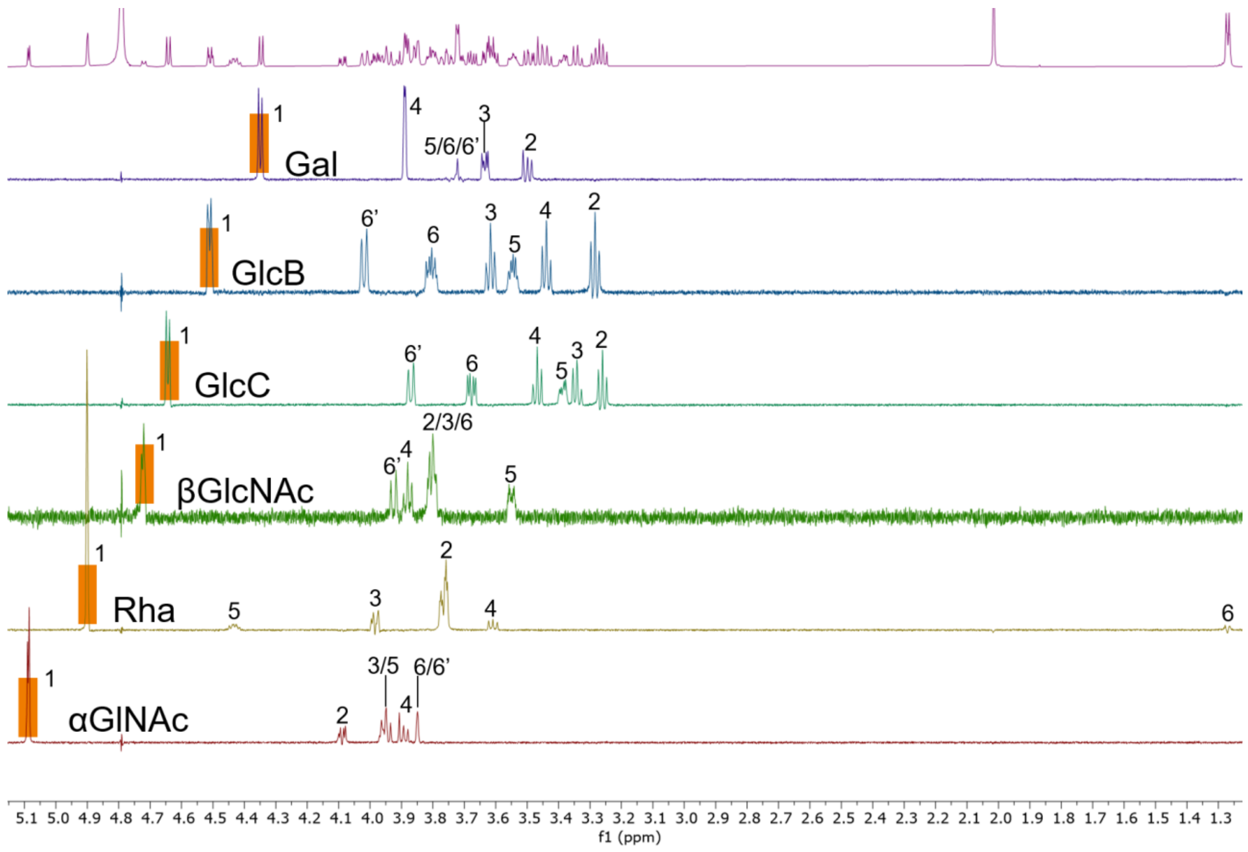

Figure S41 1D TOCSY (700 MHz, d9 = 350 ms (for Gal), 200 ms (for the other sugar), 293 K, D_2O) of **5mer** with assignments. Resonances chosen for selective excitation are highlighted in orange.

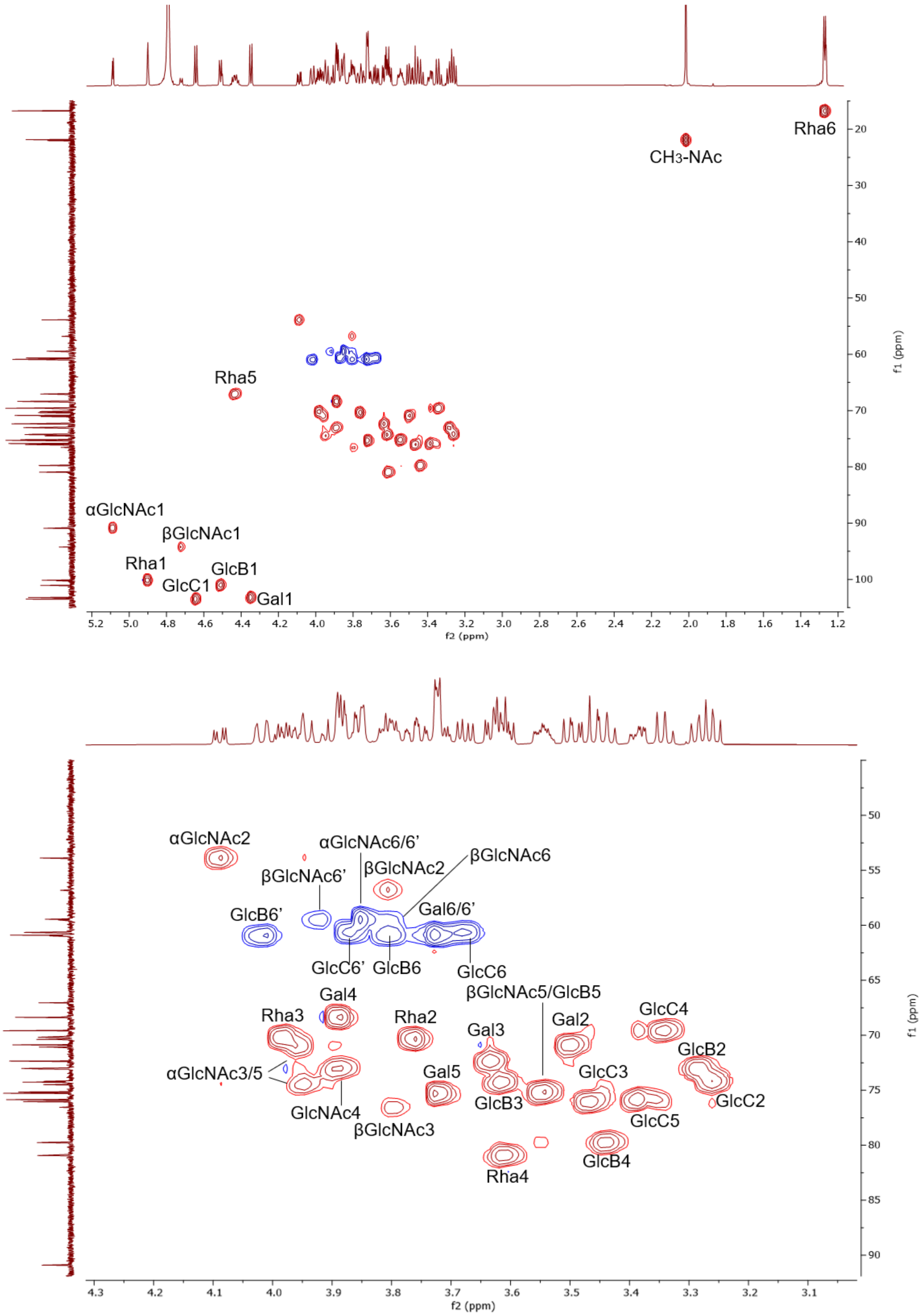

Figure S42 HSQC spectrum (D_2O) of **5mer** with assignments.

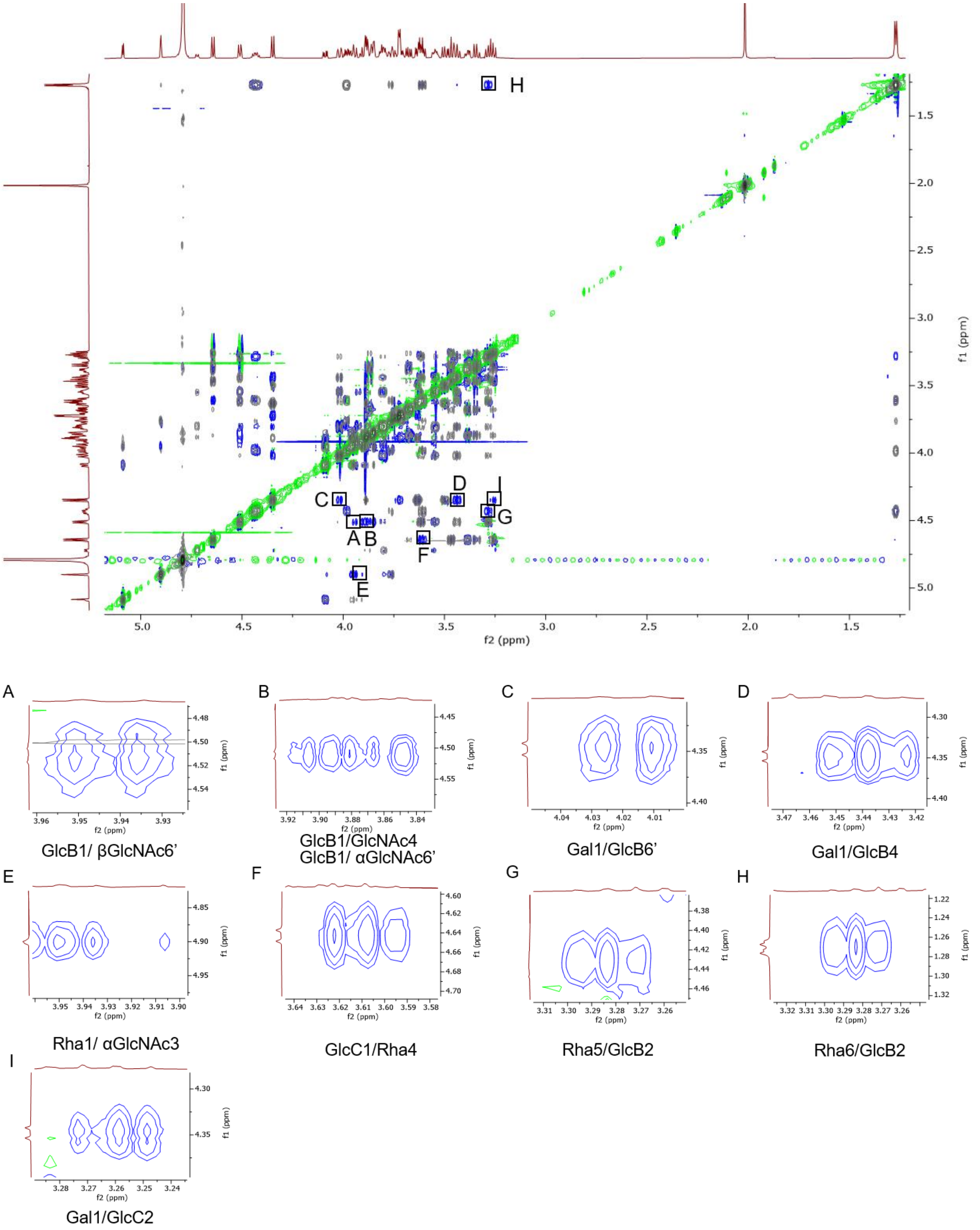

Figure S43 Overimposed 2D ROESY (green-blue, 700 MHz, P15 = 200 ms, 293 K, D_2O) of **5mer** with assignments and 2D TOCSY spectrum (gray, 700 MHz, d9 = 80 ms, 293 K, D_2O).

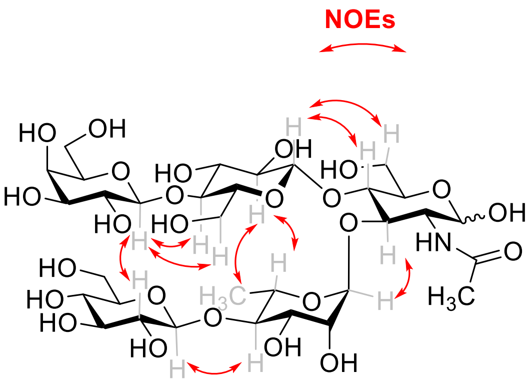

Figure S44 All experimentally observed NOEs (red arrows).

4.5 CH/π interactions analysis by NMR

4.5.1 General method

The CH/π interactions were observed by ^{1}H NMR and 1D TOCSY NMR by comparing the chemical shift changes of the glycan protons in the absence (control) and in the presence of tryptophan. A solution of **4mer-I – 4mer-V**, **5mer** in D_2O (3.5 mM or 2.4 mM) was prepared as control. A solution of **4mer-I – 4mer-V**, **5mer** in D_2O (3.5 mM or 2.4 mM) and L-tryptophan or 5OH-indole (10.5 mM or 7.5 mM) was prepared and incubated for 1 h. NMR spectra were recorded on a Varian 600-NMR (600 MHz) spectrometer. Selective 1D TOCSY (HOHAHA, pulse program: seldigpzs) spectra were recorded using different mixing times (d9 = 40, 350 ms).

4.5.2 CH/π interactions analysis: 4mer-I with L-tryptophan

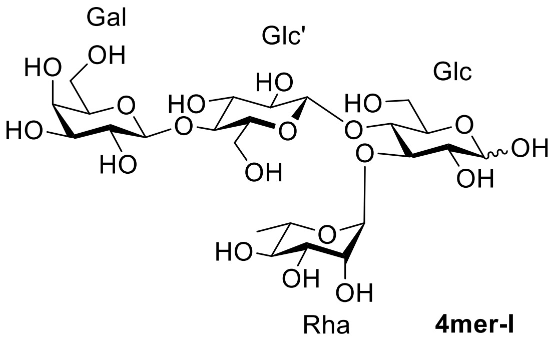

4mer-I

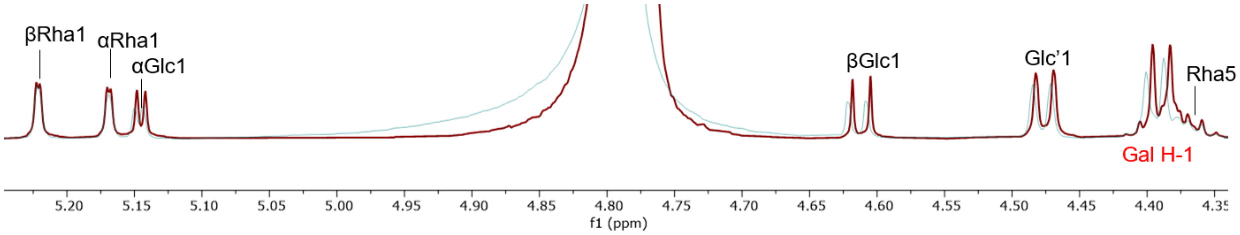

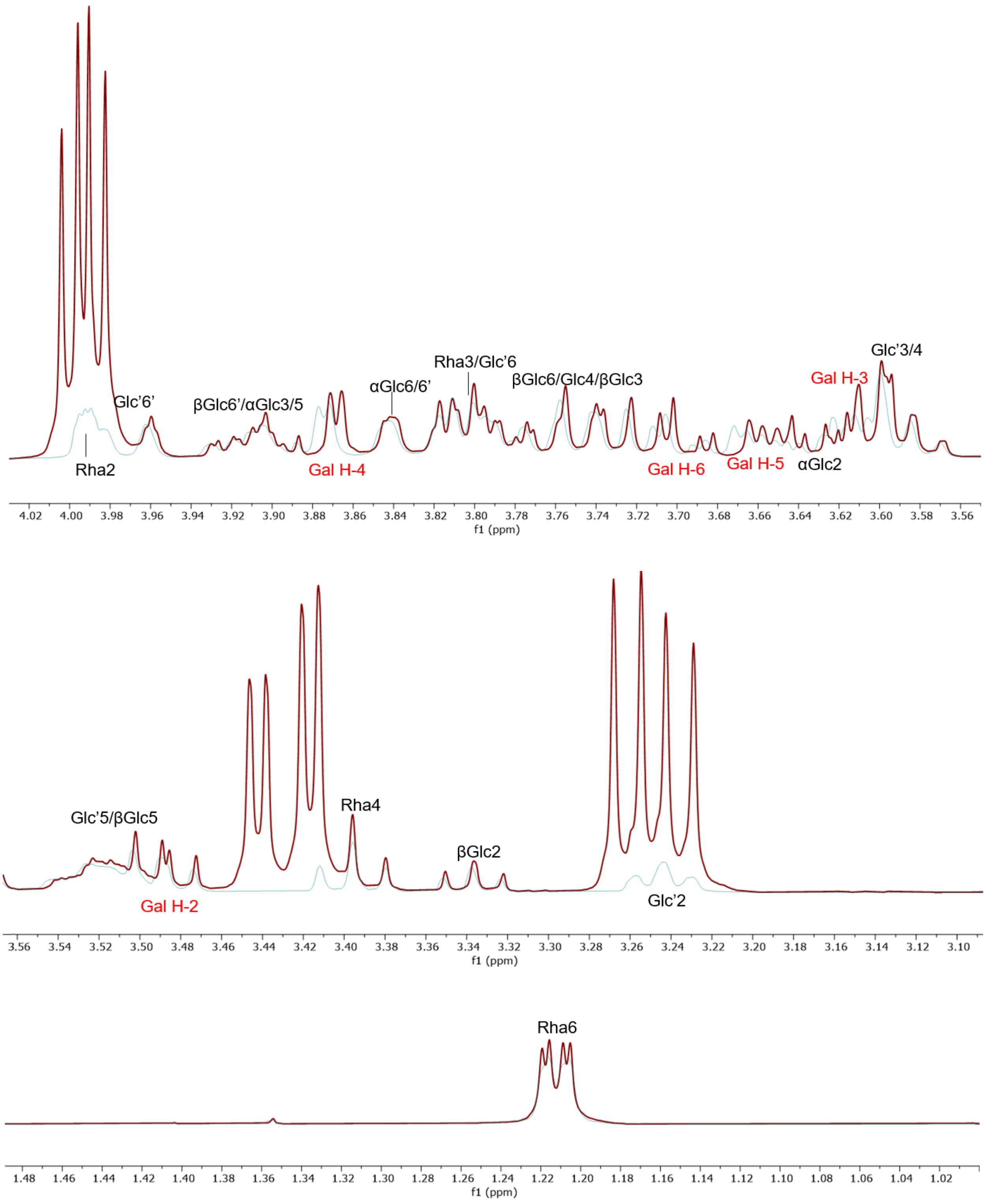

Figure S45 Overimposed ^{1}H NMR (600 MHz, D_2O) with assignments. The spectra are recorded for 3.5 mM **4mer-I** alone (green) or in the presence of 10.5 mM tryptophan (red).

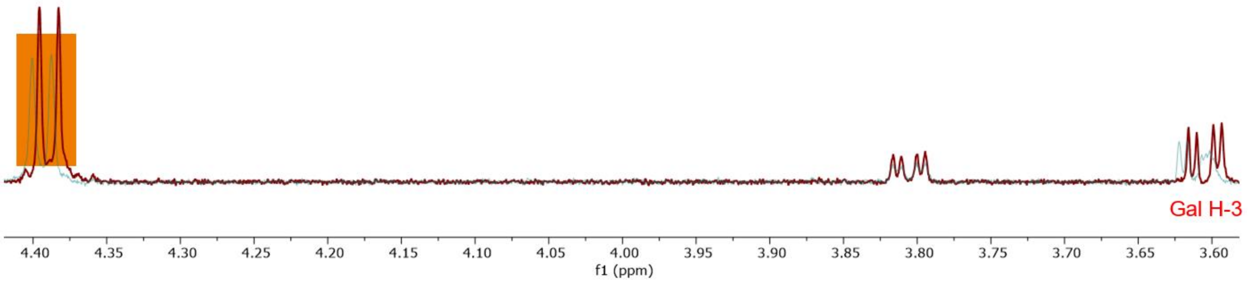

Figure S46 Overimposed 1D TOCSY (600 MHz, d9 = 40 ms, D_2O) to identify the chemical shift change of Gal-H3. The spectra are recorded for 3.5 mM **4mer-I** alone (green) or in the presence of 10.5 mM tryptophan (red). The resonance chosen for selective excitation is highlighted in orange.

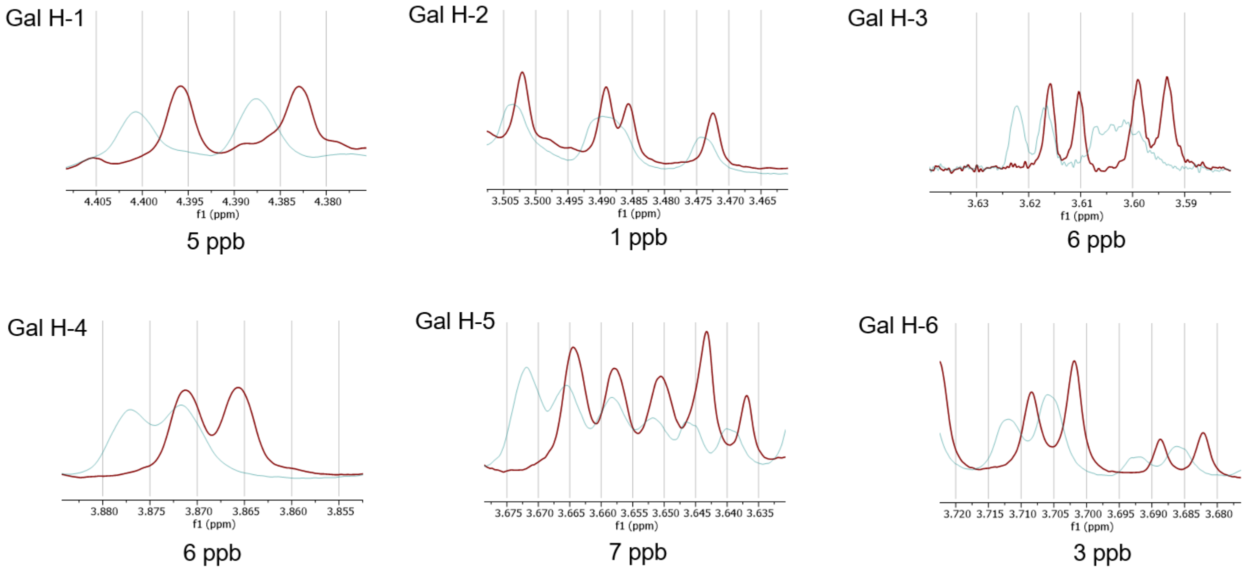

Figure S47 Excerpt of overimposed ^{1}H NMR and 1D TOCSY. The spectra are recorded for 3.5 mM **4mer-I** alone (green) or in the presence of 10.5 mM tryptophan (red).

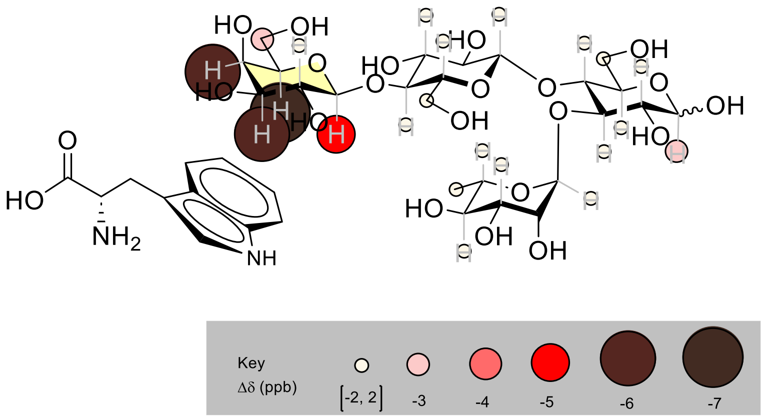

Figure S48 Experimentally observed chemical shift changes for **4mer-I** in the presence of Trp. CH/ π interactions are mainly focused on the Gal unit and most of the chemical shift changes detected for protons of the other monosaccharides are less than 2 ppb. Rha2 and Glc'2 overlapped with the side chain of tryptophan and therefore could not be analyzed.

4.5.3 CH/π interactions analysis: 4mer-I with 5OH-indole

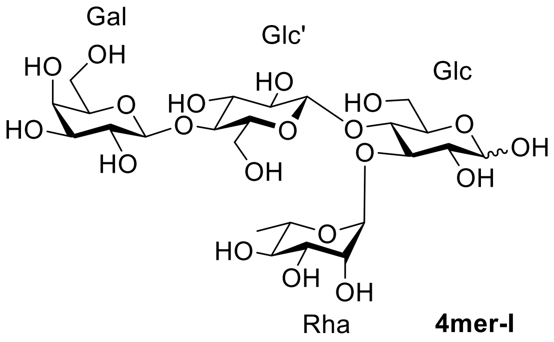

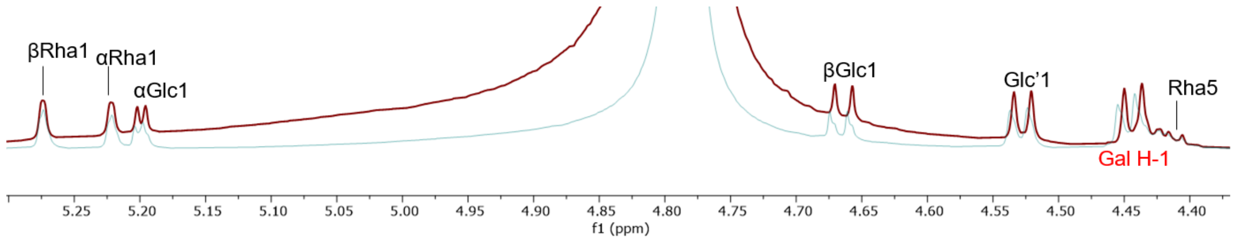

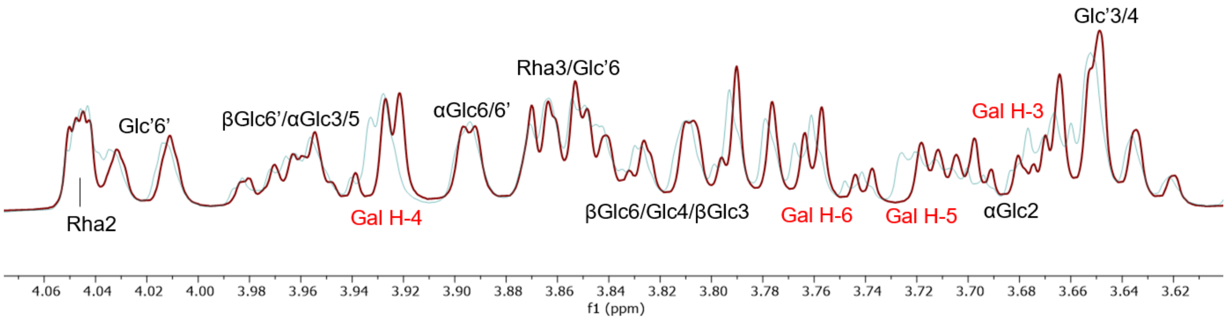

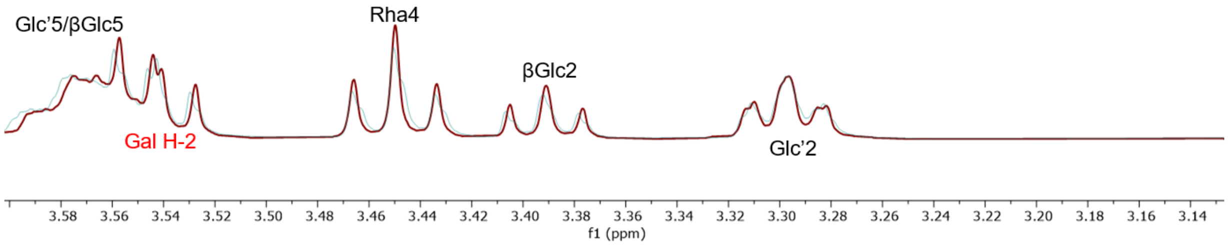

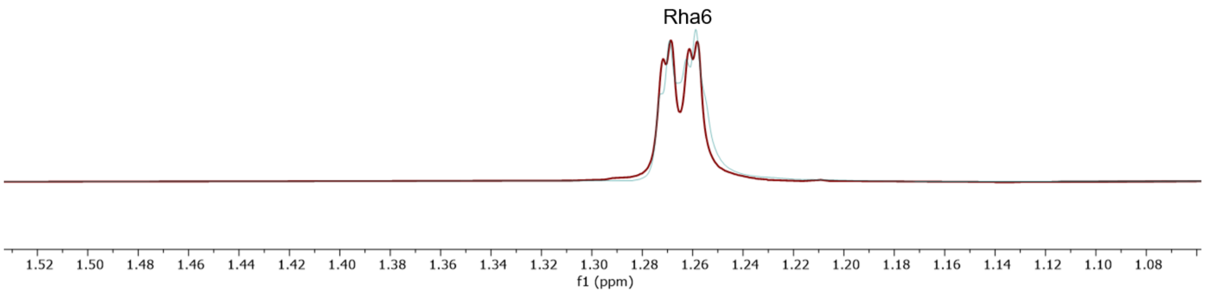

Figure S49 Overimposed ^{1}H NMR (600 MHz, D_2O) with assignments. The spectra are recorded for 2.4 mM **4mer-I** alone (green) or in the presence of 7.5 mM 5OH-indole (red).

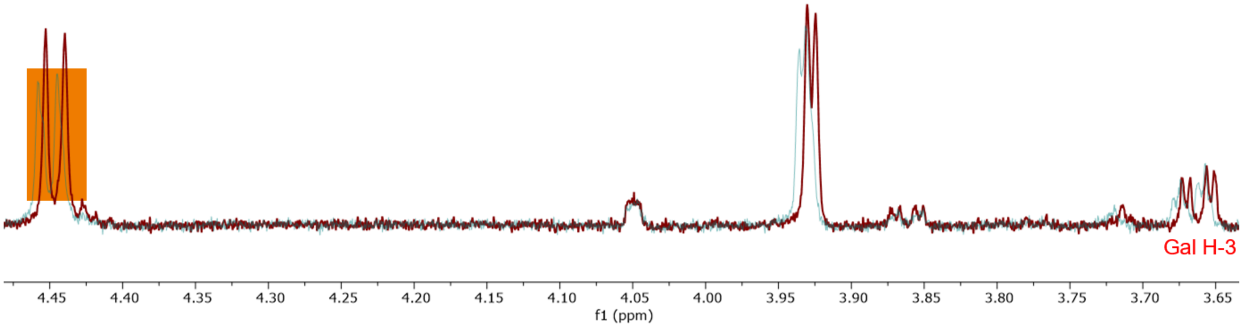

Figure S50 Overimposed 1D TOCSY (600 MHz, d9 = 350 ms, D_2O). The spectra are recorded for 2.4 mM **4mer-I** alone (green) or in the presence of 7.5 mM 5OH-indole (red). Resonances chosen with selective excitation are highlighted with orange to see the chemical shift change of Gal-H3.

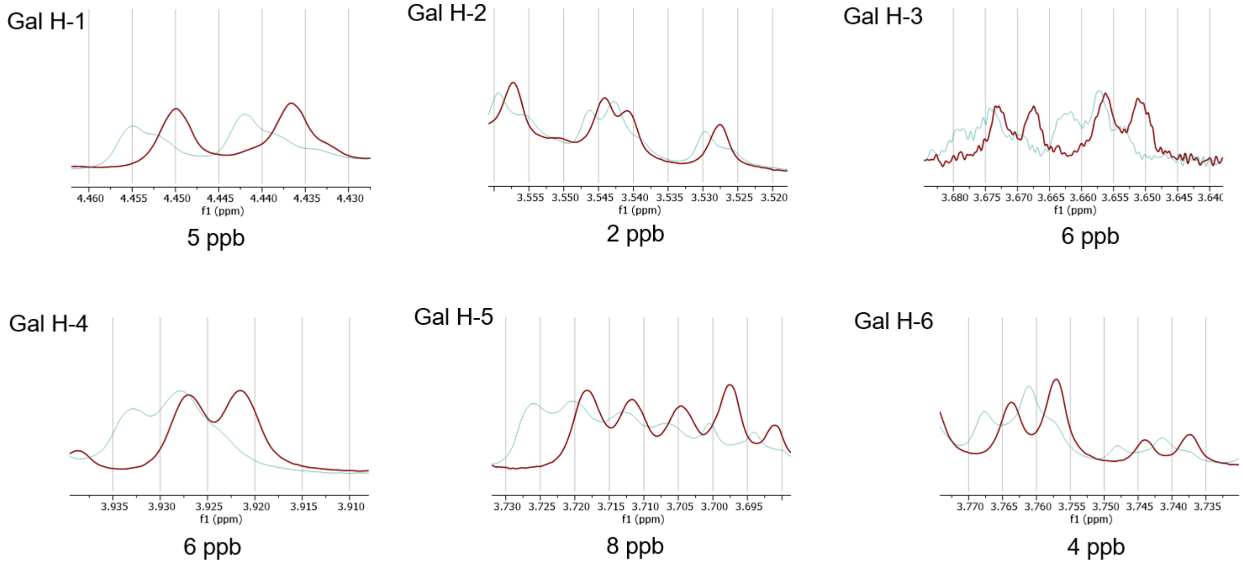

Figure S51 Excerpt of overimposed ^{1}H NMR and 1D TOCSY. The spectra are recorded for 2.4 mM **4mer-I** alone (green) or in the presence of 7.5 mM 5OH-indole (red).

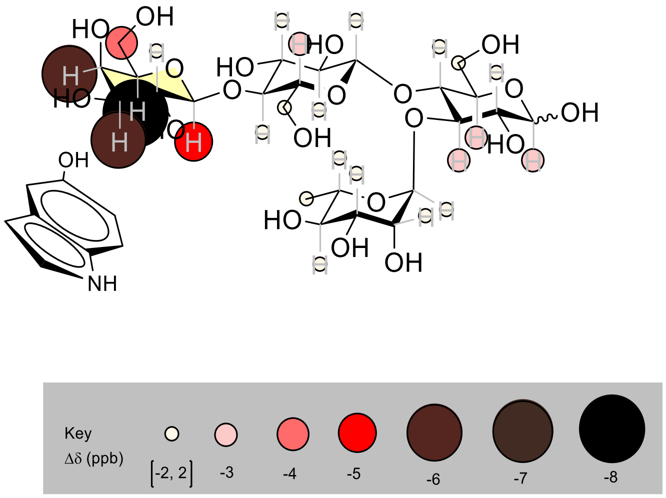

Figure S52 Experimentally observed chemical shift changes for **4mer-I** in the presence of 5OH-indole. As show in the figure, CH/ π interaction is mainly focused on the Gal unit and most of the chemical shift change of the protons in the other units are less than 3 ppb.

4.5.4 CH/π interactions analysis: 5mer with L-tryptophan

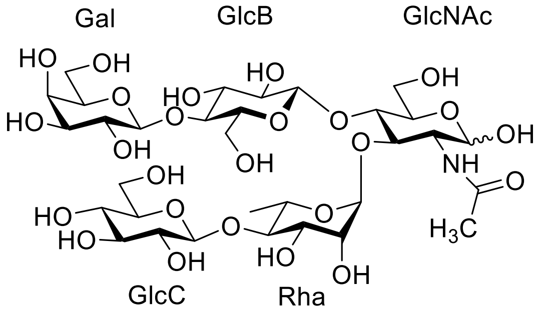

5mer

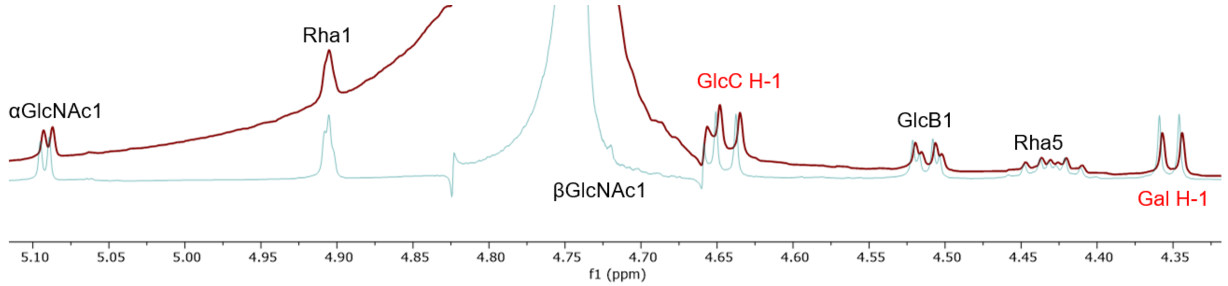

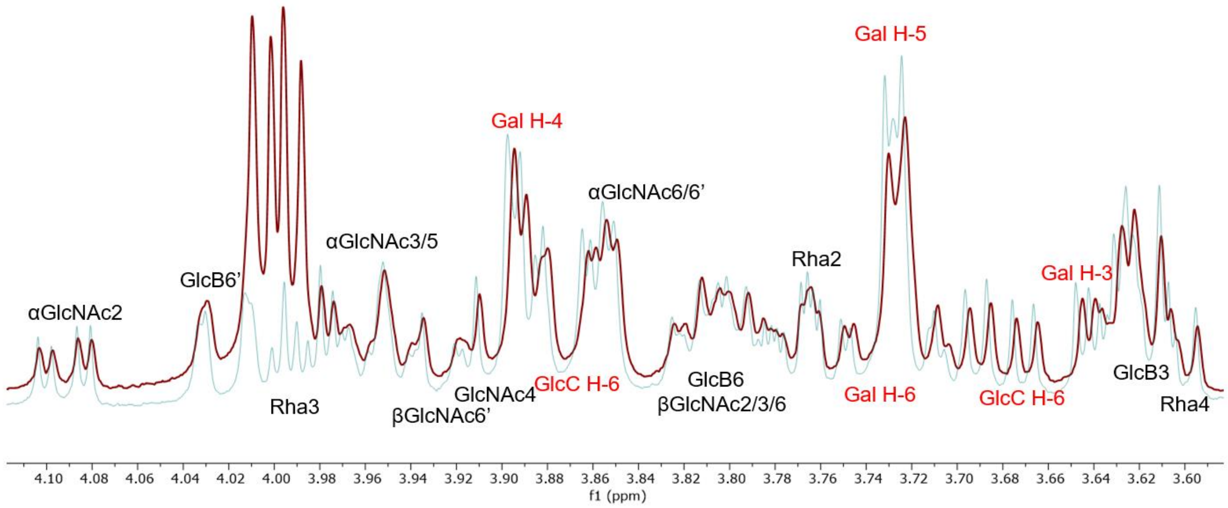

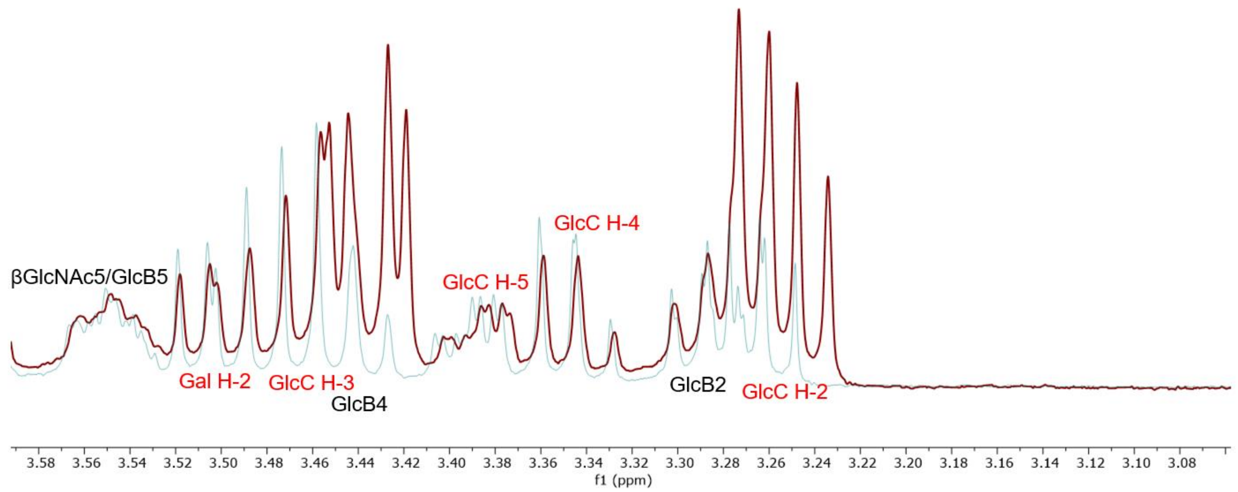

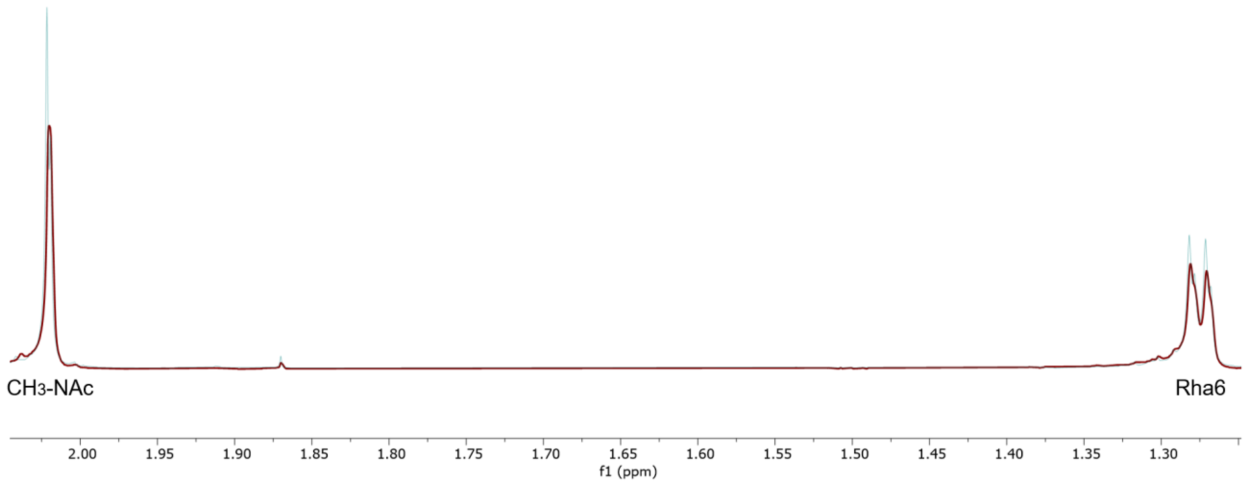

Figure S53 Overimposed ^{1}H NMR (600 MHz, D_2O) with assignments. The spectra are recorded for 3.5 mM **5mer** alone (green) or in the presence of 10.5 mM tryptophan (red).

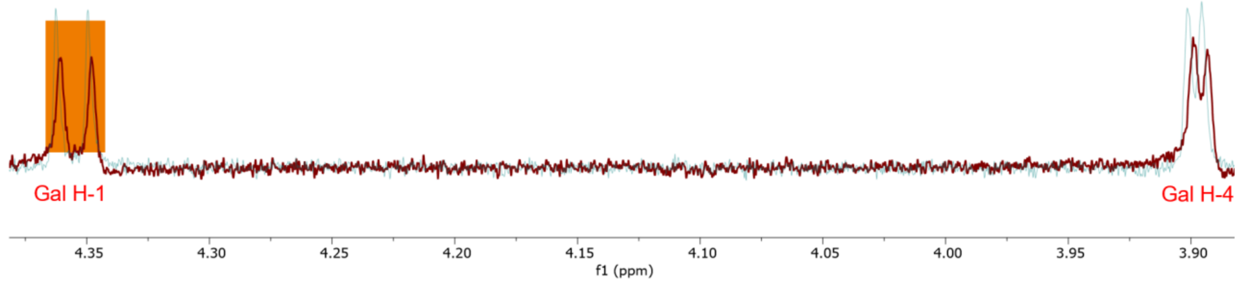

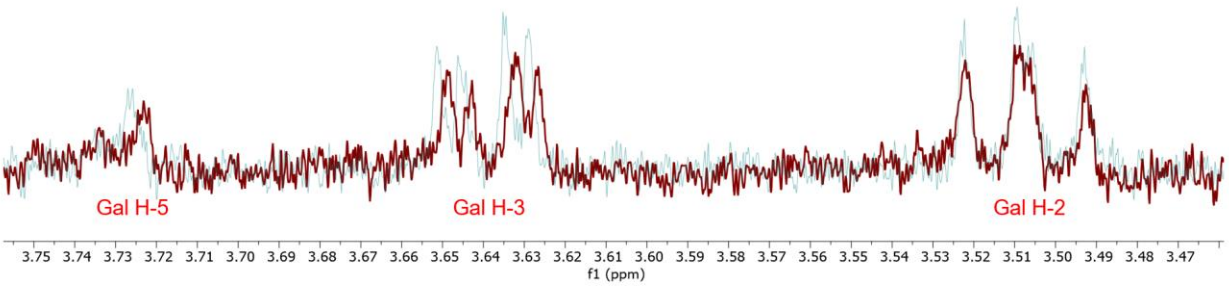

Figure S54 Overimposed 1D TOCSY (600 MHz, d9 = 350 ms, D_2O). The spectra are recorded for 3.5 mM **5mer** alone (green) or in the presence of 10.5 mM tryptophan (red). Resonances chosen with selective excitation are highlighted with orange to see the chemical shift change of Gal-H5.

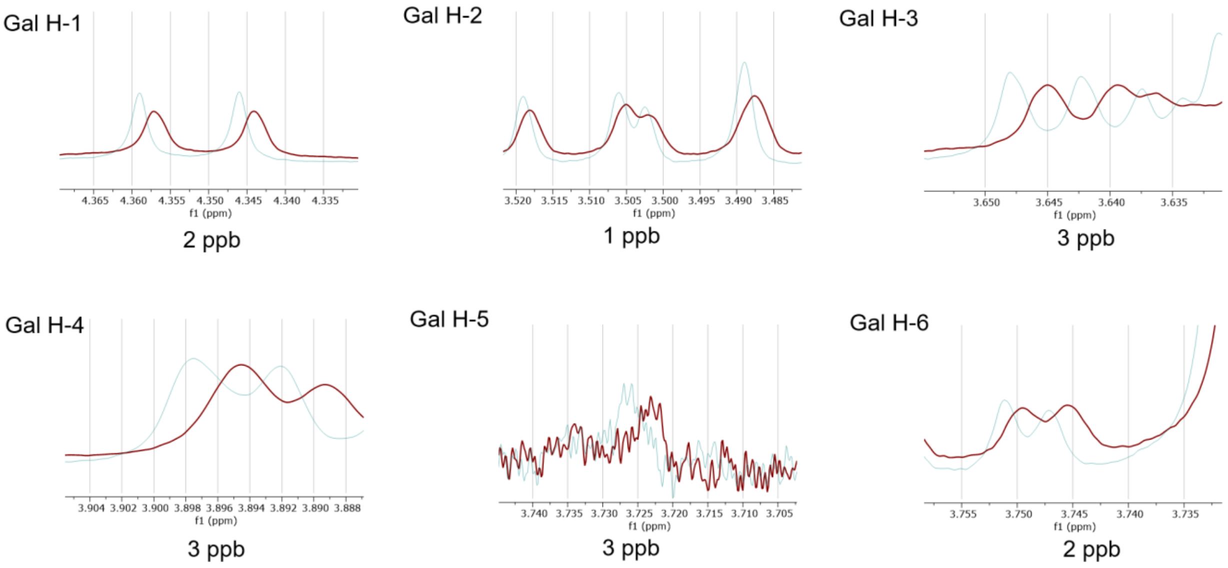

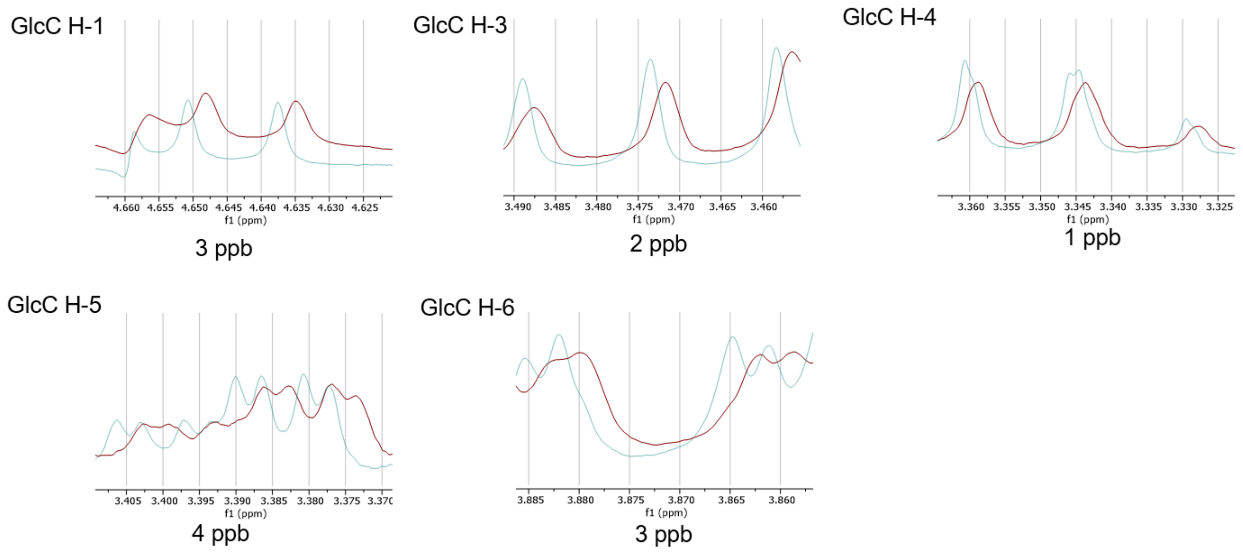

Figure S55 Excerpt of overimposed ^{1}H NMR and 1D TOCSY. The spectra are recorded for 3.5 mM **5mer** alone (green) or in the presence of 10.5 mM tryptophan (red).

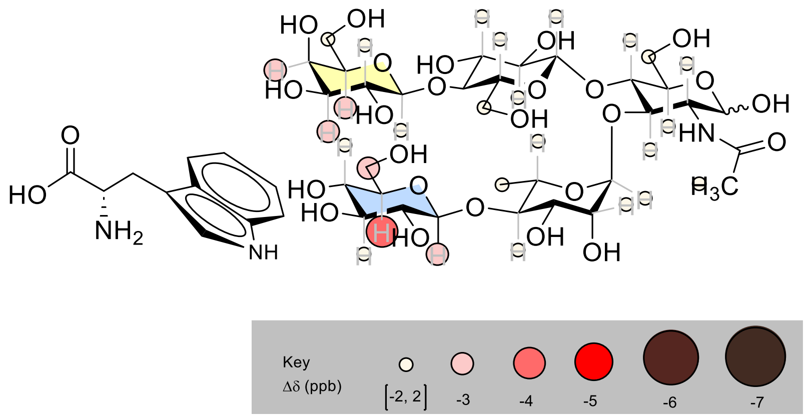

Figure S56 Experimentally observed chemical shift changes for **5mer** in the presence of Trp. βGlcNAc1, Rha3, GlcB4 and GlcC2 overlapped with the water peak and the tryptophan side chain respectively and therefore could not be analyzed.

4.5.5 CH/π interactions analysis: 5mer with 5OH-indole

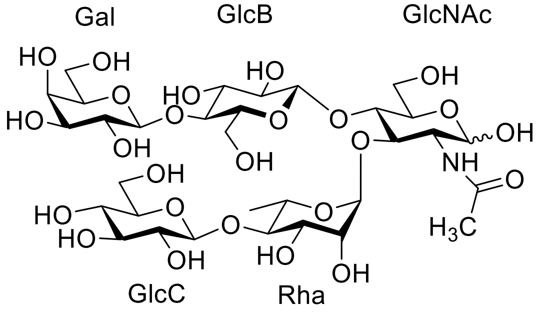

5mer

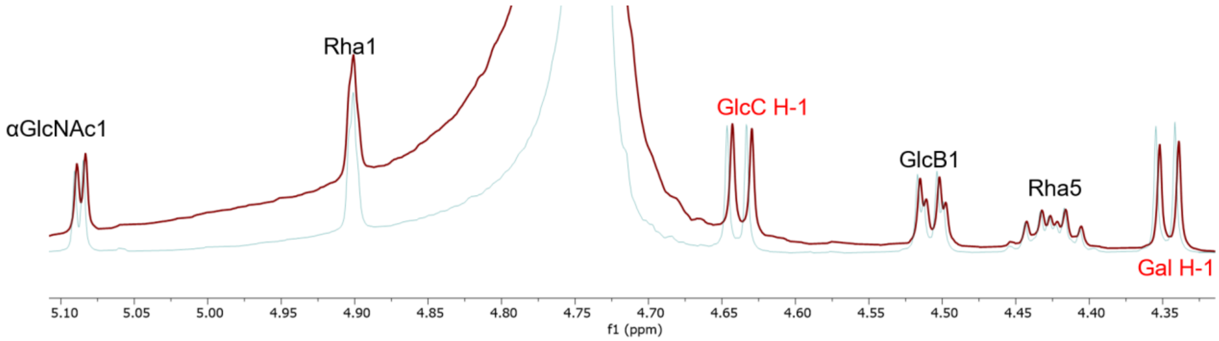

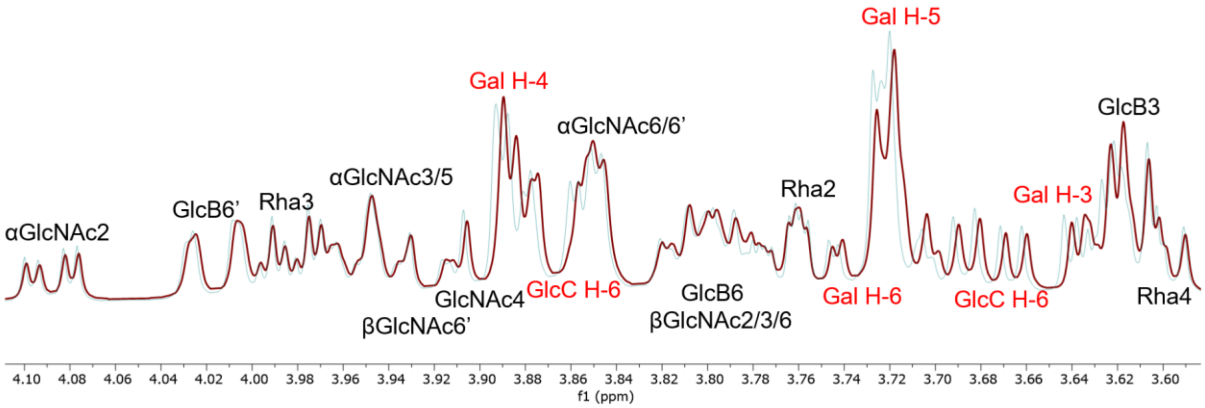

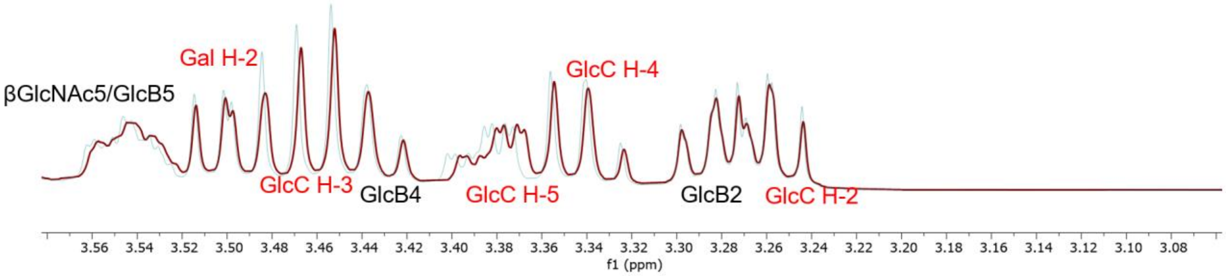

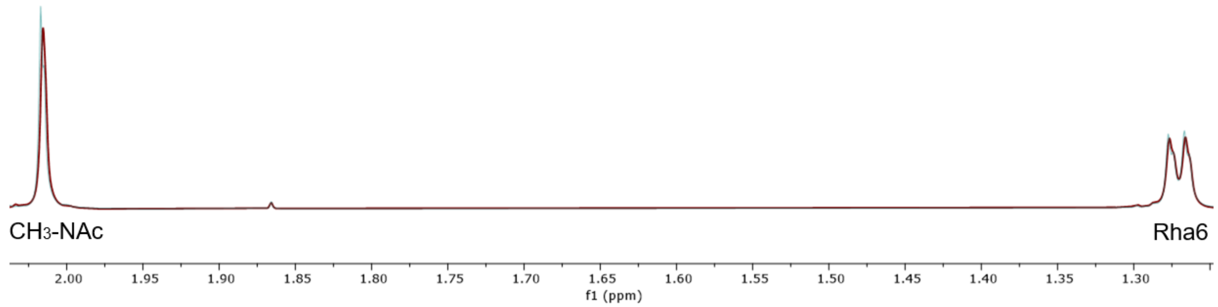

Figure S57 Overimposed ^{1}H NMR (600 MHz, D_2O) with assignments. The spectra are recorded for 2.4 mM **5mer** alone (green) or in the presence of 7.5 mM 5OH-indole (red).

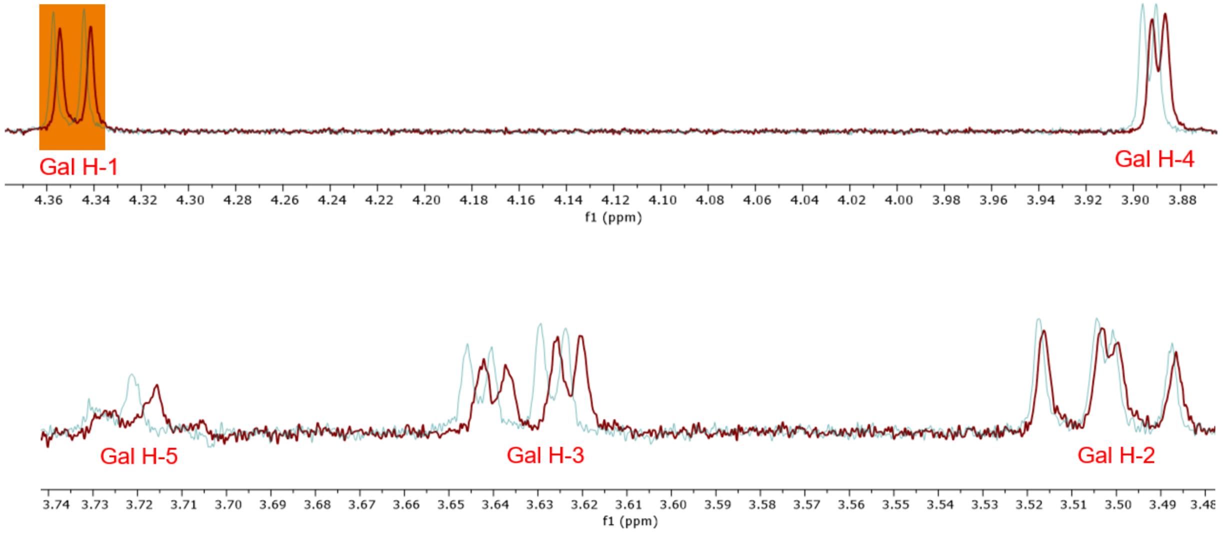

Figure S58 Overimposed 1D TOCSY (600 MHz, d9 = 350 ms, D_2O). The spectra are recorded for 2.4 mM **5mer** alone (green) or in the presence of 7.5 mM 5OH-indole (red). Resonances chosen with selective excitation are highlighted with orange to see the chemical shift change of Gal-H3 and Gal H-5.

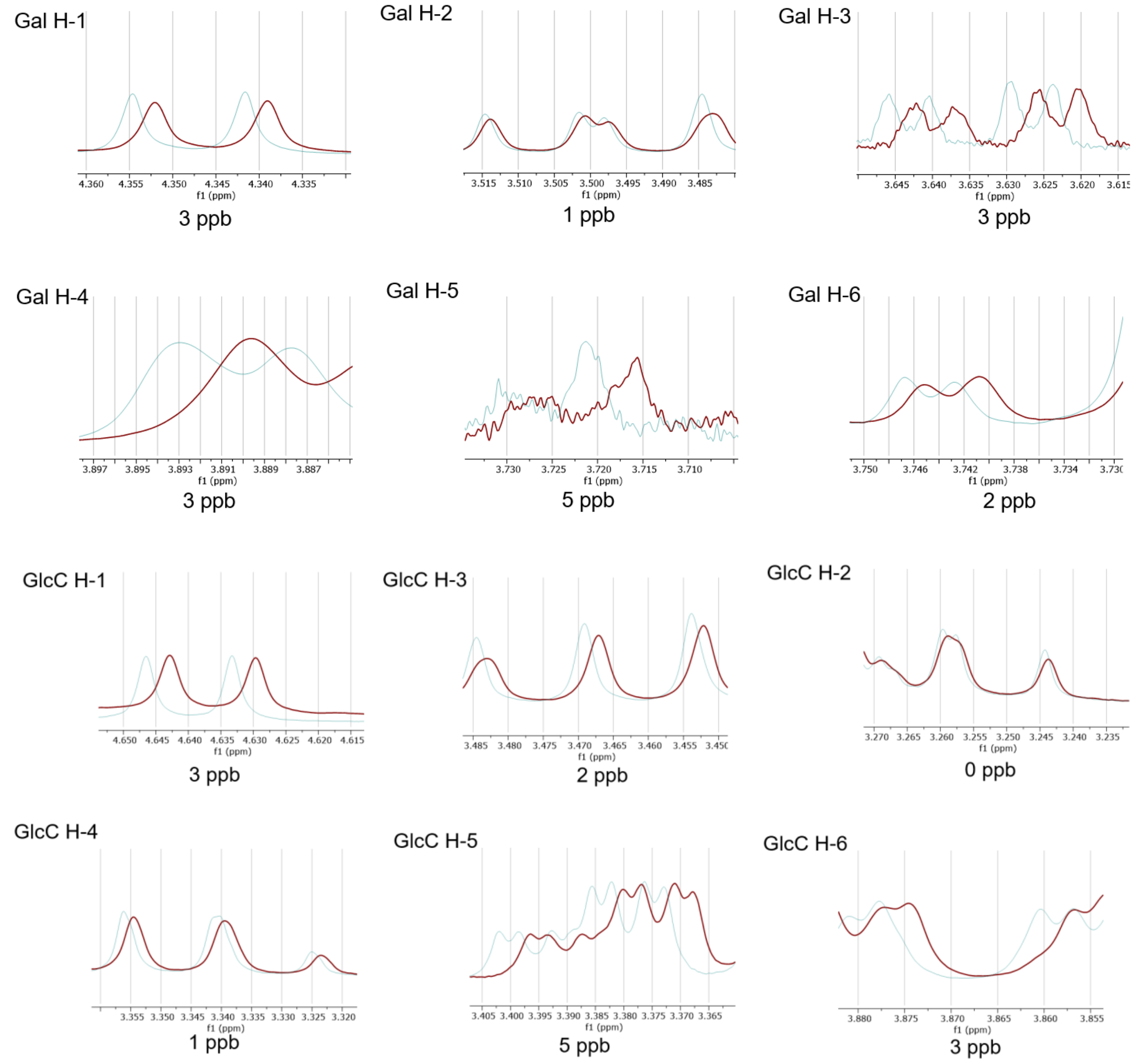

Figure S59 Excerpt of overimposed ^{1}H NMR and 1D TOCSY. The spectra are recorded for 2.4 mM **5mer** alone (green) or in the presence of 7.5 mM 5OH-indole (red).

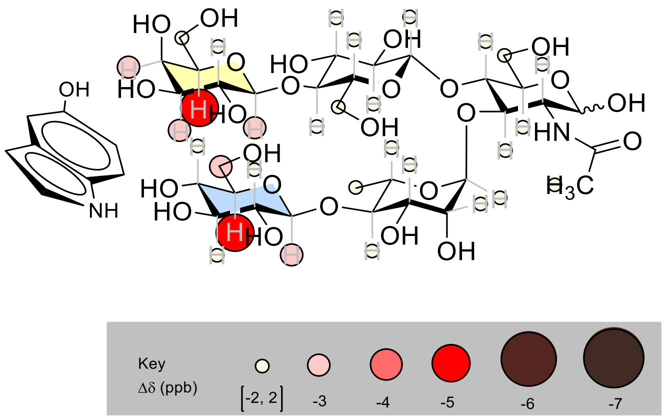

Figure S60 Experimentally observed chemical shift changes for **5mer** in the presence of 5OH-indole. βGlcNAc1 overlapped with the water peak and therefore could not be analyzed.

4.5.6 CH/π interactions analysis: 4mer-II with L-tryptophan

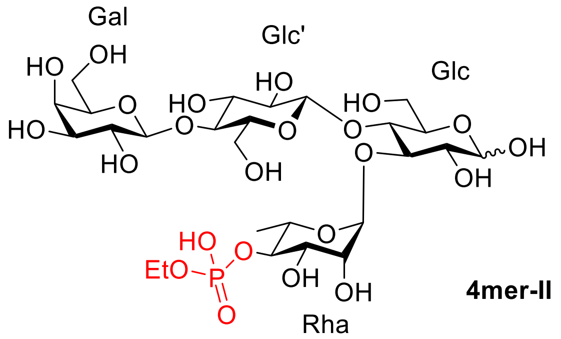

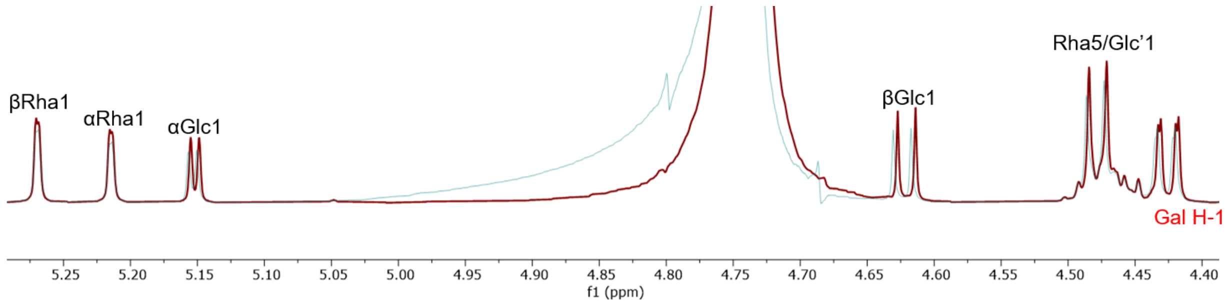

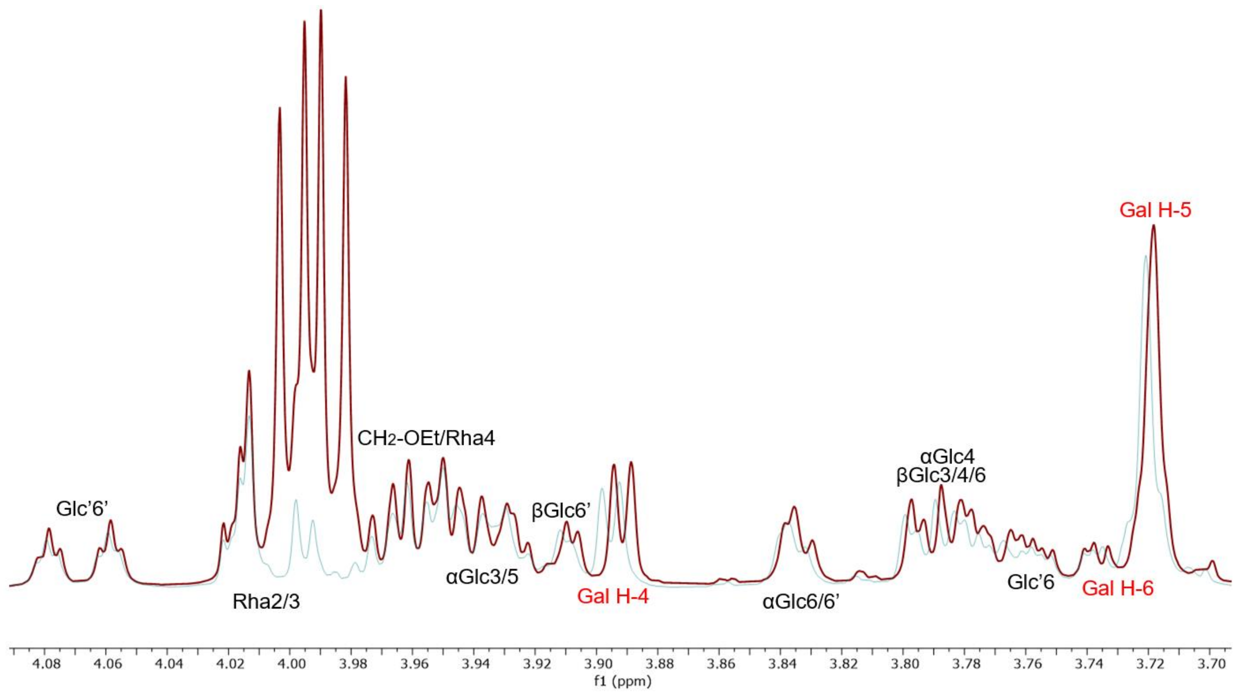

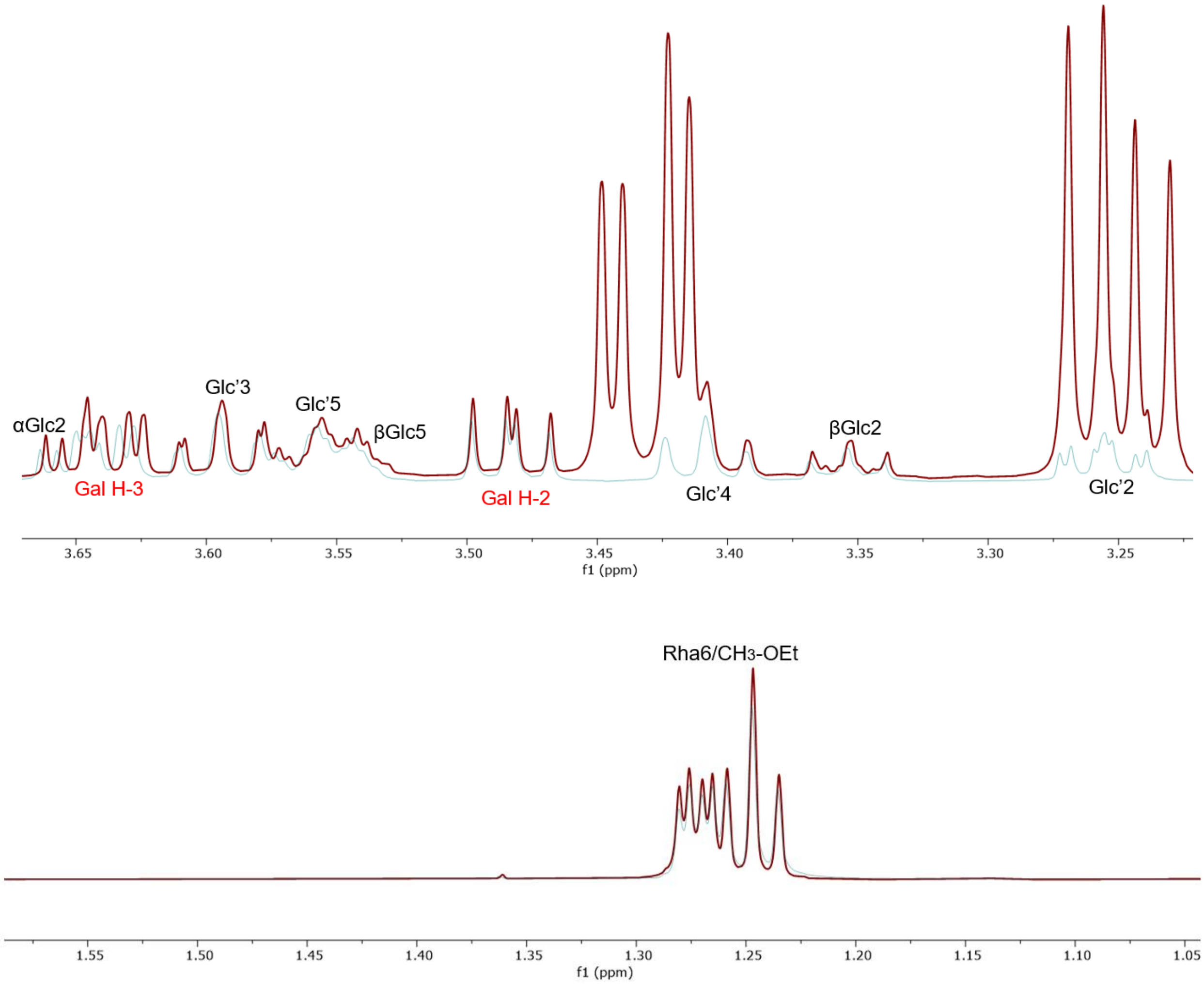

Figure S61 Overimposed ^{1}H NMR (600 MHz, D_2O) with assignments. The spectra are recorded for 3.5 mM **4mer-II** alone (green) or in the presence of 10.5 mM tryptophan (red).

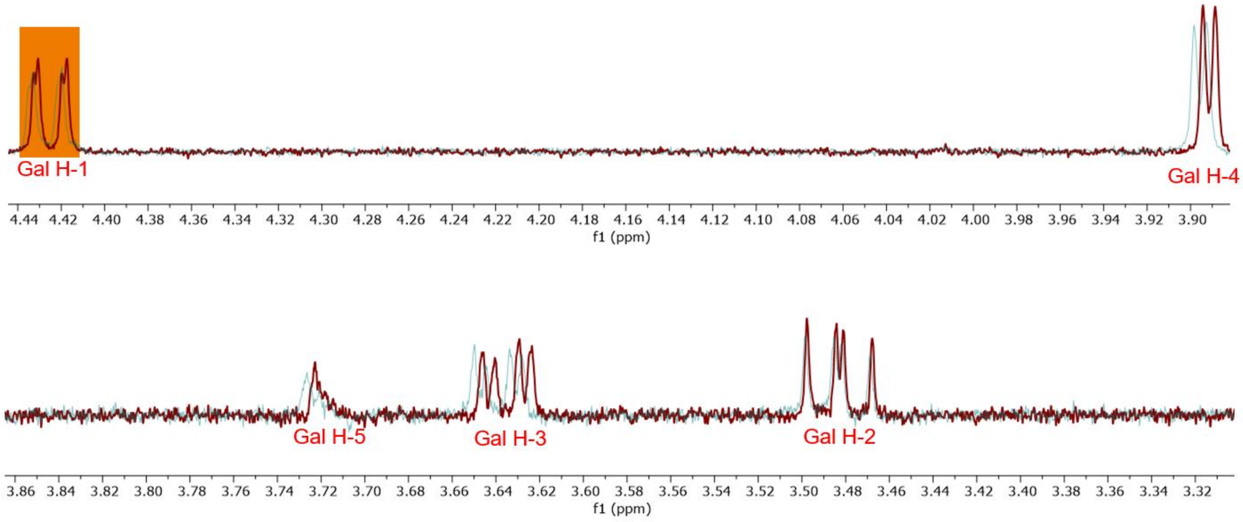

Figure S62 Overimposed 1D TOCSY (600 MHz, d9 = 350 ms, D_2O). The spectra are recorded for 3.5 mM **4mer-II** alone (green) or in the presence of 10.5 mM tryptophan (red). Resonances chosen with selective excitation are highlighted with orange to see the chemical shift change of Gal-H5.

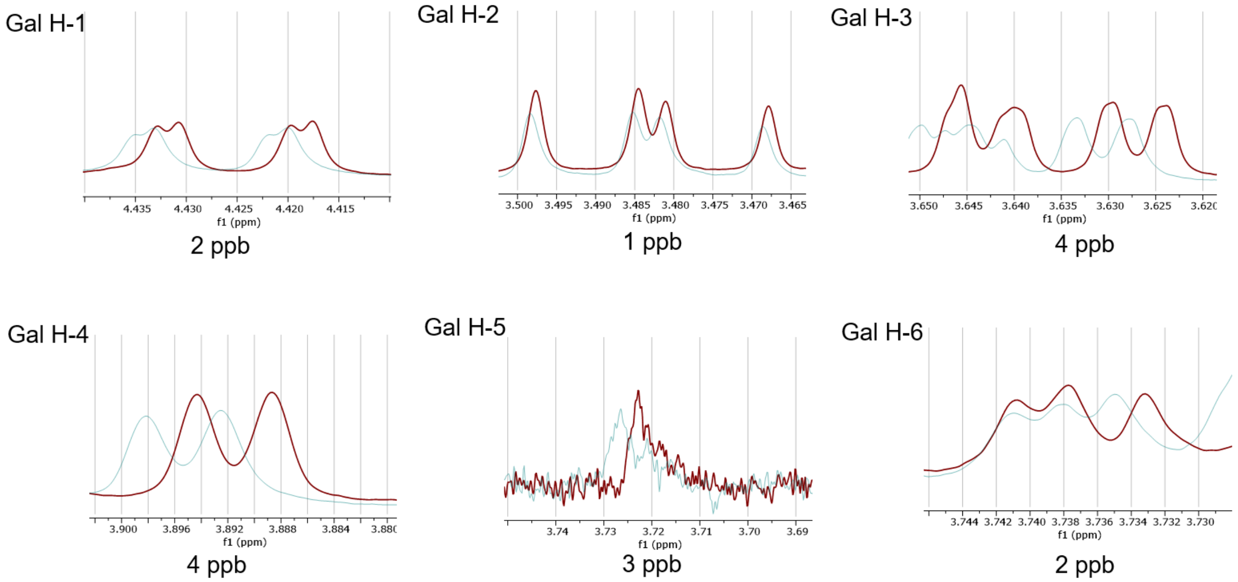

Figure S63 Excerpt of overimposed ^1H NMR and 1D TOCSY. The spectra are recorded for 3.5 mM **4mer-II** alone (green) or in the presence of 10.5 mM tryptophan (red).

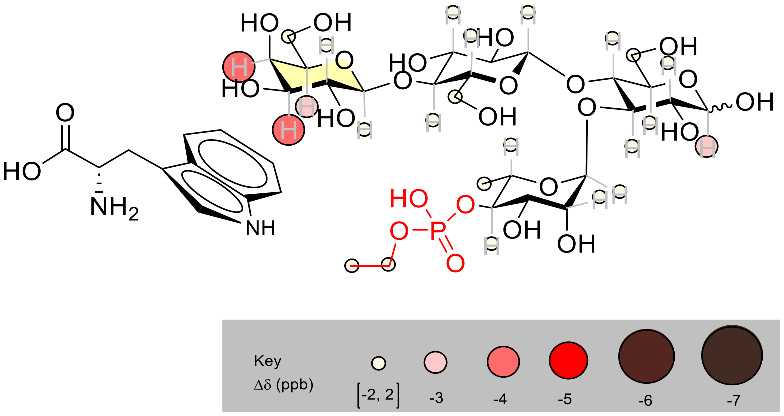

Figure S64 Experimentally observed chemical shift changes for **4mer-II** in the presence of Trp. As show in the figure, CH/ π interaction is mainly focused on the Gal unit and most of the chemical shift changes detected for protons of the other monosaccharides are less than 2 ppb. Glc'2 and Rha3 overlapped with the tryptophan side chain and therefore could not be analyzed.

4.5.7 CH/π interactions analysis: 4mer-III with L-tryptophan

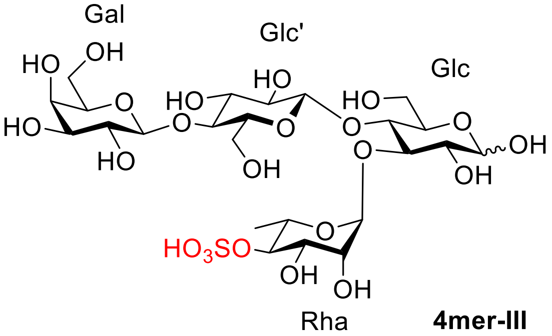

4mer-III

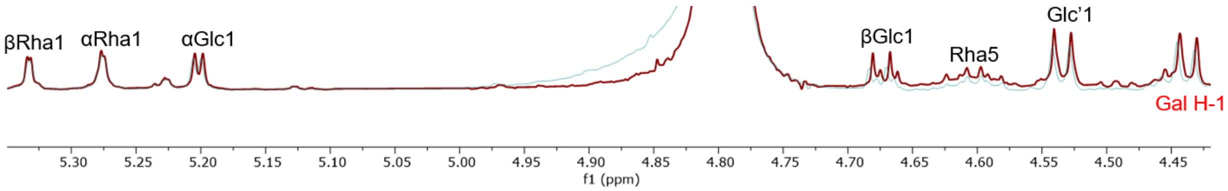

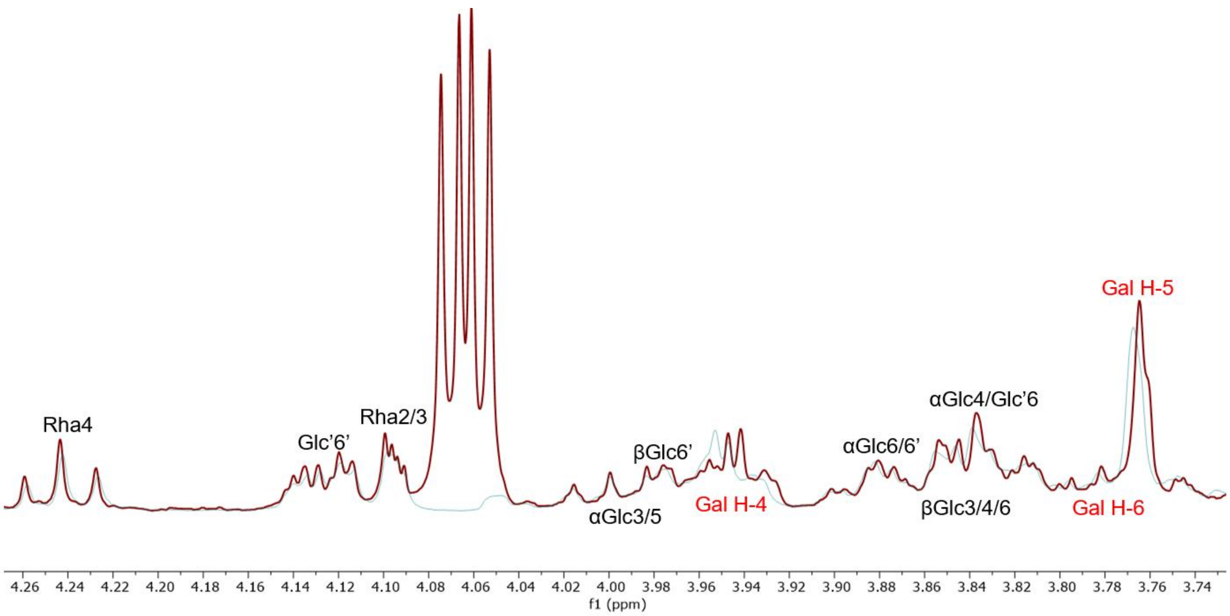

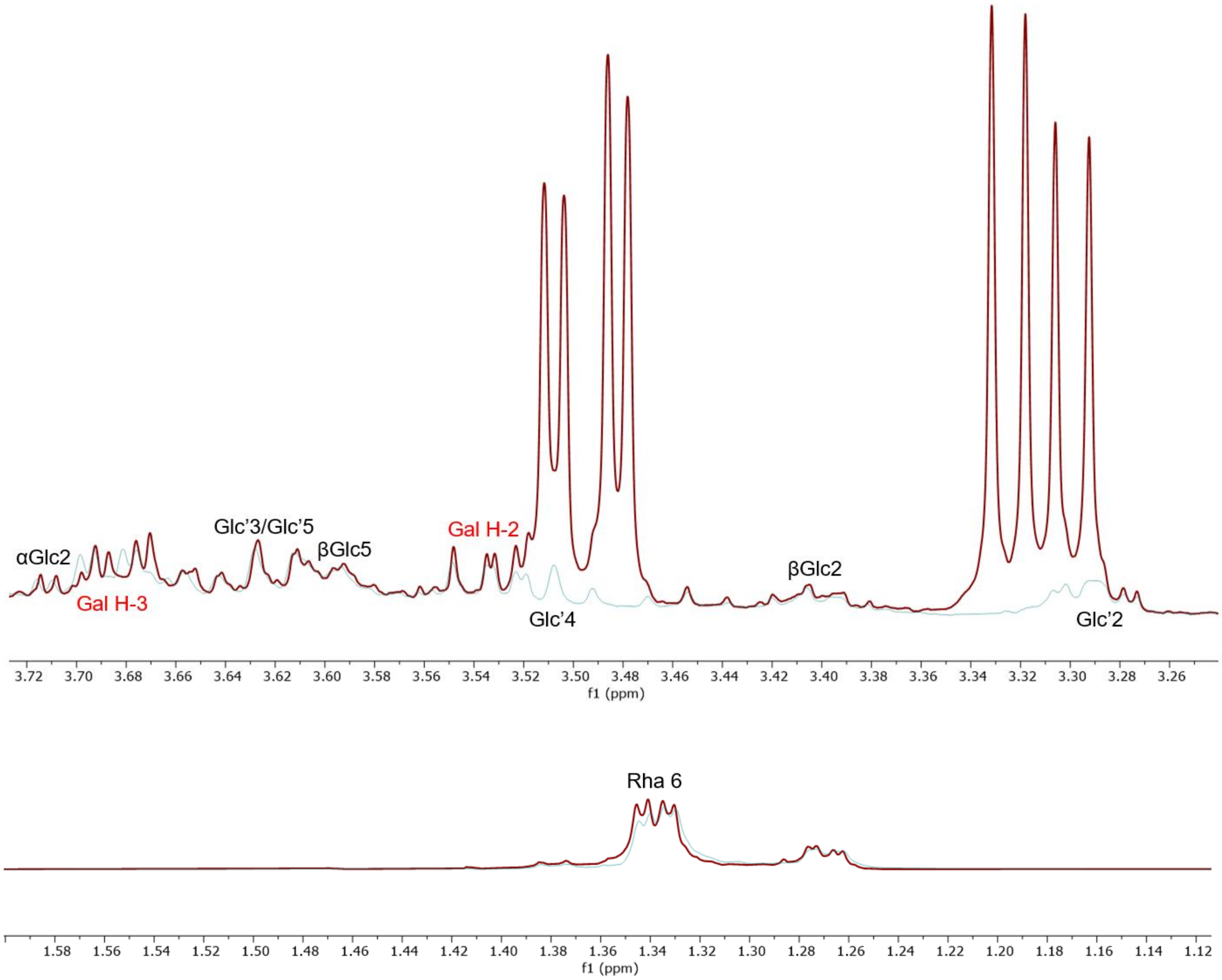

Figure S65 Overimposed ^{1}H NMR (600 MHz, D_2O) with assignments. The spectra are recorded for 3.5 mM **4mer-III** alone (green) or in the presence of 10.5 mM tryptophan (red).

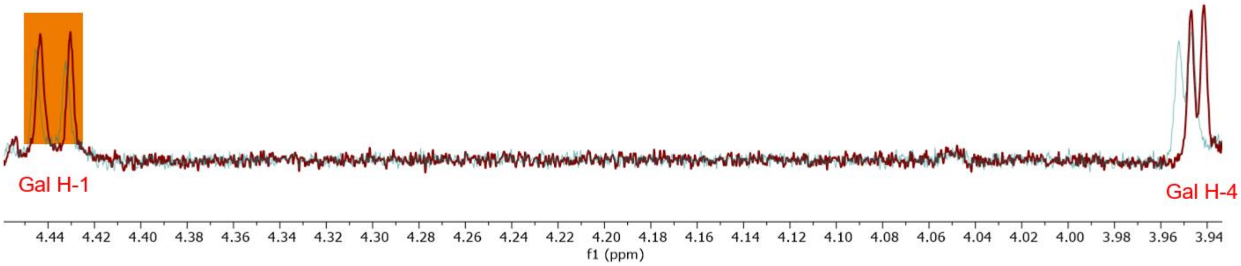

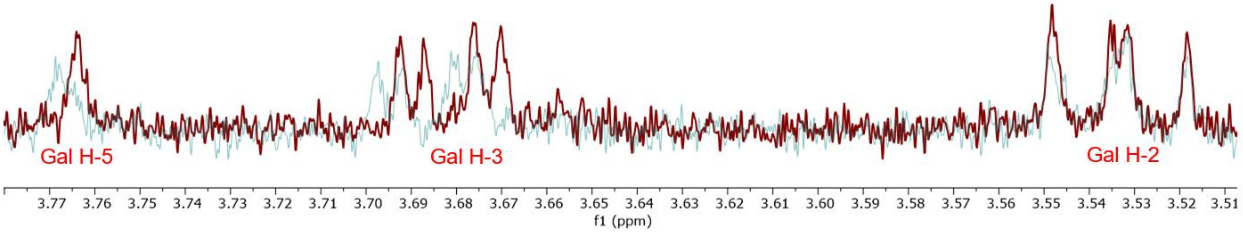

Figure S66 Overimposed 1D TOCSY (600 MHz, d9 = 350 ms, D_2O). The spectra are recorded for **4mer-III** (3.5 mM) alone (green) of in the presence of tryptophan (10.5 mM) (red). Resonances chosen with selective excitation are highlighted with orange to see the chemical shift change of Gal-H4 and Gal-H5.

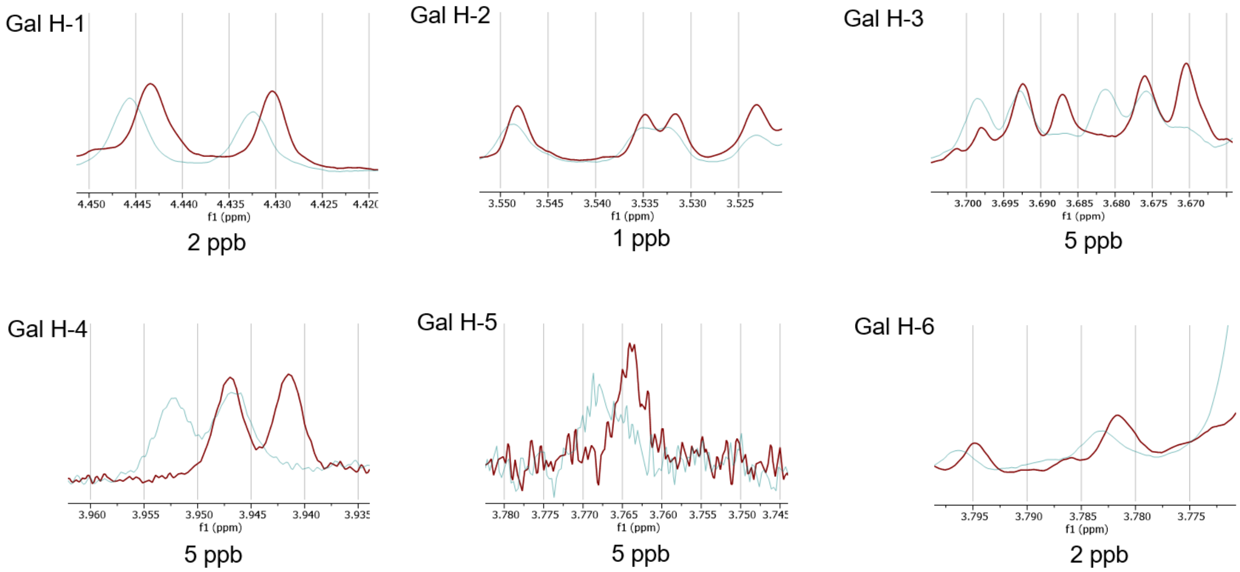

Figure S67 Excerpt of overimposed ^{1}H NMR and 1D TOCSY. The spectra are recorded for 3.5 mM **4mer-III** alone (green) or in the presence of 10.5 mM tryptophan (red).

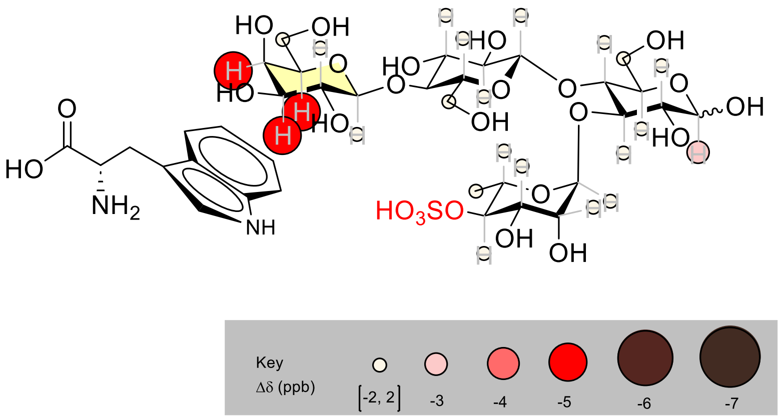

Figure S68 Experimentally observed chemical shift changes for **4mer-III** in the presence of Trp. As show in the figure, CH/ π interaction is mainly focused on the Gal unit and most of the chemical shift changes detected for protons of the other monosaccharides are less than 2 ppb. Glc'4 overlapped with the tryptophan side chain and therefore could not be analyzed.

4.5.8 CH/π interactions analysis: 4mer-IV with L-tryptophan

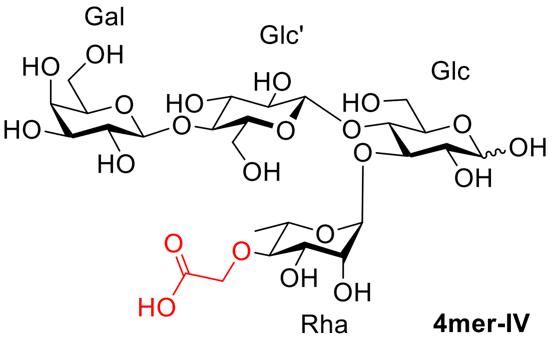

4mer-IV

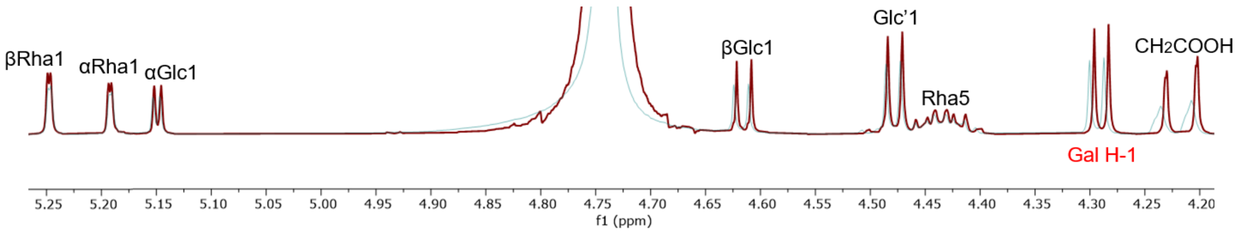

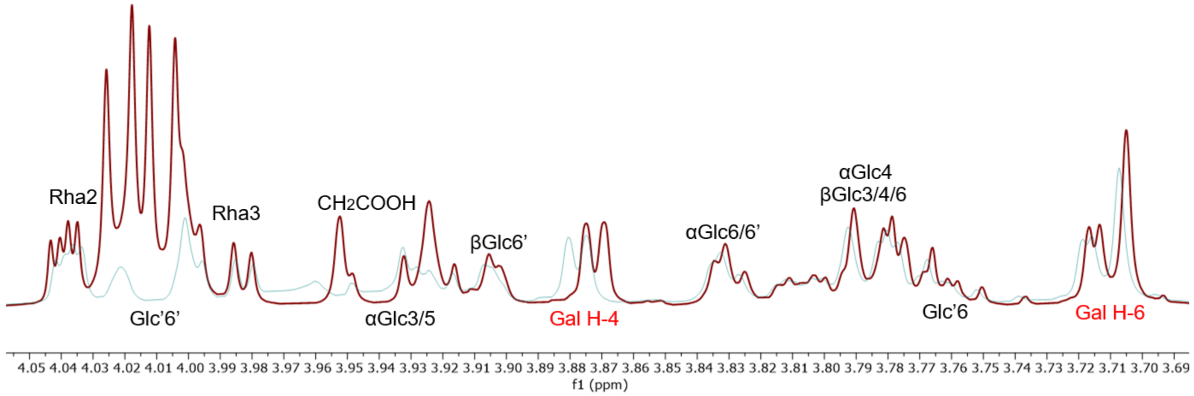

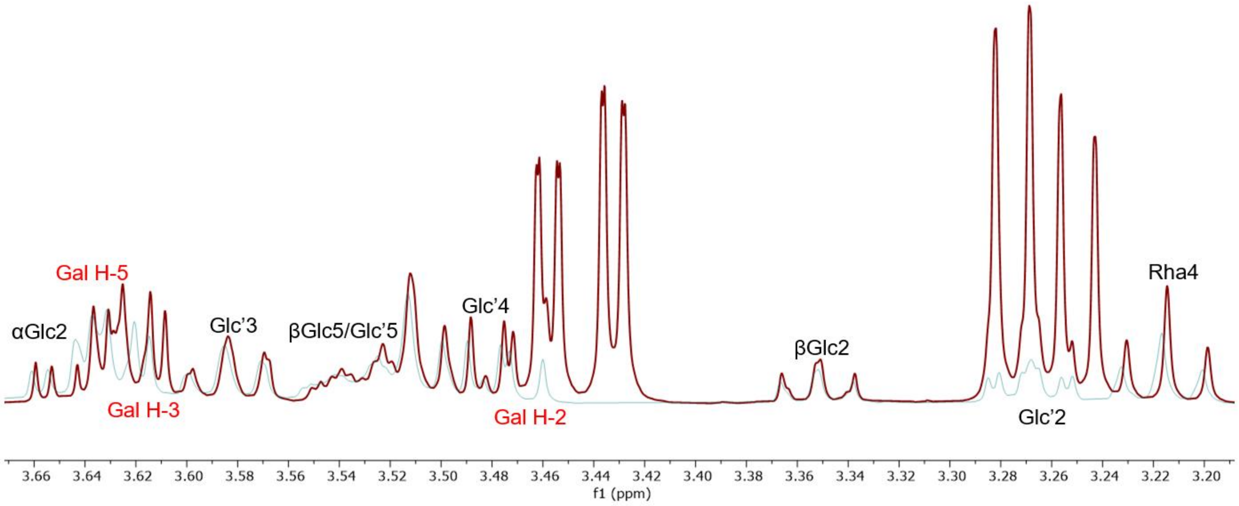

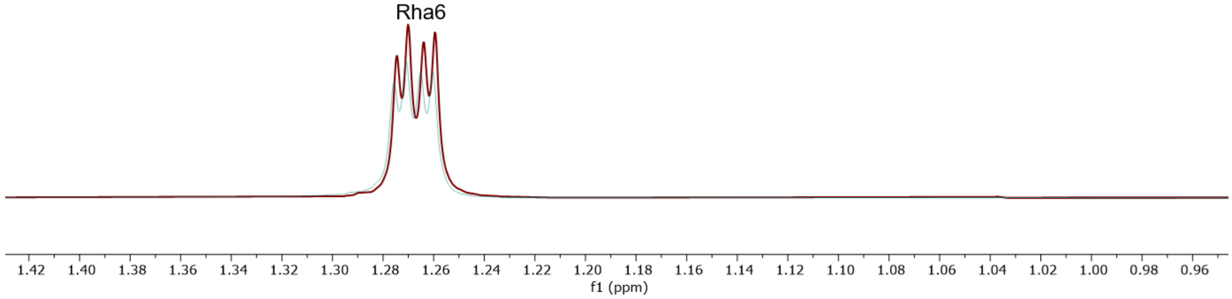

Figure S69 Overimposed ^{1}H NMR (600 MHz, D_2O) with assignments. The spectra are recorded for 3.5 mM **4mer-IV** alone (green) or in the presence of 10.5 mM tryptophan (red).

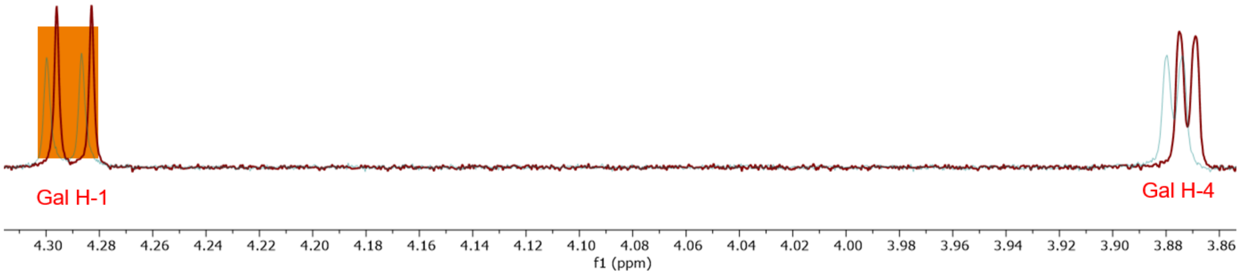

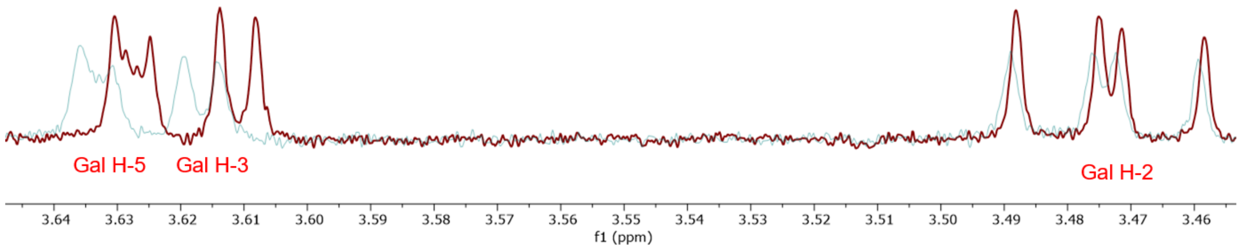

Figure S70 Overimposed 1D TOCSY (600 MHz, d9 = 350 ms, D_2O). The spectra are recorded for 3.5 mM **4mer-IV** alone (green) or in the presence of 10.5 mM tryptophan (red). Resonances chosen with selective excitation are highlighted with orange to see the chemical shift change of Gal-H2 and Gal-H5.

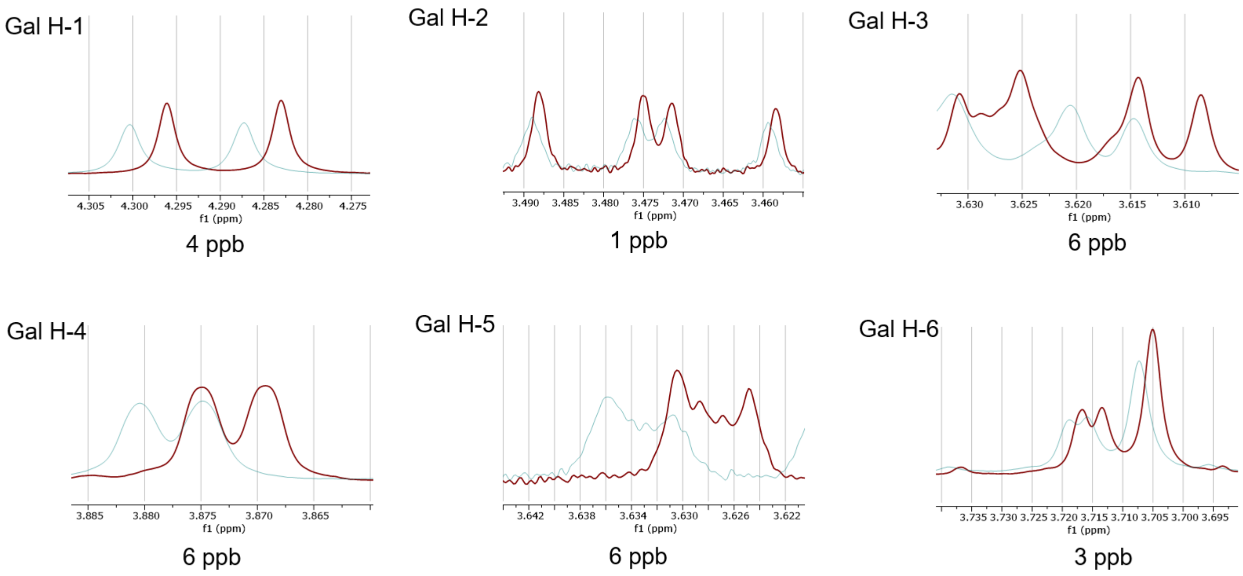

Figure S71 Excerpt of overimposed ^{1}H NMR and 1D TOCSY. The spectra are recorded for 3.5 mM **4mer-IV** alone (green) or in the presence of 10.5 mM tryptophan (red).

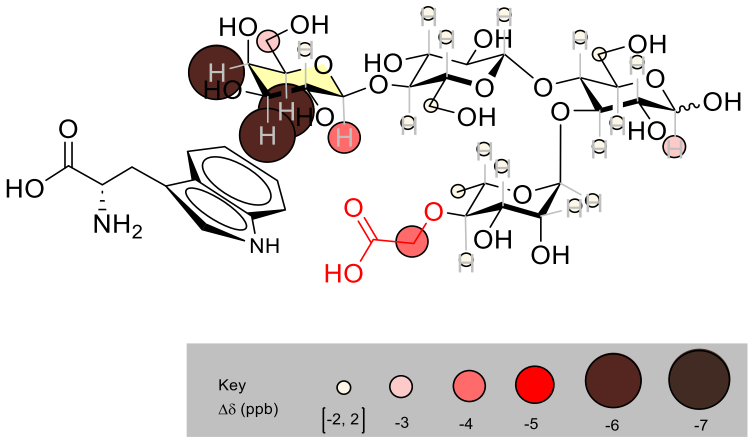

Figure S72 Experimentally observed chemical shift changes for **4mer-IV** in the presence of Trp. As show in the figure, CH/ π interaction is mainly focused on the Gal unit and most of the chemical shift changes detected for protons of the other monosaccharides are less than 2 ppb. Glc'2 overlapped with the tryptophan side chain and therefore could not be analyzed.

4.5.9 CH/π interactions analysis: 4mer-V with L-tryptophan

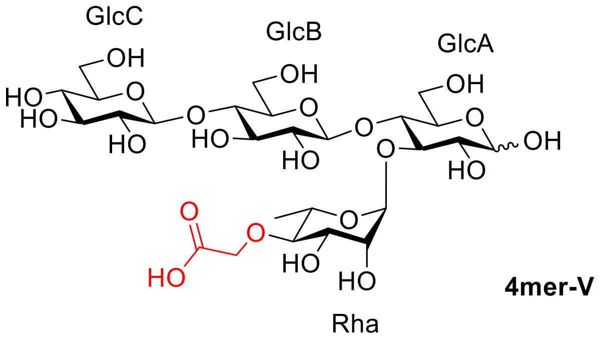

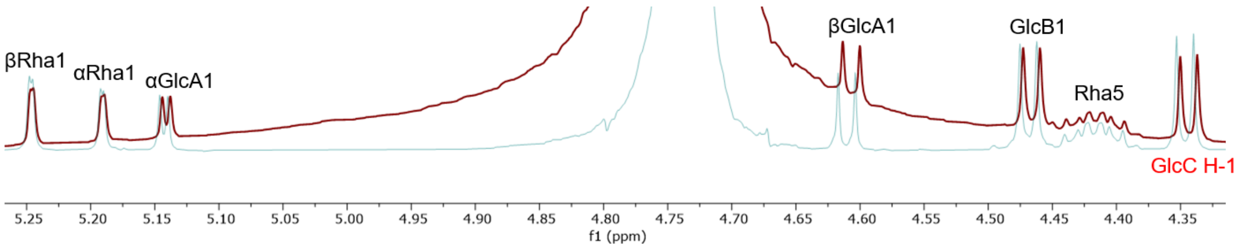

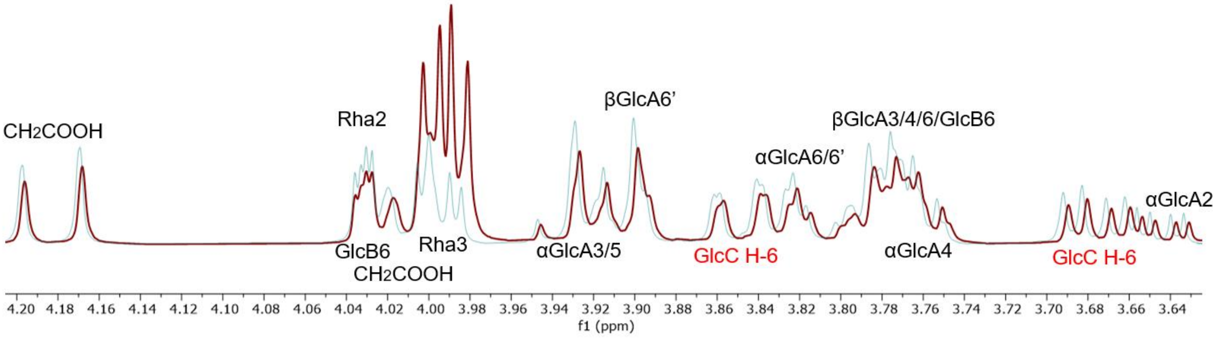

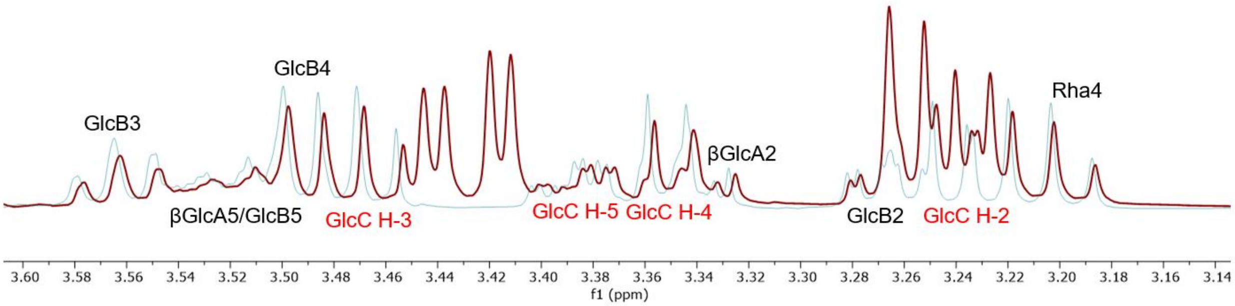

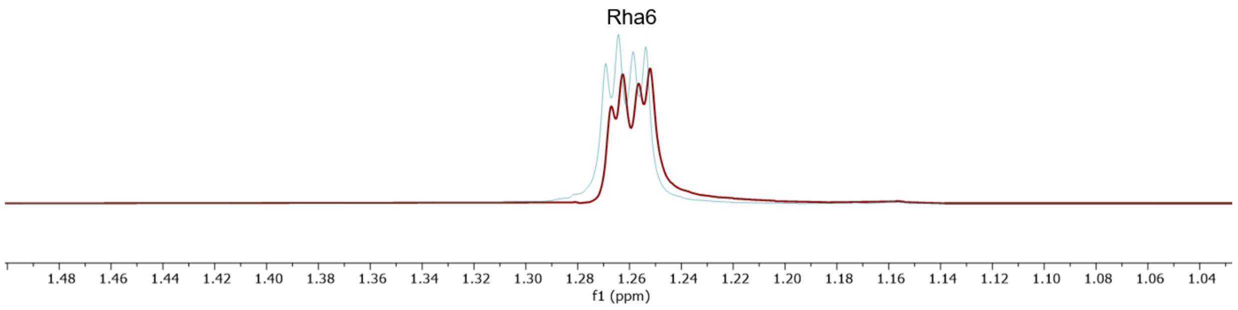

Figure S73 Overimposed ^{1}H NMR (600 MHz, D_2O) with assignments. The spectra are recorded for 3.5 mM **4mer-V** alone (green) or in the presence of 10.5 mM tryptophan (red).

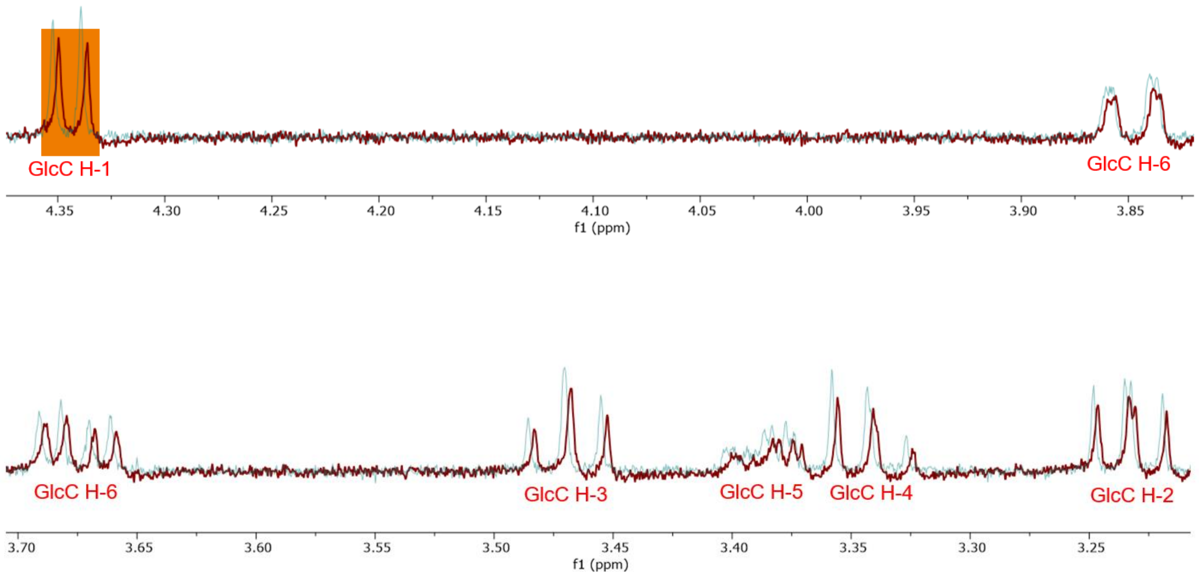

Figure S74 Overimposed 1D TOCSY (600 MHz, d9 = 350 ms, D_2O). The spectra are recorded for 3.5 mM **4mer-V** alone (green) or in the presence of 10.5 mM tryptophan (red). Resonances chosen with selective excitation are highlighted with orange to see the chemical shift change of GlcC-H2 and GlcC-H4.

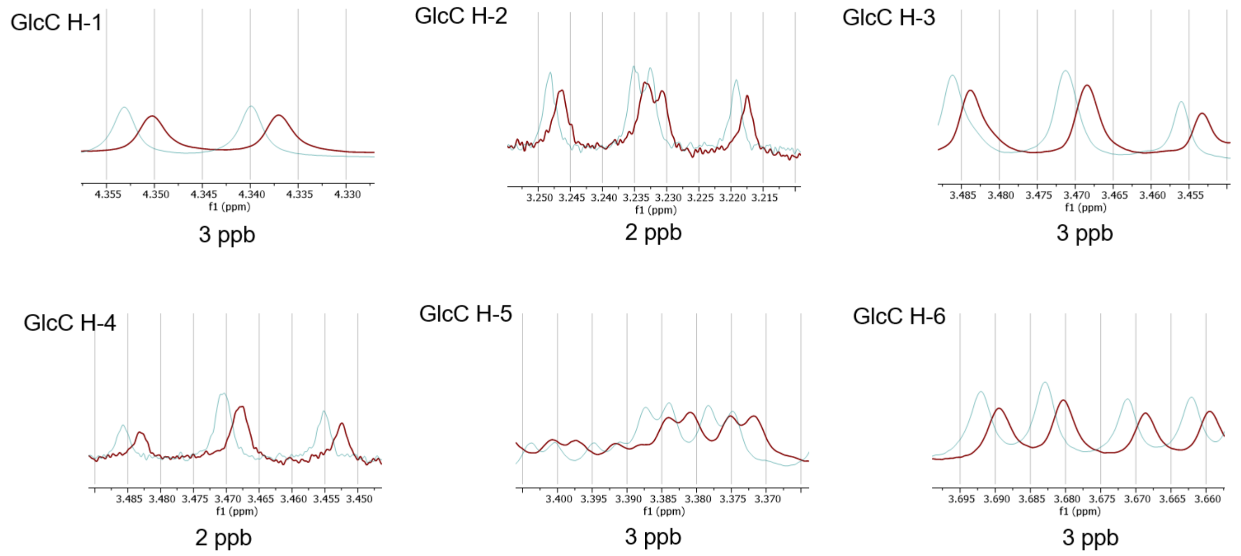

Figure S75 Excerpt of overimposed ^{1}H NMR and 1D TOCSY. The spectra are recorded for 3.5 mM **4mer-V** alone (green) or in the presence of 10.5 mM tryptophan (red).

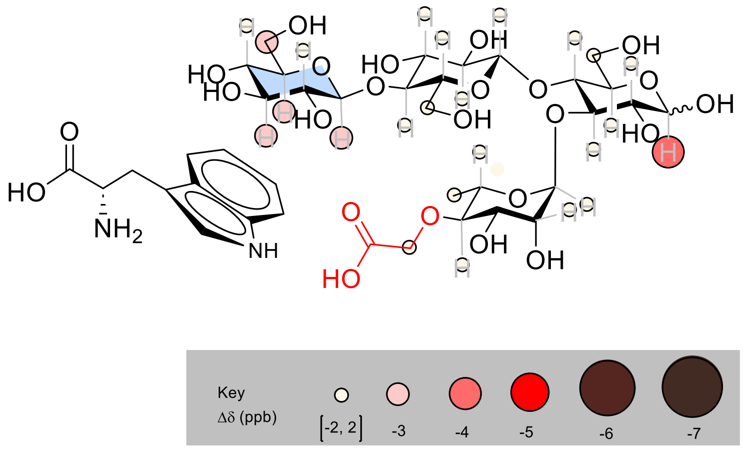

Figure S76 Experimentally observed chemical shift changes for **4mer-V** in the presence of Trp. Most of the chemical shift changes detected for protons of the other monosaccharides are less than 2 ppb. (Rha3 was overlapped with tryptophan side chain.)

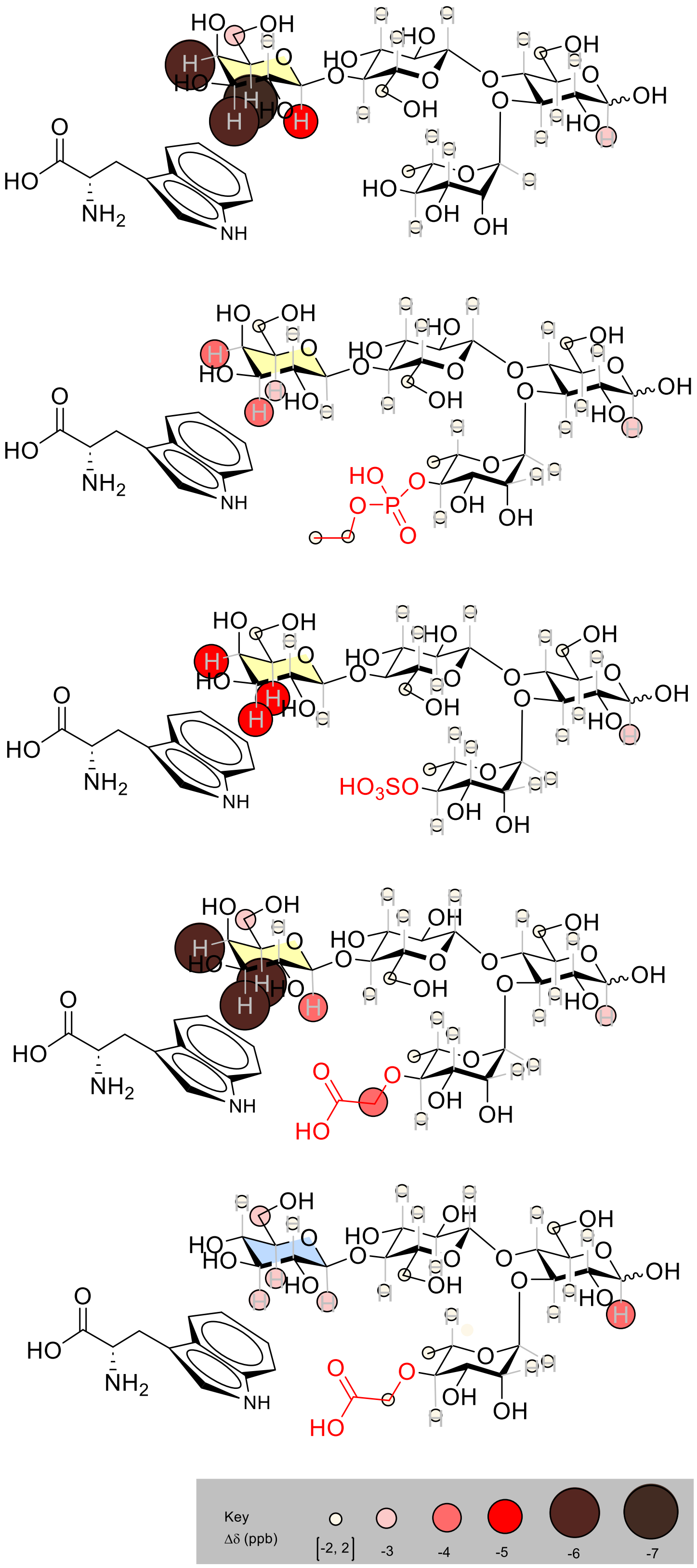

Figure S77 Comparison of CH/ π interaction between **4mer I-V** with Trp.

5 Pictet-Spengler reaction

5.1 Pictet-Spengler modification of L- tryptophan

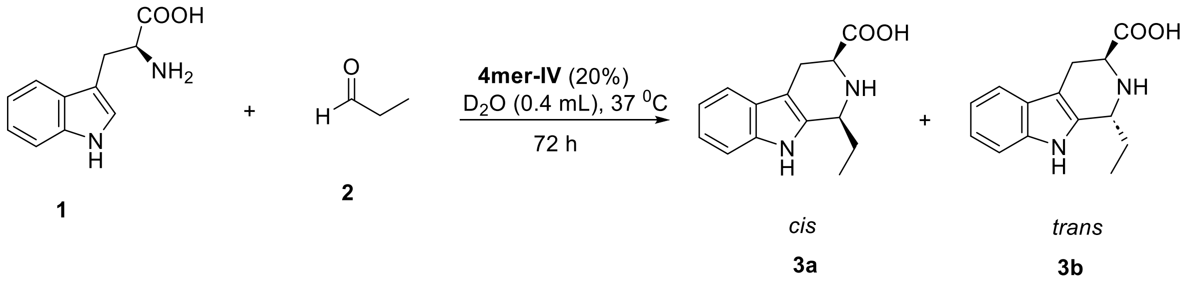

L- Tryptophan (5.1 mg, 0.025 mmol) and **4mer-IV** (3.6 mg, 0.005 mmol) was dissolved in D_2O (0.4 mL, containing DMSO (6.5 mM, 0.18 µL) as internal standard). Propionaldehyde (22 µL, 0.3 mmol) was added to the reaction while stirring at 37 ^{0}C. After 72 h, the reaction was analyzed by ^{1}H NMR to determine a 75% yield (cis : trans = 75 : 25, see Table in Section 5.2, entry 14). The target compounds were isolated by HPLC (**Method C3**).

3a: ^{1}H NMR (600 MHz, D_2O) δ 7.59 (dd, *J* = 7.9, 1.1 Hz, 1H), 7.44 (dt, *J* = 8.2, 0.9 Hz, 1H), 7.24 (m, 1H), 7.15 (m, 1H), 4.63 – 4.54 (m, 1H), 3.96 (dd, *J* = 12.2, 5.1 Hz, 1H), 3.38 (m, 1H), 3.00 (m, 1H), 2.29 (m, 1H), 1.96 (m, 1H), 1.10 (t, *J* = 7.5 Hz, 3H).

3b: ^{1}H NMR (600 MHz, D_2O) δ 7.64 – 7.57 (m, 1H), 7.44 (dt, *J* = 8.2, 1.1 Hz, 1H), 7.24 (m, 1H), 7.18 – 7.12 (m, 1H), 4.75 (s, 1H), 4.28 – 4.20 (m, 1H), 3.35 (dd, *J* = 16.4, 5.8 Hz, 1H), 3.13 (dd, *J* = 16.5, 8.3 Hz, 1H), 2.10 – 1.98 (m, 2H), 1.09 (td, *J* = 7.5, 1.2 Hz, 3H).

NMR data were consistent with the reported reference.[13]

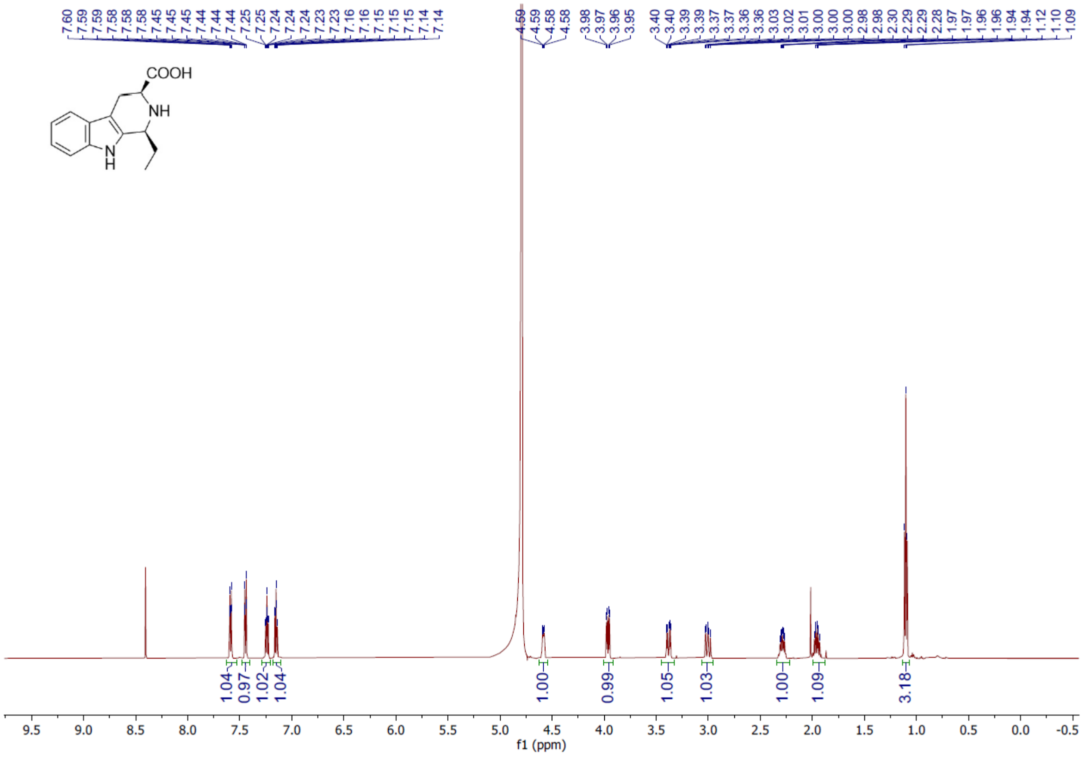

^1H NMR of **3a** (600 MHz, D_2O)

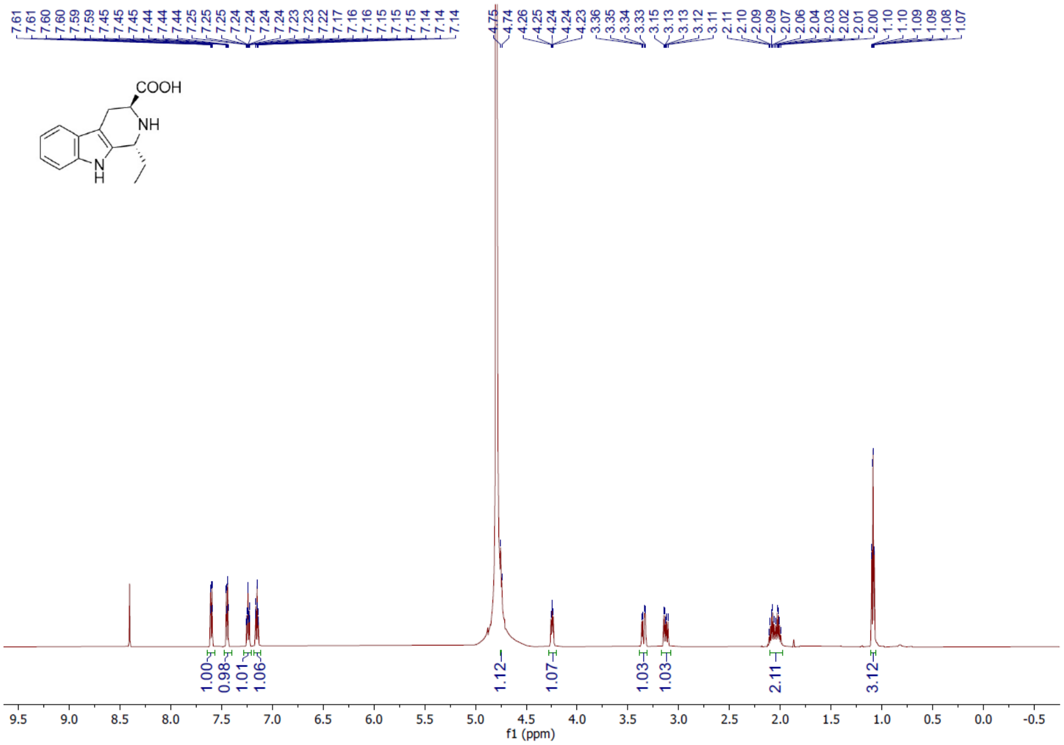

^1H NMR of **3b** (600 MHz, D_2O)

5.2 Reaction conditions optimization

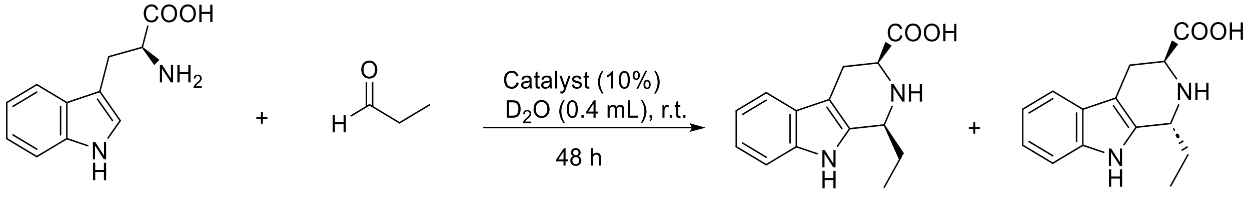

Entry	Conditions[a]	Conversion [%][b]	Yield [%][b]	cis : trans[b]
1	Without catalyst	-	<=5	-
2	**4mer-II**	-	<=5	-
3	**4mer-III**	-	<=5	-
4	**4mer-IV**	16	15	76 : 24
5	**4mer-IV**, **2** (0.3 mmol)	24	23	78 : 22
6	**4mer-IV**, **2** (0.3 mmol), DMSO (10%)	18	-	-
7	**4mer-IV**, **2** (0.3 mmol), 168 h	61	55	78 : 22
8	**3mer**, **2** (0.3 mmol), 168 h	29	28	78 : 22
9	**4mer-V**, **2** (0.3 mmol), 168 h	50	43	79 : 21
10	**4mer-IV** (20%), **2** (0.3 mmol)	38	35	79 : 21
11	**4mer-IV**, **2** (0.3 mmol), 37 °C	60	56	75 : 25
12	**4mer-IV** (20%), **2** (0.3 mmol), 37 °C, 72 h	84	75	75 : 25
13	AcOH (20%), **2** (0.3 mmol), 37 °C, 72 h	46	44	73 : 27
14	AcOH + Galactose (20%), **2** (0.3 mmol), 37 °C, 72 h	52	48	74 : 26
15	**3mer** (20%), **2** (0.3 mmol), 37 °C, 72 h	70	58	71 : 29
16	Glycolic acid (20%), **2** (0.3 mmol), 37 °C, 72 h	72	63	73 : 27
17	AcOH (stoic.), **2** (0.3 mmol), 37 °C, 72 h	80	70	73 : 27
18	HCl, **2** (0.3 mmol), 37 °C, 72 h [c]	100	16	44 : 56

[a]The reaction was conducted in a small glass vessel (1.5 mL), while stirring. [b]The conversion, yield, and selectivity were monitored by ^{1}H NMR. [c]The reaction was treated with 1 M HCl.

5.3 NMR of crude reaction system

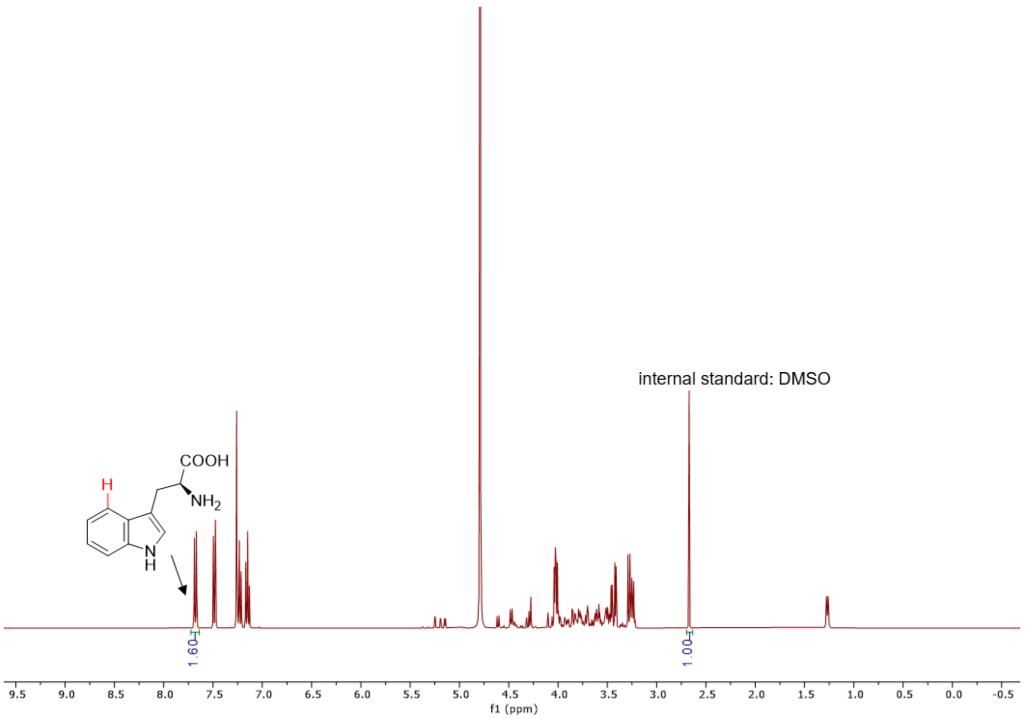

^{1}H NMR (400 MHz, D_2O) of **entry 12** at t = 0 h. The proton used for analysis is highlighted in red.

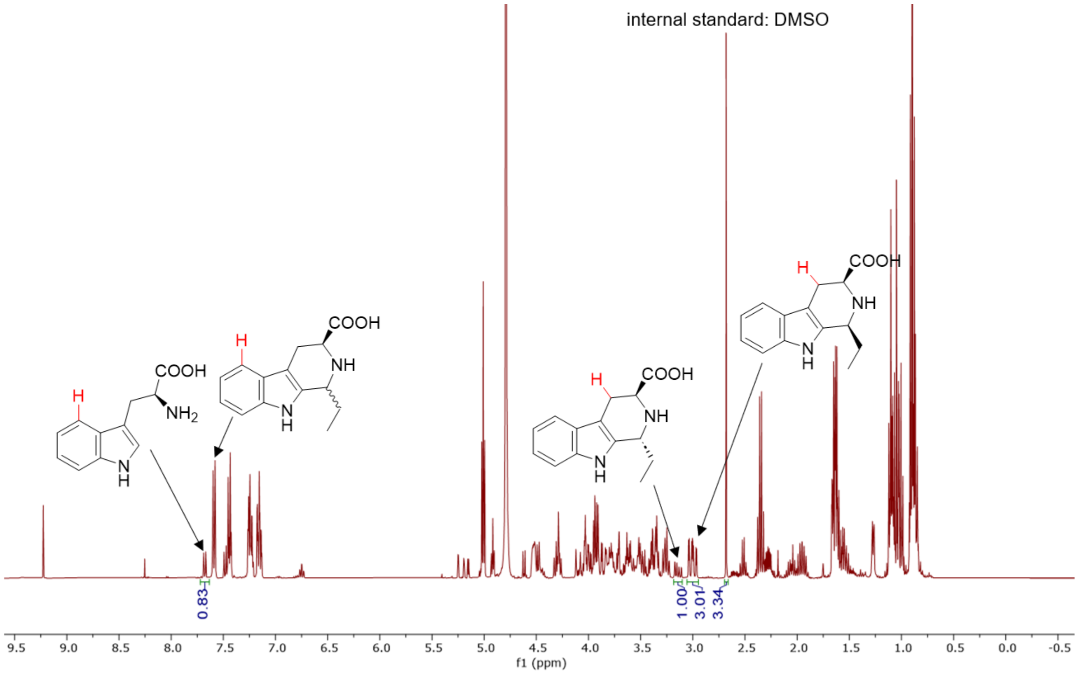

^{1}H NMR (400 MHz, D_2O) of **entry 12** at t = 72 h. The protons used for analysis is highlighted in red.

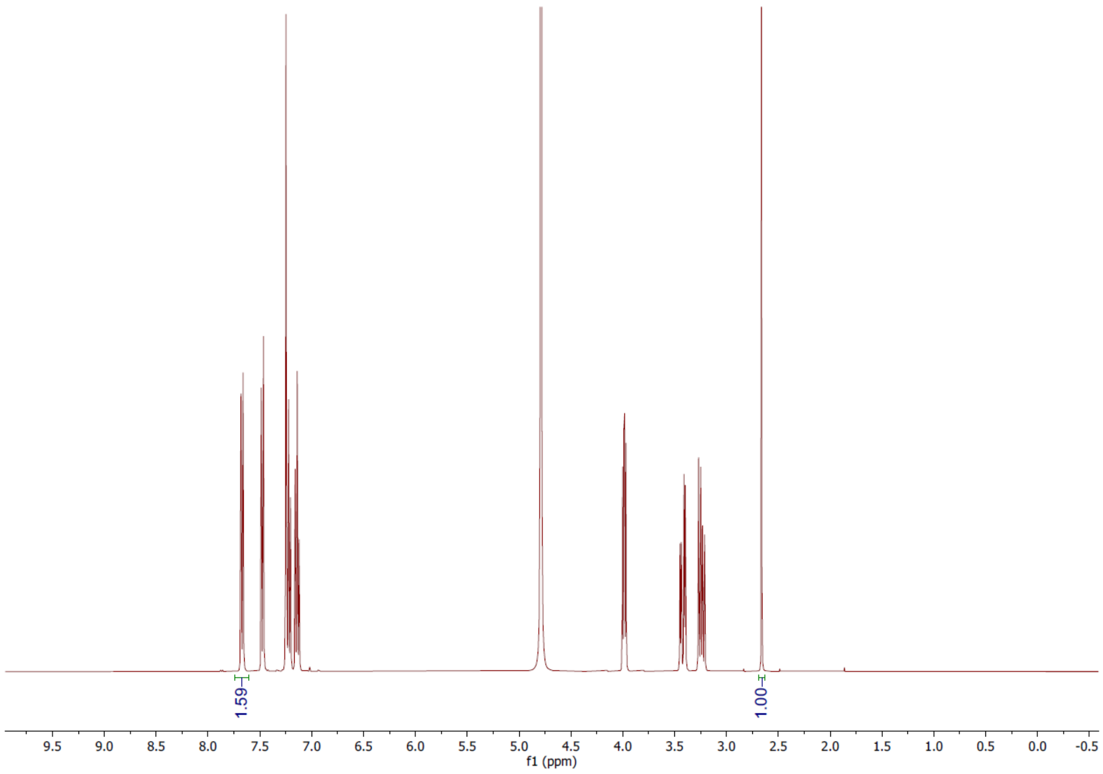

^{1}H NMR (400 MHz, D_2O) of **entry 1** at t = 0 h.

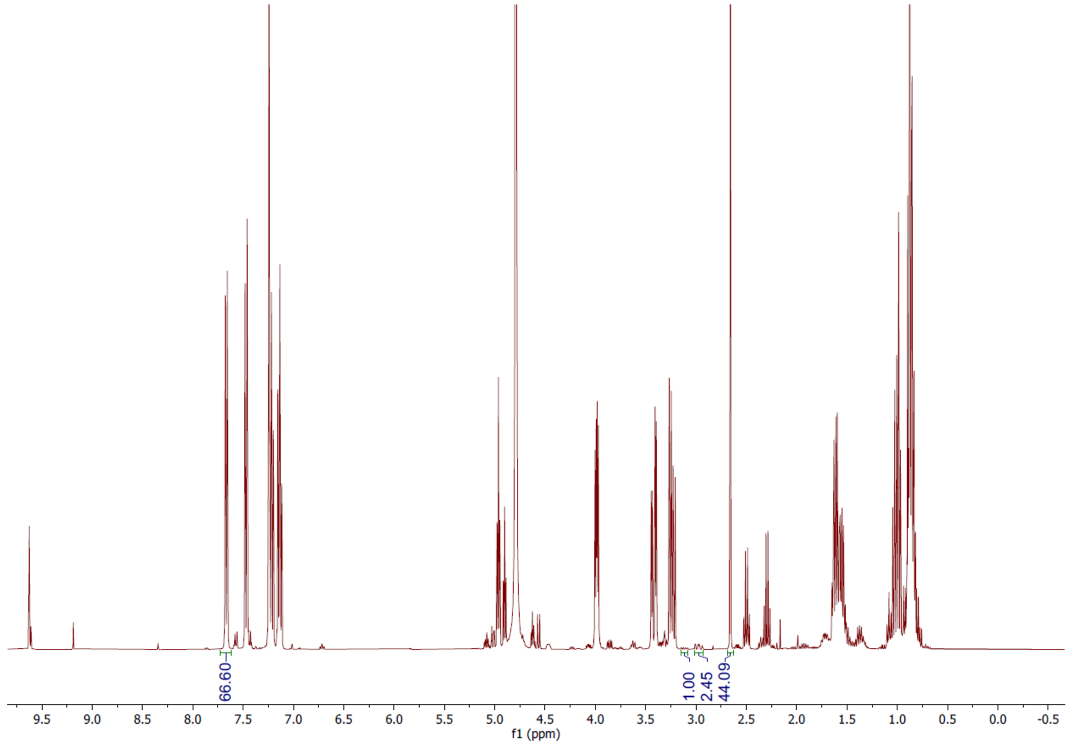

^{1}H NMR (400 MHz, D_2O) of **entry 1** at t = 48 h.

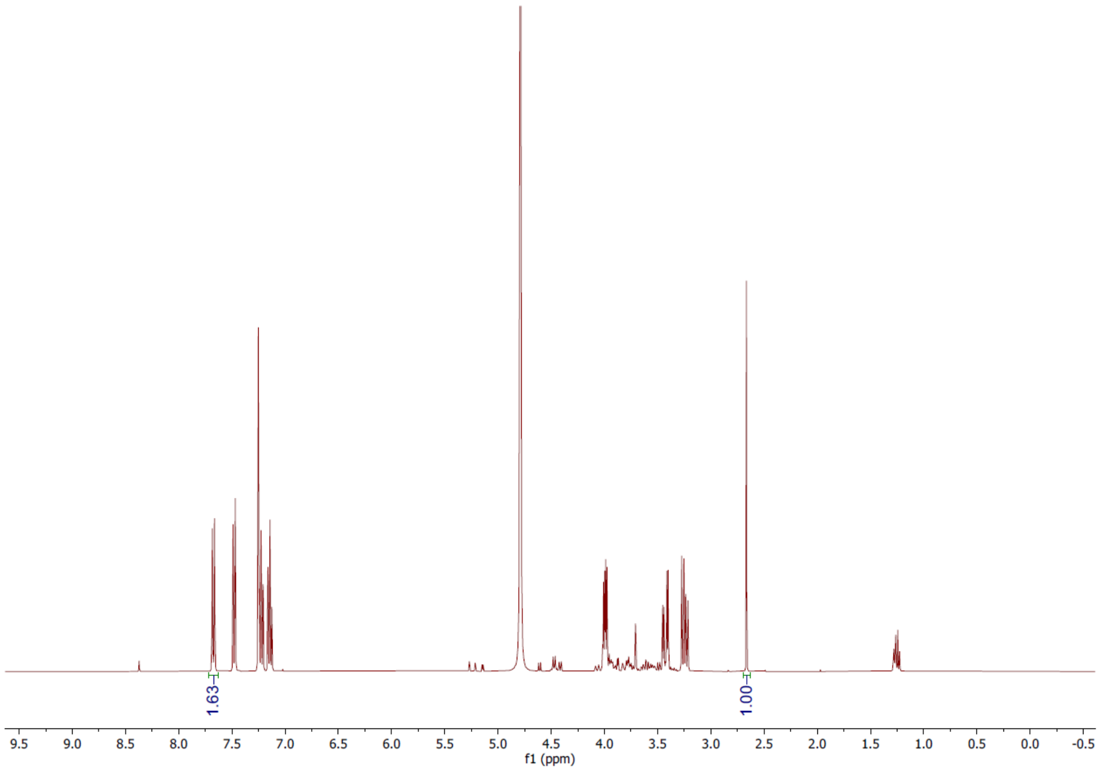

^{1}H NMR (400 MHz, D_2O) of **entry 2** at t = 0 h.

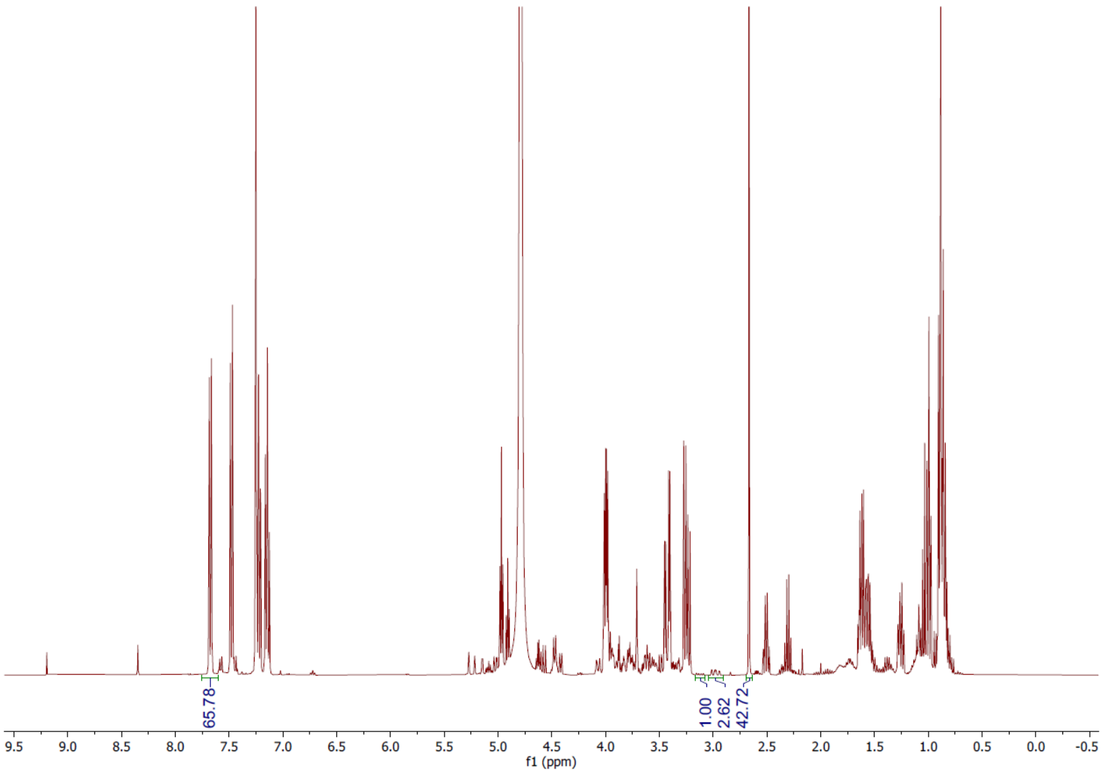

^{1}H NMR (400 MHz, D_2O) of **entry 2** at t = 48 h.

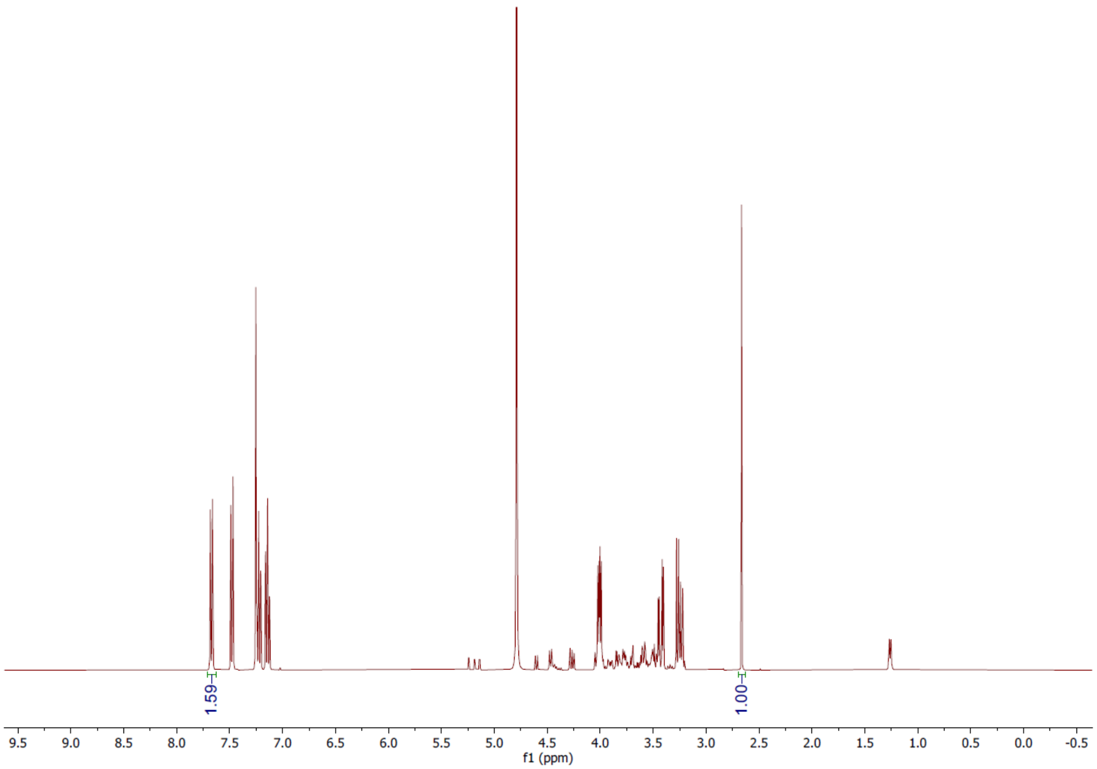

^1H NMR (400 MHz, D_2O) of **entry 4** at t = 0 h.

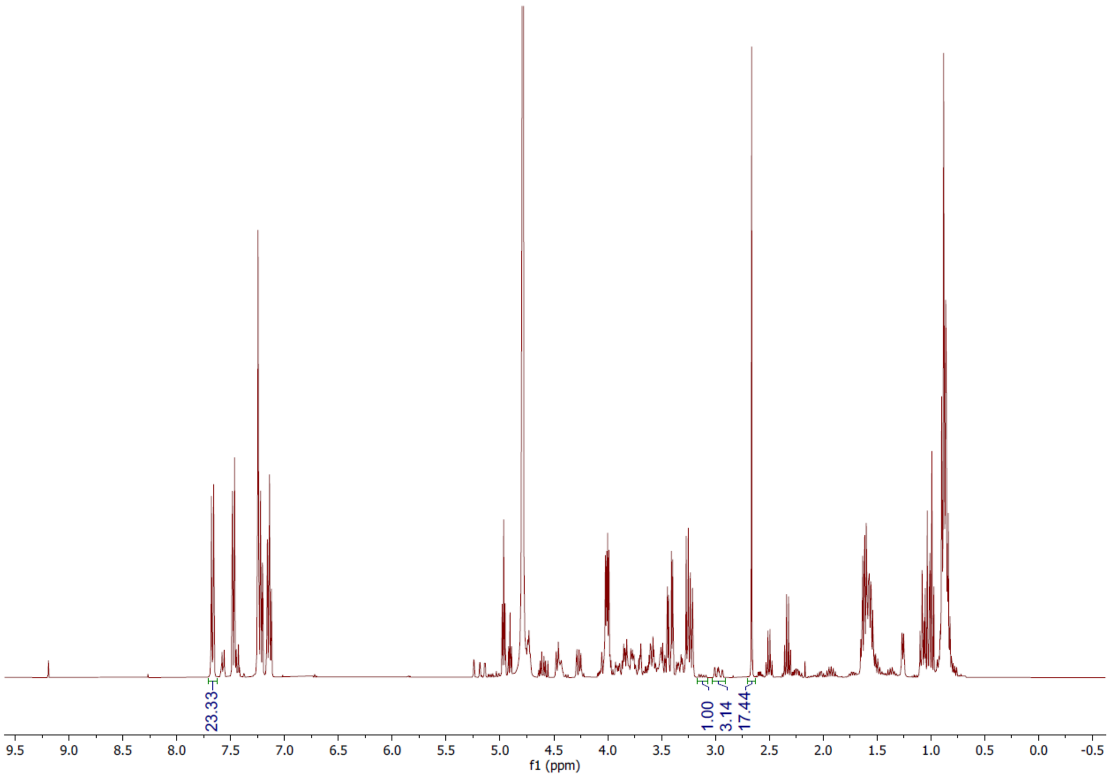

^1H NMR (400 MHz, D_2O) of **entry 4** at t = 48 h.

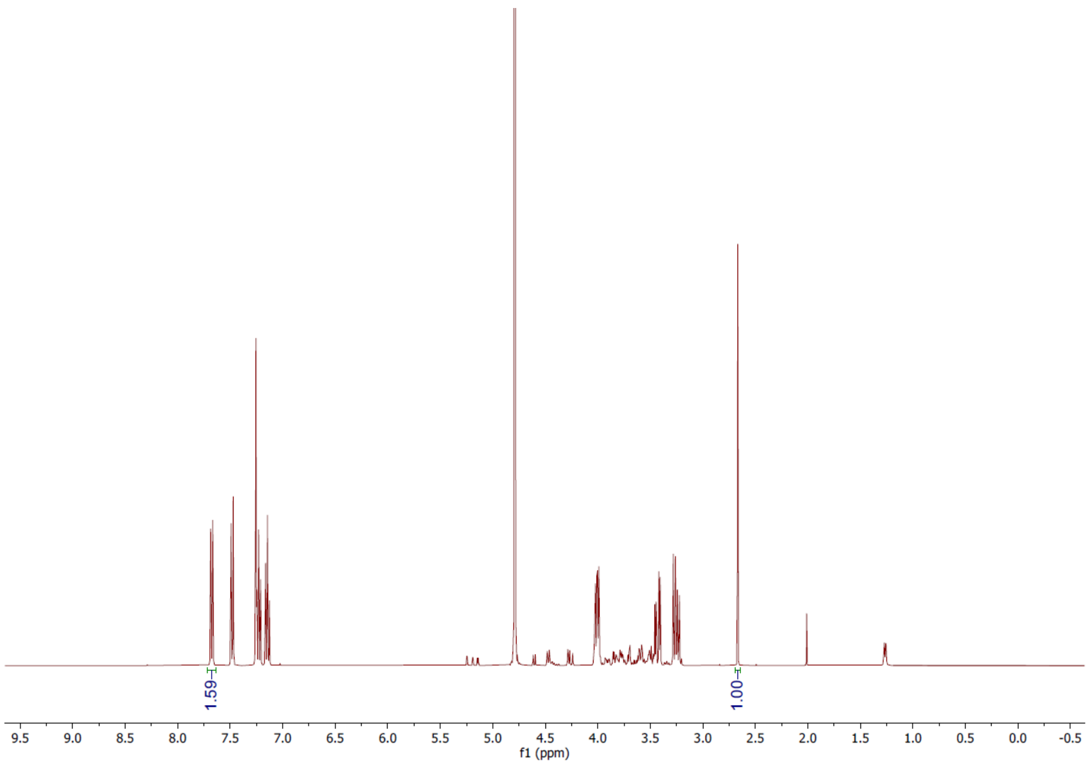

^{1}H NMR (400 MHz, D_2O) of **entry 5** at t = 0 h.

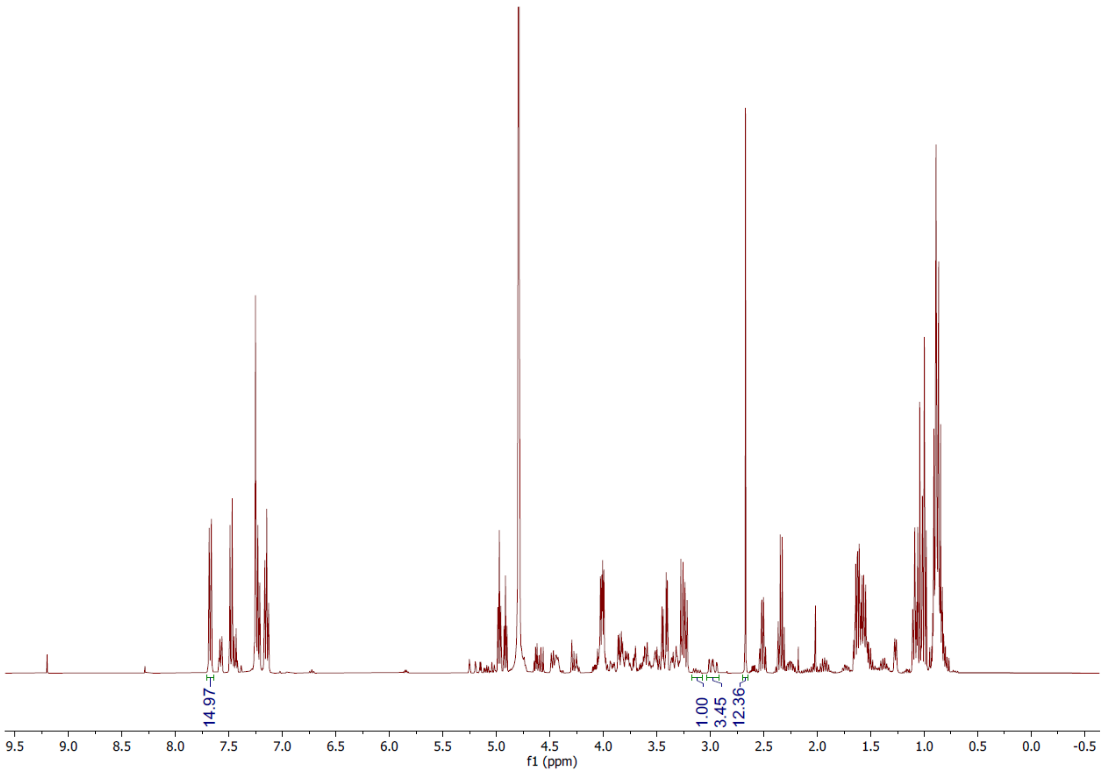

^{1}H NMR (400 MHz, D_2O) of **entry 5** at t = 48 h.

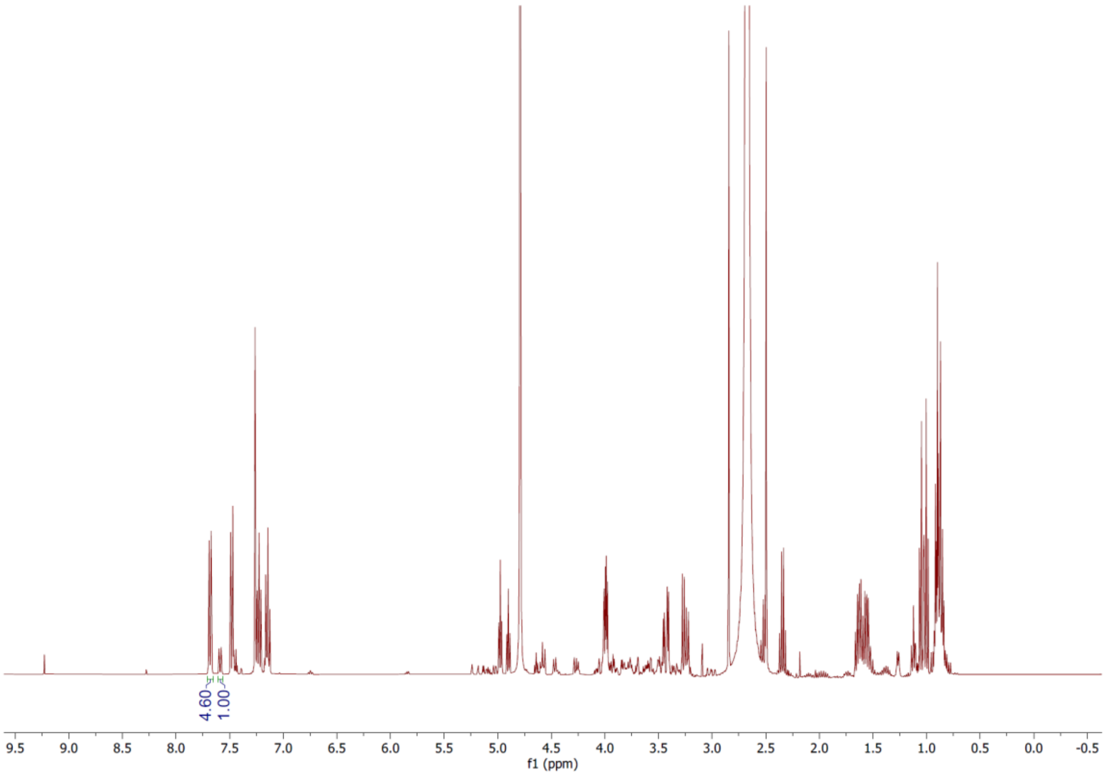

^{1}H NMR (400 MHz, D_2O) of **entry 6** at t = 48 h.

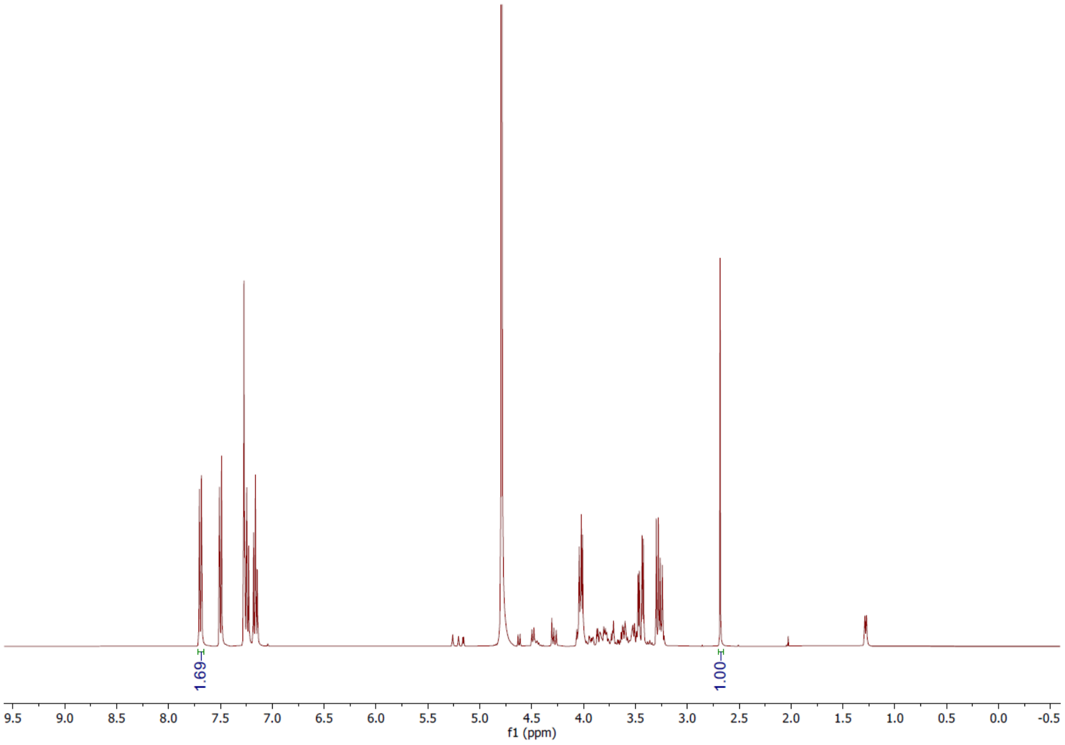

^{1}H NMR (400 MHz, D_2O) of **entry 7** at t = 0 h.

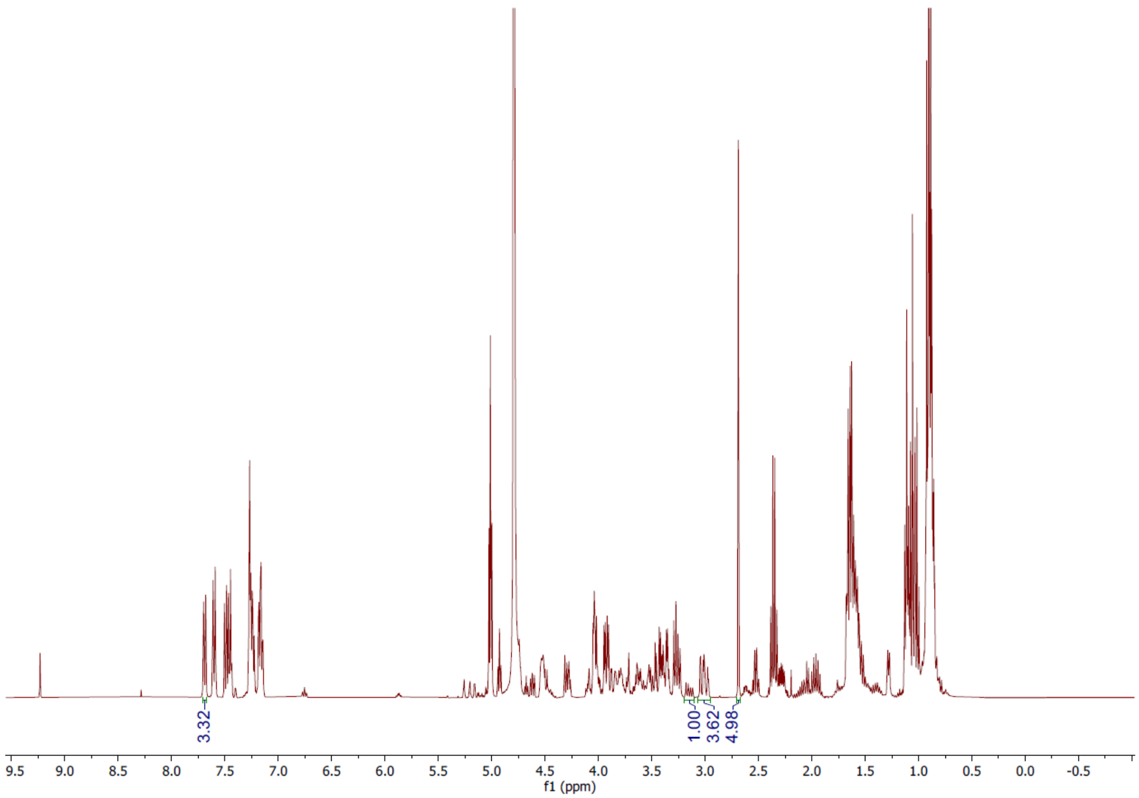

^{1}H NMR (400 MHz, D_2O) of **entry 7** at t = 168 h.

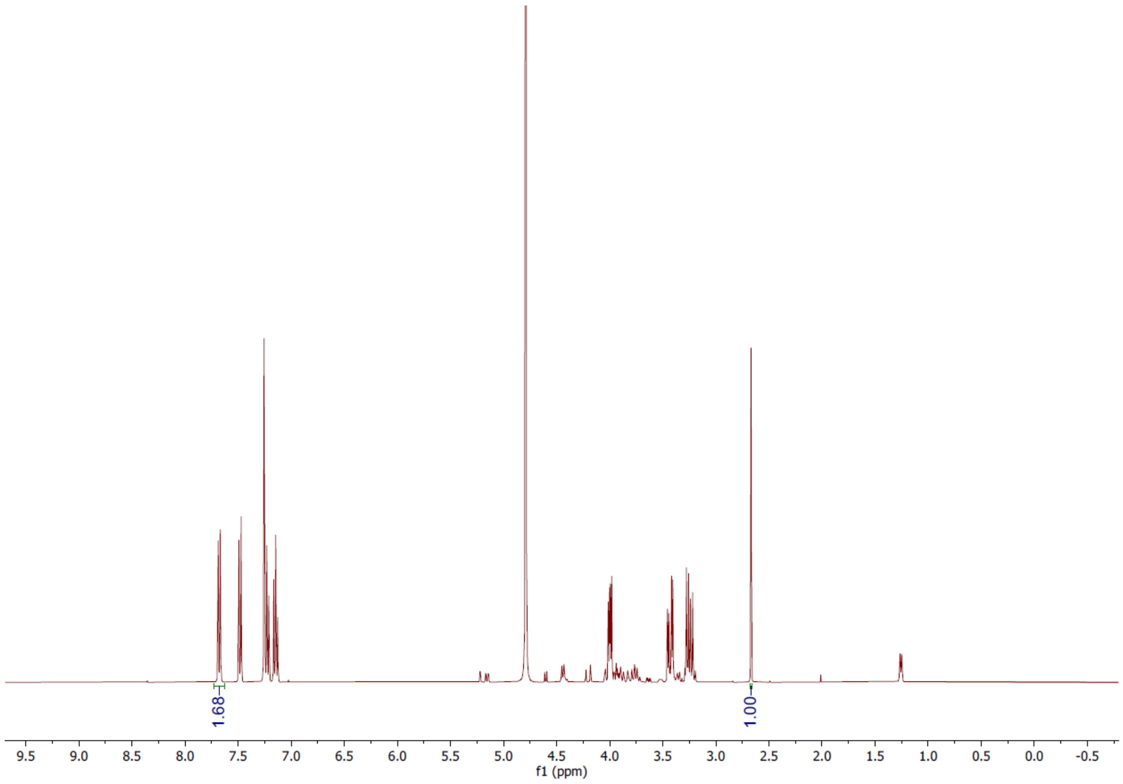

^{1}H NMR (400 MHz, D_2O) of **entry 8** at t = 0 h.

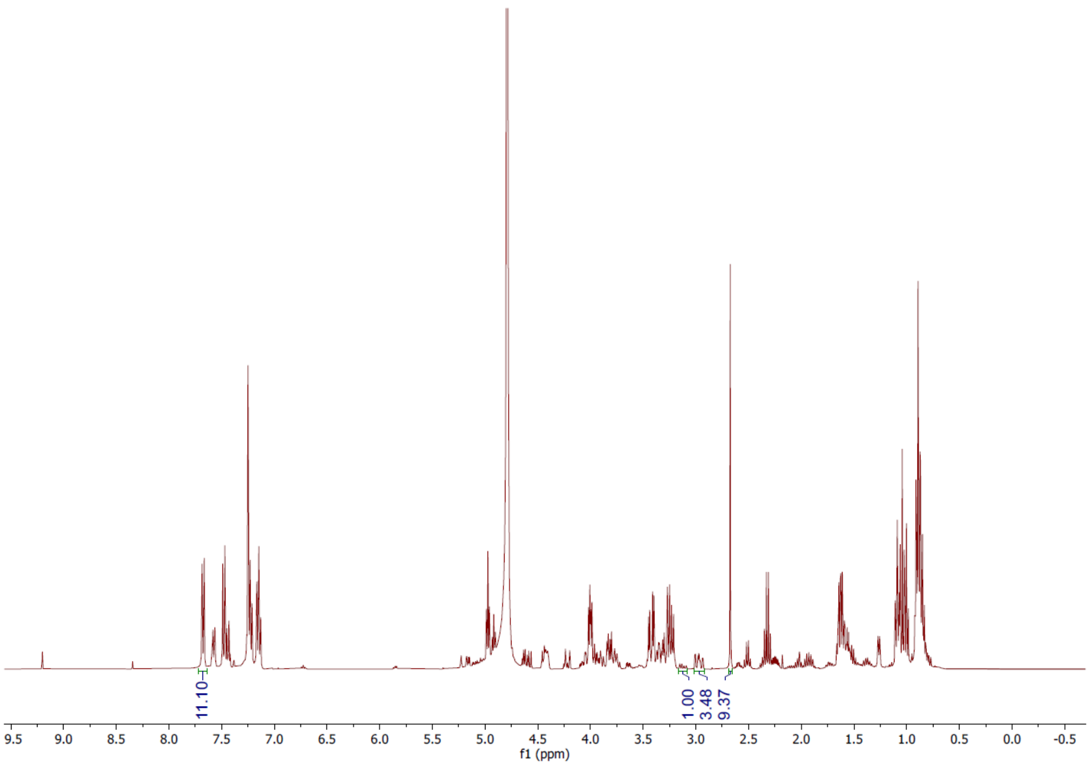

^{1}H NMR (400 MHz, D_2O) of **entry 8** at t = 168 h.

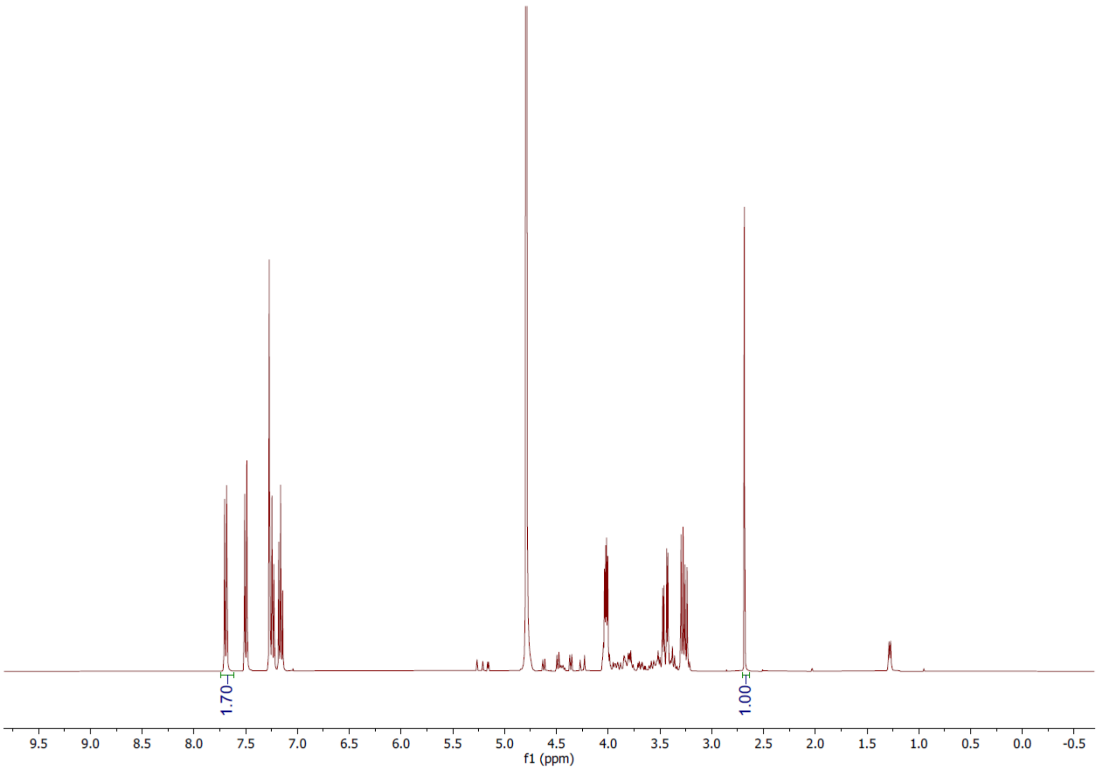

^1H NMR (400 MHz, D_2O) of **entry 9** at t = 0 h.

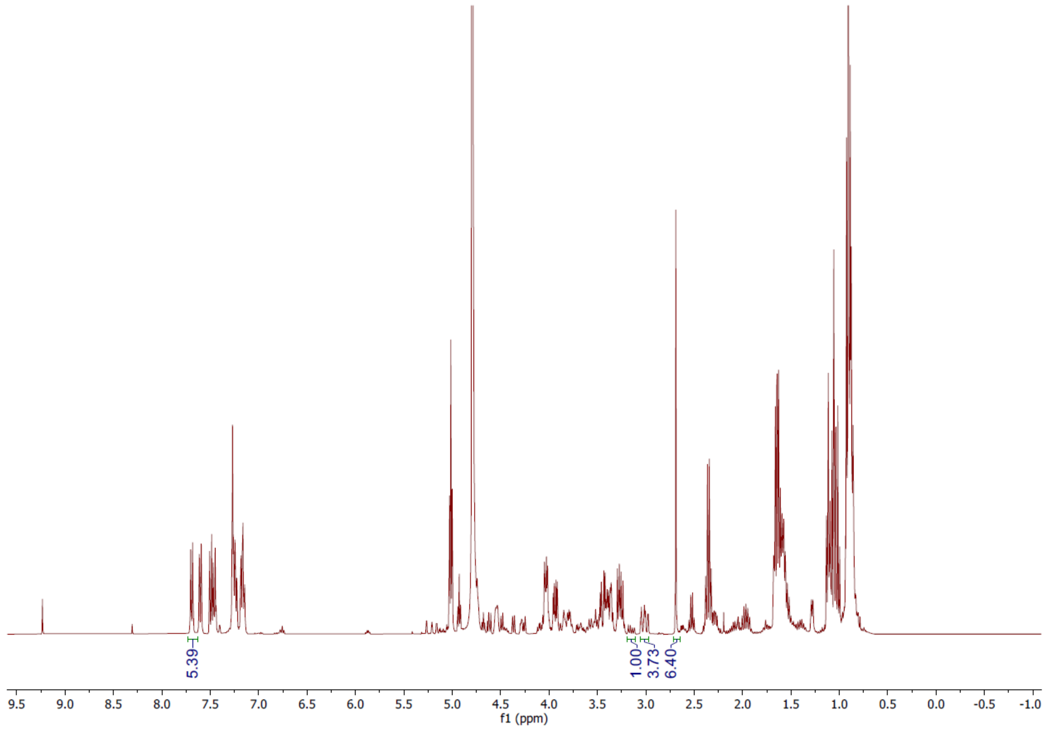

^1H NMR (400 MHz, D_2O) of **entry 9** at t = 168 h.

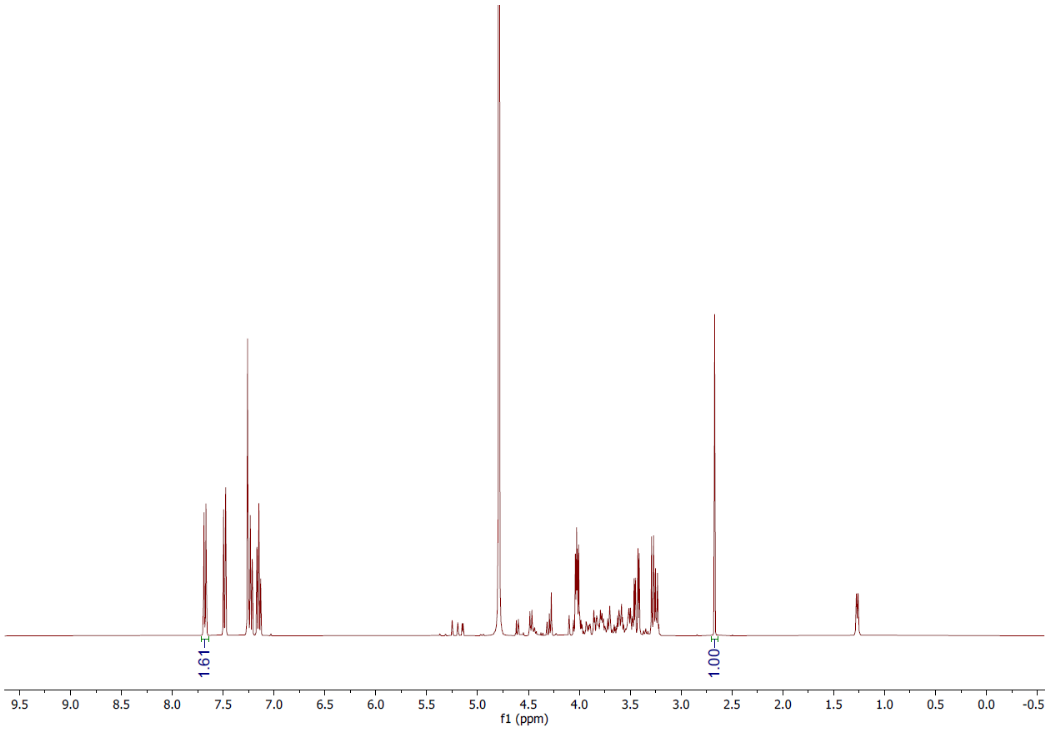

^1H NMR (400 MHz, D_2O) of **entry 10** at t = 0 h.

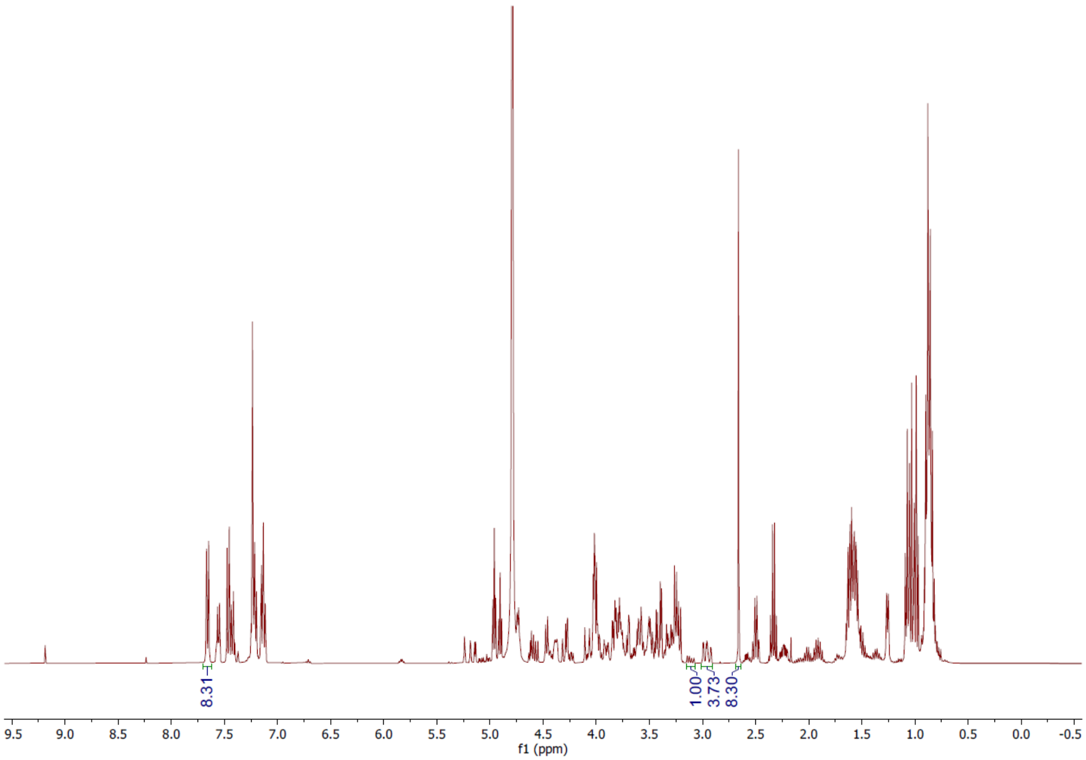

^{1}H NMR (400 MHz, D_2O) of **entry 10** at t = 48 h.

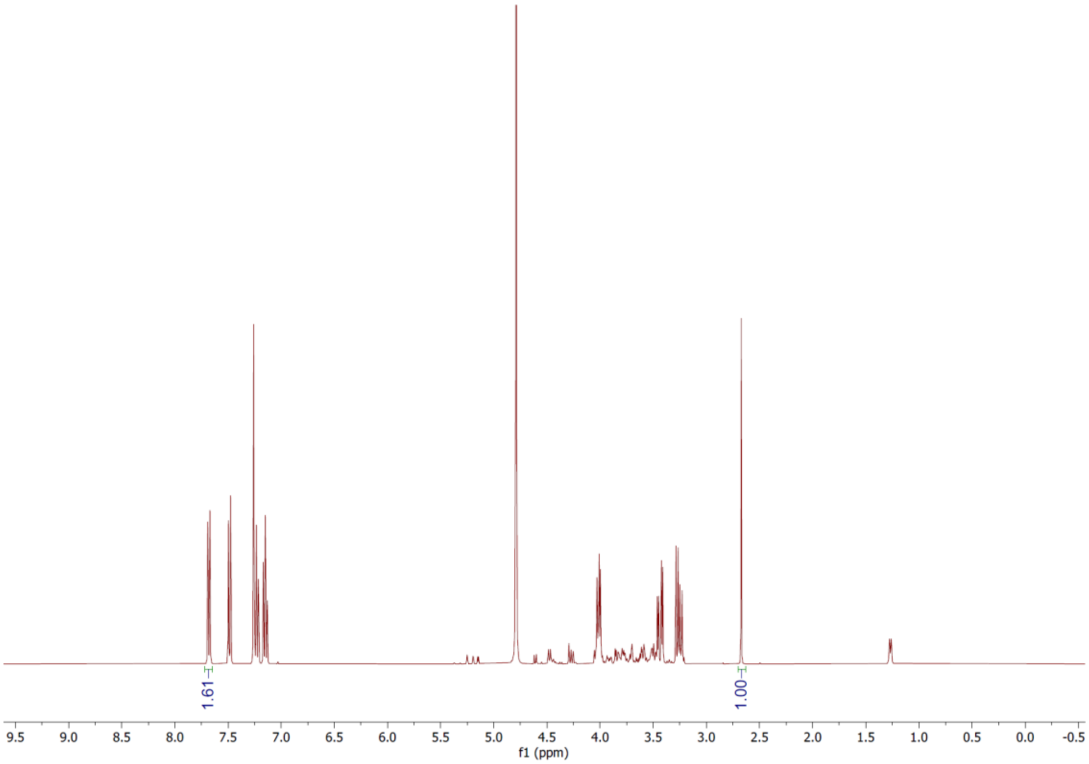

^{1}H NMR (400 MHz, D_2O) of **entry 11** at t = 0 h.

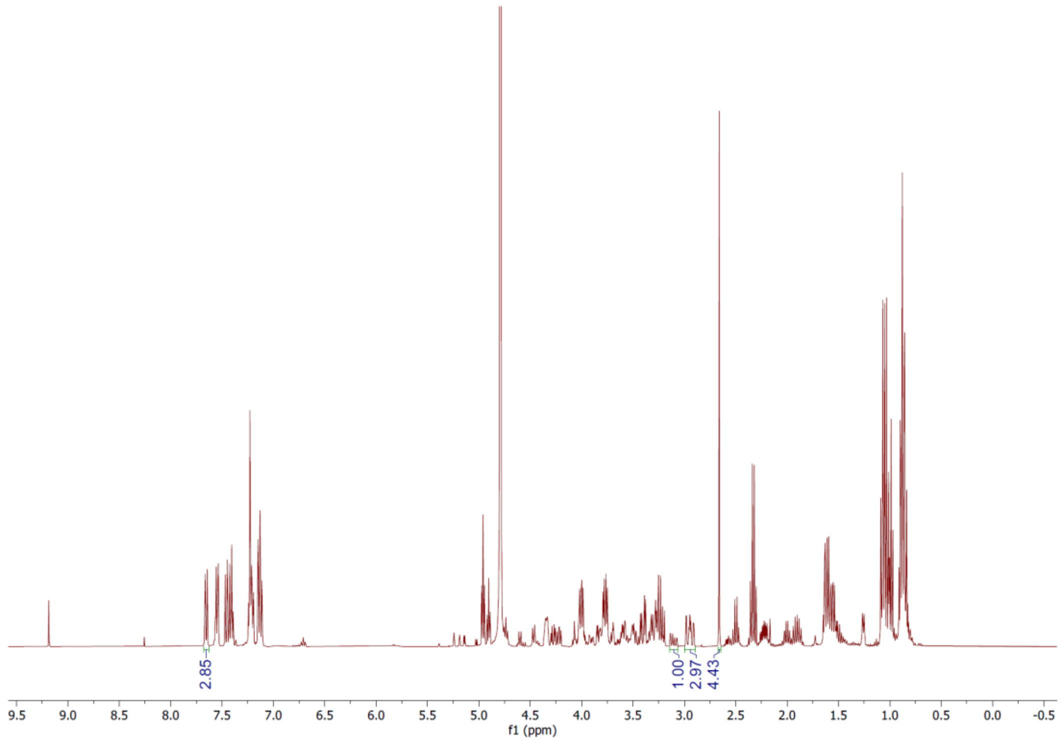

^{1}H NMR (400 MHz, D_2O) of **entry 11** at t = 48 h.

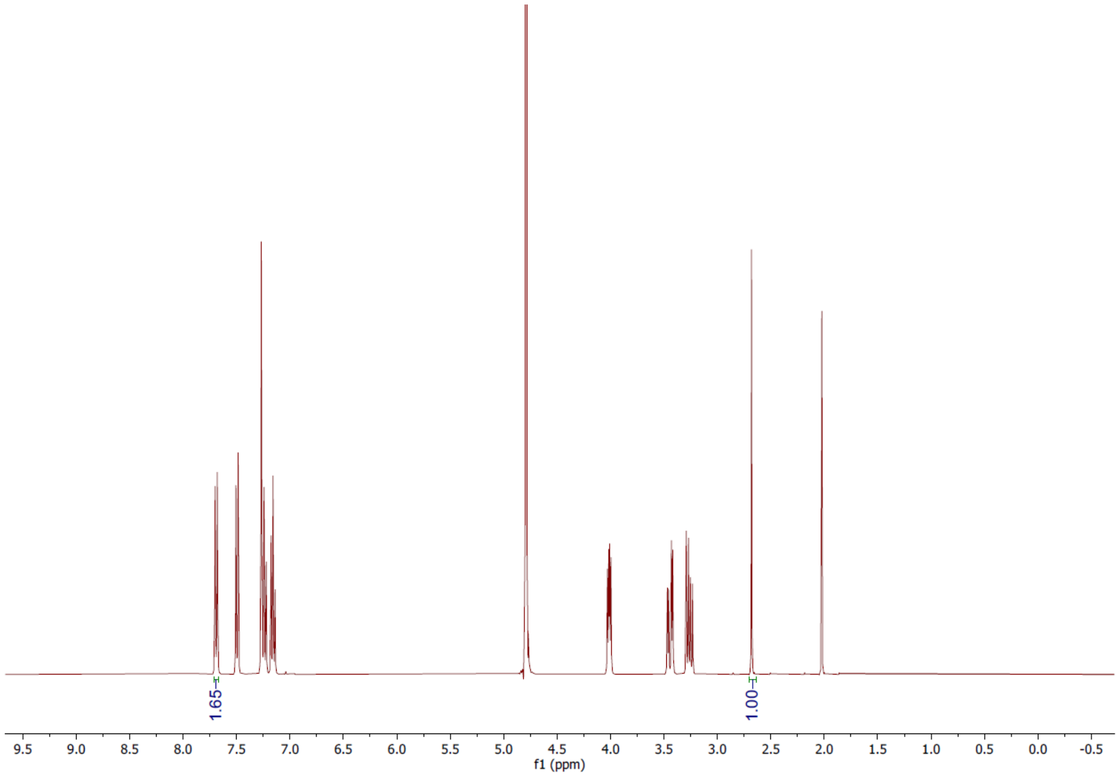

^{1}H NMR (400 MHz, D_2O) of **entry 13** at t = 0 h.

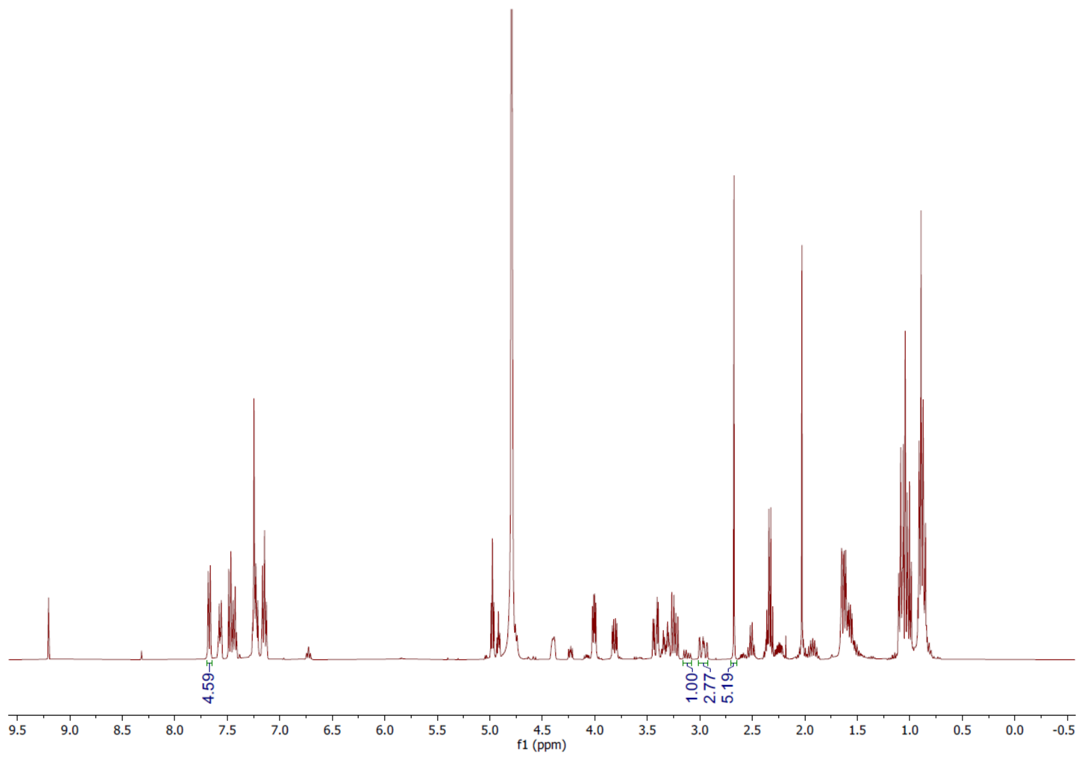

^{1}H NMR (400 MHz, D_2O) of **entry 13** at t =72 h.

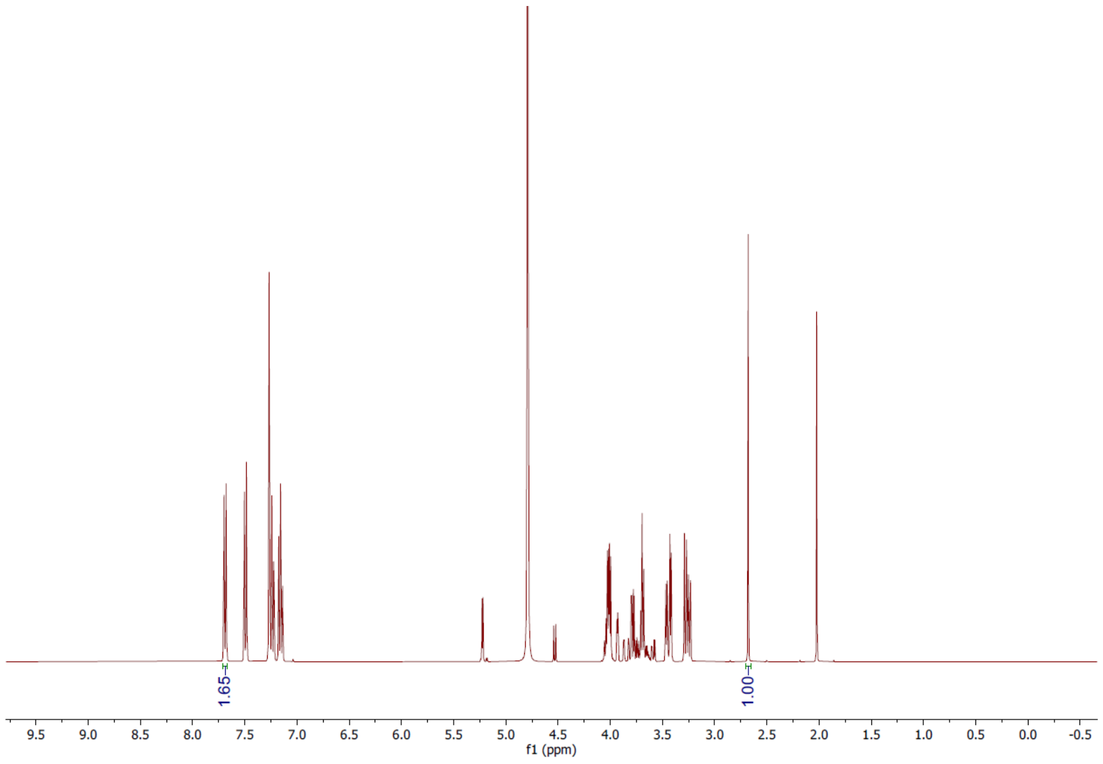

^{1}H NMR (400 MHz, D_2O) of **entry 14** at t = 0 h.

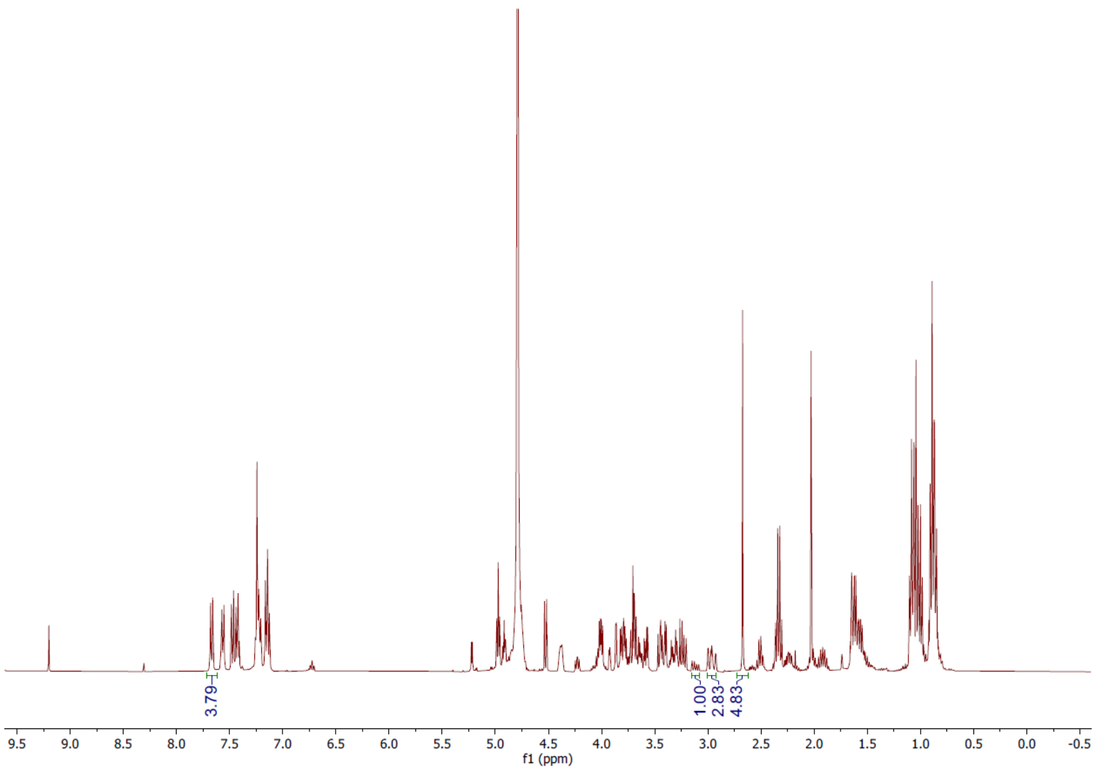

^{1}H NMR (400 MHz, D_2O) of **entry 14** at t = 72 h.

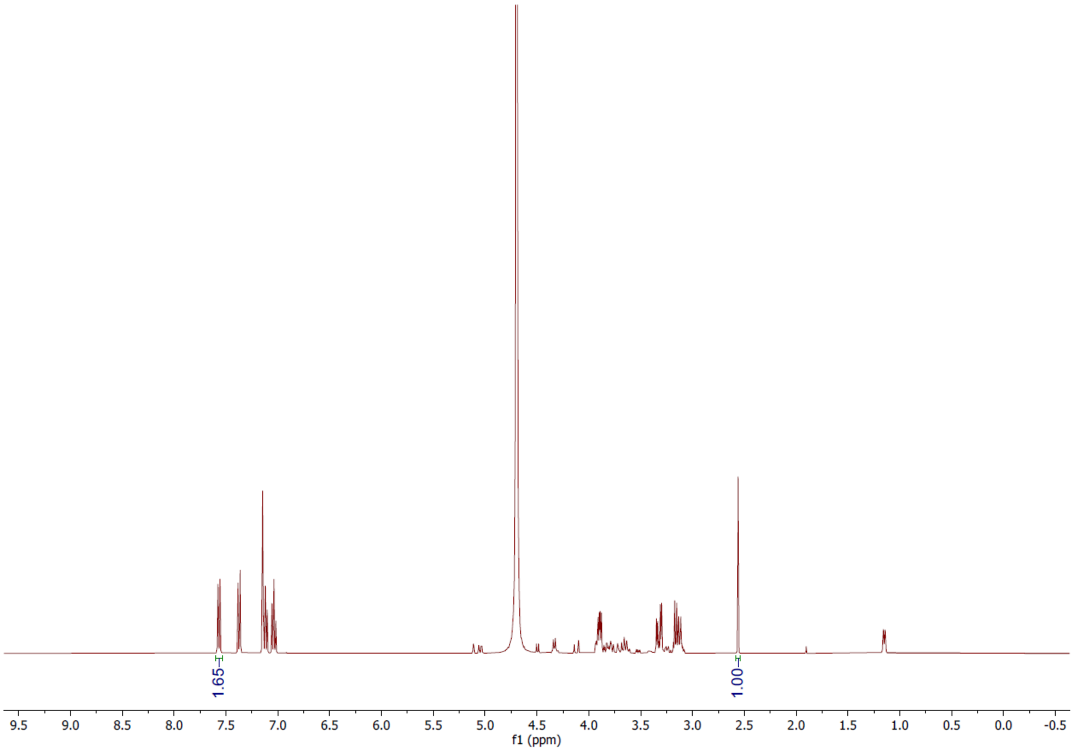

^{1}H NMR (400 MHz, D_2O) of **entry** 15 at t = 0 h.

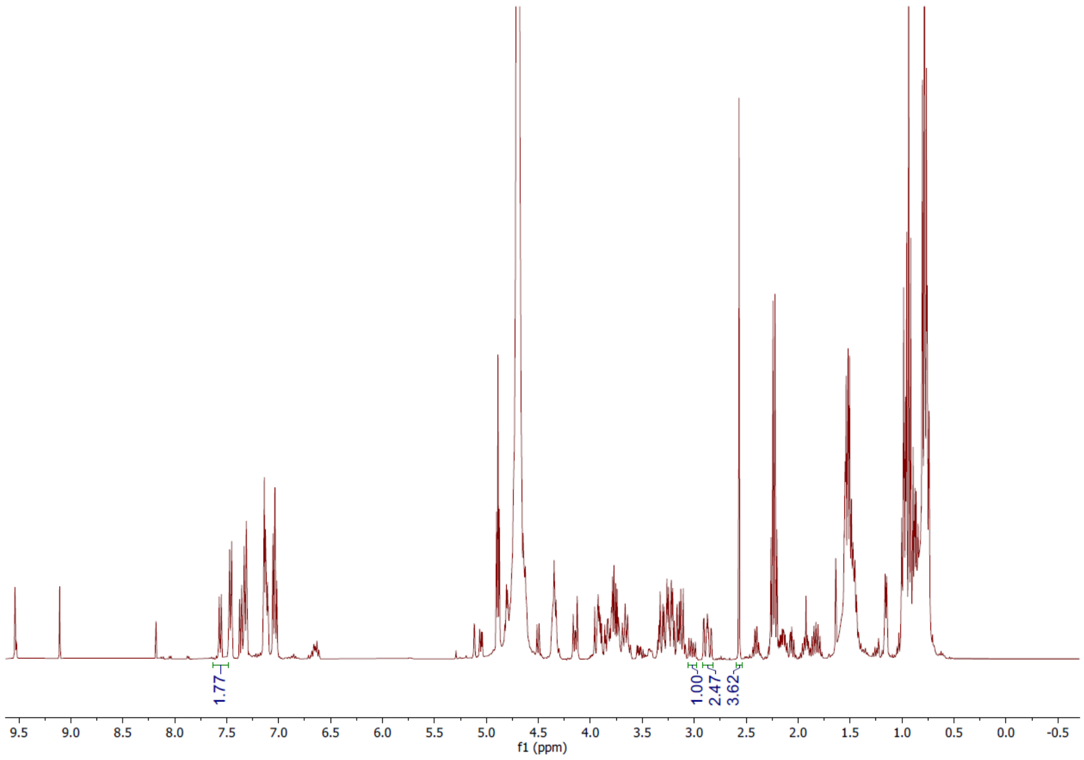

^{1}H NMR (400 MHz, D2O) of **entry** 15 at t = 72 h.

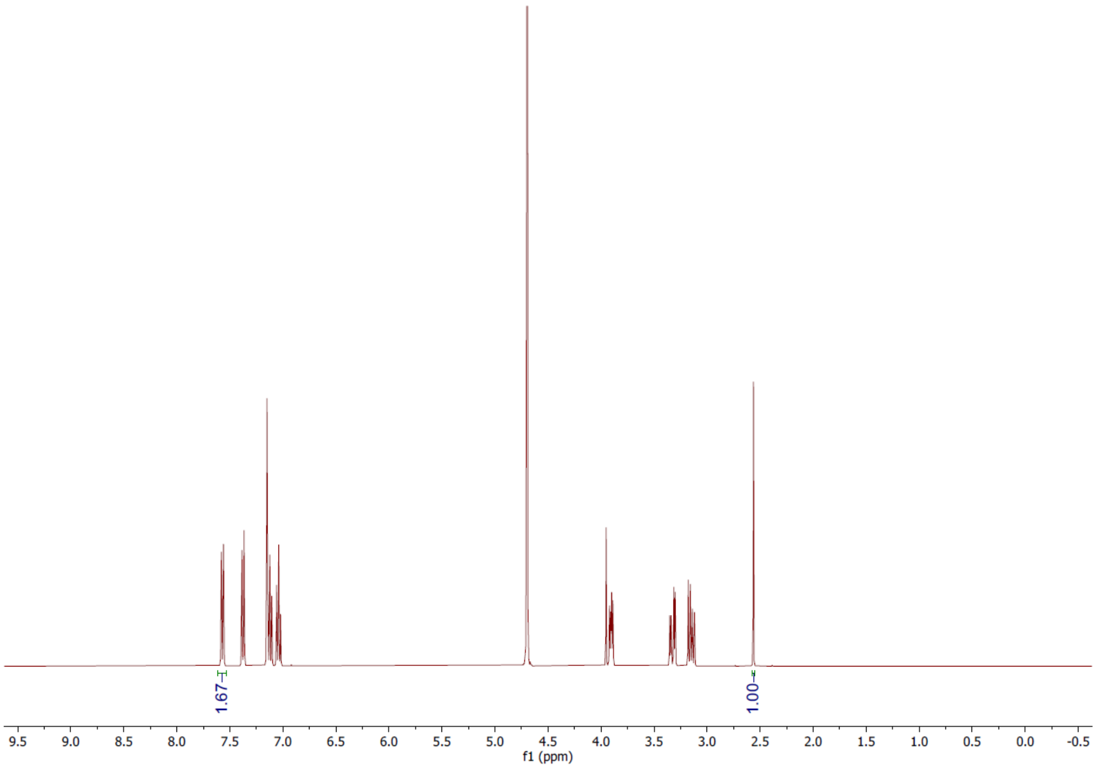

^{1}H NMR (400 MHz, D_2O) of **entry** 16 at t = 0 h.

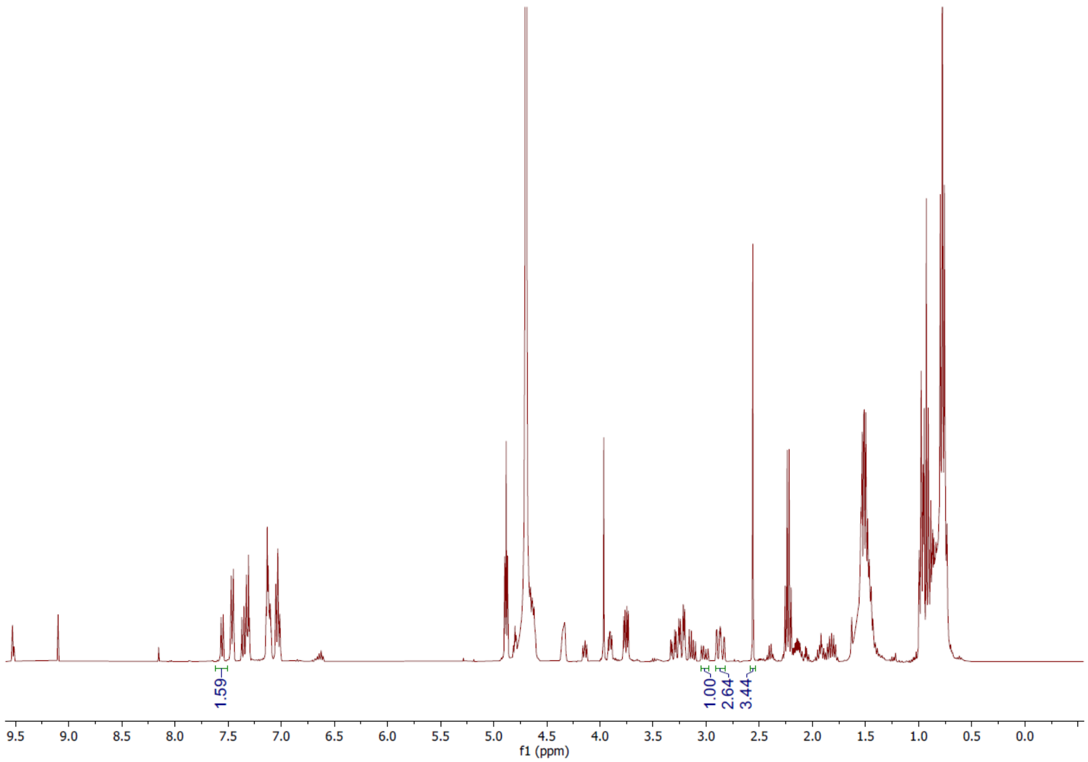

^1H NMR (400 MHz, D_2O) of **entry** 16 at t = 72 h.

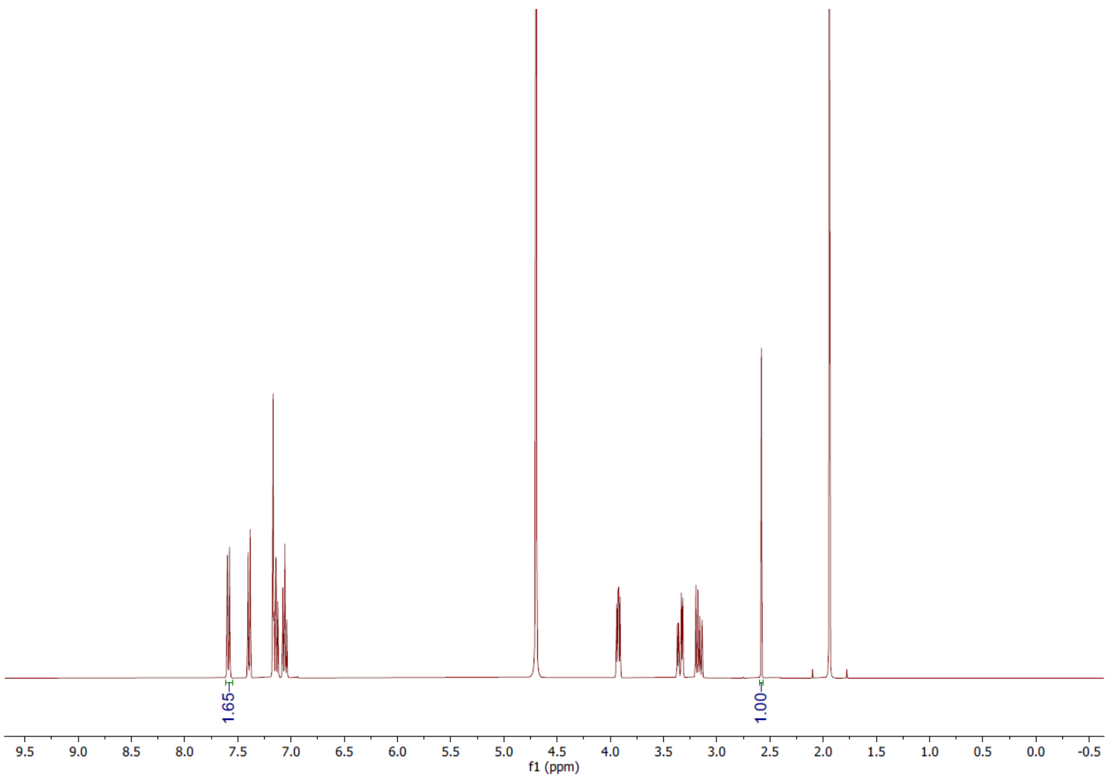

^1H NMR (400 MHz, D_2O) of **entry** 17 at t = 0 h.

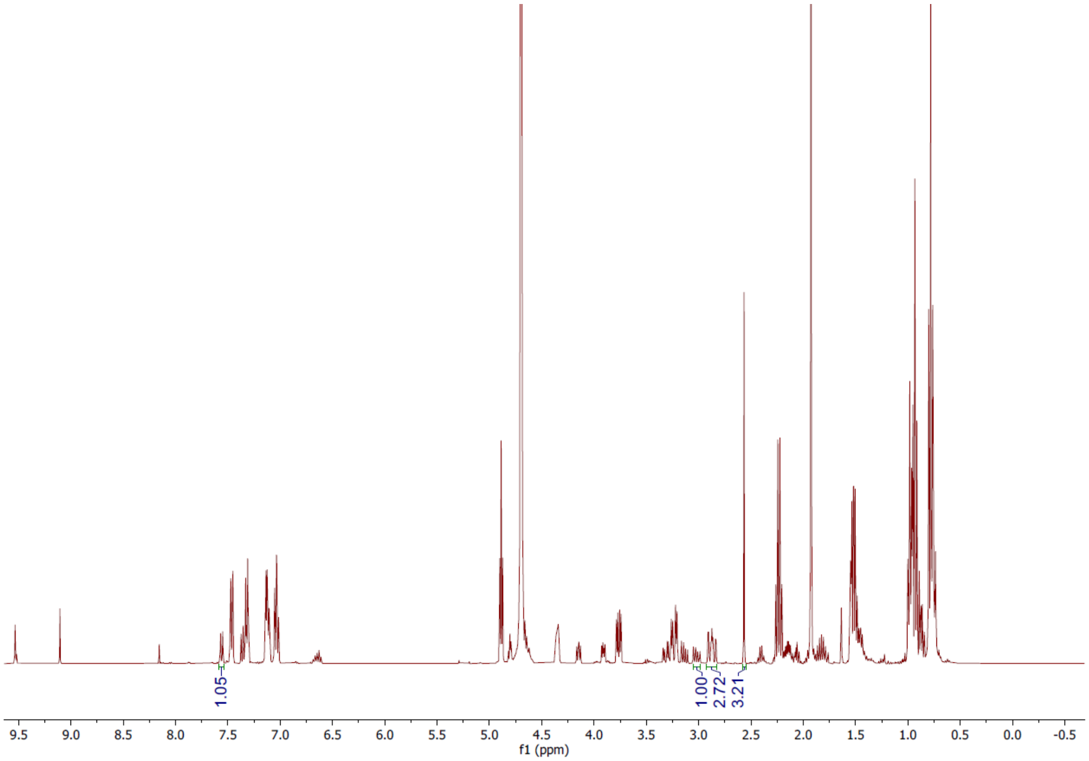

^{1}H NMR (400 MHz, D_2O) of **entry** 17 at t = 72 h.

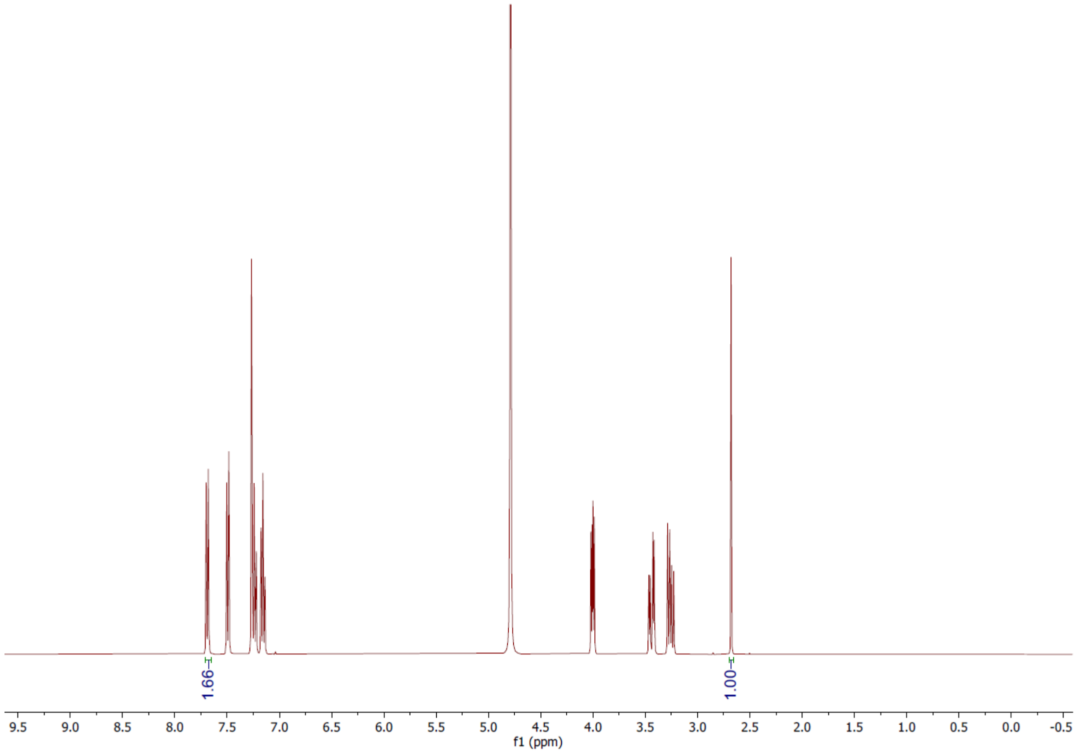

^{1}H NMR (400 MHz, D_2O) of **entry** 18 at t = 0 h.

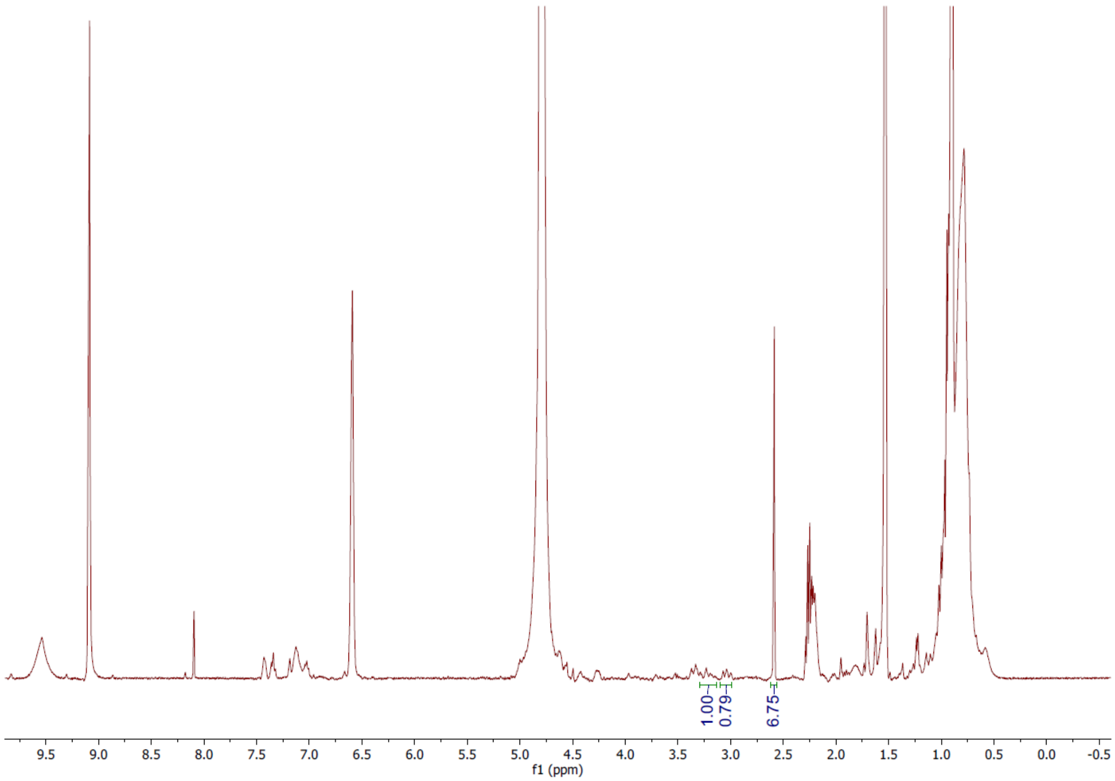

^{1}H NMR (400 MHz, D_2O) of **entry** 18 at t = 72 h.

5.4 Kinetic investigation

5.4.1 General method

Five experiments (**A** and **B**, **E** (in water), **C** and **D** (in DPBS pH= 7.4 buffer)) were carried out in parallel using the optimized conditions. **A**: L- Tryptophan (5.1 mg, 0.025 mmol) and **4mer-IV** (3.6 mg, 0.005 mmol) were dissolved in D_2O (0.4 mL, containing DMSO (6.5 mM, 0.18 µL) as internal standard). Propionaldehyde (22 µL, 0.3 mmol) was added to the reaction at 37 °C. **B**: L-Tryptophan (5.1 mg, 0.025 mmol) was dissolved in a AcOH (12.5 mM) solution in D_2O (0.4 mL, containing DMSO (6.5 mM, 0.18 µL) as internal standard). Propionaldehyde (22 µL, 0.3 mmol) was added to the reaction at 37 °C. **C**: L- Tryptophan (5.1 mg, 0.025 mmol) and **4mer-IV** (3.6 mg, 0.005 mmol) were dissolved in DPBS buffer (0.4 mL, containing DMSO (6.5 mM, 0.18 µL) as internal standard). Propionaldehyde (22 µL, 0.3 mmol) was added to the reaction at 37 °C. **D**: L-Tryptophan (5.1 mg, 0.025 mmol) was dissolved in a AcOH (12.5 mM) solution in DPBS buffer (0.4 mL, containing DMSO (6.5 mM, 0.18 µL) as internal standard). Propionaldehyde (22 µL, 0.3 mmol) was added to the reaction at 37 °C. E: L- Tryptophan (5.1 mg, 0.025 mmol) was dissolved in a glycolic acid (12.5 mM) solution in D2O (0.4 mL, containing DMSO (6.5 mM, 0.18 µL) as

internal standard). Propionaldehyde (22 µL, 0.3 mmol) was added to the reaction at 37 °C. Every group was further divided into four subgroups and monitored at 15 h, 30 h, 45 h, 72 h by ^{1}H NMR.

Yield / % Time / h	A	B	C	D	E
0	0	0	0	0	0
15	25	9	8	5	15
30	40	17	19	11	28
45	52	25	29	21	38
72	70	42	56	41	56

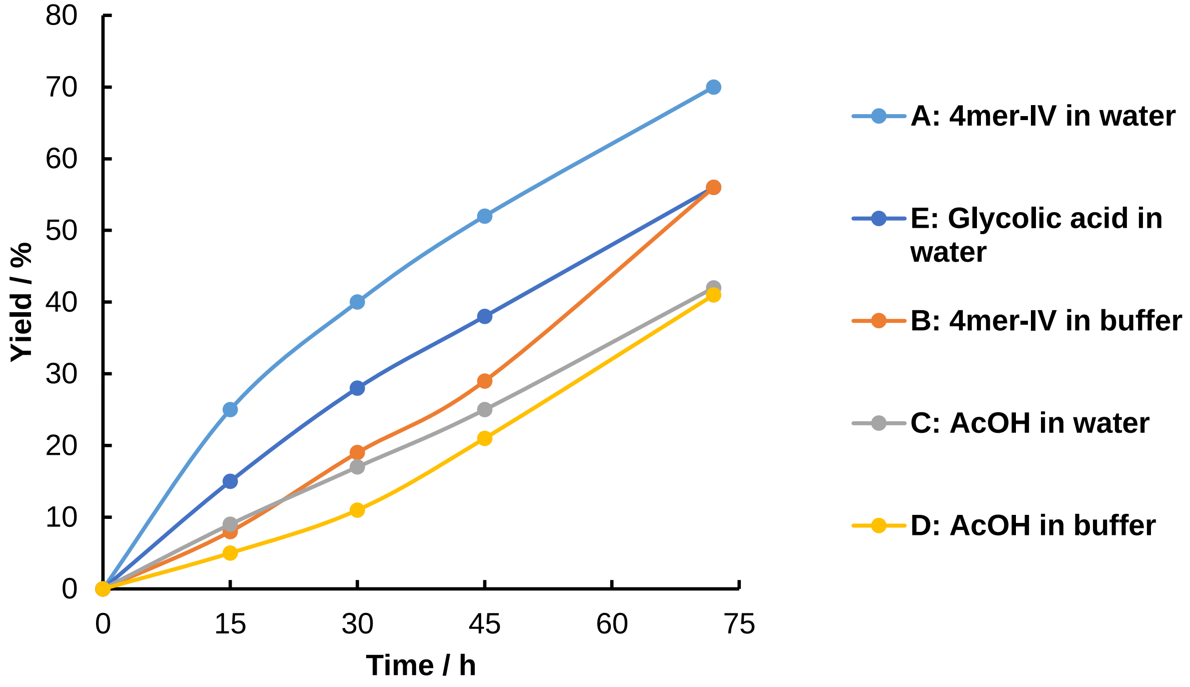

5.4.2 NMR of crude reaction

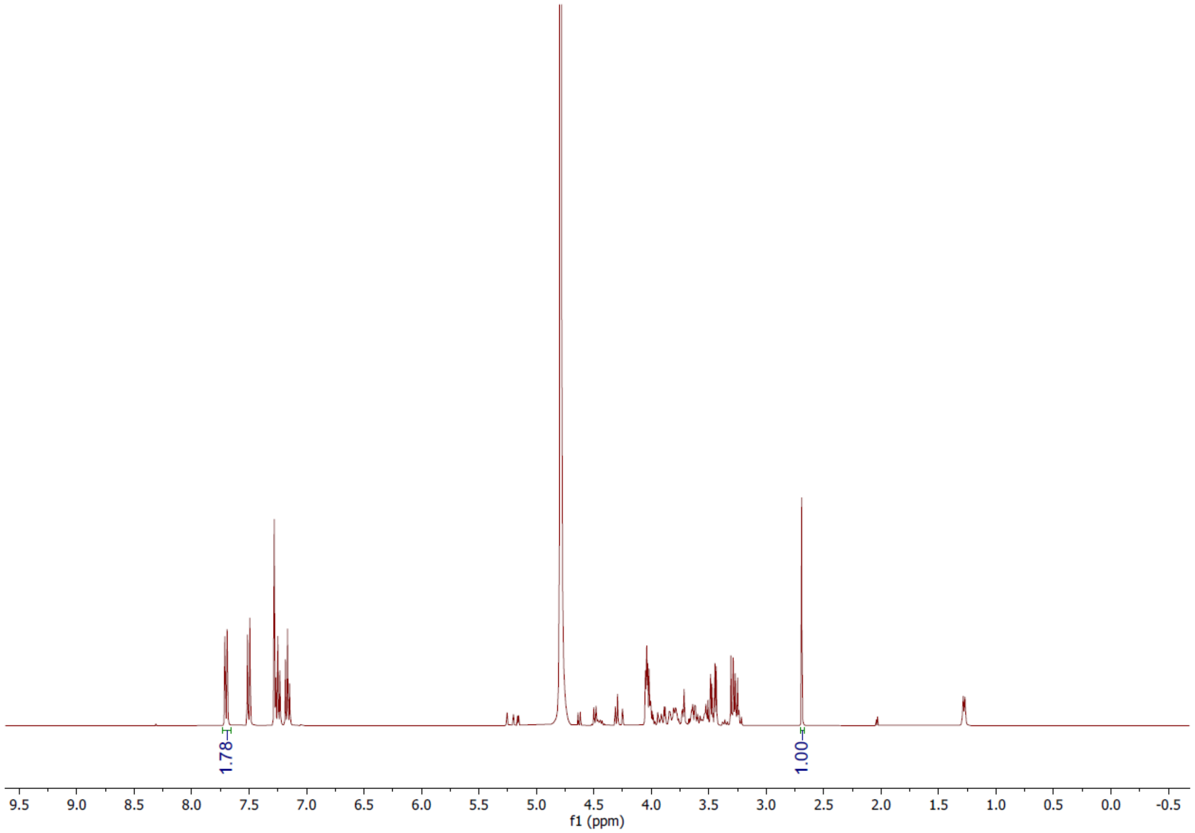

^{1}H NMR (400 MHz, D_2O) of **A** group at t = 0 h.

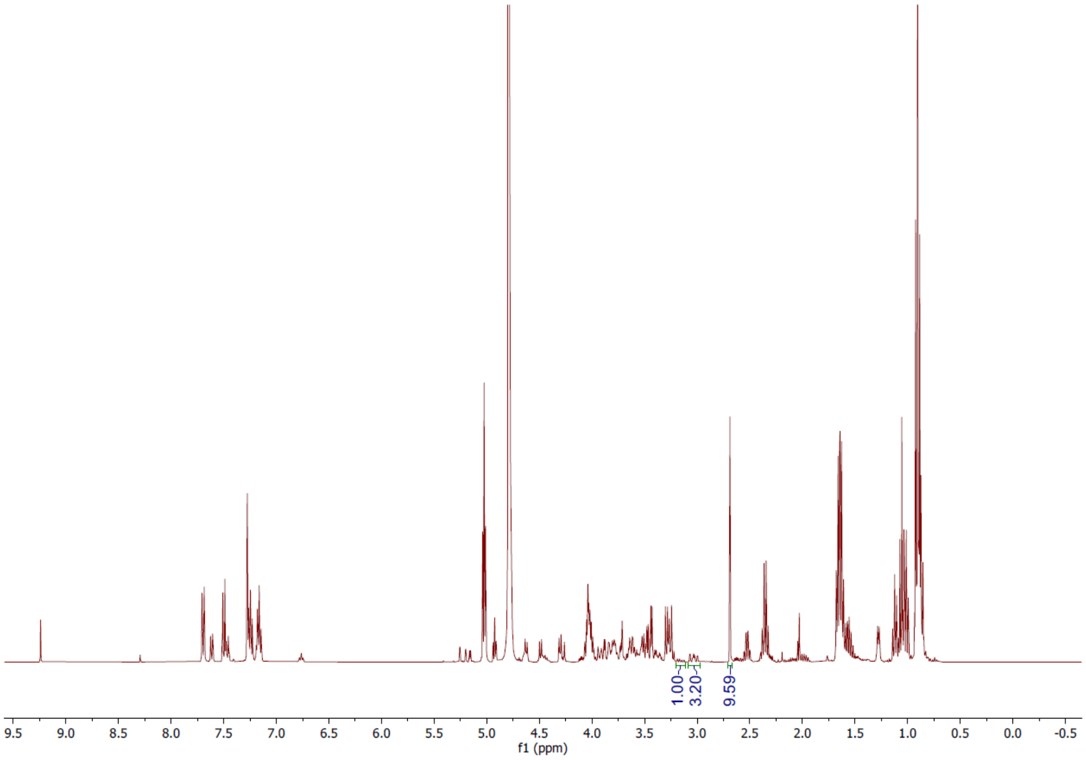

^{1}H NMR (400 MHz, D_2O) of **A** group at t = 15 h.

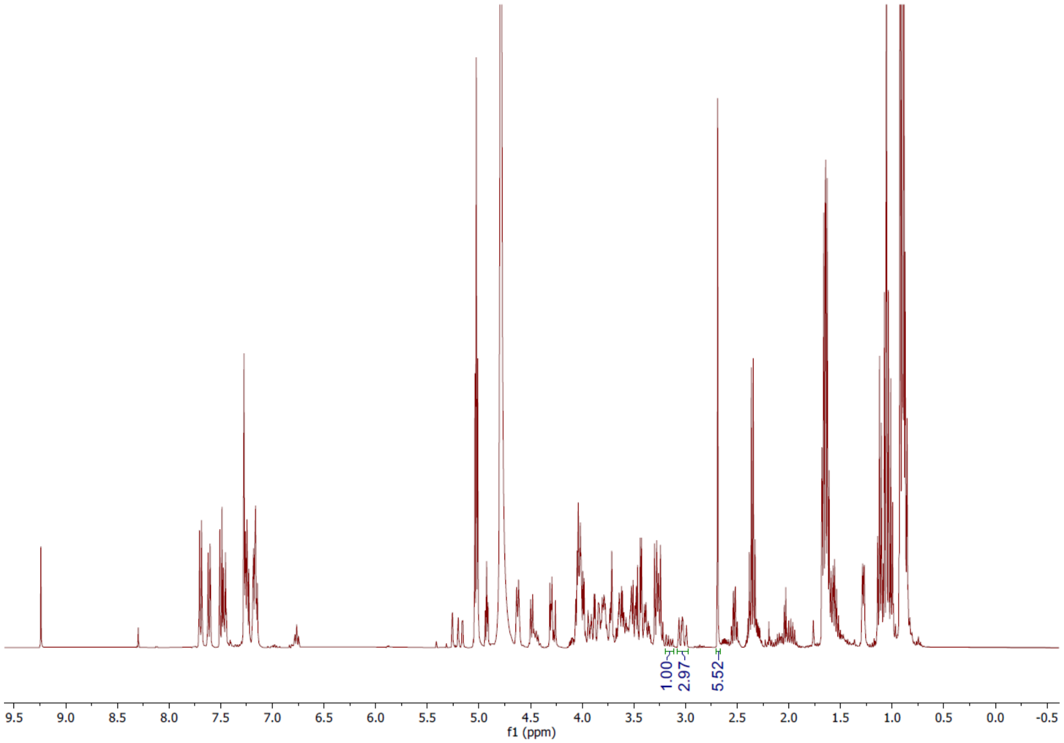

^{1}H NMR (400 MHz, D_2O) of **A** group at t = 30 h.

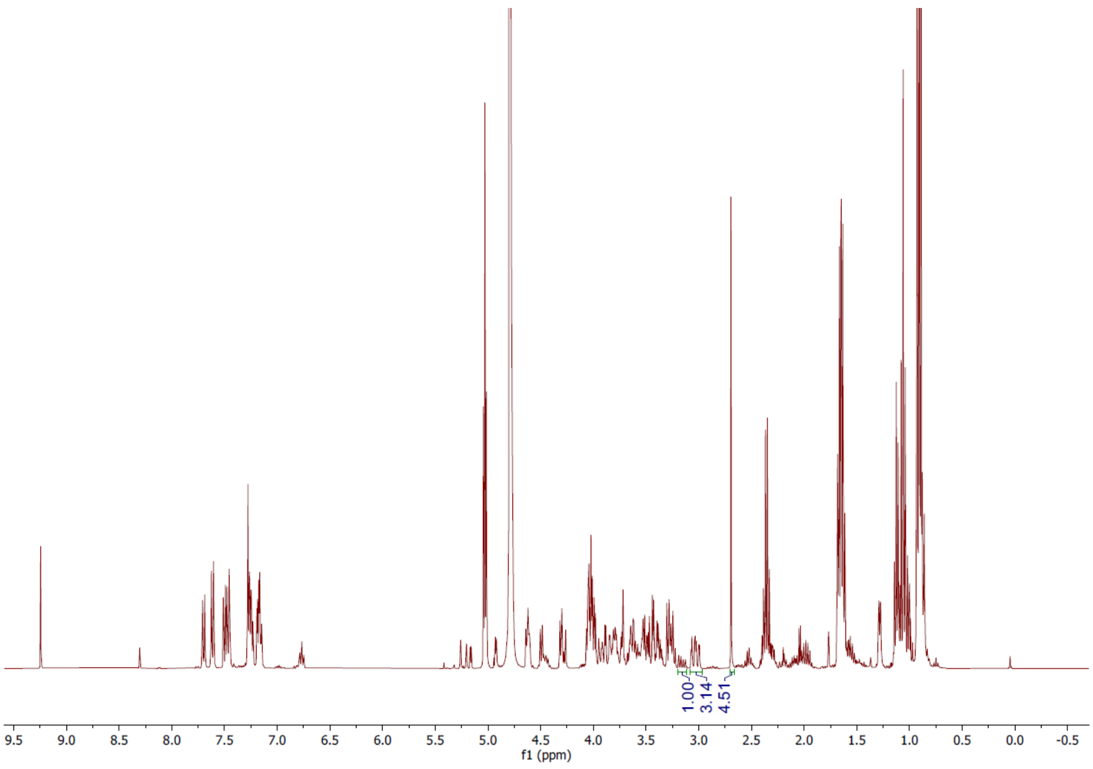

^{1}H NMR (400 MHz, D_2O) of **A** group at t = 45 h.

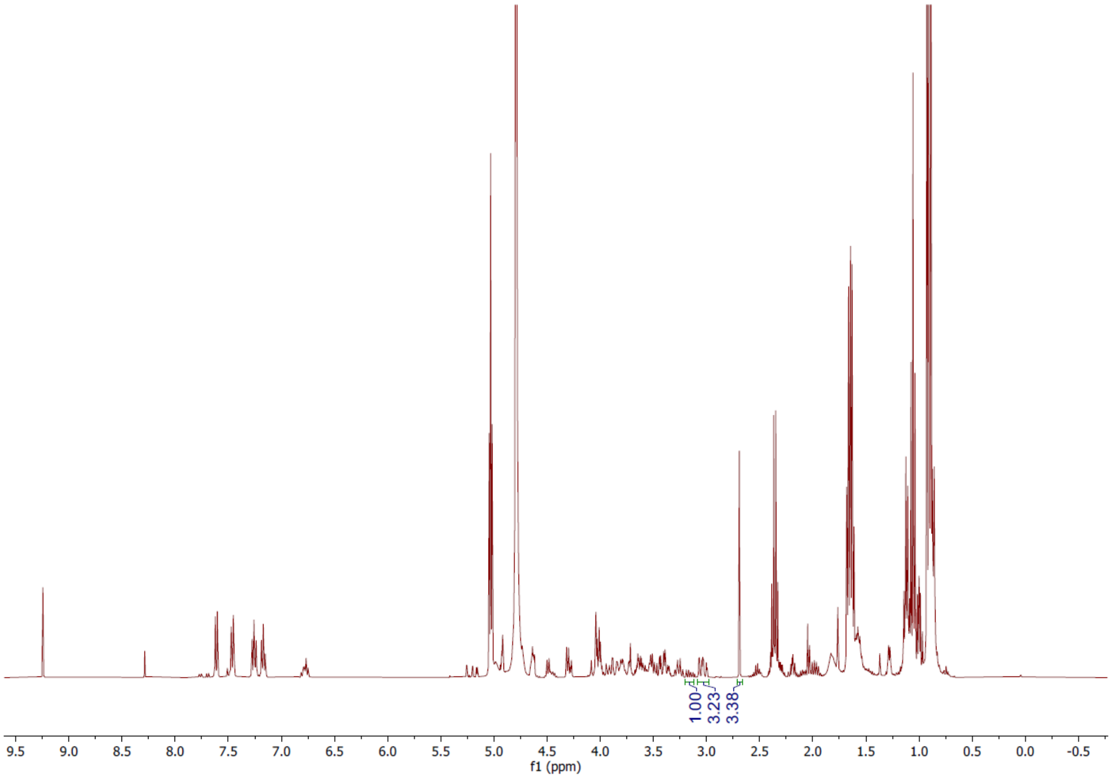

^{1}H NMR (400 MHz, D_2O) of **A** group at t = 72 h.

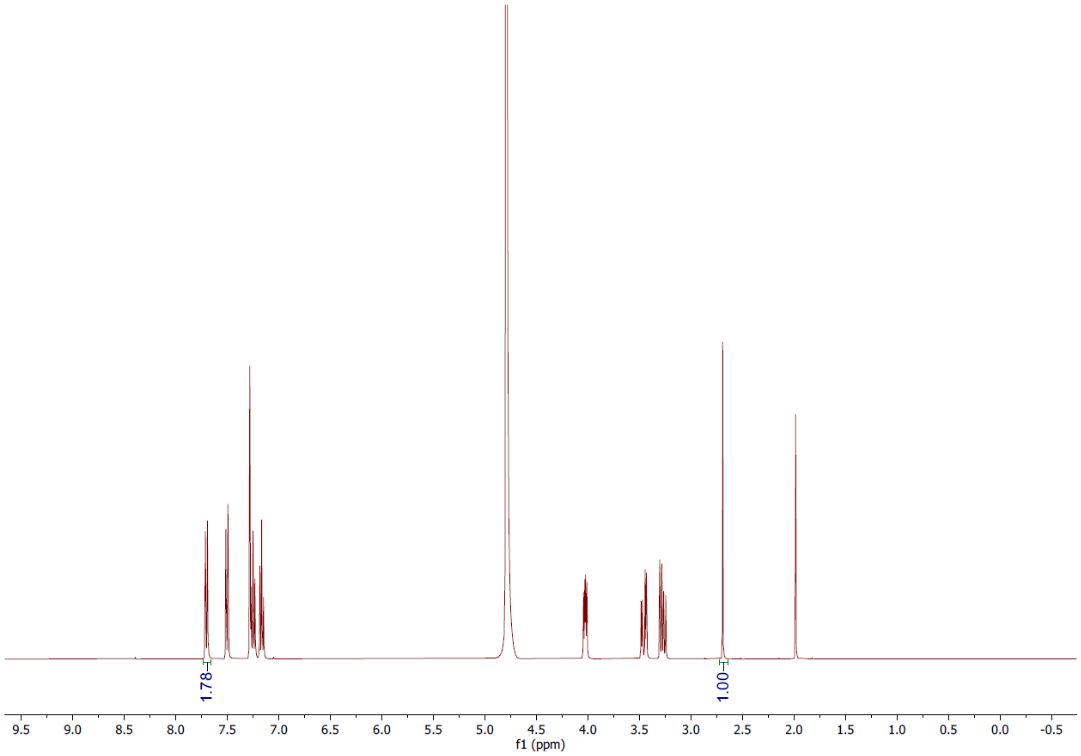

^{1}H NMR (400 MHz, D_2O) of **B** group at t = 0 h.

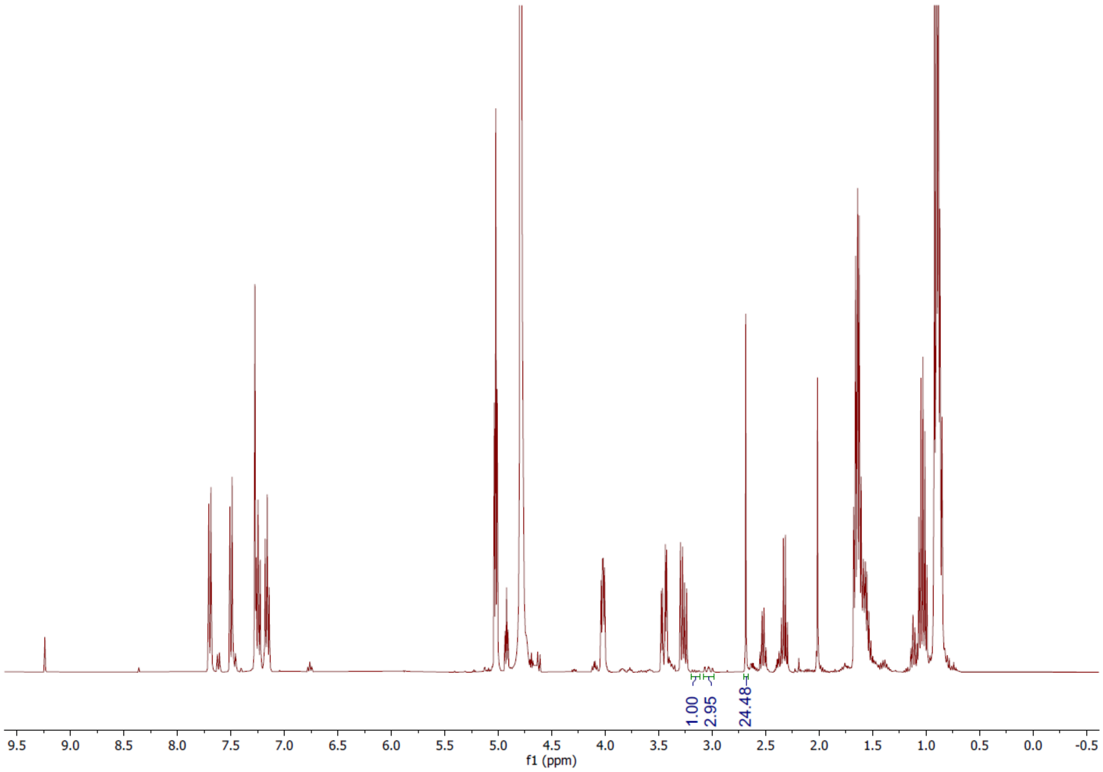

^{1}H NMR (400 MHz, D_2O) of **B** group at t = 15 h.

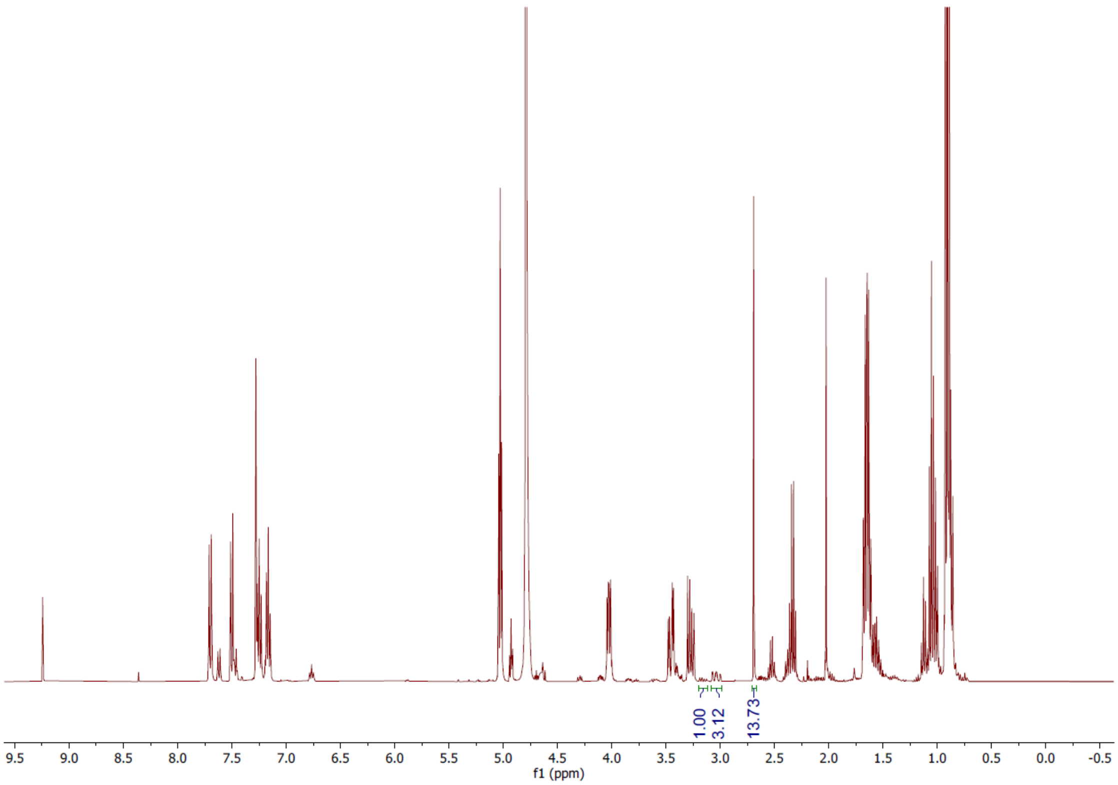

^1H NMR (400 MHz, D_2O) of **B** group at t = 30 h.

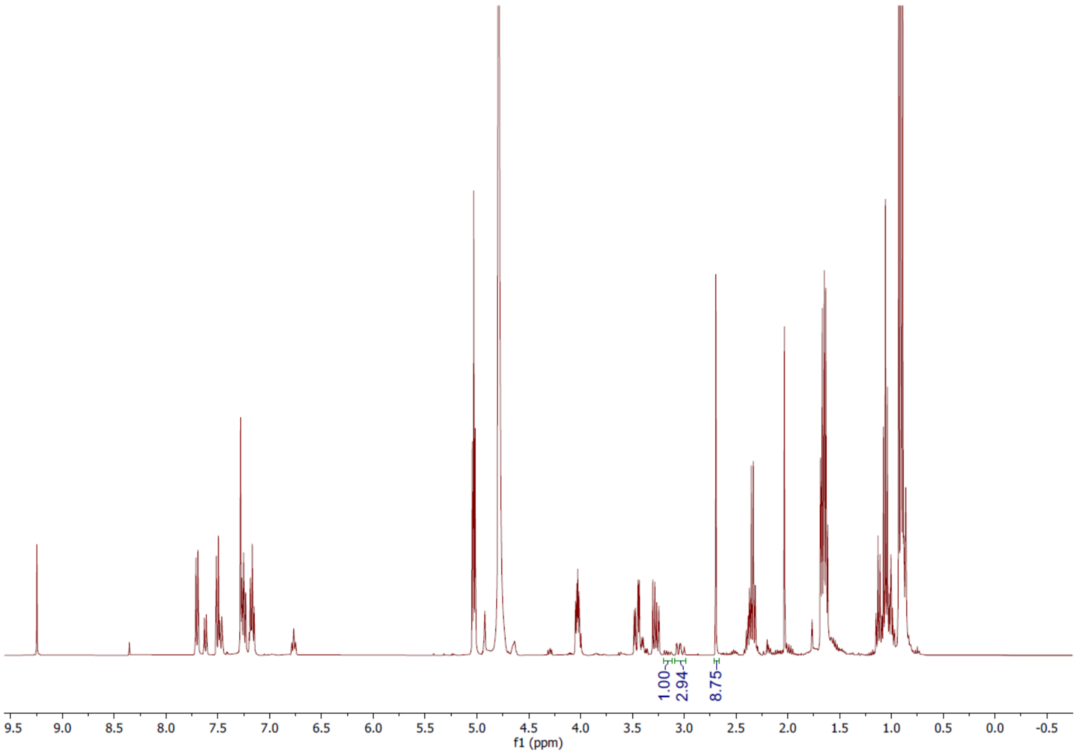

^1H NMR (400 MHz, D_2O) of **B** group at t = 45 h.

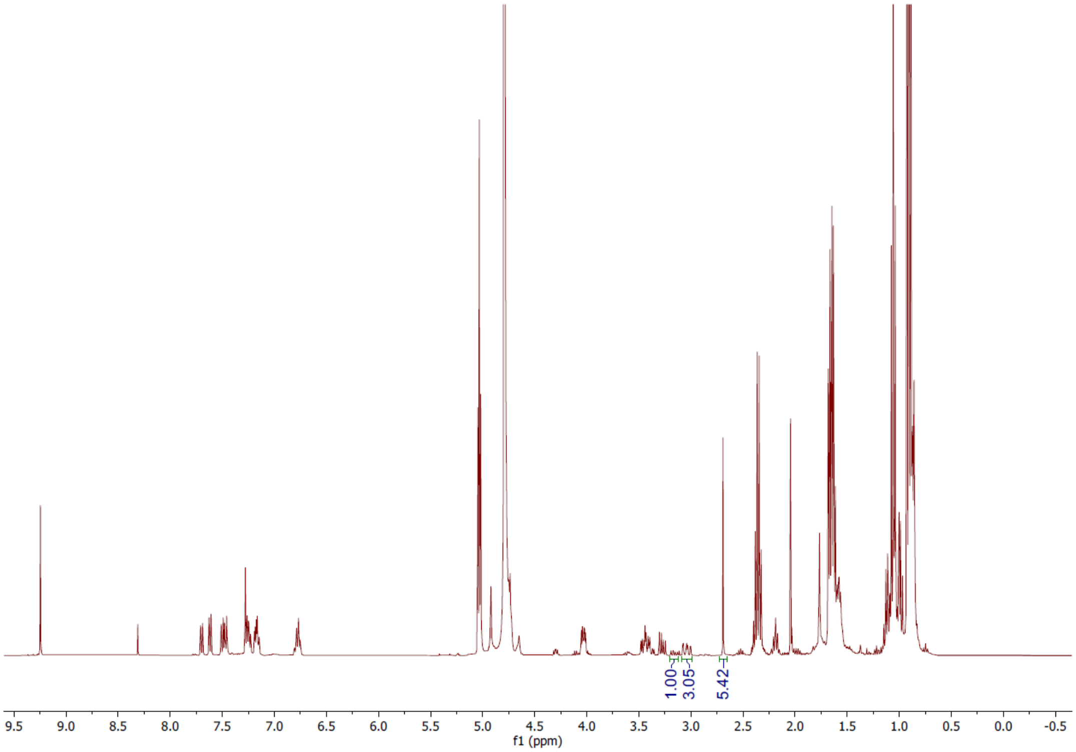

^{1}H NMR (400 MHz, D_2O) of **B** group at t = 72 h.

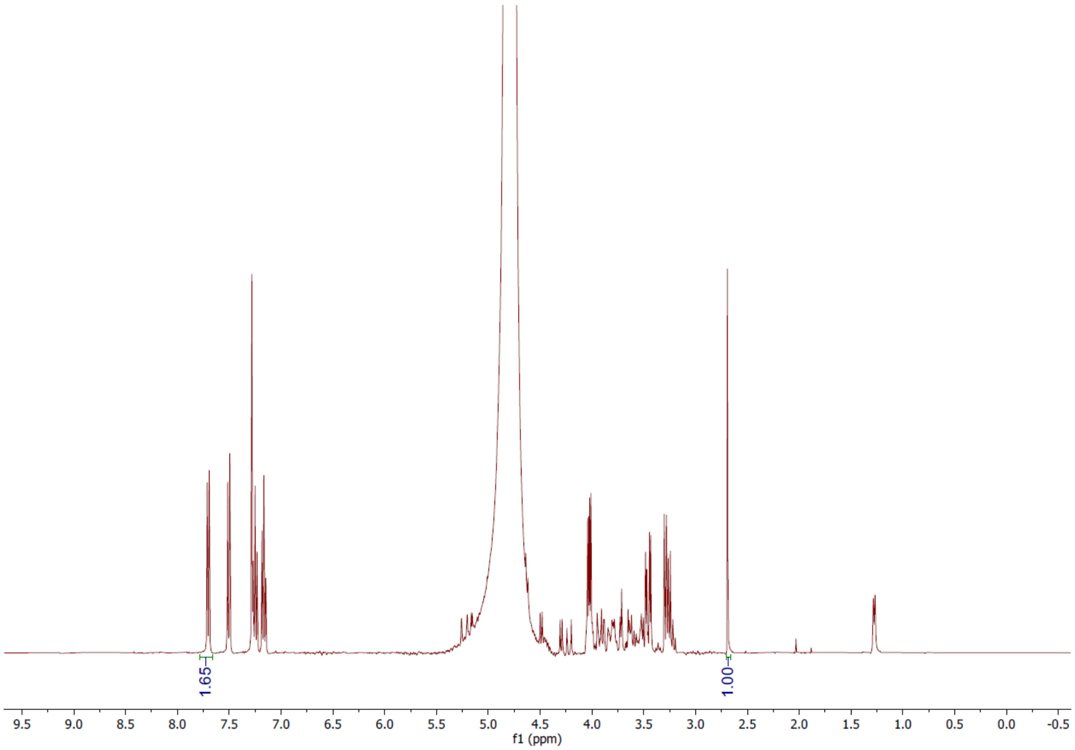

^{1}H NMR (400 MHz, D_2O) of **C** group at t = 0 h.

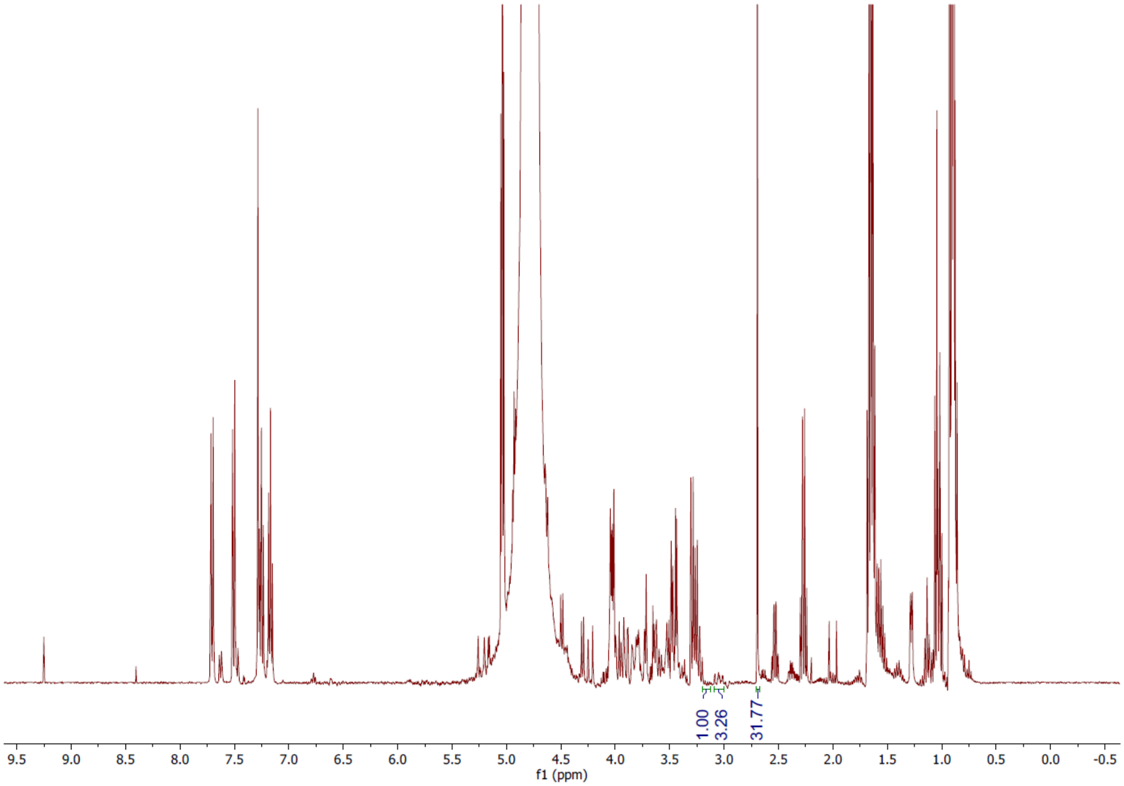

^{1}H NMR (400 MHz, D_2O) of **C** group at t = 15 h.

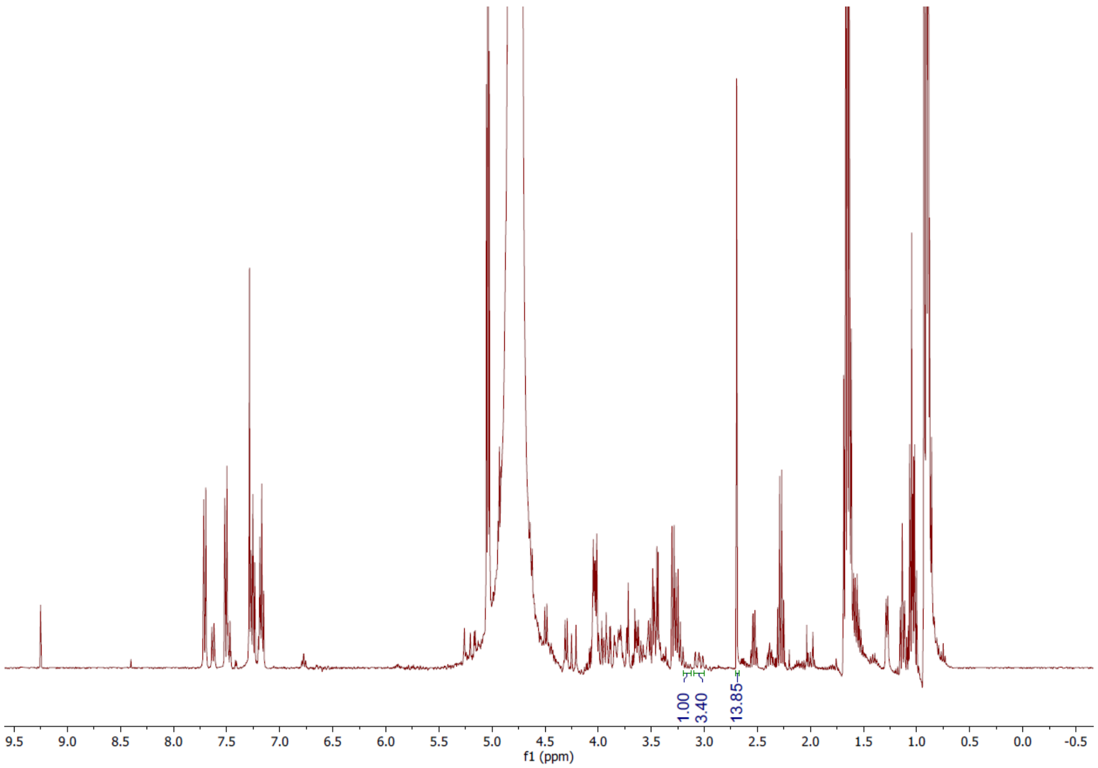

^{1}H NMR (400 MHz, D_2O) of **C** group at t = 30 h.

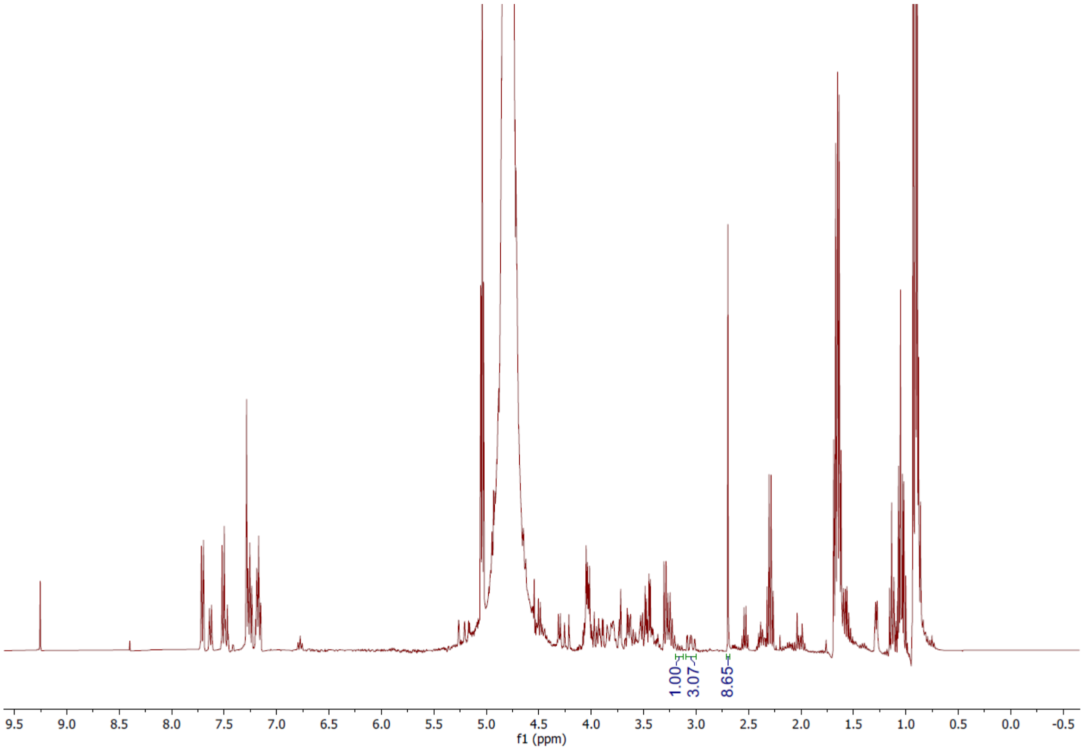

^{1}H NMR (400 MHz, D_2O) of **C** group at t = 45 h.

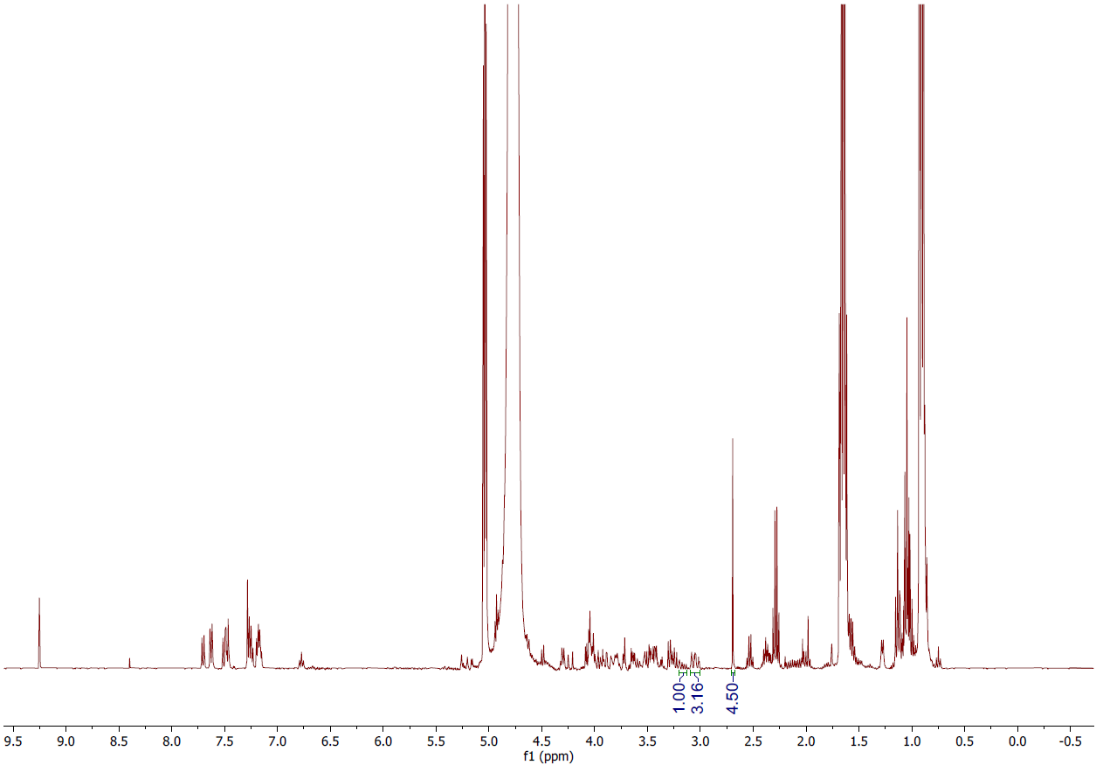

^{1}H NMR (400 MHz, D_2O) of **C** group at t = 72 h.

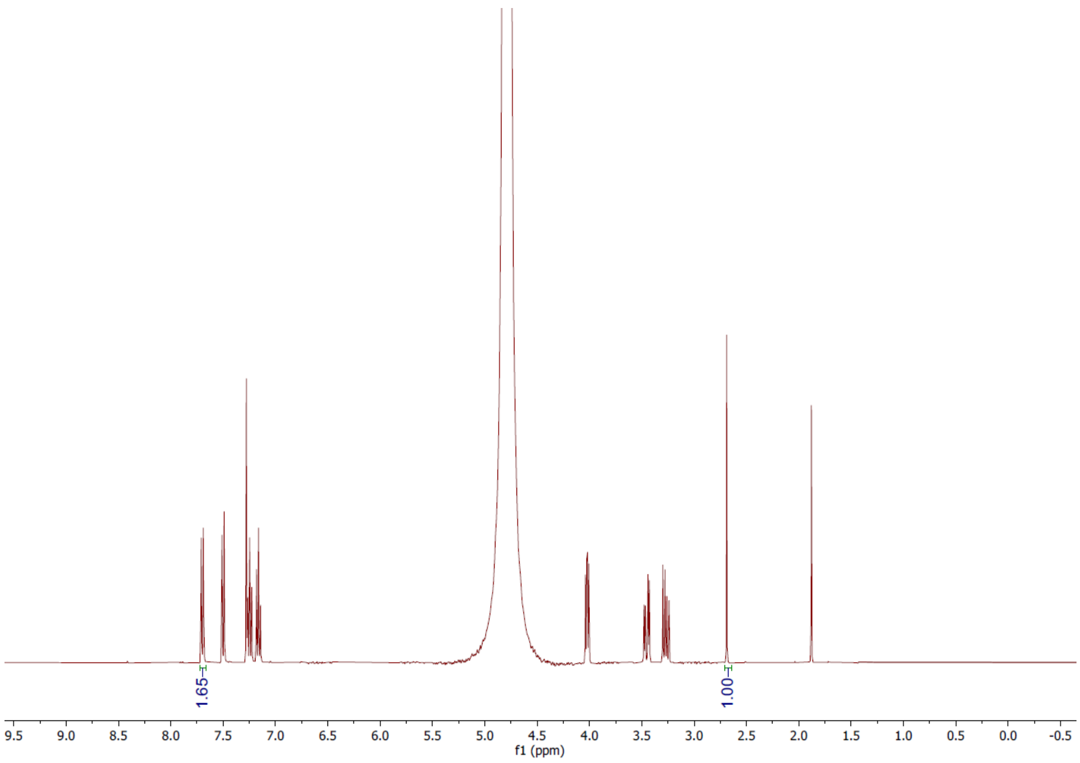

^{1}H NMR (400 MHz, D_2O) of **D** group at t = 0 h.

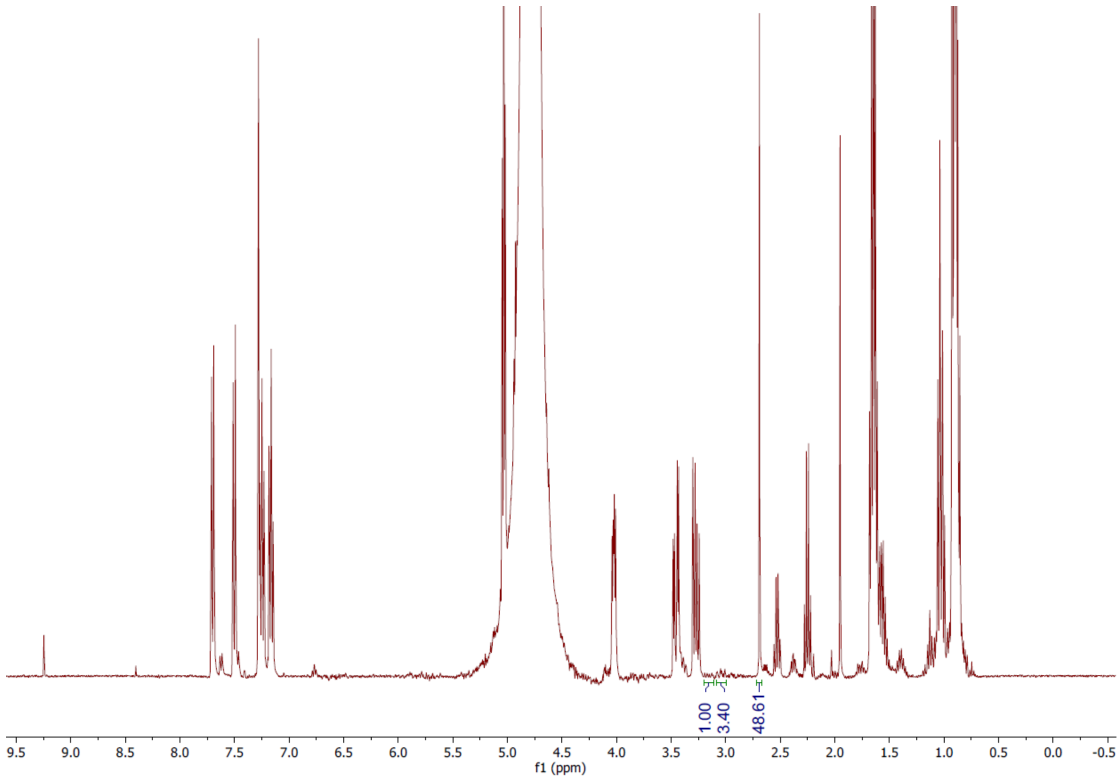

^{1}H NMR (400 MHz, D_2O) of **D** group at t = 15 h.

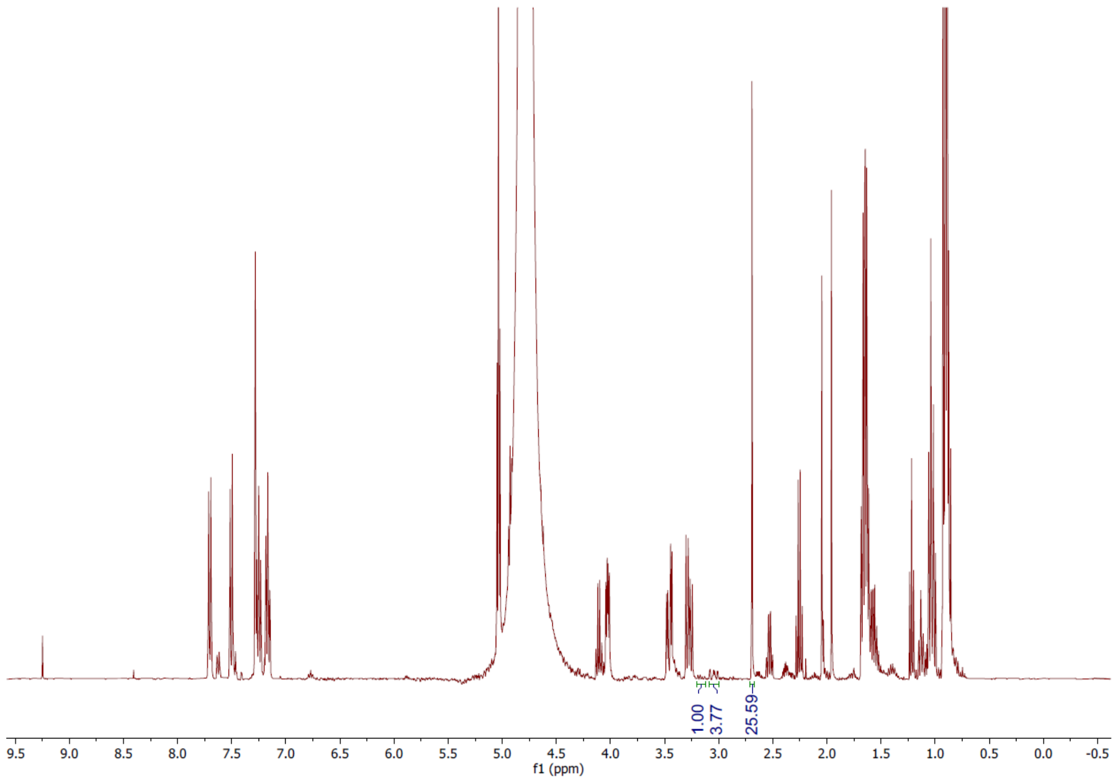

^{1}H NMR (400 MHz, D_2O) of **D** group at t = 30 h.

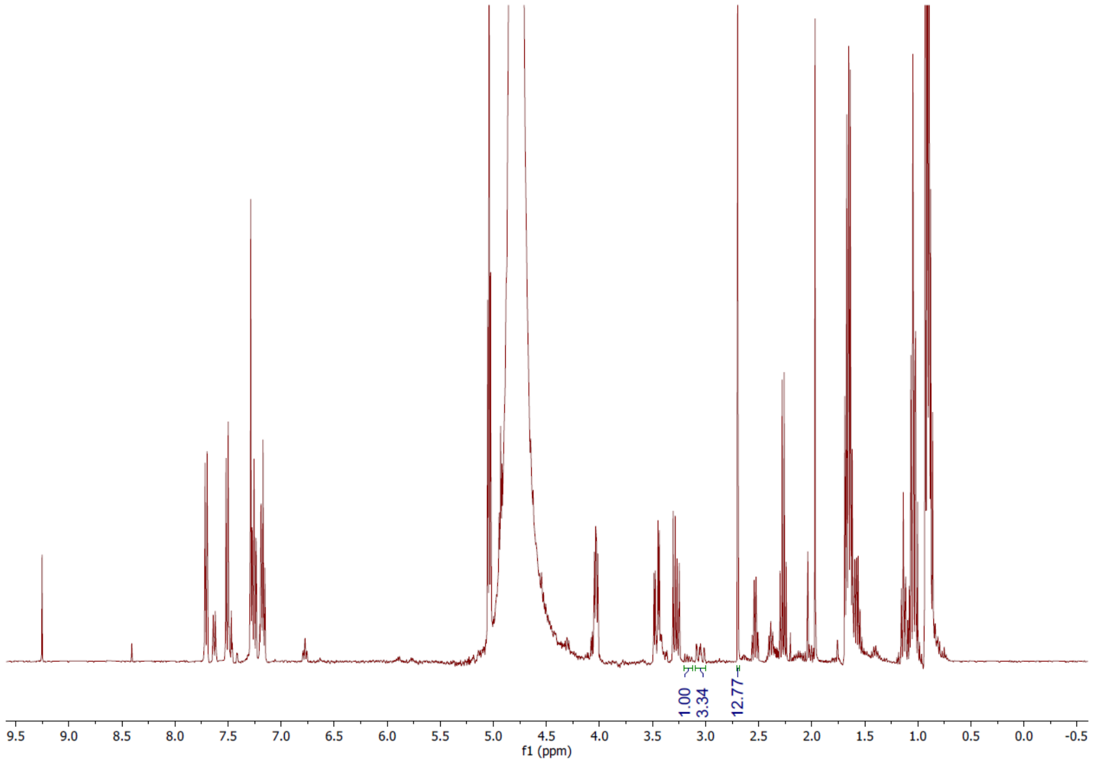

^{1}H NMR (400 MHz, D_2O) of **D** group at t = 45 h.

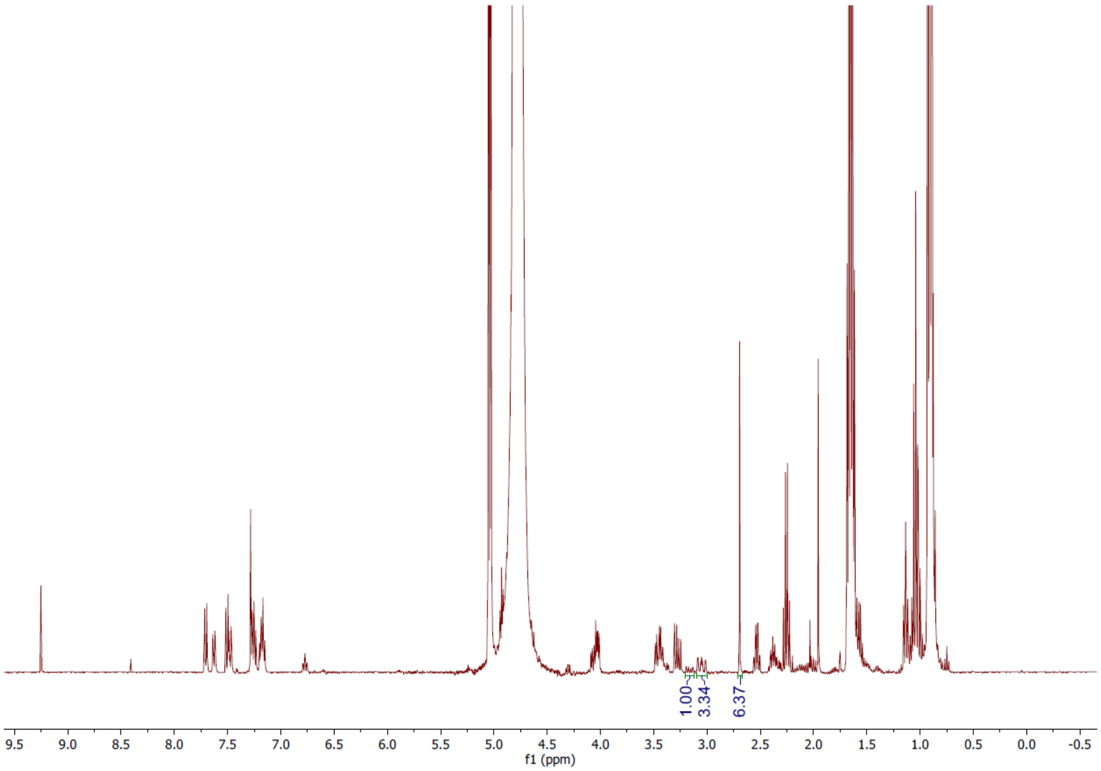

^{1}H NMR (400 MHz, D_2O) of **D** group at t = 72 h.

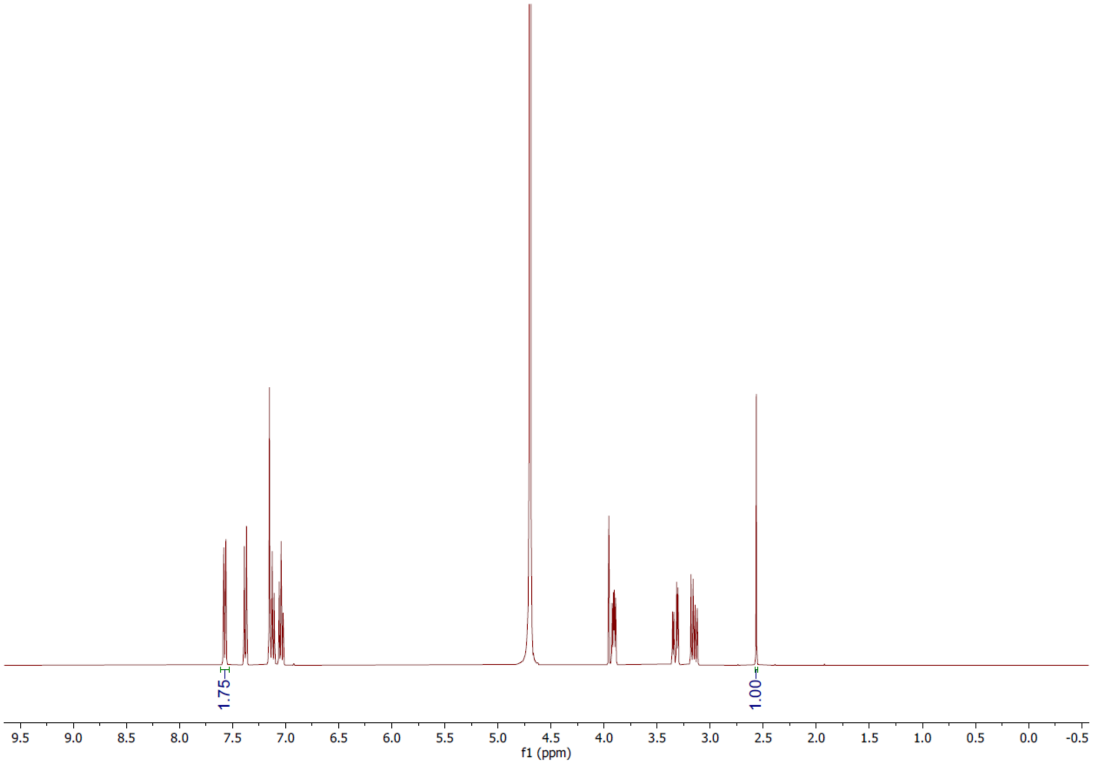

^{1}H NMR (400 MHz, D_2O) of **E** group at t = 0 h.

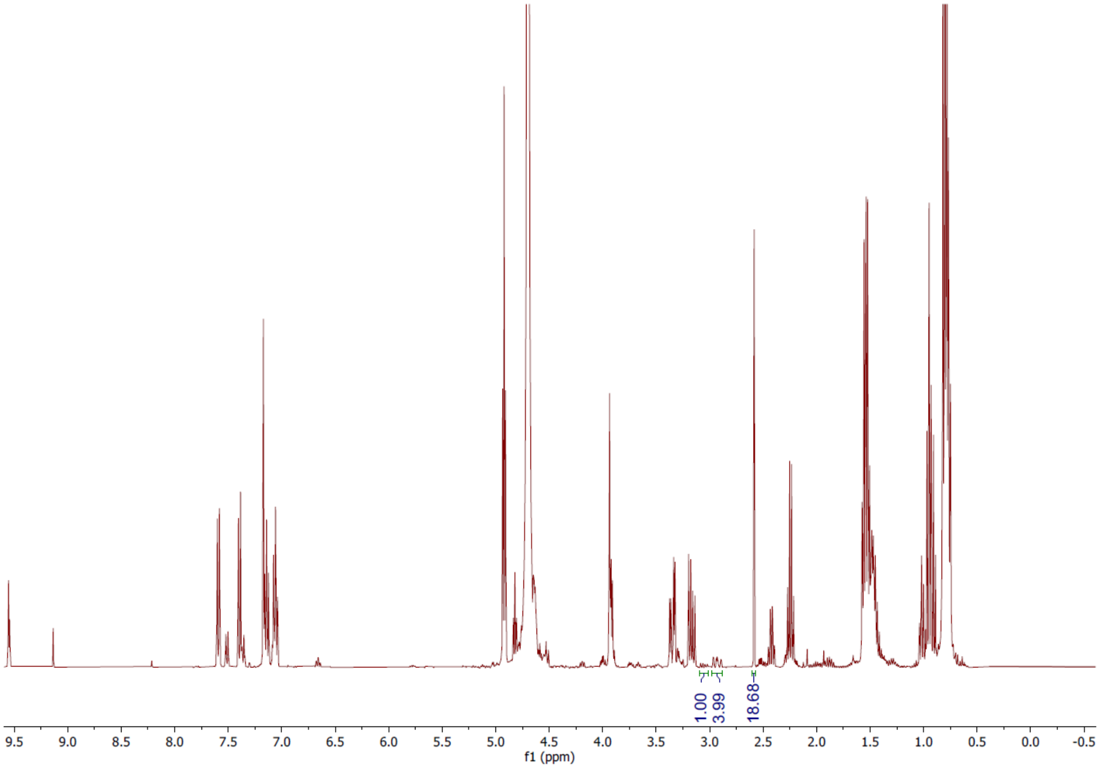

^{1}H NMR (400 MHz, D_2O) of **E** group at t = 15 h.

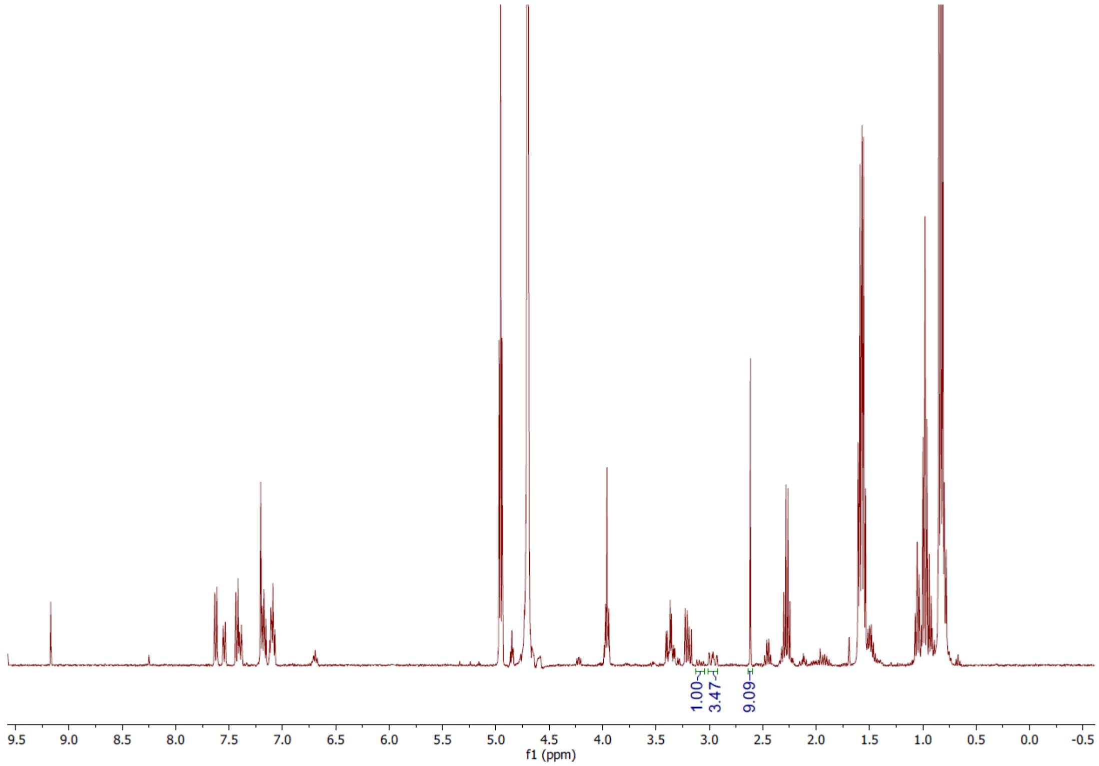

^{1}H NMR (400 MHz, D_2O) of **E** group at t = 30 h.

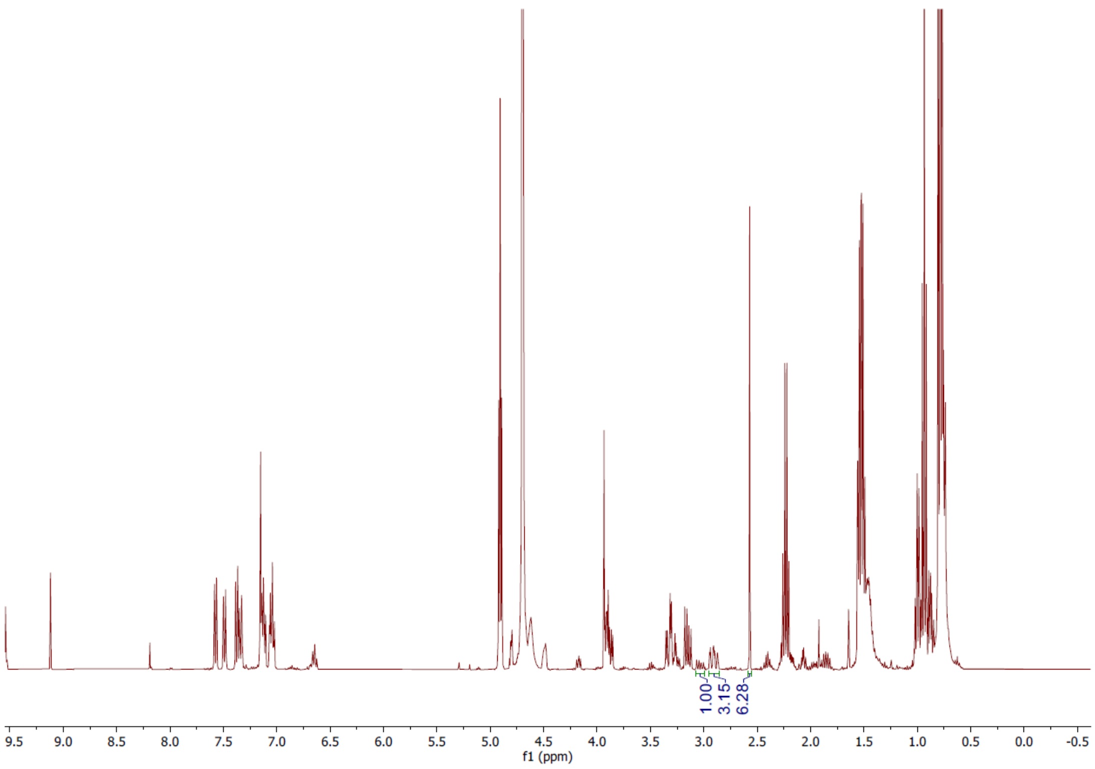

^{1}H NMR (400 MHz, D_2O) of **E** group at t = 45 h.

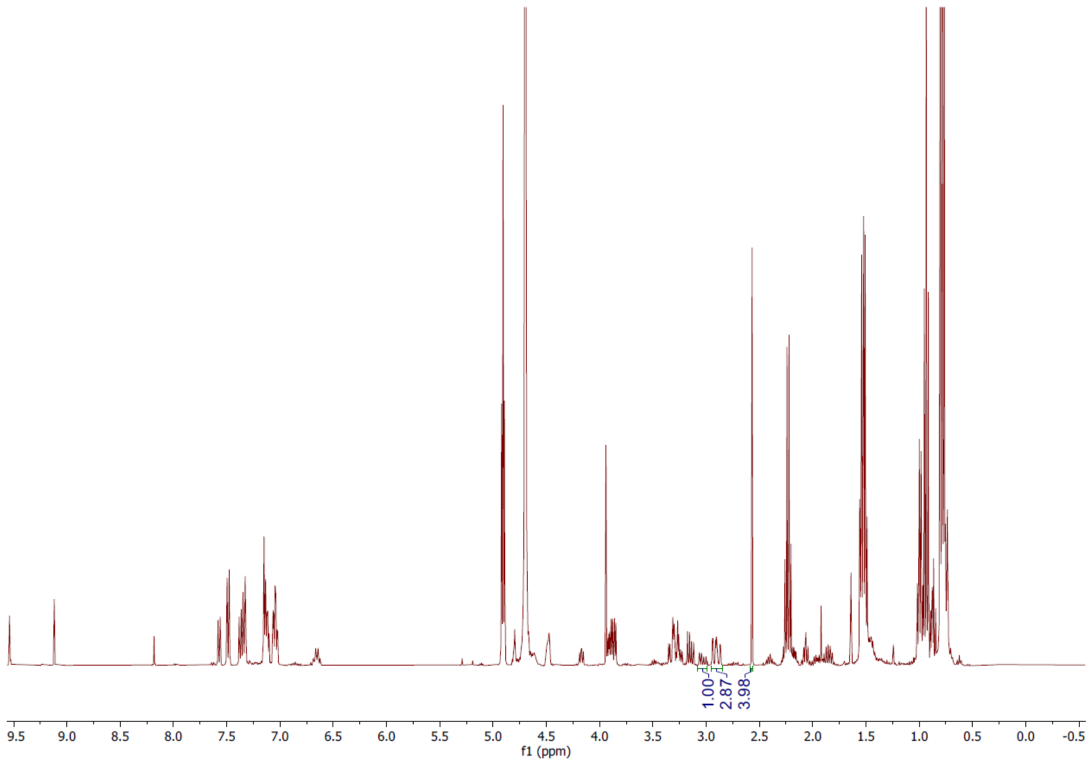

^{1}H NMR (400 MHz, D_2O) of **E** group at t = 72 h.

5.5 Pictet-Spengler modification of peptides

5.5.1 Functionalization of H-Trp-Gly-OH in the presence of 4mer-IV

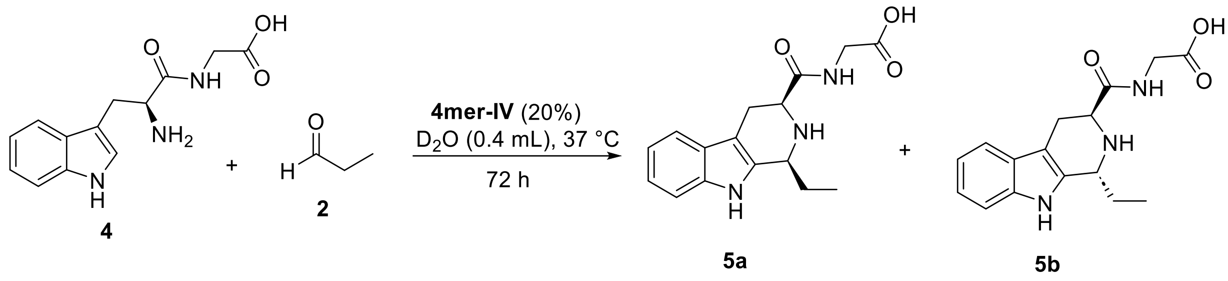

H-Trp-Gly-OH (6.5 mg, 0.025 mmol) and **4mer-IV** (3.6 mg, 0.005 mmol) were dissolved into D_2O (0.4 mL, containing DMSO (6.5 mM, 0.18 µL) as internal standard). Propionaldehyde (44 µL, 0.3 mmol) was added to the reaction while stirring at 37 °C. After 72 h, the reaction was analyzed by ^{1}H NMR to determine a 63% yield and cis : trans = 73 : 27. The target compounds were isolated by HPLC (**Method C3**).

For **5a** ^{1}H NMR (400 MHz, D_2O) δ 7.61 (dt, *J* = 7.9, 1.1 Hz, 1H), 7.49 (dt, *J* = 8.2, 0.9 Hz, 1H), 7.28 (m, 1H), 7.19 (m, 1H), 4.72 (dt, *J* = 8.7, 2.6 Hz, 1H), 4.35 (dd, *J* = 12.0, 5.0 Hz, 1H), 3.99 (d, *J* = 17.5 Hz, 1H), 3.88 (d, *J* = 17.4 Hz, 1H), 3.44 (m, 1H), 3.19 (m, 1H), 2.36 (m, 1H), 2.00 (m, 1H), 1.14 (t, *J* = 7.4 Hz, 3H). ^{13}C NMR (101 MHz, D_2O) δ 175.36, 169.21, 167.74, 136.62, 129.29, 125.29, 122.80, 119.95, 118.28, 111.68, 105.15, 56.76, 55.41, 42.64, 24.28, 22.88, 8.57. (ESI-HRMS) m/z 302.1517 $[M+H]^+$ ($C_{16}H_{20}N_3O_3$ requires 302.1505).

For **5b**: ^{1}H NMR (700 MHz, D_2O) δ 7.60 (d, *J* = 7.9 Hz, 1H), 7.47 (d, *J* = 8.2 Hz, 1H), 7.26 (m, 1H), 7.21 – 7.14 (m, 1H), 4.58 (dd, *J* = 10.3, 5.5 Hz, 1H), 3.92 (d, *J* = 17.2 Hz, 1H), 3.73 (d, *J* = 17.2 Hz, 1H), 3.45 (dd, *J* = 16.3, 5.4 Hz, 1H), 3.21 – 3.10 (m, 1H), 2.09 (m, 2H), 1.16 – 1.08 (m, 3H). ^{13}C NMR (176 MHz, D_2O) δ 175.52, 169.46, 168.29, 136.37, 129.13, 125.11, 122.82, 119.83, 118.21, 111.60, 104.14, 53.42, 52.34, 42.75, 22.68, 9.20. (ESI-HRMS) m/z 302.1513 $[M+H]^+$ ($C_{16}H_{20}N_3O_3$ requires 302.1505).

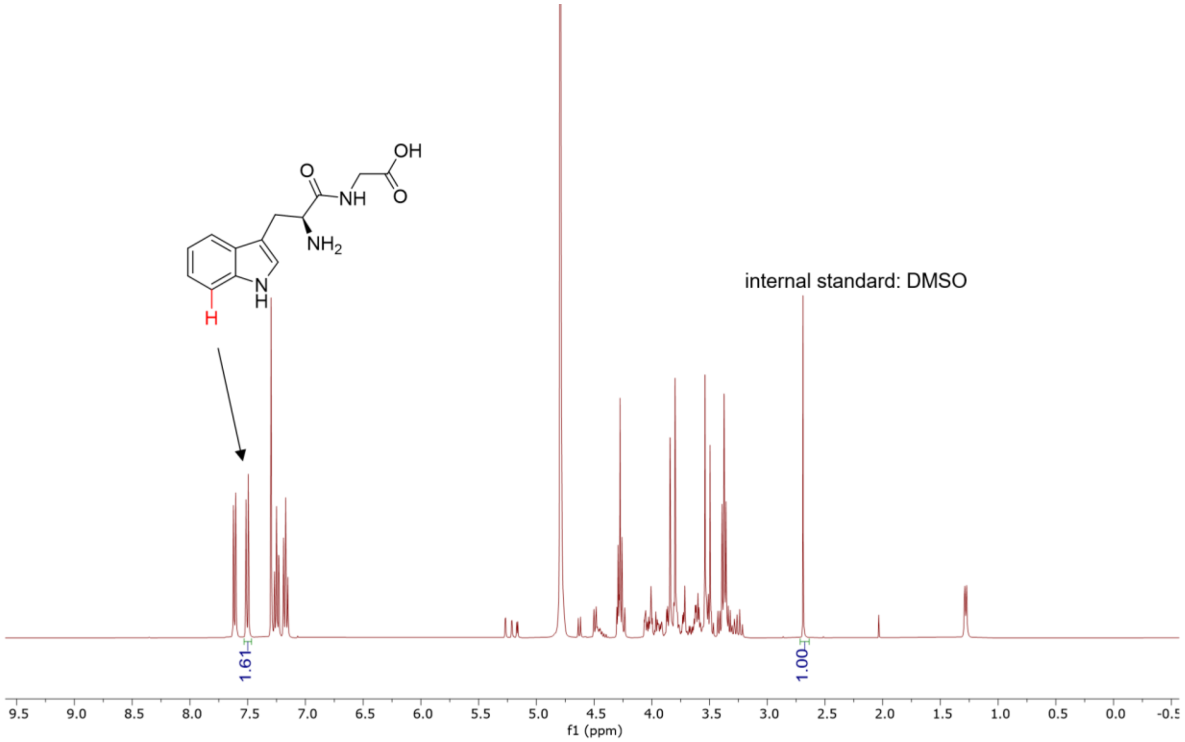

^{1}H NMR (400 MHz, D_2O) of the reaction system at t = 0 h. The proton used for analysis is highlighted in red.

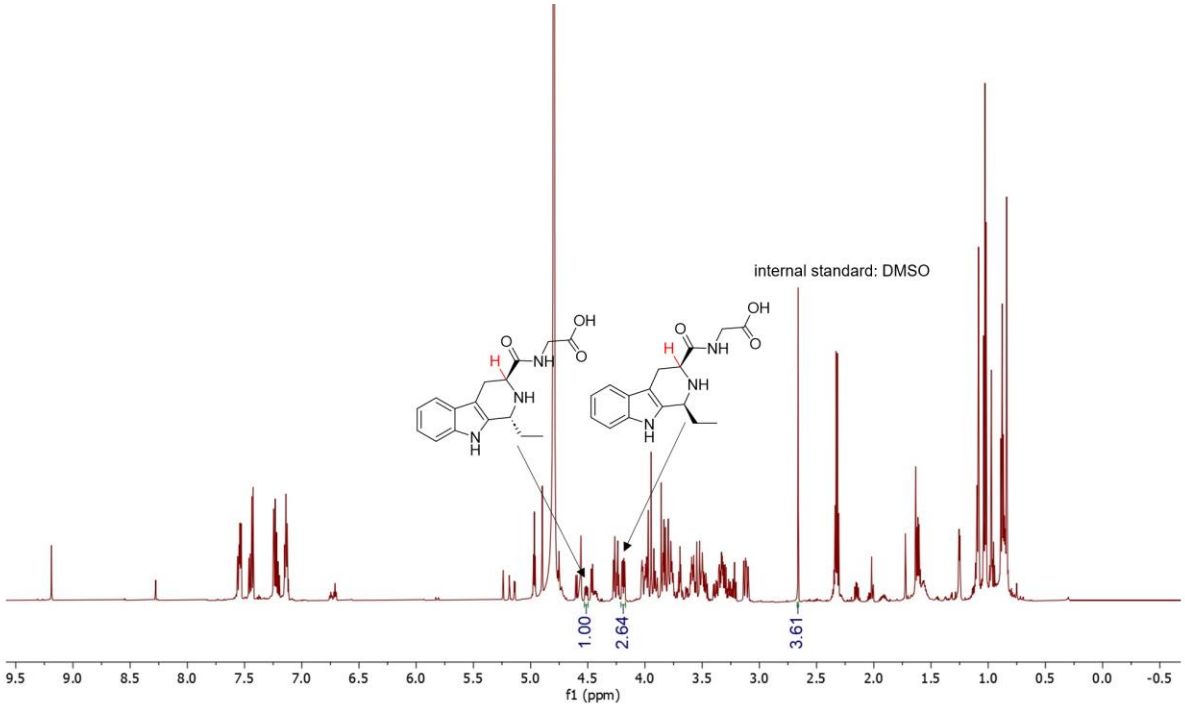

^{1}H NMR (400 MHz, D_2O) of the reaction system at t = 72 h. The protons used for analysis are highlighted in red.

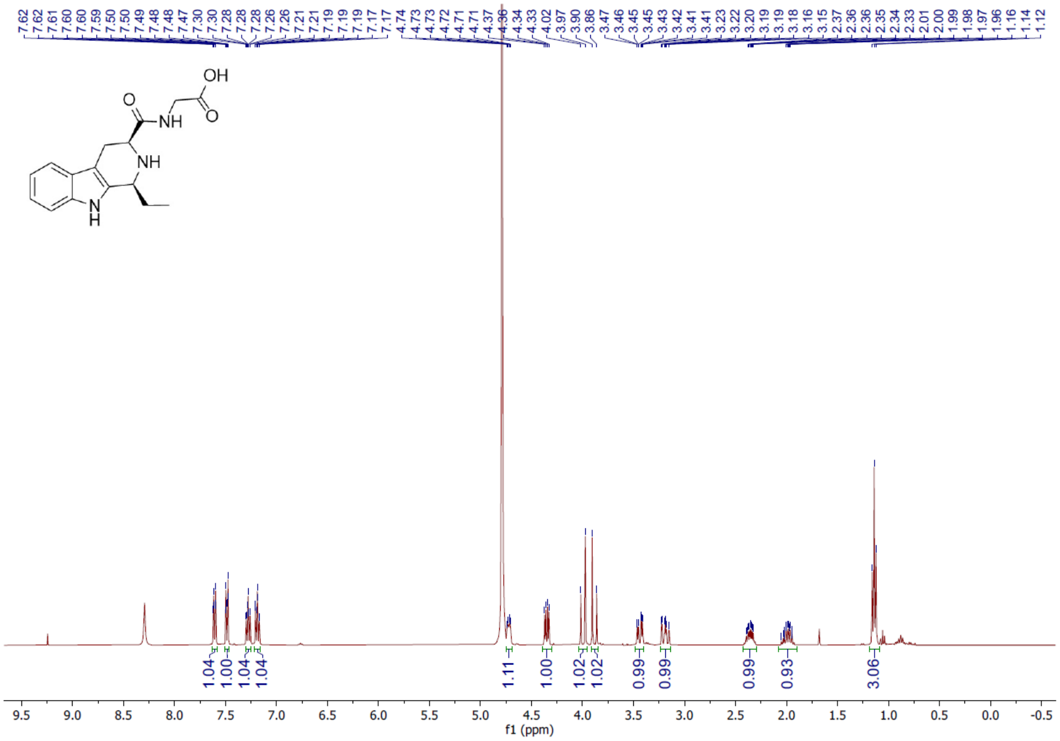

^{1}H NMR of **5a** (400 MHz, D_2O)

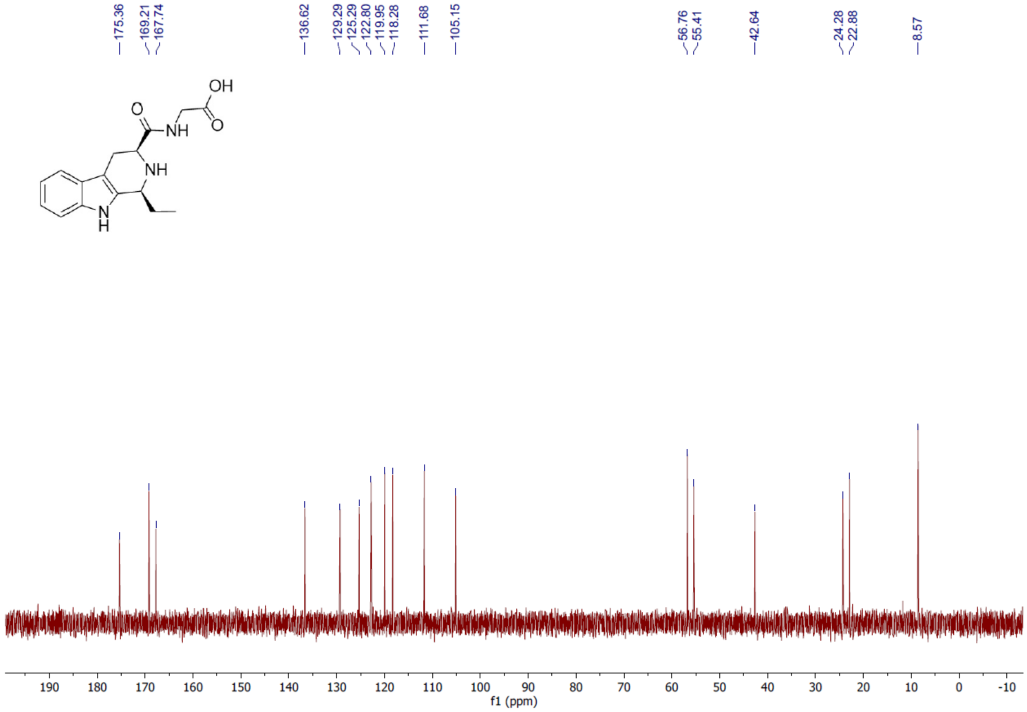

^{13}C NMR of 5a (101 MHz, D_2O)

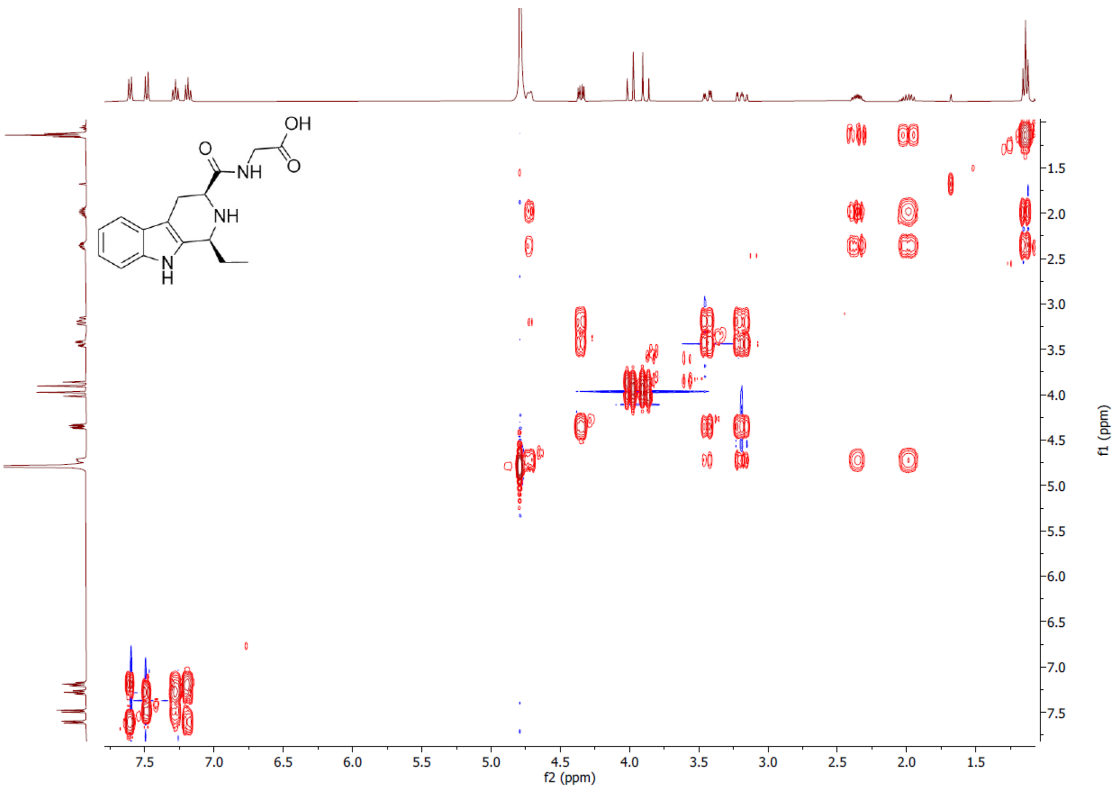

COSY NMR of 5a

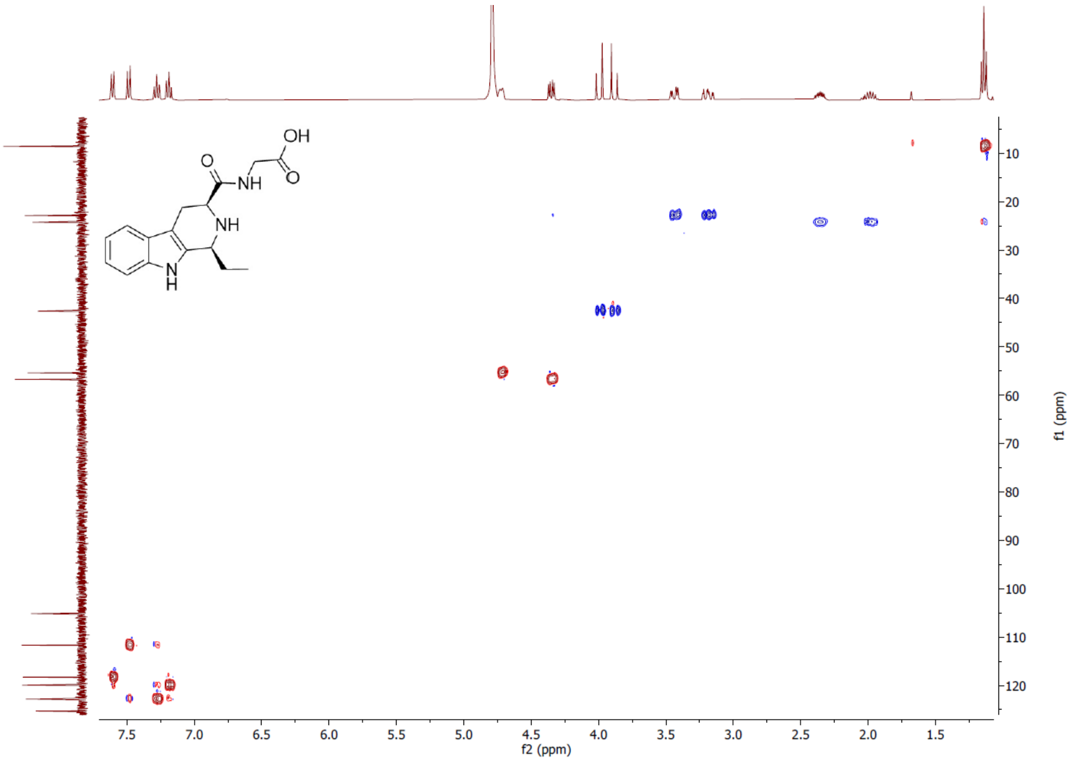

HSQC NMR of 5a

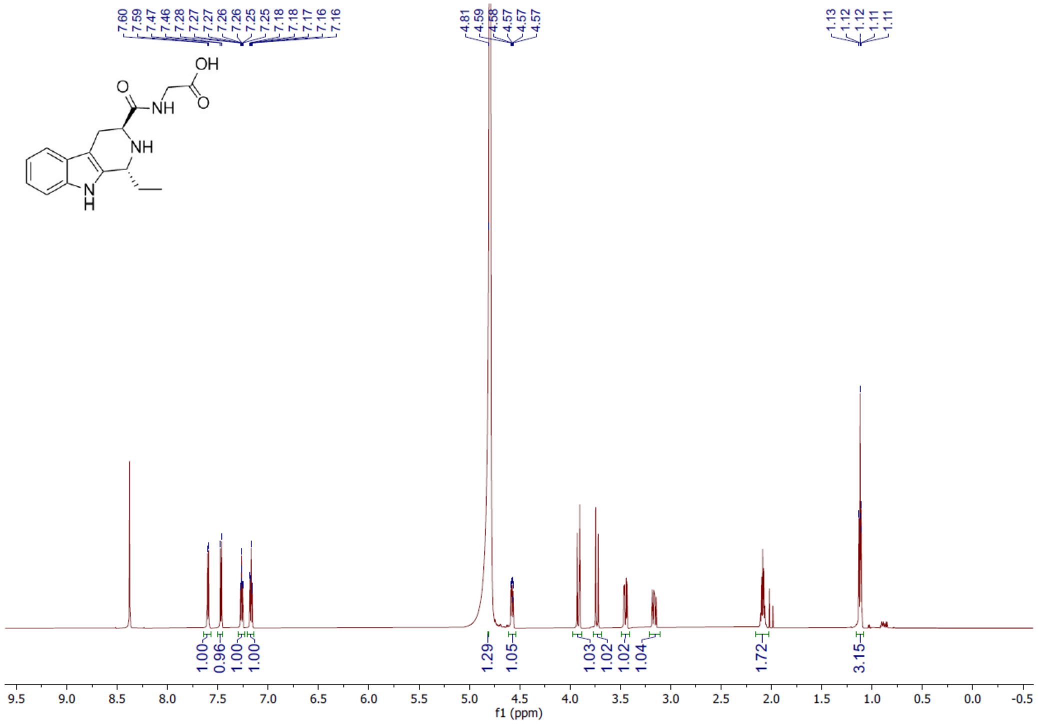

^{1}H NMR of **5b** (700 MHz, D_2O)

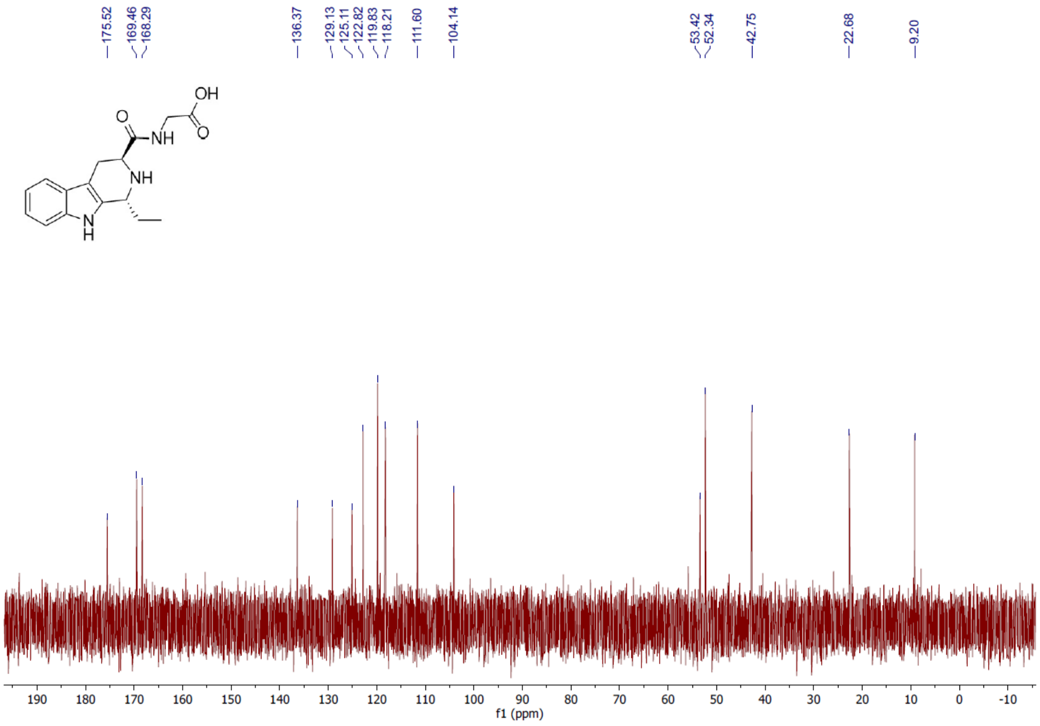

^{13}C NMR of 5b (176 MHz, D_2O)

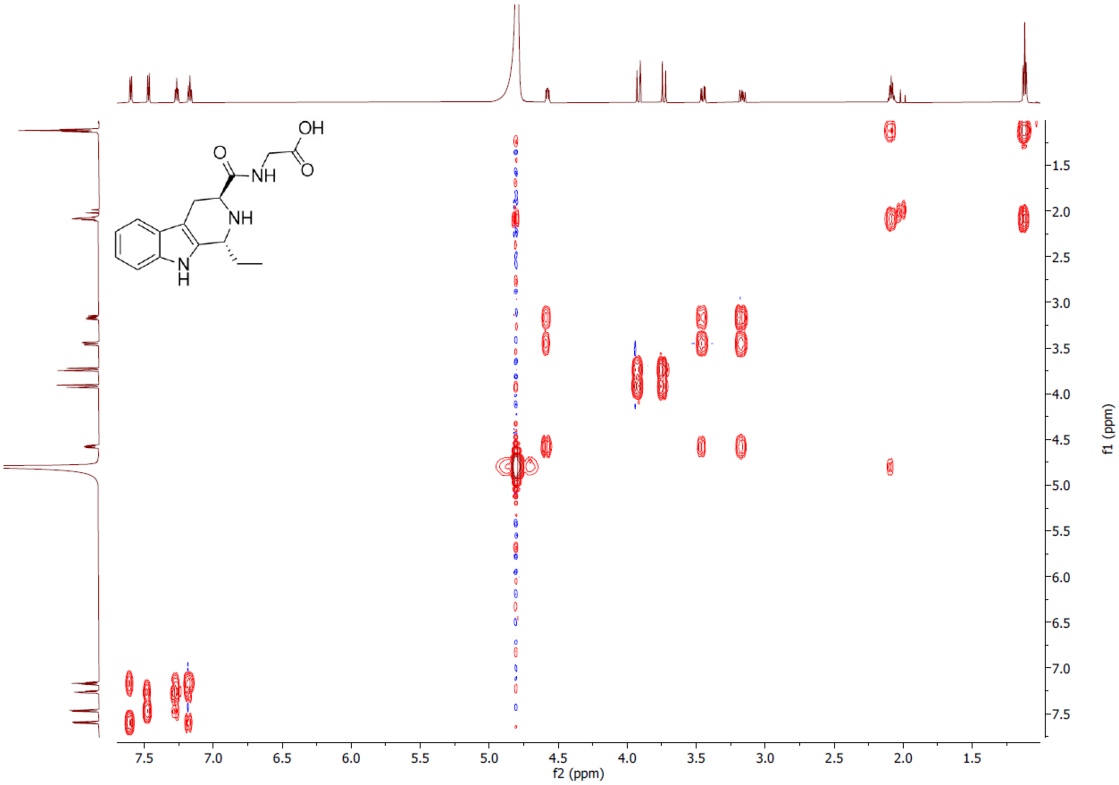

COSY NMR of 5b

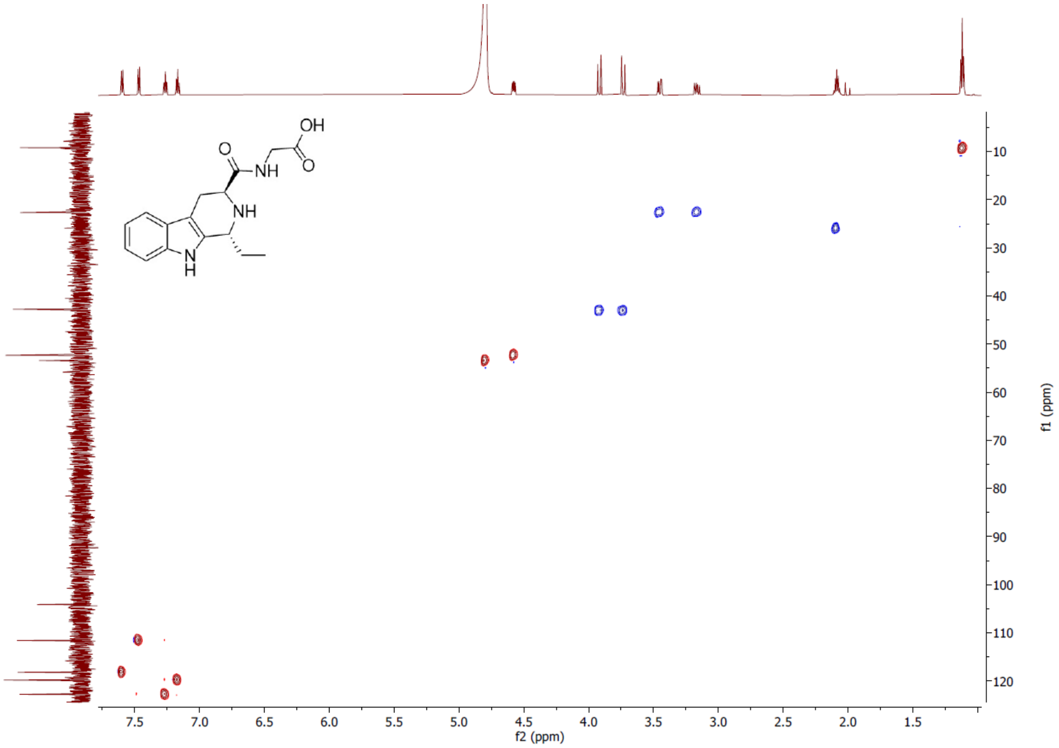

HSQC NMR of 5b

5.5.2 Functionalization of H-Trp-Gly-OH in the presence of AcOH

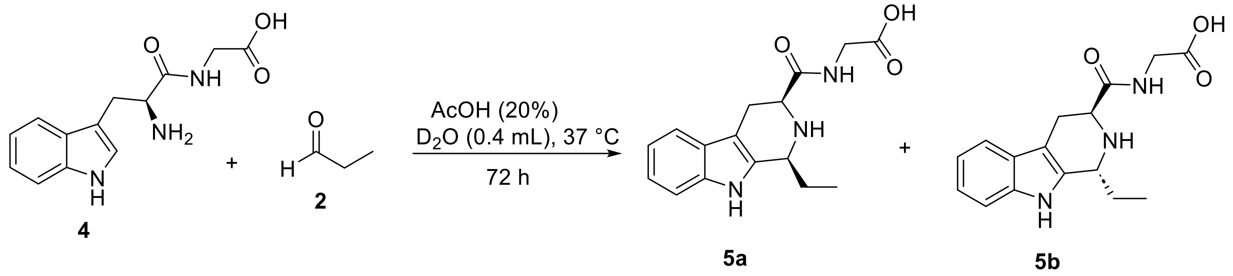

H-Trp-Gly-OH (6.5 mg, 0.025 mmol) was added to a AcOH (12.5 mM) solution in D_2O (0.4 mL, containing DMSO (6.5 mM, 0.18 µL) as internal standard). Propionaldehyde (44 µL, 0.3 mmol) was added to the reaction while stirring at 37 °C. After 72 h, the reaction was analyzed by ^1H NMR to determine a 46% yield and cis : trans = 72 : 28.

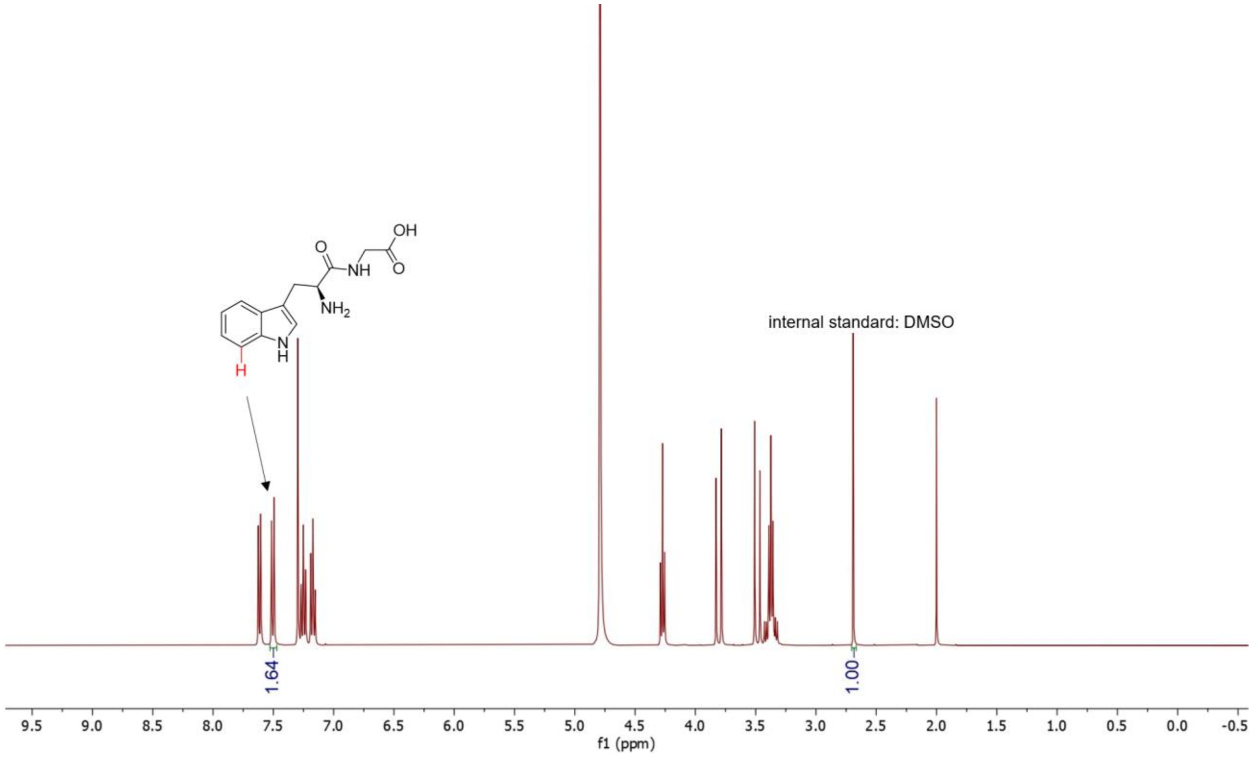

^1H NMR (400 MHz, D_2O) of the reaction system at t = 0 h. The proton used for analysis is highlighted in red.

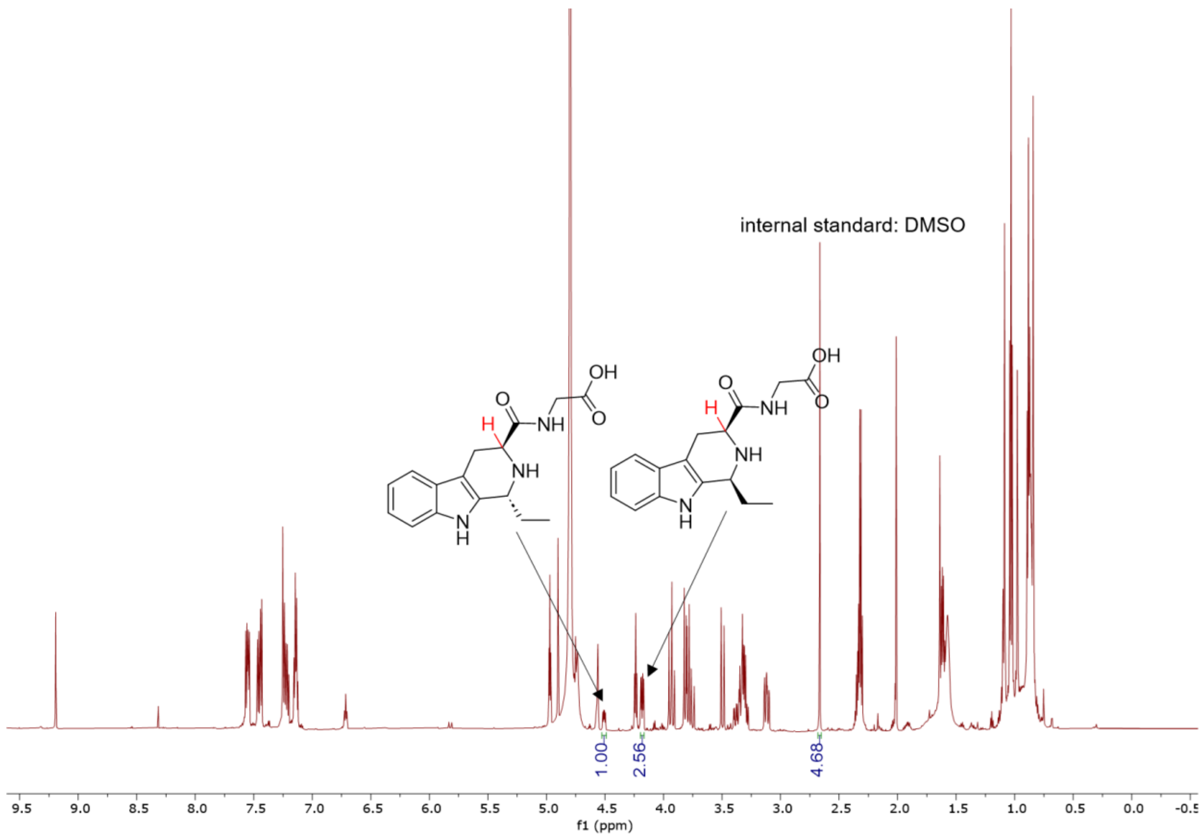

^{1}H NMR (400 MHz, D_2O) of the reaction system at t = 72 h. The protons used for analysis are highlighted in red.

5.5.3 Functionalization of H-Trp-Ala-Gly-Gly-Asp-Ala-Ser-Gly-Glu-OH in the presence of 4mer-IV

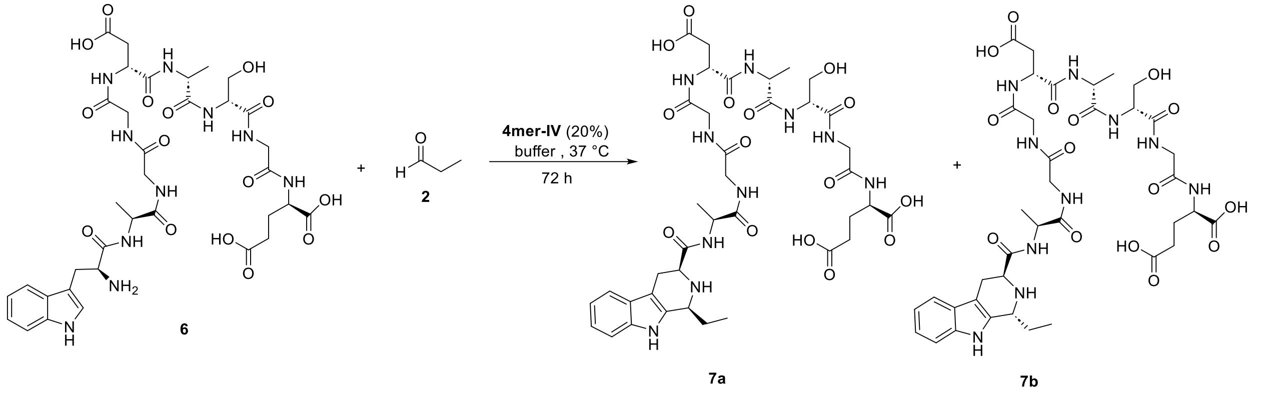

Peptide **6** (2.7 mg, 3.1 µmol) and **4mer-IV** (0.45 mg, 0.63 µmol) were dissolved into DPBS buffer (50 µL). Propionaldehyde (5.5 µL, 0.038 mmol) was added to the reaction while stirring at 37 °C. After 72 h, the reaction was analyzed by ^{1}H NMR to determine a 84% conversion. The target compounds were isolated by HPLC (1.9 mg, 69% yield, cis : trans = 75 : 25) (**Method C3**).

7a: ^{1}H NMR (400 MHz, D_2O) δ 7.58 (d, *J* = 7.9 Hz, 1H), 7.51 – 7.46 (m, 1H), 7.31 – 7.24 (m, 1H), 7.21 – 7.14 (m, 1H), 4.78 (m, 1H), 4.58 (dd, *J* = 7.5, 5.8 Hz, 1H), 4.49 (q, *J* = 7.1 Hz, 1H), 4.39 (dd, *J* = 12.0, 4.9 Hz, 1H), 4.30 (t, *J* = 4.9 Hz, 1H), 4.18 (q, *J* = 7.1 Hz, 1H), 4.10 – 4.05 (m, 1H), 4.04 – 3.75 (m, 8H), 3.45 (dd, *J* = 16.1, 5.0 Hz, 1H), 3.22 – 3.10 (m, 1H), 2.68 (m, 2H), 2.36 (m, 1H), 2.17 (t, *J* = 8.0 Hz, 2H), 2.06 – 1.96 (m, 2H), 1.88 – 1.77 (m, 1H), 1.46 (d, *J* = 7.1 Hz, 3H), 1.27 (d, *J* = 7.2 Hz, 3H), 1.15 (t, *J* = 7.5 Hz, 3H).^{13}C NMR (176 MHz, D_2O) δ 177.56, 175.46, 174.91, 173.35, 172.17, 172.02, 171.19, 171.01, 170.29, 119.77, 118.14, 111.61, 61.00, 56.63, 55.89, 55.11, 51.38, 49.81, 49.65, 42.62, 42.41, 42.38, 38.52, 33.96, 28.37, 16.41, 16.12, 8.64. (ESI-HRMS) m/z 889.3690 $[M+H]^+$ ($C_{38}H_{53}N_{10}O_{15}$ requires 889.3692).

7b: ^{1}H NMR (700 MHz, D_2O) δ 7.59 (d, *J* = 7.9 Hz, 1H), 7.47 (d, *J* = 8.2 Hz, 1H), 7.27 (t, *J* = 7.6 Hz, 1H), 7.17 (t, *J* = 7.5 Hz, 1H), 4.84 (m, 1H), 4.60 (dd, *J* = 9.3, 5.4 Hz, 1H), 4.55 (dd, *J* = 7.5, 5.7 Hz, 1H), 4.42 (q, *J* = 7.2 Hz, 1H), 4.33 (t, *J* = 4.9 Hz, 1H), 4.25 (q, *J* = 7.2 Hz, 1H), 4.10 (dd, *J* = 9.1, 4.5 Hz, 1H), 3.95 (d, *J* = 16.9 Hz, 1H), 3.90 – 3.77 (m, 7H), 3.46 (dd, *J* = 16.4, 5.4 Hz, 1H), 3.22 (dd, *J* = 16.4, 9.2 Hz, 1H), 2.69 (dd, *J* = 16.2, 5.7 Hz, 1H), 2.63 (dd, *J* = 16.2, 7.6 Hz, 1H), 2.18 (t, *J* = 8.0 Hz, 2H), 2.09 (m, 2H), 2.02 (dd, *J* = 14.7, 5.4 Hz, 1H), 1.82 (dt, *J* = 16.1, 8.0 Hz, 1H), 1.43 (d, *J* = 7.2 Hz, 3H), 1.31 (d, *J* = 7.2 Hz, 3H), 1.12 (t, *J* = 7.5 Hz, 3H). ^{13}C NMR (176 MHz, D_2O) δ 175.13, 174.94, 173.37, 172.23, 172.20, 171.15, 170.92, 170.37, 136.37, 125.09, 111.68, 103.96, 60.97, 55.97, 54.83, 53.55, 52.33, 51.34, 49.90, 49.76, 42.42, 27.95, 26.06, 22.88, 16.17, 16.10, 9.39. (ESI-HRMS) m/z 889.3687 $[M+H]^+$ ($C_{38}H_{53}N_{10}O_{15}$ requires 889.3692).

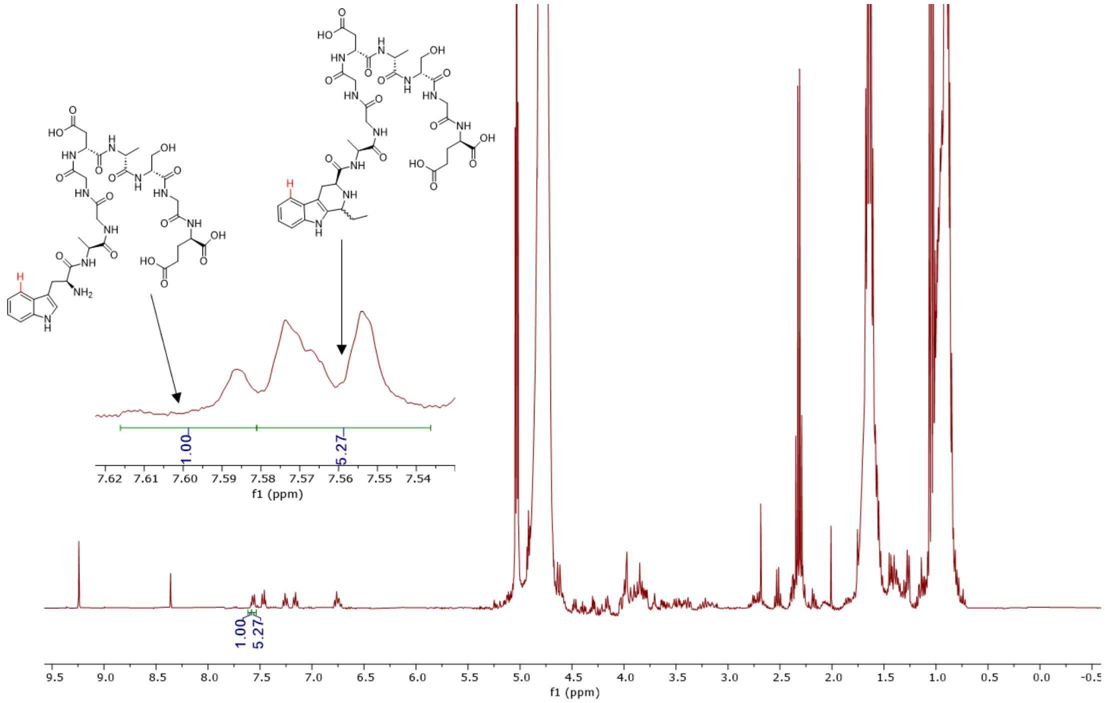

^{1}H NMR (400 MHz) of the reaction system at t = 72 h. The proton used for analysis is highlighted in red.

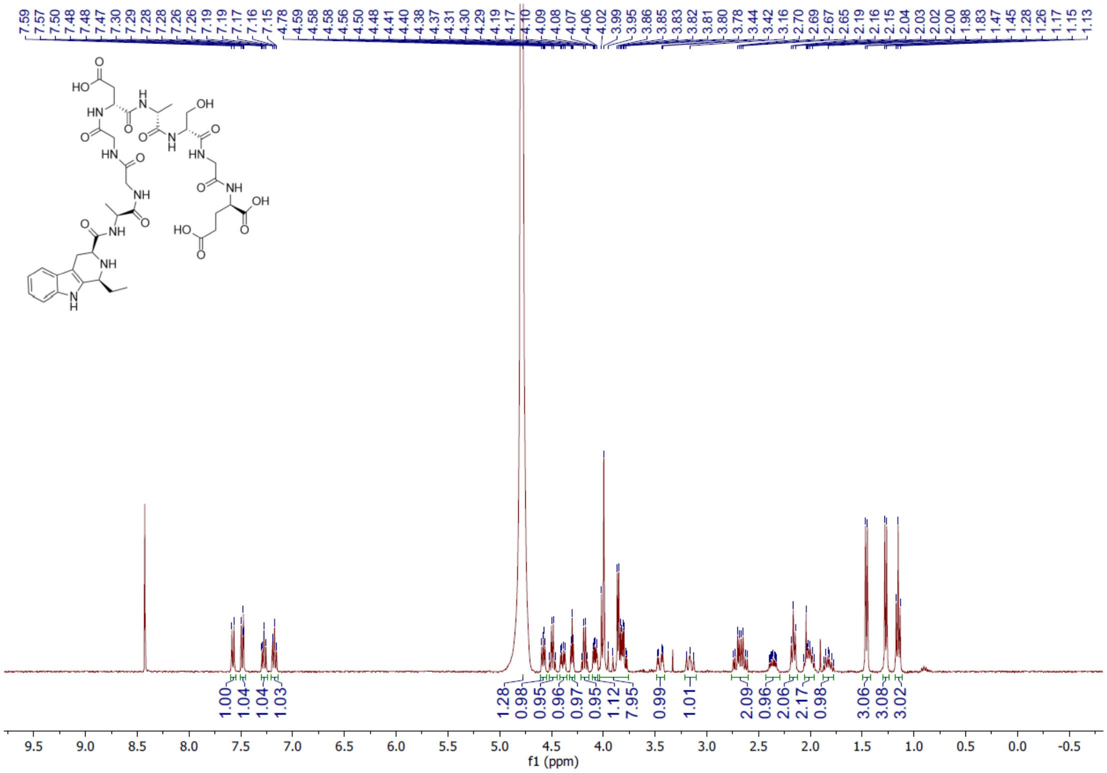

^{1}H NMR of **7a** (400 MHz, D_2O)

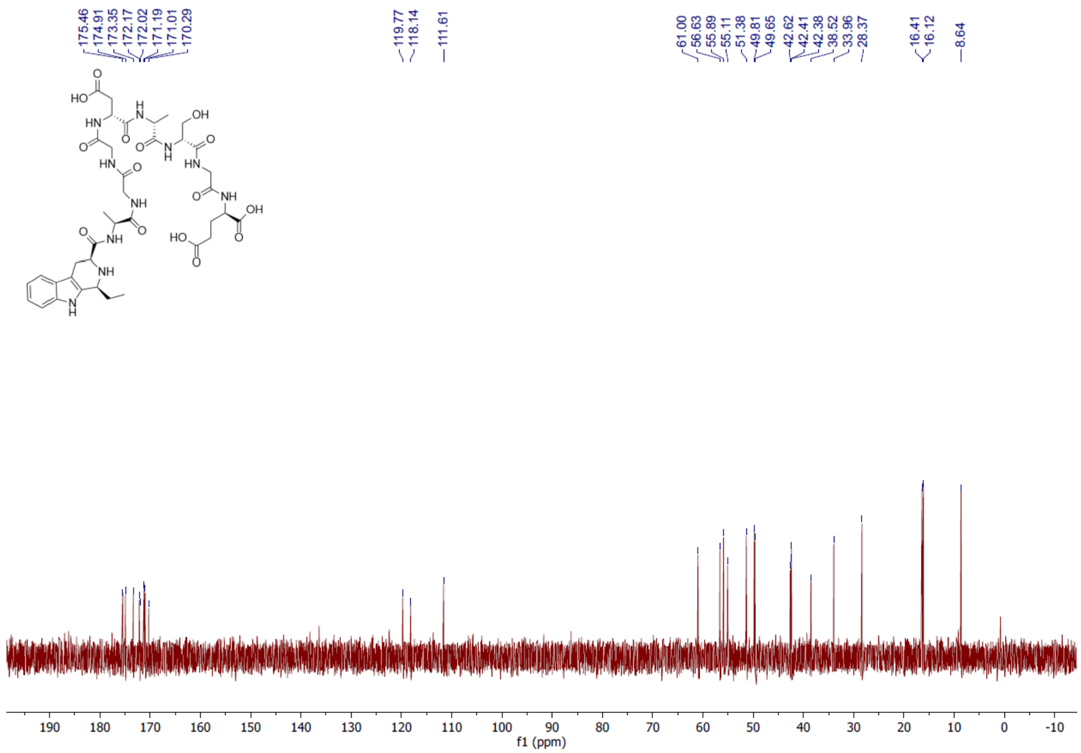

^{13}C NMR of 7a (176 MHz, D_2O)

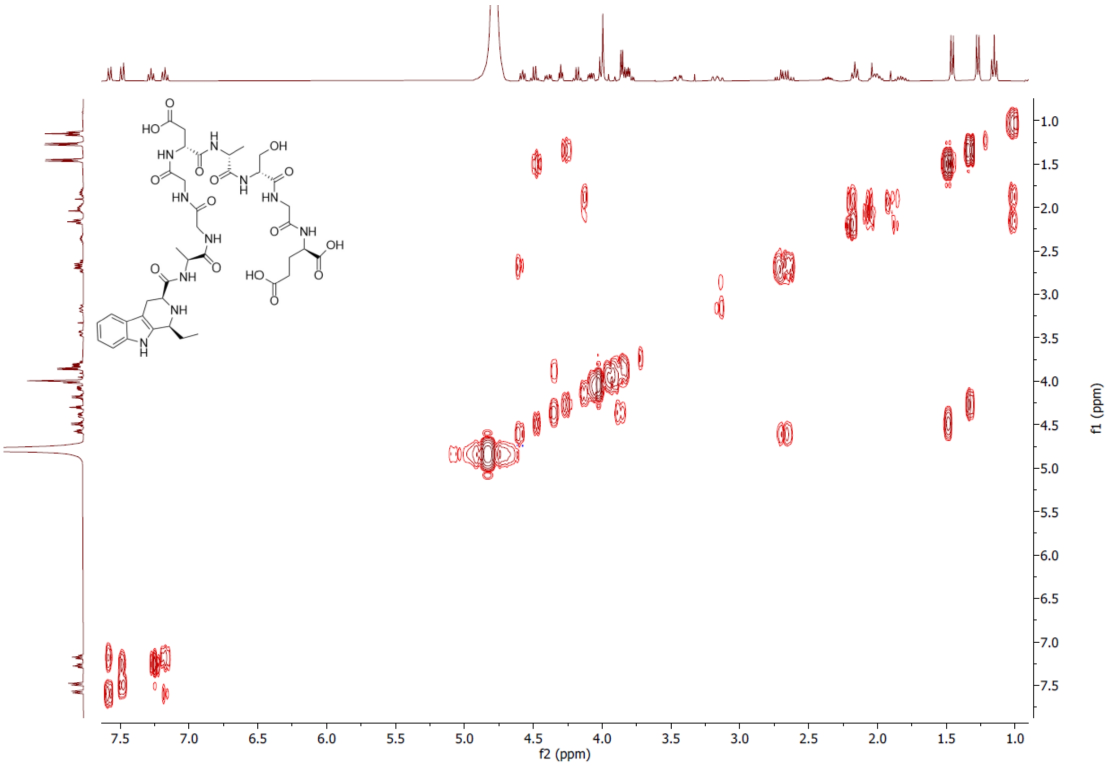

COSY NMR of 7a

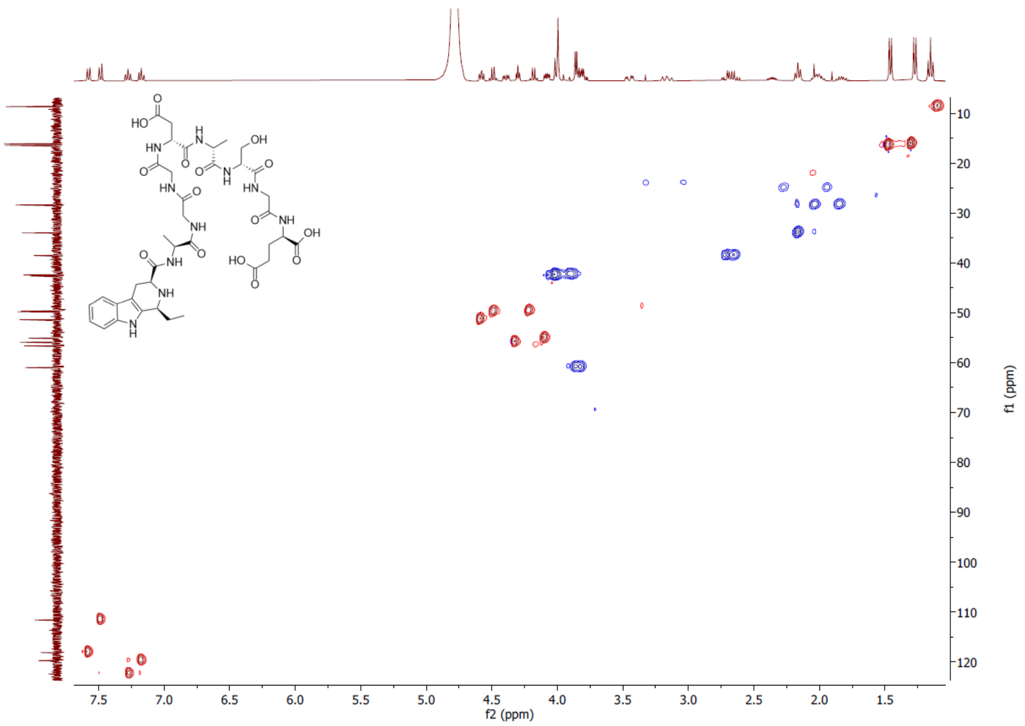

HSQC NMR of 7a

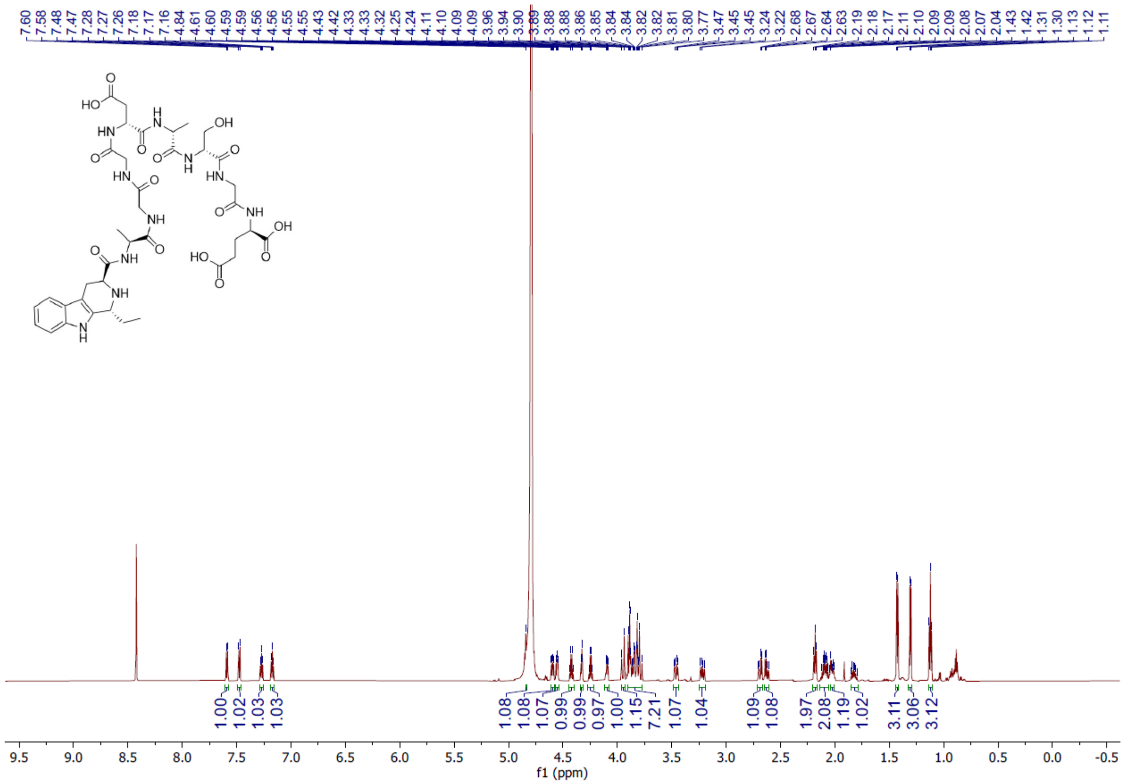

^{1}H NMR of **7b** (700 MHz, D_2O)

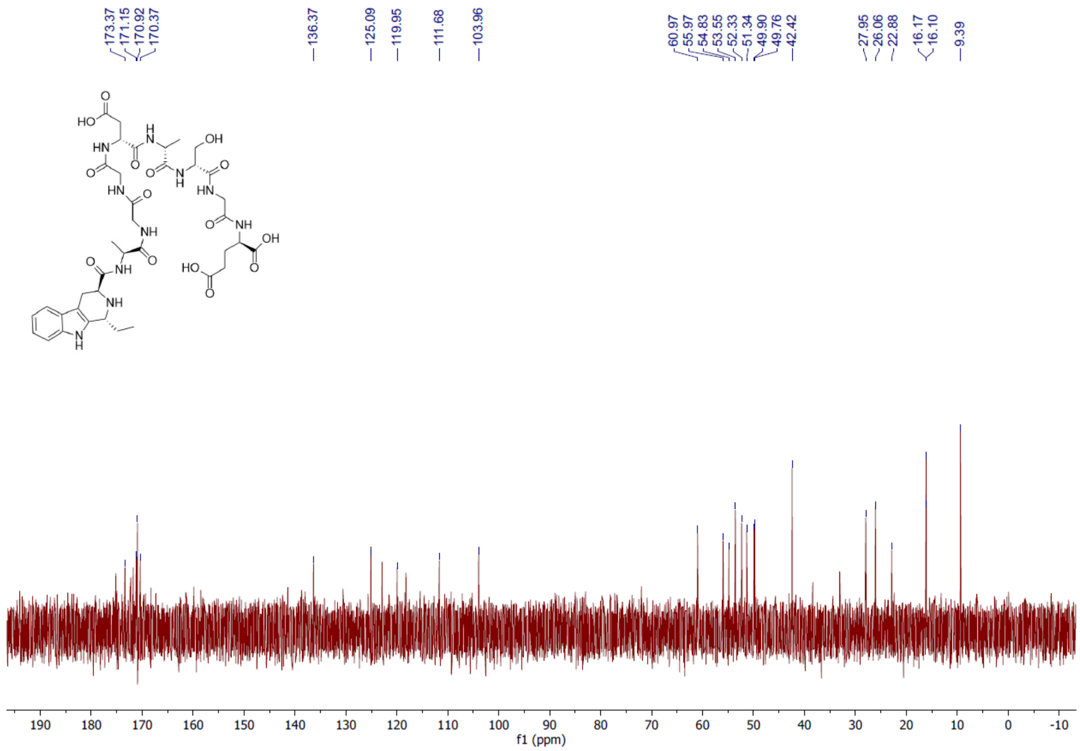

^{13}C NMR of 7b (176 MHz, D_2O)

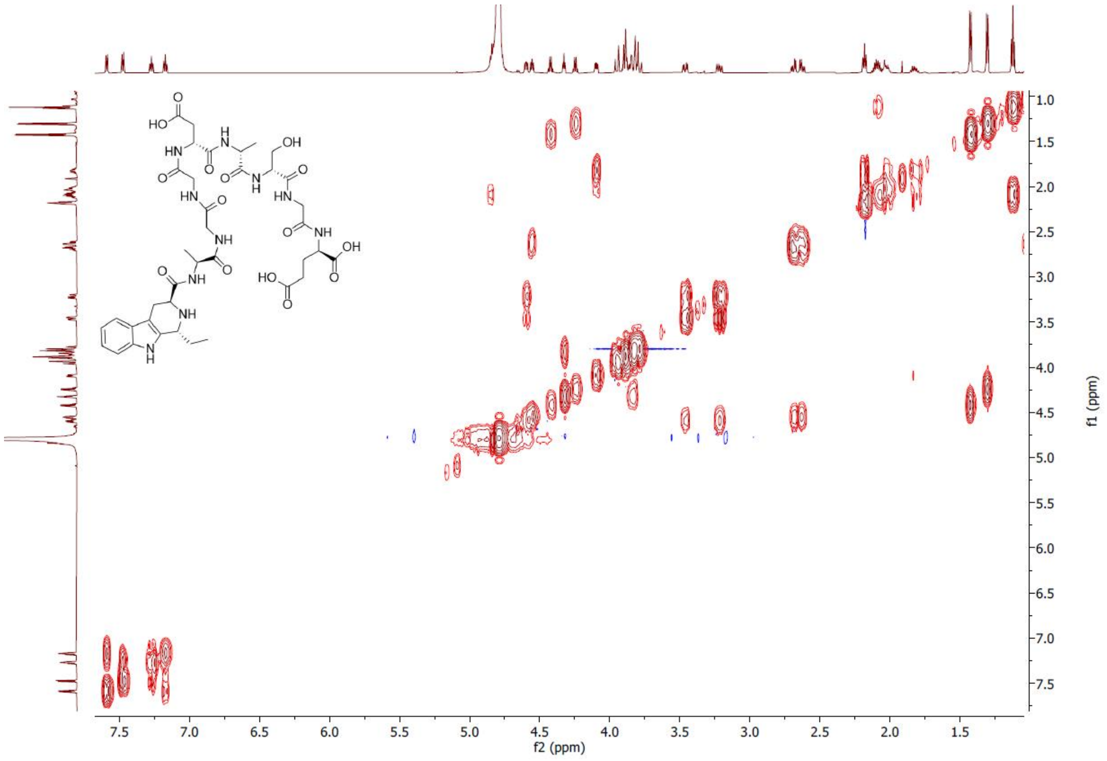

COSY NMR of 7b

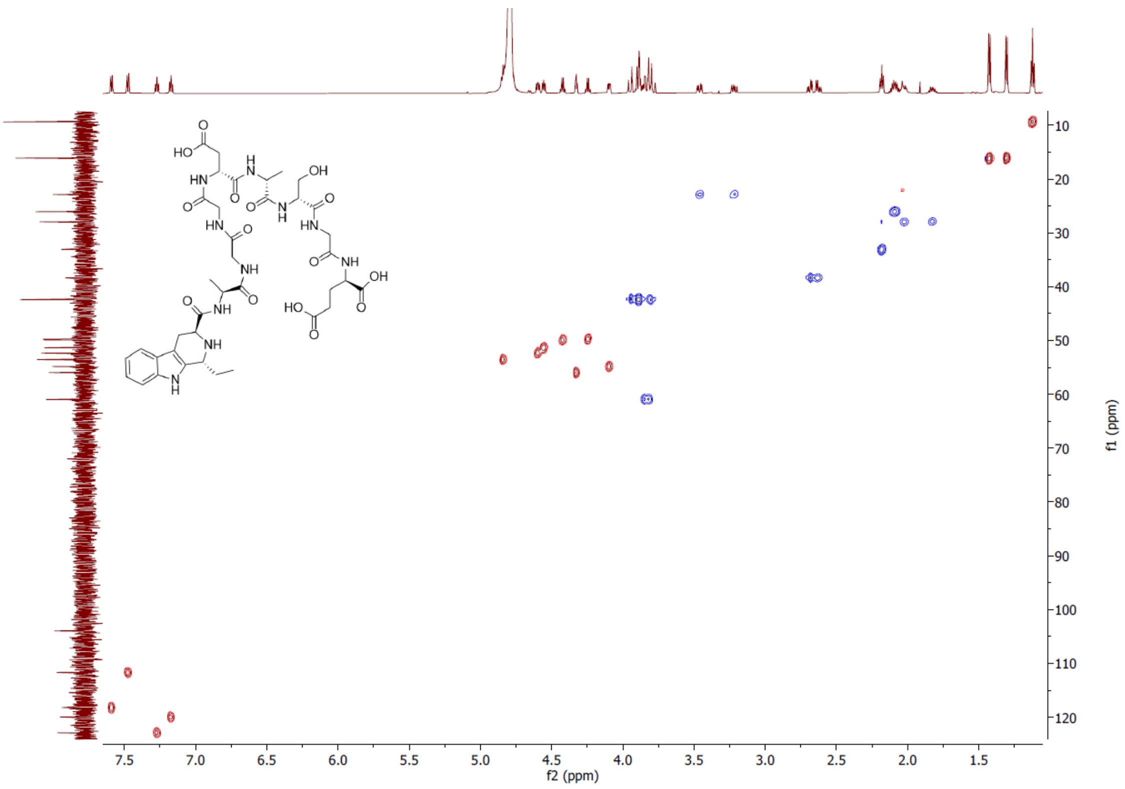

HSQC NMR of 7b

5.5.4 Functionalization of H-Trp-Ala-Gly-Gly-Asp-Ala-Ser-Gly-Glu-OH in the presence of AcOH

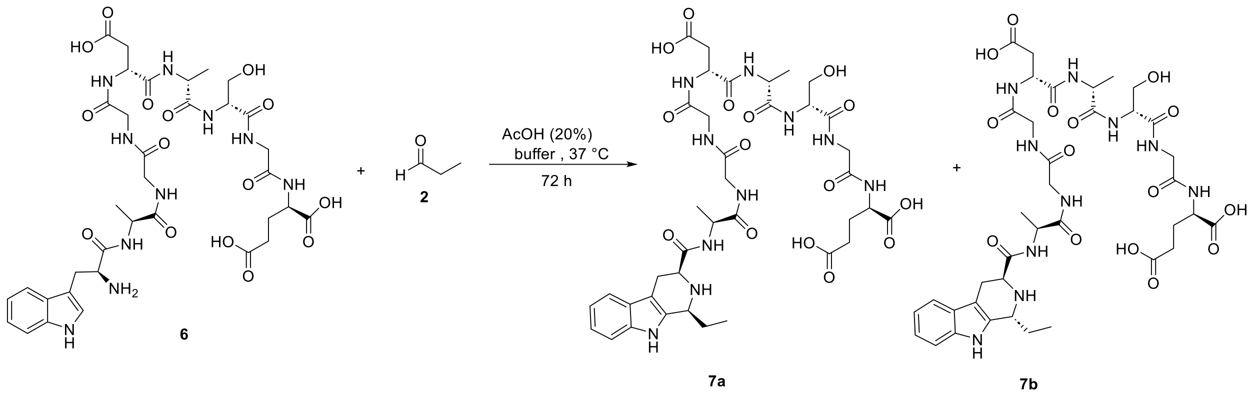

Peptide **6** (2.7 mg, 3.1 µmol) was added to an AcOH (12.5 mM) solution in DPBS buffer (50 µL). Propionaldehyde (5.5 µL, 0.038 mmol) was added to the reaction while stirring at 37 °C. After 72 h, the reaction was analyzed by ^1H NMR to determine a 68% conversion.

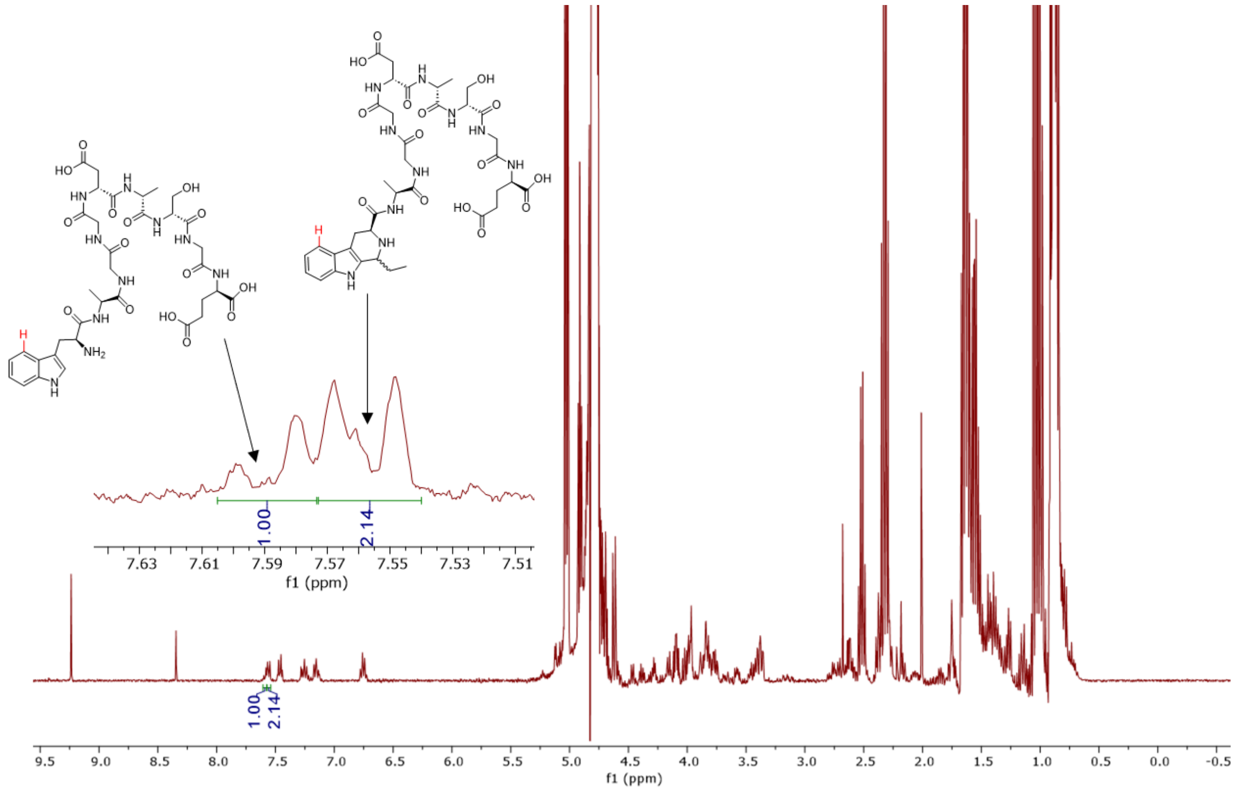

^1H NMR (400 MHz) of the reaction system at t = 72 h. The proton used for analysis is highlighted in red.

5.6 Limitations of the method

5.6.1 Functionalization of tryptamine

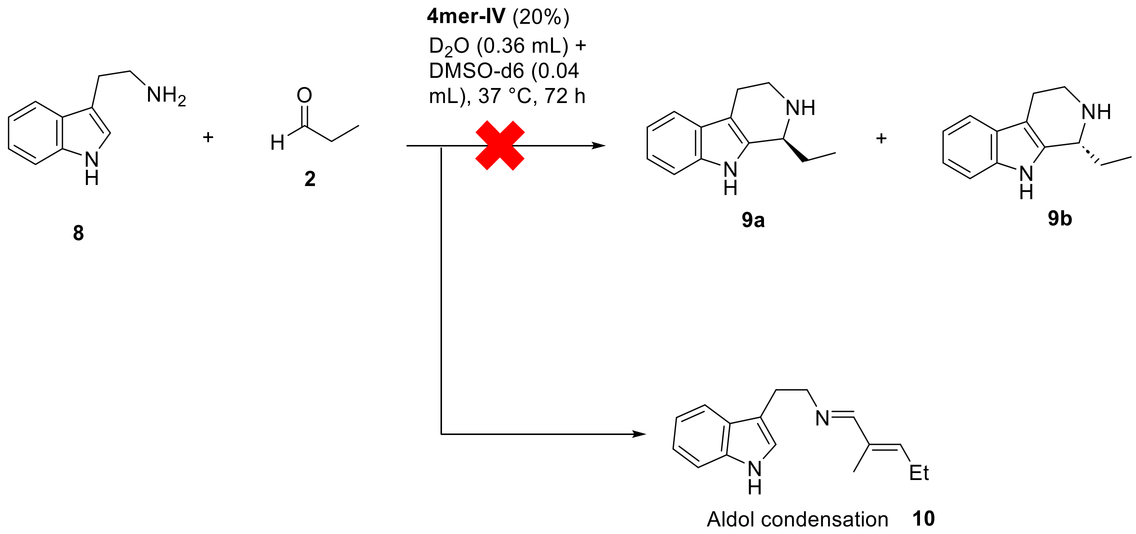

Tryptamine (4.0 mg, 0.025 mmol) and **4mer-IV** (3.6 mg, 0.005 mmol) were dissolved into a mixture of D_2O (0.36 mL) with DMSO-d_6 (0.04 mL, for solubility). Propionaldehyde (22 µL, 0.3 mmol) was added to the reaction while stirring at 37 °C. After 72 h, NMR analysis showed very little conversion. No desired product but only trace amount of aldol condensation side product was isolated. This result is consistent with reported observations.[14]

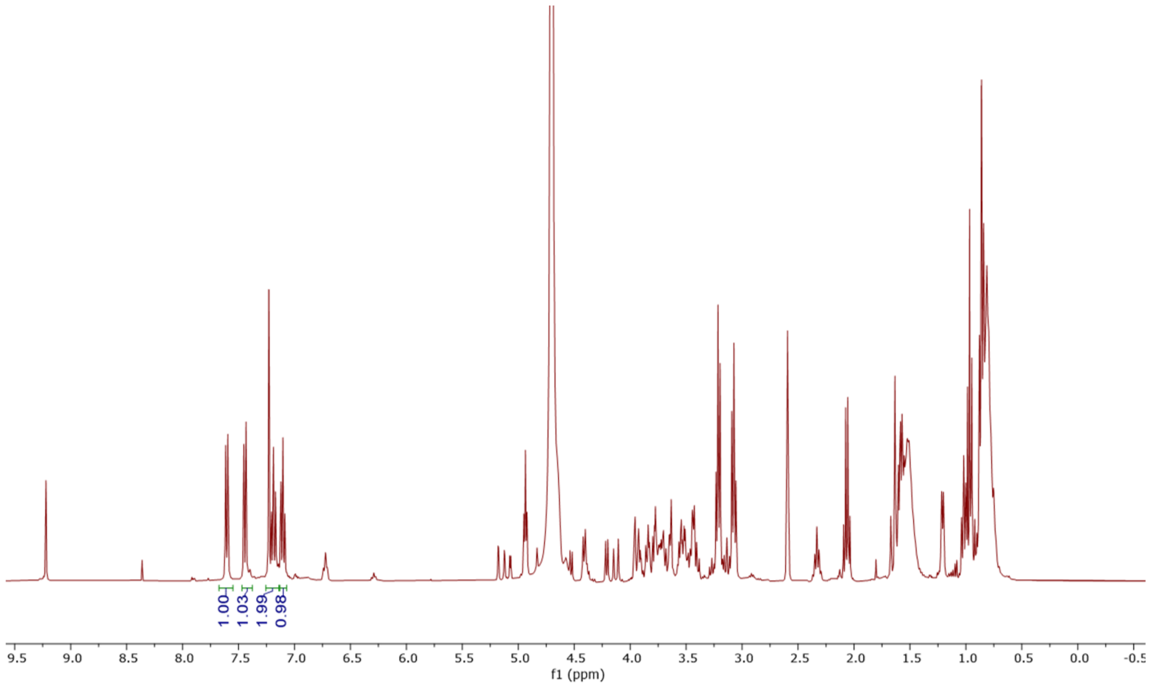

^1H NMR (400 MHz, D_2O) of the reaction system at t = 72 h.

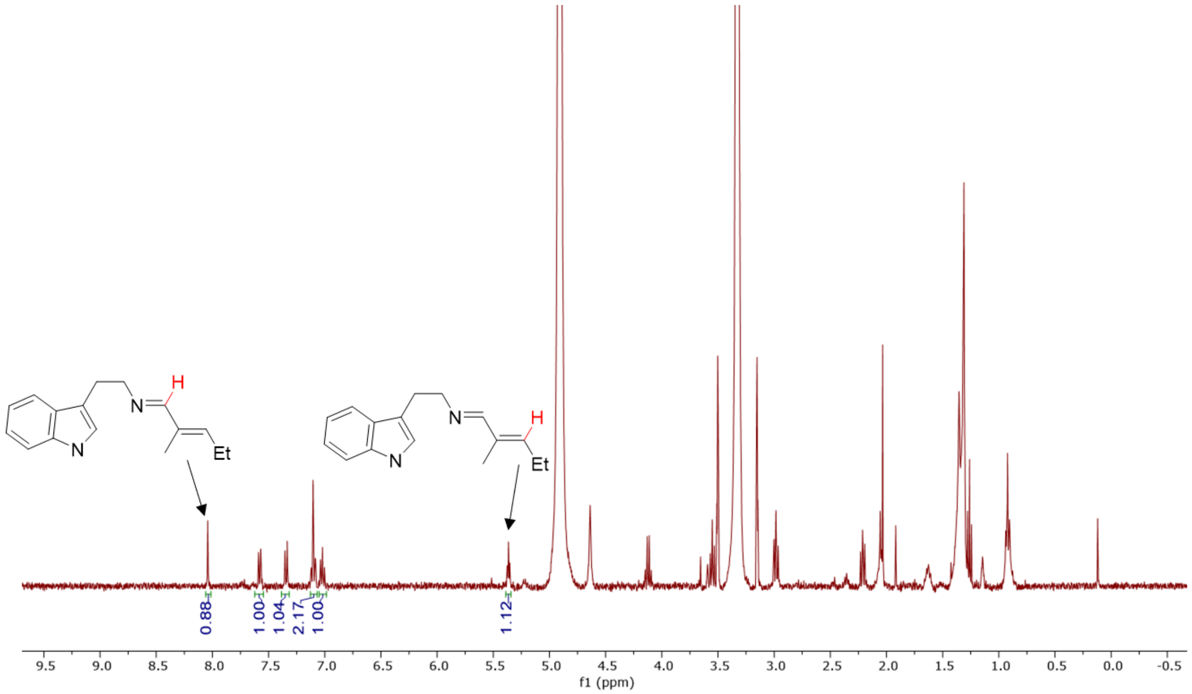

^{1}H NMR (400 MHz, D_2O) of the side product **10**. The protons used for analysis are highlighted in red.

5.6.2 Functionalization of an internal Trp

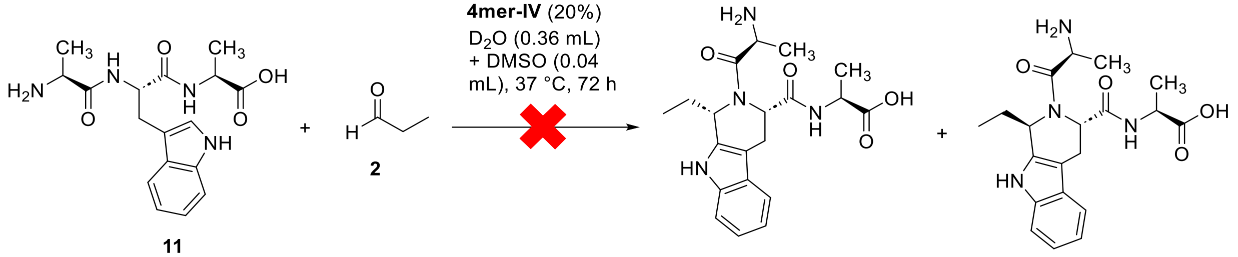

H-Ala-Trp-Ala-OH (8.7 mg, 0.025 mmol) and **4mer-IV** (3.6 mg, 0.005 mmol) were dissolved into D_2O (0.4 mL, containing DMSO (12.5 mM, 0.35 µL) as internal standard). Propionaldehyde (22 µL, 0.3 mmol) was added to the reaction while stirring at 37 °C. After 72 h, NMR analysis didn't show any obvious conversion.

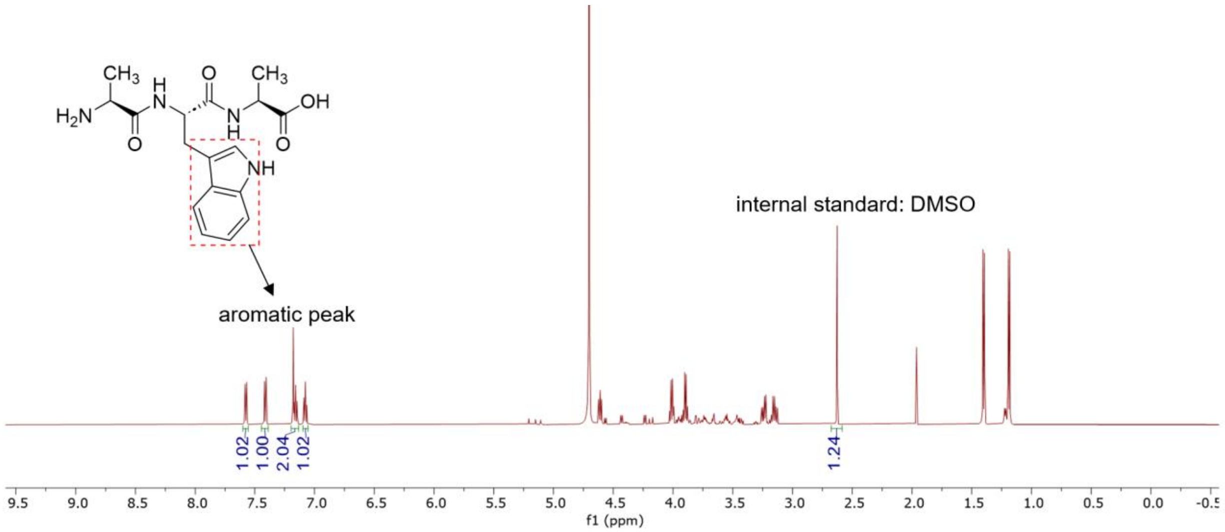

^1H NMR (400 MHz, D_2O) of the reaction system at t = 0 h. The protons used for analysis are highlighted with a red rectangle.

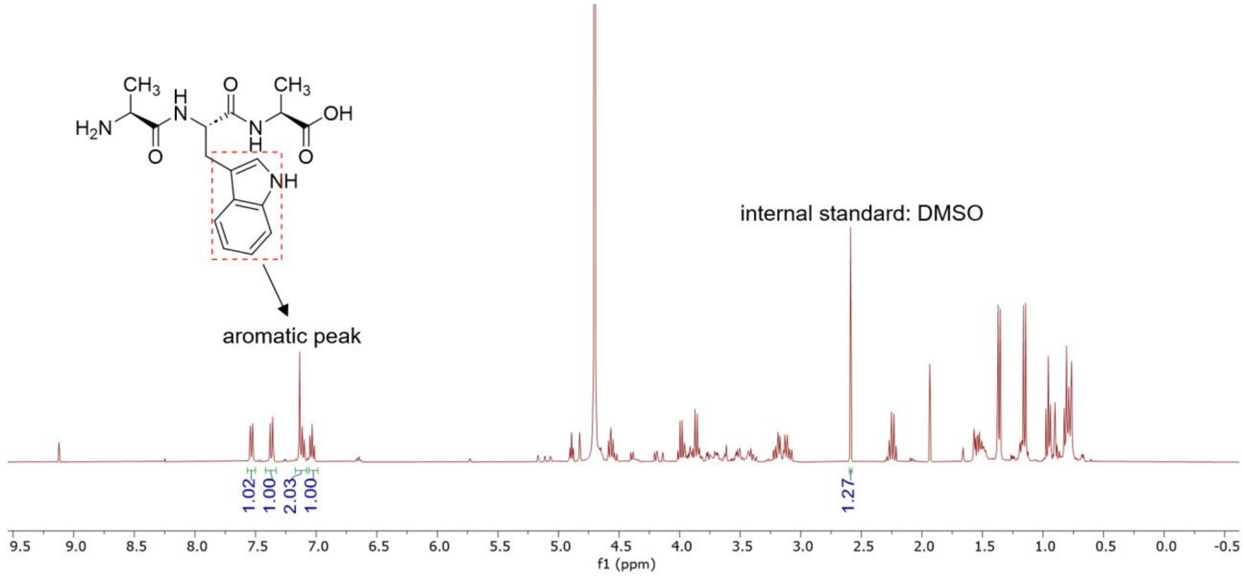

^1H NMR (400 MHz, D_2O) of the reaction system at t = 72 h. The protons used for analysis are highlighted with a red rectangle.

6 References

(1) Eller, S.; Collot, M.; Yin, J.; Hahm, H. S.; Seeberger, P. H. Automated Solid-Phase Synthesis of Chondroitin Sulfate Glycosaminoglycans. *Angew. Chem. Int. Ed.* **2013**, *52* (22), 5858-5861. https://doi.org/10.1002/anie.201210132.

(2) Fittolani, G.; Tyrikos-Ergas, T.; Poveda, A.; Yu, Y.; Yadav, N.; Seeberger, P. H.; Jiménez-Barbero, J.; Delbianco, M. Synthesis of a Glycan Hairpin. *Nat. Chem.* **2023**, *15* (10), 1461-1469. https://doi.org/10.1038/s41557-023-01255-5.

(3) Dallabernardina, P.; Schuhmacher, F.; Seeberger, P. H.; Pfrengle, F. Mixed-Linkage Glucan Oligosaccharides Produced by Automated Glycan Assembly Serve as Tools to Determine the Substrate Specificity of Lichenase. *Chem. – Eur. J.* **2017**, *23* (13), 3191-3196. https://doi.org/10.1002/chem.201605479.

(4) Le Mai Hoang, K.; Pardo-Vargas, A.; Zhu, Y.; Yu, Y.; Loria, M.; Delbianco, M.; Seeberger, P. H. Traceless Photolabile Linker Expedites the Chemical Synthesis of Complex Oligosaccharides by Automated Glycan Assembly. *J. Am. Chem. Soc.* **2019**, *141* (22), 9079-9086. https://doi.org/10.1021/jacs.9b03769.

(5) Gude, M.; Ryf, J.; White, P. D. An Accurate Method for the Quantitation of Fmoc-Derivatized Solid Phase Supports. *Lett. Pept. Sci.* **2002**, *9* (4), 203-206. 10.1023/A:1024148619149.

(6) Hurevich, M.; Kandasamy, J.; Ponnappa, B. M.; Collot, M.; Kopetzki, D.; McQuade, D. T.; Seeberger, P. H. Continuous Photochemical Cleavage of Linkers for Solid-Phase Synthesis. *Org. Lett.* **2014**, *16* (6), 1794-1797. https://doi.org/10.1021/ol500530q.

(7) Kirschner, K. N.; Yongye, A. B.; Tschampel, S. M.; González-Outeiriño, J.; Daniels, C. R.; Foley, B. L.; Woods, R. J. Glycam06: A Generalizable Biomolecular Force Field. Carbohydrates. *J. Comput. Chem.* **2008**, *29* (4), 622-655. https://doi.org/10.1002/jcc.20820.

(8) Mahoney, M. W.; Jorgensen, W. L. A Five-Site Model for Liquid Water and the Reproduction of the Density Anomaly by Rigid, Nonpolarizable Potential Functions. *J. Chem. Phys.* **2000**, *112* (20), 8910-8922. https://doi.org/10.1063/1.481505.

(9) Darden, T.; York, D.; Pedersen, L. Particle Mesh Ewald: An N·Log(N) Method for Ewald Sums in Large Systems. *J. Chem. Phys.* **1993**, *98* (12), 10089-10092. https://doi.org/10.1063/1.464397.

(10) Van Der Spoel, D.; Lindahl, E.; Hess, B.; Groenhof, G.; Mark, A. E.; Berendsen, H. J. C. Gromacs: Fast, Flexible, and Free. *J. Comput. Chem.* **2005**, *26* (16), 1701-1718. https://doi.org/10.1002/jcc.20291.

(11) Evans, D. J.; Holian, B. L. The Nose–Hoover Thermostat. *J. Chem. Phys.* **1985**, *83* (8), 4069-4074. https://doi.org/10.1063/1.449071.

(12) Parrinello, M.; Rahman, A. Polymorphic Transitions in Single Crystals: A New Molecular Dynamics Method. *J. Appl. Phys.* **1981**, *52* (12), 7182-7190. https://doi.org/10.1063/1.328693.

(13) Kuo, F.-M.; Tseng, M.-C.; Yen, Y.-H.; Chu, Y.-H. Microwave Accelerated Pictet–Spengler Reactions of Tryptophan with Ketones Directed toward the Preparation of 1,1-Disubstituted Indole Alkaloids. *Tetrahedron* **2004**, *60* (52), 12075-12084. https://doi.org/10.1016/j.tet.2004.10.025.

(14) Seayad, J.; Seayad, A. M.; List, B. Catalytic Asymmetric Pictet−Spengler Reaction. *J. Am. Chem. Soc.* **2006**, *128* (4), 1086-1087. 10.1021/ja057444l.
